# Supplementary material for: In silico modeling guides identification of novel JAK1 variants associated with immune dysregulation
Source: EMBO Mol Med. 2025 Oct 24;17(12):3275–99. doi: 10.1038/s44321-025-00317-0 (PMC12686074; doi:10.1038/s44321-025-00317-0)
Supplement: Supplementary file 1 — Appendix [file 44321_2025_317_MOESM1_ESM.pdf]

**Appendix**

**Appendix table of contents:**


1. **Appendix Figure S1. Validation of predicted human JAK1 conformations (page 2)**
2. **Appendix Figure S2. Supporting information on structural and energetic consequences of h-JAK1 variants (page 3)**
3. **Appendix Figure S3. *In silico* impact score of JAK1 variants and JAK1 activation loop amino acids conservation across species (page 4)**
4. **Appendix Figure S4. Quantification of immune subsets defined following CyTOF analysis. (pages 5-6)**
5. **Appendix Supplementary Method 1 (pages 7-16)**
6. **Appendix Supplementary case reports (pages 17-19)**

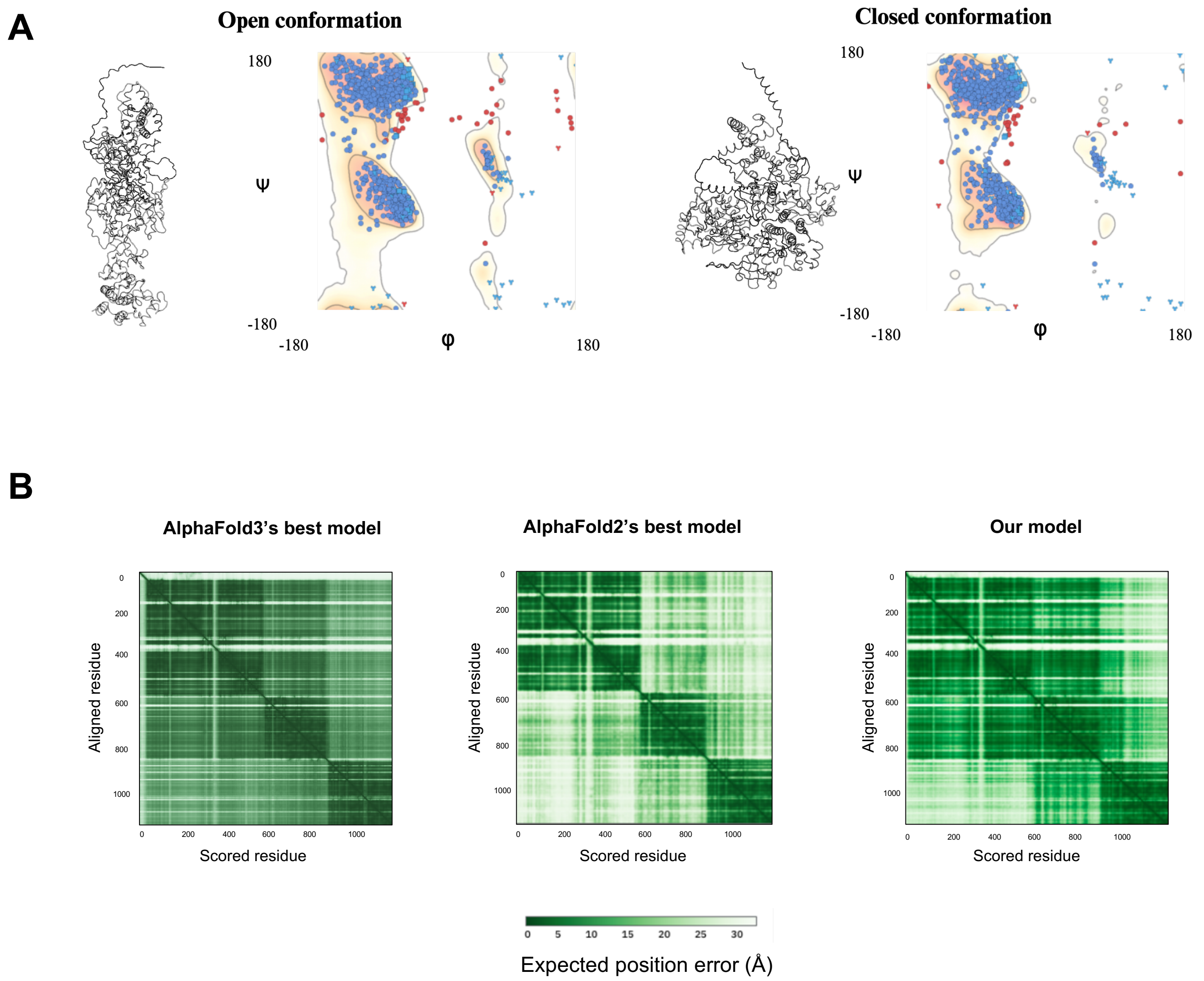


**Appendix Figure S1. Validation of predicted human JAK1 conformations**

**A**. Ramachandran's plots of predicted open (left) and closed conformations (right) allowing to know possible secondary structure divergence between predicted models. Possible Ramachandran's outliers are colored in red.

**B**. Comparison between the AlphaFold3 (left), the AlphaFold2 PDB entry (AF-P23458-F1) structure (midle) and our model (right)**.** The Predicted Aligned Error map illustrates the expected positional error at residue x compared to residue y and is an asymmetric measure. The dark diagonal indicates *x* = *y*. Dark green indicates high confidence, while white indicates low confidence per pair of residues.

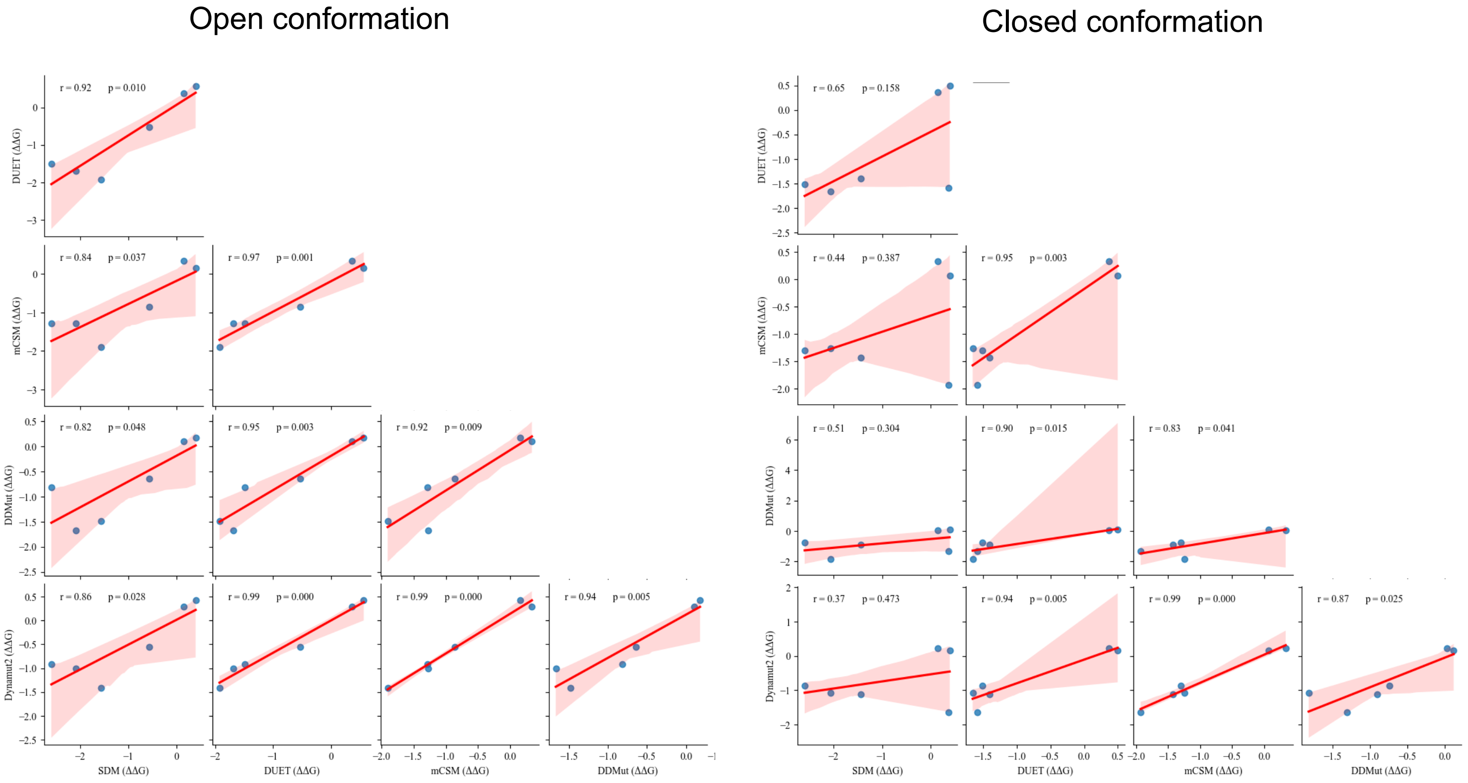


**Appendix Figure S2. Supporting information on structural and energetic consequences of h-JAK1 variants**

Pair plots presenting Pearson correlation analyses across methodologies for ΔΔG predictions. Regression lines are highlighted in red, and their standard deviations are indicated in light red. The Pearson correlation coefficient is displayed at the top of each plot in blue. Left panel corresponds to computations on the open conformation, right panel on the closed conformation.

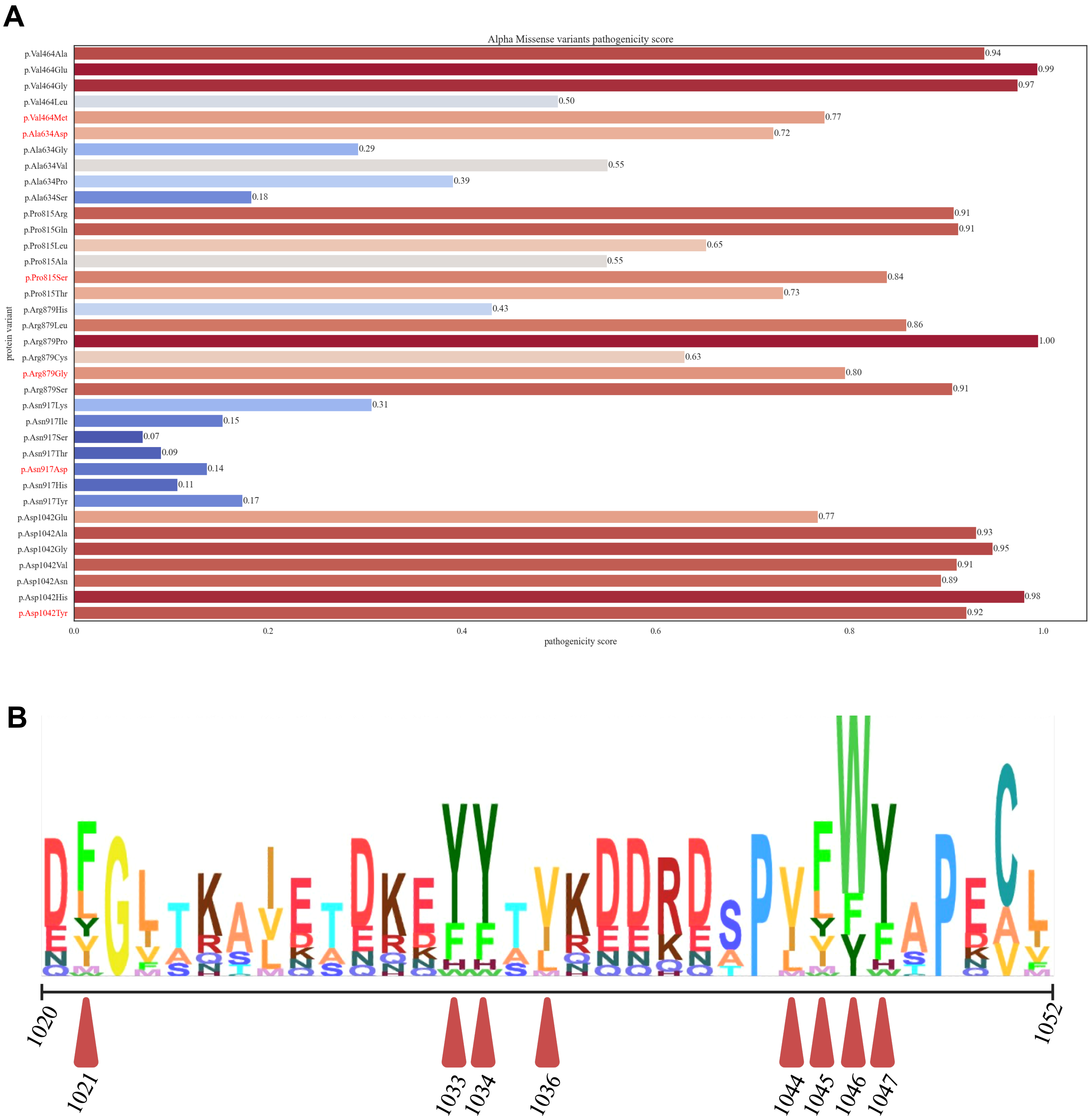


**Appendix Figure S3.** ***In silico*** **impact score of JAK1 variants and JAK1 activation loop amino acids conservation across species**

**A.** Horizontal histogram of retrieved AlphaMissense scores at every mutant localization, colored and annotated by values. Variants identified in this study are depicted in red.

**B.** Sequence logo of JAK1 activation loop consensus sequence. Residues heights are proportional by their frequency and color-coded by type or by residue' contribution to overall conservation score on considered position. A red triangle indicates position of tyrosines.

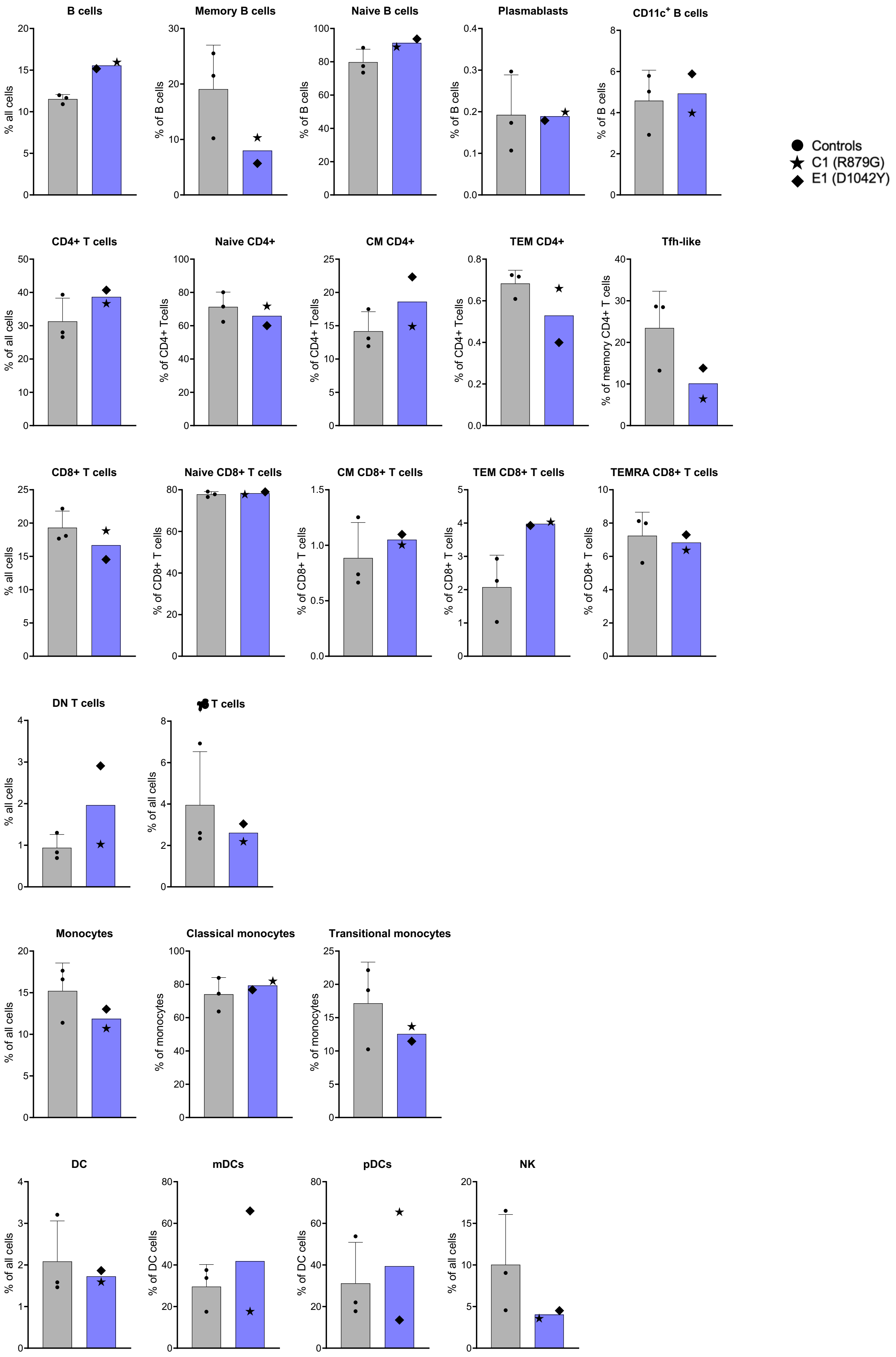
B cells
% all cells
Memory B cells
% of B cells
Naive B cells
% of B cells
Plasmablasts
% of B cells
CD11c+ B cells
% of B cells
Controls
C1 (R879G)
E1 (D1042Y)
CD4+ T cells
% of all cells
Naive CD4+
% of CD4+ Tcells
CM CD4+
% of CD4+ Tcells
TEM CD4+
% of CD4+ Tcells
Tfh-like
% of memory CD4+ T cells
CD8+ T cells
% all cells
Naive CD8+ T cells
% of CD8+ T cells
CM CD8+ T cells
% of CD8+ T cells
TEM CD8+ T cells
% of CD8+ T cells
TEMRA CD8+ T cells
% of CD8+ T cells
DN T cells
% all cells
T cells
% of all cells
Monocytes
% of all cells
Classical monocytes
% of monocytes
Transitional monocytes
% of monocytes
DC
% of all cells
mDCs
% of DC cells
pDCs
% of DC cells
NK
% of all cells

**Appendix Figure S4. Quantification of immune subsets defined following CyTOF analysis.**

Barplots showing the proportion of each identified immune subset across control (grey bars) and JAK1 (blue bars) groups (x-axis) with dots shaped by patient identification. Indicated proportion is the frequency among all singlet viable PBMCs. mDC: myeloid dendritic cell; CM: central memory; EM: effector memory; TEM: terminal effector memory; TEMRA: terminally differentiated effector memory; pDC: plasmacytoid dendritic cell; GD: gamma delta; DN: double negative; NK: natural killer.

**Appendix Supplementary Method 1**

## 1. AlphaFold adaptation for proteic models generation

We hypothesized that JAK1 conformations may correspond to two distinct thermodynamic optima.

To capture this divergence, we modified AlphaFold2 by introducing a penalty term in the loss function and increasing the number of recycling steps. Specifically, a penalty factor of 0.1 was added to the loss function to discourage steric clashes (Invariant Point Attention function), and the number of recycling steps was increased from the default value of 5 to 30 to enhance conformational sampling (see Algorithm 1).

In computational protein modeling, steric clashes often arise as artifacts during conformational sampling due to limitations in energy functions and discrete search spaces (Bouchiba *et al*, 2021).

The penalty term of 0.1 applied to steric clashes in the loss function limits these artifacts by acting as a soft constraint to discourage unrealistic atomic overlaps.

This approach, reducing clash-prone intermediates, enhances the sampling of distinct local energy minima.

This modification was found to enable the selection of two optimal models: one representing the open conformation and the other the closed conformation. The validation of these models is discussed in the results section.

Further analyses, illustrated in the Results section, were performed using Alphafold2's classic implementation to generate individual models for each variant (Jumper *et al*, 2021a).

AlphaFold3 best model was obtained with default parameters (Abramson *et al*, 2024).

### 1.1. Algorithmic details of AlphaFold2 adaptation

In the following pseudocodes detailing the AlphaFold2 adaptation, all function names are inspired by the function names used in the original AlphaFold2 paper (Jumper *et al*, 2021a).

**Algorithm 1** Modified AlphaFold2 part 1 for conformational sampling

```
Require: Protein sequence S, MSA database, structure templates
Ensure: Initial structure prediction with features for refinement
MSA ← BuildMSA(S)
templates ← SearchTemplates(S)
features ← ExtractFeatures(S, MSA, templates)
encoder ← InitializeEvoformer()
decoder ← InitializeStructureModule()
L ← length(S)
representation ← InitializeRepresentation(L)
loss_penalty ← 0.1                                   ▷ Penalty for steric clashes
num_iters ← 30                                       ▷ Increased recycling steps
for i = 1 to num_iters do
    representation ← encoder(features, representation)
    {positions, angles, frames} ← decoder(representation)
    pairwise_distances ← ComputeDistances(positions)
    violations ← CheckStericViolations(positions)
    backbone_loss ← ComputeBackboneLoss(angles, frames)
    distance_loss ← ComputeDistogramLoss(pairwise_distances)
    total_loss ← backbone_loss + distance_loss + (loss_penalty × violations)   ▷ Apply penalty on the loss
    representation ← UpdateRepresentation(total_loss)
end
initial_structure ← {positions, angles, frames}
confidence_metrics ← {PredictpLDDT(representation), PredictTMscore(representation)}
return initial_structure, representation, confidence_metrics
```

After Algorithm 1, AlphaFold2 classic refinement step was used, including relaxation using the AMBER force field and ranking of models (n=10) by confidence metrics (pLDDT, PAE, and predicted TM-score). The final model was selected based on the highest confidence score, and structural domains were scored using per-residue pLDDT values (see Results section).

Predicted Aligned Error were generated using python 3.11 with an in-house script. Ramachandrans plots were generated using Moorhen© (Coot software).

## 2. Semi-supervised K-means clustering of all JAK1 missense variants based on Gower distance

We implemented a semi-supervised clustering approach to classify JAK1 missense variants, using combined feature distances to account for missing data. In fact, many JAK1 missense variants lack annotations or computed scores across all metrics, making traditional clustering approaches suboptimal. To address this, our algorithm incorporated prior knowledge from a small set of 10 labeled variants (see below) to help classify the entire dataset of 21,926 variants. By incorporating a small amount of prior information for imputation and allowing the clustering to be mostly data-driven and unsupervised, this approach minimizes bias while potentially improving the accuracy of the clustering.

### 2.1 Feature selection and preprocessing

We selected 11 features characterizing each variant:

- Evolutionary conservation score from ESM-1b (Brandes *et al*, 2023) (0% missing)
- AlphaMissense pathogenicity score (70.27% missing), see (Cheng *et al*, 2023a).
- $\Delta\Delta G$ stability predictions in closed/open conformations (see Section 3) clipped between -10 and 10, estimated with PyRosetta 4 (Chaudhury *et al*, 2010). (0% missing)
- Open - closed energy change, computed as $\Delta\Delta G_{\text{open}} - \Delta\Delta G_{\text{closed}}$ (0% missing)
- Combined Annotation-Dependent Depletion score (CADD) (Rentzsch *et al*, 2019).
- MutFunc score (Wagih *et al*, 2018) (92.07% missing)
- Sorting Intolerant From Tolerant score (SIFT) (Sim *et al*, 2012) (43.74% missing)
- PolyPhen2 pathogenicity score (Adzhubei *et al*, 2013) (43.74% missing)
- BLOSUM62 substitution matrix score (Eddy, 2004) (43.74% missing)
- Clinical prediction score (ClinPred) (Alirezaie *et al*, 2018) (85.75% missing)

The resulting input was then transformed by computing the Gower distance.

We chose the Gower distance for clustering because it inherently handles missing data without imputation, even if all our training features are numerical. It normalizes feature contributions on a per-observation basis, ensuring no single feature disproportionately influences the clustering due to scale differences or missingness. Additionally, the Gower distance provides a unified metric that adapts to the variability in feature ranges. The Gower distance (Gower, 1971) (or dissimilarity) between two variants *i* and *j* is calculated as:

$$d_{ij} = \frac{\sum_{k=1}^{p} w_k \delta_{ij}^{(k)} d_{ij}^{(k)}}{\sum_{k=1}^{p} w_k \delta_{ij}^{(k)}}$$

where $p$ is the number of features, $w_k$ is the weight assigned to feature k, $\delta_{ij}^{(k)}$ is a binary variable indicating presence (1) or absence (0) of valid values (0 = missingness) for both variants in feature *k*, and $d_{ij}^{(k)}$ is the normalized distance for feature *k*.

**2.2. Implementation semi-supervised clustering with Gower distance**

The clustering was initialized using 10 labeled variants: 5 variants known to be common in the general population (from gnomAD v4: p.S383G, p.I62V, p.I878V, p.T138H, p.S512L (Chen *et al*, 2024)) and 5 variants clinically reported to cause gain-of-function: p.S703I, p.A634D, p.C787F, p.H596D, and p.V985I (Gruber *et al*, 2020), (Bel *et al*, 2017), (Horesh *et al*, 2024), (Takeichi *et al*, 2021), (Fayand *et al*, 2023). These variants served as inital anchors for the two clusters, which we denoted later on as "Cluster 1" and "Cluster 2". The algorithm then used these seed clusters to classify the remaining 21,916 JAK1 missense variants.

**Algorithm 2** Semi-supervised clustering with Gower distance

```
Data: Feature matrix X of size 21926 * 11, set of 5 gnomAD variants S_G, set of 5 gain-of-function variants
      S_GOF
Result: Assignment of each variant to either cluster 1 or cluster 2
features ← {ESMb1_score, AlphaMissense_score, ΔΔG_open − closed,
           ΔΔG_closed, ΔΔG_open, CADD, Mutfunc_score,
           SIFT_score, PolyPhen2_score, BLOSUM62, ClinPred}
n ← |X|                                                        ▷ Number of variants
 mask ← ∅                                                      ▷ Initialize mask
foreach v ∈ S_G do
 | mask[v] ← 1
end
foreach v ∈ S_GOF do
 | mask[v] ← 2
end
D ← ComputeGowerDistance(X, features)                          ▷ Compute Gower distance matrix
cluster_centers ← InitializeClusterCentersX, mask)
assignments ← InitializeArray(n)                               ▷ Initialize assignments
num_iterations ← 10
converged ← False
iteration ← 0
old_assignments ← 0
while iteration < num_iterations and not converged do
 | for i = 1 to n do
 |  | if i ∈ mask then
 |  |  | assignments[i] ← mask[i]
 |  | end
 |  | else
 |  |  | d_1 ← GowerDistance(X[i], cluster_centers[1])
 |  |  | d_2 ← GowerDistance(X)[i], cluster_centers[2])
 |  |  | if d_1 < d_2 then
 |  |  |  | assignments[i] ← 1
 |  |  | end
 |  |  | else
 |  |  |  | assignments[i] ← 2
 |  |  | end
 |  | end
 | end
 | for c = 1 to 2 do
 |  | cluster_centers[c] ← ComputeClusterCenter(X, assignments, c)
 | end
 | changed ← SumOfDifferences(assignments, old_assignments)
 | if changed = 0 then
 |  | converged ← True
 | end
 | iteration ← iteration +1
 | old_assignments ← assignments
end
silhouette ← ComputeSilhouetteScore(X), assignments, D)
cluster_means ← ComputeClusterFeatureMeans(X, assignments)
return assignments, cluster_centers, silhouette, cluster_means
```

### 3. Thermodynamic modeling.

The thermodynamic stability of a protein is given by the Gibbs free energy for the reaction of protein folding,

$$\Delta G = \Delta G_{\text{folded}} - \Delta G_{\text{unfolded}} = -RT \ln\left(\frac{[\text{folded}]}{[\text{unfolded}]}\right)$$

, where $\Delta G < 0$ is required for folding stability, Figure 1. $\Delta G$ is a fundamental physical quantity collectively determined by the intramolecular interactions between amino acid

residues within the protein as well as the interactions between the protein and the physiological environment around it. The influence of mutations on protein thermostability, denoted as $\Delta\Delta\mathrm{G_m}$, is usually defined as the change in $\Delta G$ between mutant and wild type proteins, $\Delta\Delta\mathrm{G_m} = \Delta\mathrm{G_{WT}} - \Delta\mathrm{G_m}$. Hence,

- If $\Delta\Delta\mathrm{G_m} < 0$, the mutation is destabilizing (Figure 1).
- If $\Delta\Delta\mathrm{G_m} > 0$, the mutation is stabilizing.

Now, the same reasoning can be applied to different alternative protein structures such as open and closed configurations, where the open configuration is, for instance, favored under protein phosphorylation. In this setting, the stabilities of the open (o) and closed (c) states can be differently altered upon mutation (m). In particular, $\Delta\Delta\mathrm{G_{o,m}} > \Delta\Delta\mathrm{G_{c,m}}$ corresponds to a stronger stabilization (or weaker destabilization) of the open versus closed states upon mutation, leading to more open configurations in mutants as compared to wild-type.

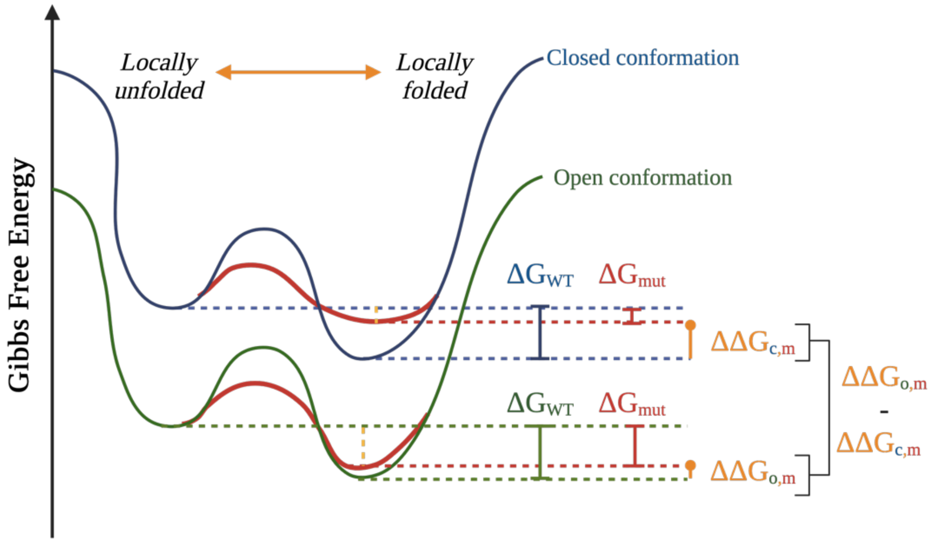


Figure 1. Schematic representation of reported values in the results section.

### 3.2. $\Delta\Delta\mathrm{G}$ quantification

The thermodynamic stability shifts $\Delta\Delta\mathrm{G}$ for JAK1 variants was first estimated with PyRostta 4 due to its computational efficiency (Chaudhury *et al*, 2010). Then, thermodynamic stability were more diversely computed using SDM (Pandurangan *et al*, 2017), Cutoff Scanning Matrix (mCSM) (Pires *et al*, 2014a), DUET (Pires *et al*,

2014b), DDMut (Zhou *et al*, 2023) and DynaMut2 (Rodrigues *et al*, 2021) tools. Pearson correlation analysis was performed on python 3.11.

## 4. In silico structural analysis

### 4.1. Mutational analysis on sequential data

All AlphaMissence (Cheng *et al*, 2023a) predicted variants were retrieved for each position with associated pathogenicity score according to AlphaMissence's github implementation (Cheng *et al*, 2023b). Sequence homologs of JAK1 were search with HMMER(Johnson *et al*, 2010). Subsequent Multiple Sequence Alignement file for Homo sapiens, Pan troglodytes, Pan paniscus, Mus musculus, Rattus novergicus, and Bos taurus were aligned with Muscle (Edgar, 2004) and plotted on python 3.11 with pyMSAviz (Shimoyama, 2022). Sequence logo for residues was generated with Skylign (Wheeler *et al*, 2014).

### 4.2. Protein map similarities assessment

To assess structural similarities, we aligned our model and AlphaFold2-predicted JAK1 model against the m-JAK1 electron microscopy map (Caveney *et al*, 2023) using ChimeraX (v1.8) (Meng *et al*, 2023). The fitmap function was applied iteratively for both AlphaFold2 (AF-P23458-F1)(Jumper *et al*, 2021b; Varadi *et al*, 2022) and our open-state JAK1 model to compute corresponding map correlation between a model and the cryoEM ground truth map. We applied the same procedure to JAK1 structure predicted by AlphaFold3 (Abramson *et al*, 2024). Root Mean Square Deviation between models was always assessed by using ChimeraX function (version 1.8).

### 4.3. Visualization

Snapshots of JAK1 were extracted from ChimeraX sessions (version 1.8)(Meng *et al*, 2023) and Mol* (Sehnal *et al*, 2021). Interactions were modeled in a 3.5Å distance. Predicted Aligned Error for predicted models and Ramachandran plots were generated using python 3.11 with in-house scripts.

## 5. Docking

### 5.1. ADP molecular docking

ADP molecular docking was performed on the JAK1 kinase domain with AutoDock Vina (Trott & Olson, 2010) over 50 iterations on both open and closed conformations.

Site-pocket reference of reported h-JAK1 complexed with ADP 5KHW (Caspers *et al*, 2016) model was used for confirmation of AutoDock Vina best pose on JAK1 open conformation. 5KHW model was not used itself as it lacks report of position N917.

### 5.2. Evaluation of ligand affinity perturbation

Predicted affinity perturbation on ADP binding upon mutation was evaluated on docked ADP with mCSM-lig (Pires *et al*, 2016) with a putative wild-type affinity of 0.100 nM that was previously reported (Murphy *et al*, 2014; Babon *et al*, 2012).

## References


Abramson J, Adler J, Dunger J, Evans R, Green T, Pritzel A, Ronneberger O, Willmore L, Ballard AJ, Bambrick J, *et al* (2024) Accurate structure prediction of biomolecular interactions with AlphaFold 3. *Nature* 630: 493–500

Adzhubei I, Jordan DM & Sunyaev SR (2013) Predicting Functional Effect of Human Missense Mutations Using PolyPhen-2. *Curr Protoc Hum Genet Editor Board Jonathan Haines Al* 0 7: Unit7.20

Alirezaie N, Kernohan KD, Hartley T, Majewski J & Hocking TD (2018) ClinPred: Prediction Tool to Identify Disease-Relevant Nonsynonymous Single-Nucleotide Variants. *Am J Hum Genet* 103: 474–483

Babon JJ, Kershaw NJ, Murphy JM, Varghese LN, Laktyushin A, Young SN, Lucet IS, Norton RS & Nicola NA (2012) Suppression of Cytokine Signaling by SOCS3: Characterization of the Mode of Inhibition and the Basis of Its Specificity. *Immunity* 36: 239–250

Bel KLD, Ragotte RJ, Saferali A, Lee S, Vercauteren SM, Mostafavi SA, Schreiber RA, Prendiville JS, Phang MS, Halparin J, *et al* (2017) JAK1 gain-of-function causes an autosomal dominant immune dysregulatory and hypereosinophilic syndrome. *J Allergy Clin Immunol* 139: 2016-2020.e5

Bouchiba Y, Cortés J, Schiex T & Barbe S (2021) Molecular flexibility in computational protein design: an algorithmic perspective. *Protein Eng Des Sel* 34: gzab011

Brandes N, Goldman G, Wang CH, Ye CJ & Ntranos V (2023) Genome-wide prediction of disease variant effects with a deep protein language model. *Nat Genet* 55: 1512–1522

Caspers NL, Han S, Rajamohan F, Hoth LR, Geoghegan KF, Subashi TA, Vazquez ML, Kaila N, Cronin CN, Johnson E, *et al* (2016) Development of a high-throughput

crystal structure-determination platform for JAK1 using a novel metal-chelator soaking system. *Acta Crystallogr Sect F Struct Biol Commun* 72: 840–845

Caveney NA, Saxton RA, Waghray D, Glassman CR, Tsutsumi N, Hubbard SR & Garcia KC (2023) Structural basis of Janus kinase trans-activation. *Cell Rep* 42

Chaudhury S, Lyskov S & Gray JJ (2010) PyRosetta: a script-based interface for implementing molecular modeling algorithms using Rosetta. *Bioinformatics* 26: 689–691

Chen S, Francioli LC, Goodrich JK, Collins RL, Kanai M, Wang Q, Alföldi J, Watts NA, Vittal C, Gauthier LD, *et al* (2024) A genomic mutational constraint map using variation in 76,156 human genomes. *Nature* 625: 92–100

Cheng J, Novati G, Pan J, Bycroft C, Žemgulytė A, Applebaum T, Pritzel A, Wong LH, Zielinski M, Sargeant T, *et al* (2023a) Accurate proteome-wide missense variant effect prediction with AlphaMissense. *Science* 381: eadg7492

Cheng J, Novati G, Pan J, Bycroft C, Žemgulytė A, Applebaum T, Pritzel A, Wong LH, Zielinski M, Sargeant T, *et al* (2023b) Accurate proteome-wide missense variant effect prediction with AlphaMissense. *Science* 381: eadg7492

Eddy SR (2004) Where did the BLOSUM62 alignment score matrix come from? *Nat Biotechnol* 22: 1035–1036

Edgar RC (2004) MUSCLE: a multiple sequence alignment method with reduced time and space complexity. *BMC Bioinformatics* 5: 113

Fayand A, Hentgen V, Posseme C, Lacout C, Picard C, Moguelet P, Cescato M, Sbeih N, Moreau TRJ, Zhu YYJ, *et al* (2023) Successful treatment of JAK1-associated inflammatory disease. *J Allergy Clin Immunol* 152: 972–983

Gower JC (1971) A General Coefficient of Similarity and Some of Its Properties. *Biometrics* 27: 857

Gruber CN, Calis JJA, Buta S, Evrony G, Martin JC, Uhl SA, Caron R, Jarchin L, Dunkin D, Phelps R, *et al* (2020) Complex Autoinflammatory Syndrome Unveils Fundamental Principles of JAK1 Kinase Transcriptional and Biochemical Function. *Immunity* 53: 672-684.e11

Horesh ME, Martin-Fernandez M, Gruber C, Buta S, Le Voyer T, Puzenat E, Lesmana H, Wu Y, Richardson A, Stein D, *et al* (2024) Individuals with JAK1 variants are affected by syndromic features encompassing autoimmunity, atopy, colitis, and dermatitis. *J Exp Med* 221: e20232387

Johnson LS, Eddy SR & Portugaly E (2010) Hidden Markov model speed heuristic and iterative HMM search procedure. *BMC Bioinformatics* 11: 431

Jumper J, Evans R, Pritzel A, Green T, Figurnov M, Ronneberger O, Tunyasuvunakool K, Bates R, Žídek A, Potapenko A, *et al* (2021a) Highly accurate protein structure prediction with AlphaFold. *Nature* 596: 583–589

Jumper J, Evans R, Pritzel A, Green T, Figurnov M, Ronneberger O, Tunyasuvunakool K, Bates R, Žídek A, Potapenko A, *et al* (2021b) Highly accurate protein structure prediction with AlphaFold. *Nature* 596: 583–589

Meng EC, Goddard TD, Pettersen EF, Couch GS, Pearson ZJ, Morris JH & Ferrin TE (2023) UCSF ChimeraX: Tools for structure building and analysis. *Protein Sci* 32: e4792

Murphy JM, Zhang Q, Young SN, Reese ML, Bailey FP, Eyers PA, Ungureanu D, Hammaren H, Silvennoinen O, Varghese LN, *et al* (2014) A robust methodology to subclassify pseudokinases based on their nucleotide-binding properties. *Biochem J* 457: 323–334

Pandurangan AP, Ochoa-Montaño B, Ascher DB & Blundell TL (2017) SDM: a server for predicting effects of mutations on protein stability. *Nucleic Acids Res* 45: W229–W235

Pires DEV, Ascher DB & Blundell TL (2014a) mCSM: predicting the effects of mutations in proteins using graph-based signatures. *Bioinformatics* 30: 335–342

Pires DEV, Ascher DB & Blundell TL (2014b) DUET: a server for predicting effects of mutations on protein stability using an integrated computational approach. *Nucleic Acids Res* 42: W314–W319

Pires DEV, Blundell TL & Ascher DB (2016) mCSM-lig: quantifying the effects of mutations on protein-small molecule affinity in genetic disease and emergence of drug resistance. *Sci Rep* 6: 29575

Rentzsch P, Witten D, Cooper GM, Shendure J & Kircher M (2019) CADD: predicting the deleteriousness of variants throughout the human genome. *Nucleic Acids Res* 47: D886–D894

Rodrigues CHM, Pires DEV & Ascher DB (2021) DynaMut2: Assessing changes in stability and flexibility upon single and multiple point missense mutations. *Protein Sci Publ Protein Soc* 30: 60–69

Sehnal D, Bittrich S, Deshpande M, Svobodová R, Berka K, Bazgier V, Velankar S, Burley SK, Koča J & Rose AS (2021) Mol* Viewer: modern web app for 3D visualization and analysis of large biomolecular structures. *Nucleic Acids Res* 49: W431–W437

Shimoyama Y (2022) pyMSAviz: MSA visualization python package for sequence analysis.

Sim N-L, Kumar P, Hu J, Henikoff S, Schneider G & Ng PC (2012) SIFT web server: predicting effects of amino acid substitutions on proteins. *Nucleic Acids Res* 40: W452–W457

Takeichi T, Lee JYW, Okuno Y, Miyasaka Y, Murase Y, Yoshikawa T, Tanahashi K, Nishida E, Okamoto T, Ito K, *et al* (2021) Autoinflammatory Keratinization Disease With Hepatitis and Autism Reveals Roles for JAK1 Kinase Hyperactivity in Autoinflammation. *Front Immunol* 12: 737747

Trott O & Olson AJ (2010) AutoDock Vina: Improving the speed and accuracy of docking with a new scoring function, efficient optimization, and multithreading. *J Comput Chem* 31: 455–461

Varadi M, Anyango S, Deshpande M, Nair S, Natassia C, Yordanova G, Yuan D, Stroe O, Wood G, Laydon A, *et al* (2022) AlphaFold Protein Structure Database: massively expanding the structural coverage of protein-sequence space with high-accuracy models. *Nucleic Acids Res* 50: D439–D444

Wagih O, Galardini M, Busby BP, Memon D, Typas A & Beltrao P (2018) A resource of variant effect predictions of single nucleotide variants in model organisms. *Mol Syst Biol* 14: e8430

Wheeler TJ, Clements J & Finn RD (2014) Skylign: a tool for creating informative, interactive logos representing sequence alignments and profile hidden Markov models. *BMC Bioinformatics* 15: 7

Zhou Y, Pan Q, Pires DEV, Rodrigues CHM & Ascher DB (2023) DDMut: predicting effects of mutations on protein stability using deep learning. *Nucleic Acids Res* 51: W122–W128

**Appendix Supplementary case report. Complete clinical description for the five patients with *JAK1* GOF variant.**

**Family A:**

**A1:**

Patient A1 was diagnosed with selective IgA deficiency, short stature and type-1 autoimmune hepatitis at 12 years of age. She was then on prolonged immunosuppression with glucocorticoids and Azathioprine. During the liver disease, she developed portal hypertension, splenomegaly with pathological elastography that progresses to liver cirrhosis, without digestive bleeding or esophageal varices. Due to the progressive deterioration of the liver disease, she was hospitalized for a liver transplant that was performed in June 2023, with a good progression to date. During her illness, she developed rosacea and chalazion as inflammatory complications. The patient also presented with cellulitis of the thigh of unknown origin. Biological workup evidenced global lymphopenia in the context of the disease and immunosuppression. Lymphocytes phenotype showed normal relative values with the presence of naïve T cells. She presented hypergammaglobulinemia (IgM and IgG) and a good response to vaccine polysaccharide and protein antigens. Considering the IgA deficiency, autoimmunity (HAI T1), and inflammatory component, she was genetically studied by WES. The genetic results revealed a heterozygous variant in *JAK1* (c.1390G/A; p.V464M). The asymptomatic father carried the same mutation.

**Family B:**

**B1:**

Patient B1 presented at 2 month of life with severe eczema and atopic dermatitis and failure to thrive. At 10 years of age he presented with arthralgia, dyspnea, and Raynaud's phenomenon. Additionally, the main feature was necrotizing vasculitis in the lower and upper extremities, with acral predominance, and his skin exhibited sclerosis. This clinical picture involved biochemical inflammatory and the presence of autoantibodies.  Treatment with steroids and cyclophosphamide showed a partial response. WES revealed a heterozygous variant in the *JAK1* gene (c.2443C/T; p.P815S). Currently, the patient is under treatment with tofacitinib at a dose of 4 mg every 12 hours orally. At the last visit, he was clinically quiescent with normalized biochemical parameters.

**Family C:**

**C1:**

Patient C1 presented at 15-year-old with a medical history significant for late-onset cord separation, followed by early development of dermatosis characterized by prurigo nodularis, and an episode of severe lymphoproliferation marked by generalized lymphadenopathy at age 13. At 15 years of age, the patient experienced a prolonged hospitalization due to a psoas-iliac abscess accompanied by severe systemic inflammation. Laboratory findings indicated persistent fever, with a C-reactive protein (CRP) level of 209 mg/L, a procalcitonin (PCT) level of 0.33 ng/mL, and elevated transaminase levels (AST 162 U/L; ALT 86 U/L). The patient also presented with proteinuria (5.17 mg/m²/h), hypoalbuminemia (23.6 g/L), and polyserositis, which included moderate ascitis and pleural effusion. The presentation necessitated treatment with intravenous antibiotics and corticosteroids. Currently, the patient is clinically stable, although she continues to experience significant prurigo nodularis and perniosis. Initial treatment with tofacitinib at a dose of 5 mg twice daily was ineffective; however, an escalation of the dose to 5 mg four times daily resulted in noticeable improvement in her condition. Due to the atypic presentation, a comprehensive 485 Primary Immunodeficiences NGS panel sequencing was performed and revealed a heterozygous JAK1 variant (c.2635 C>G; p.R879G).

**Family D:**

**D1:**

Patient D1 presented at the age of 6 months with a transient episode of pancytopenia, interpreted as bone marrow arrest secondary to an infectious process. At 2 years of age, the patient developed pneumonia, although no microbiological agent was isolated. A year later, the patient was hospitalized with symptoms evocating bacteremia, again without microbial isolation. At 5 years old, the patient presented with an adenophlegmon requiring both antibiotic therapy and surgical debridement. Prior to this, insect bites had caused episodes of prurigo, skin infections, and mild transient atopic dermatitis. Additionally, some retained primary teeth required dental intervention. At the age of 9, the patient developed a paravertebral infection imputed to *Staphylococcus aureus*. At this time, clinical evaluation revealed hyperextensive

joints, eosinophilia, and markedly elevated levels of serum IgE, with normal levels of IgG, IgA, and IgM. With a presumptive diagnosis of Hyper-IgE Syndrome (HIES), initial immunological investigations documented a normal percentage of Th17 cells, and no pathogenic variants were identified in STAT3 or CARD11. Further genetic testing via WES revealed a heterozygous variant (c.2749A/G, p.N917D) in the *JAK1* gene. As of the latest follow-up, the patient self-reports overall good health and well-being, with only mild atopic manifestations and persistent splenomegaly, managed without specific treatment.

**Family E:**

**E1:**

Patient E1 was born at 37 weeks gestation via emergency cesarean section due to cardio-fetal rhythm anomalies, with a diagnosis of moderate intrauterine growth restriction. Early childhood was marked by developmental and language impairments, associated with intellectual disabilities. Phenotypically, the patient exhibited prominent ears, a long face, arachnodactyly (characterized by particularly long and slender fingers), and moderate joint hyperlaxity. Between the ages of 2 and 3, the patient developed severe vitiligo, and enamel dysplasia, resulting in atypically pointed teeth in both primary and permanent dentition. Alopecia developed during adolescence. The family history was significant, as a maternal aunt’s child died of sudden infant death syndrome at three months of age, and two relatives had developmental delays, though they were not formally evaluated. At the age of 12, the patient presented with ataxia and oculomotor apraxia type 2, with MRI findings of cerebellar cortico-subcortical atrophy. A targeted panel sequencing identified a homozygous mutation in the SETX gene. At 13 years old, the patient presented to the Intensive Care Unit (ICU) with hemodynamic instability, manifested by severe hyponatremia and hyperkalemia. Further evaluation confirmed adrenal insufficiency with low cortisolaemia, and positive anti-21-hydroxylase antibodies, pointing to an autoimmune cause of adrenal insufficiency and leading to a diagnosis of Addison's disease. WES identified a heterozygous variant (c.3124G/T; p.D1042Y) in the *JAK1* gene. Extensive evaluation for biological autoimmunity was largely negative, except for positive anti-nuclear antibodies (1/160) and positive anti-ENA antibodies with anti-Ro/SS-A positivity. Treatment with hydrocortisone and fludrocortisone led to gradual clinical improvement.
