# Supplementary material for: In silico modeling guides identification of novel JAK1 variants associated with immune dysregulation
Source: EMBO Mol Med. 2025 Oct 24;17(12):3275–99. doi: 10.1038/s44321-025-00317-0 (PMC12686074; doi:10.1038/s44321-025-00317-0)
Supplement: Supplementary file 4 — Movie EV1 [file 44321_2025_317_MOESM4_ESM.zip › Movie_EV1/Legend Movie EV1.docx]

**Movie EV1: Open JAK1 conformation (AlphaFold3) on dimerized m-JAK1 cryo-EM map**

Superimposed structure of dimerized m-JAK1 cryo-EM map and AlphaFold3 best model
