# Supplementary material for: In silico modeling guides identification of novel JAK1 variants associated with immune dysregulation
Source: EMBO Mol Med. 2025 Oct 24;17(12):3275–99. doi: 10.1038/s44321-025-00317-0 (PMC12686074; doi:10.1038/s44321-025-00317-0)
Supplement: Supplementary file 5 — Movie EV2 [file 44321_2025_317_MOESM5_ESM.zip › Movie_EV2/Legend Movie EV2.docx]

**Movie EV2: Open JAK1 conformation (AlphaFold2) on dimerized m-JAK1 cryo-EM map**

Superimposed structure of dimerized m-JAK1 cryo-EM map and AlphaFold2 best model
