# Supplementary material for: In silico modeling guides identification of novel JAK1 variants associated with immune dysregulation
Source: EMBO Mol Med. 2025 Oct 24;17(12):3275–99. doi: 10.1038/s44321-025-00317-0 (PMC12686074; doi:10.1038/s44321-025-00317-0)
Supplement: Supplementary file 6 — Movie EV3 [file 44321_2025_317_MOESM6_ESM.zip › Movie_EV3/Legend Movie EV3.docx]

**Movie EV3: Open JAK1 conformation (our model) on dimerized m-JAK1 cryo-EM map**

Superimposed structure of dimerized m-JAK1 cryo-EM map and our model
