# Supplementary material for: In silico modeling guides identification of novel JAK1 variants associated with immune dysregulation
Source: EMBO Mol Med. 2025 Oct 24;17(12):3275–99. doi: 10.1038/s44321-025-00317-0 (PMC12686074; doi:10.1038/s44321-025-00317-0)
Supplement: Supplementary file 8 — Source data Fig. 3 [file 44321_2025_317_MOESM8_ESM.zip › Figure 3/Replicates Fig.3A/n = 1/pSTAT1 quantif.pdf]

Image Report: pSTAT1 quantif

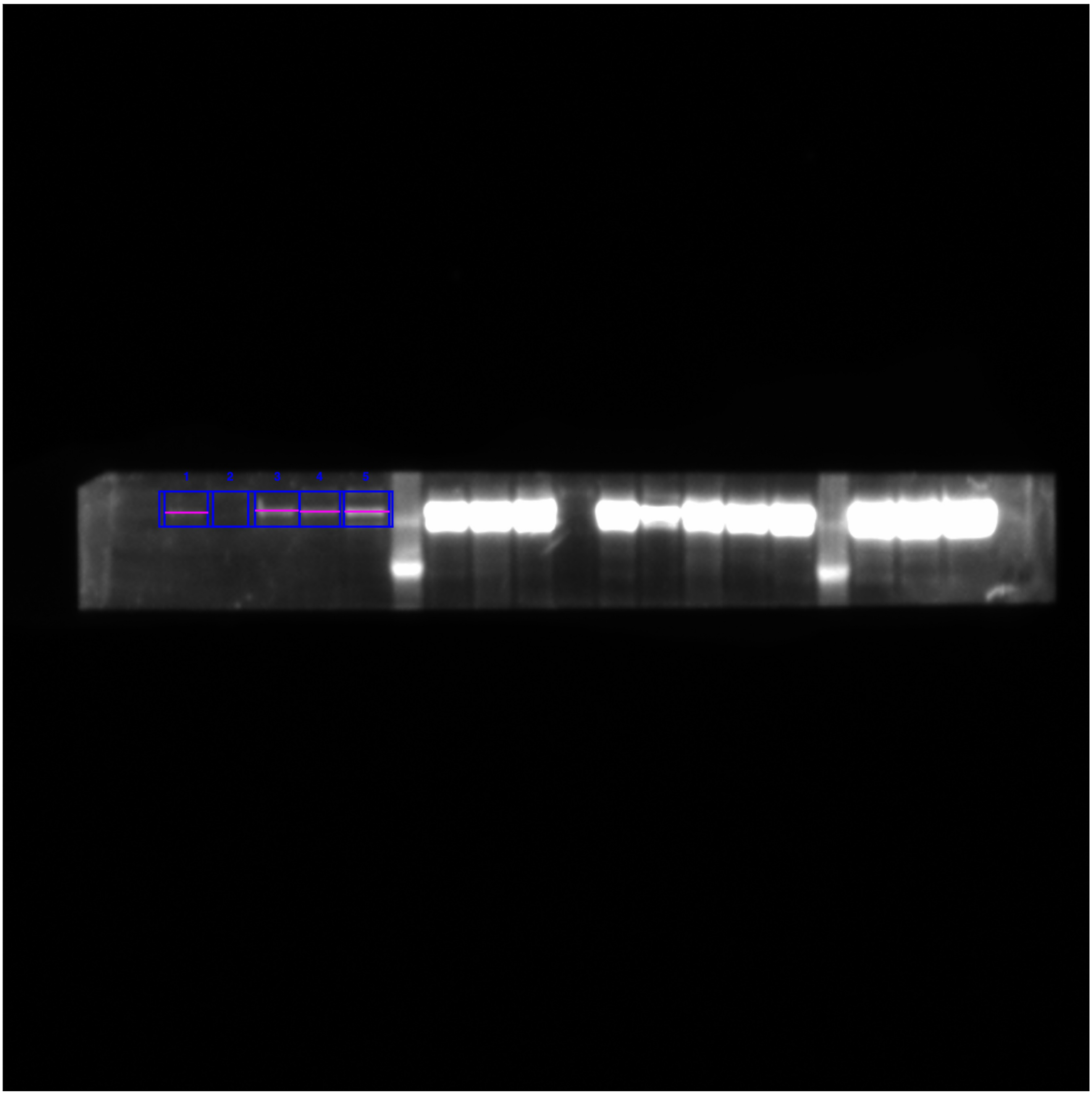

/Volumes/FRL-lab/FRL's Team/Marie Jeanpierre/JAK1/Papier JAK1/Nouvelle submission EMBO/ Source data WB new depot/Quantification Fig.3A/n = 1/pSTAT1 quantif.scn

Acquisition Information

Image Information

Acquisition Date	unknown
User Name	Marie Jeanpierre

Image Area (mm)	X: 15.2 Y: 15.2
Pixel Size (µm)	X: 14.1 Y: 14.1
Data Range (Int)	138 - 65534

Analysis Settings

Detection	Lane detection:<br>Manually created lanes<br><br>Band detection:<br>Automatically detected bands with sensitivity: Low<br>Manually adjusted bands<br><br>Lane Background Subtraction:<br>Lane background subtracted with disk size: 0.1<br><br>Lane width: Variable

Lane Statistics

Lane No.	Adj. Total Band Vol. (Int)	Total Band Vol. (Int)	Adj. Total Lane Vol. (Int)	Total Lane Vol. (Int)	Bkgd. Vol. (Int)	Norm. Factor
1	2 451 215	17 308 016	2 510 942	21 716 763	19 205 821	N/A
2	N/A	N/A	313 880	14 277 830	13 963 950	N/A
3	6 974 484	27 026 560	6 974 484	28 074 552	21 100 068	N/A
4	4 123 840	17 532 840	4 246 920	22 971 640	18 724 720	N/A
5	7 994 385	23 484 015	8 599 995	35 414 325	26 814 330	N/A

Lane And Band Analysis

Lane 1

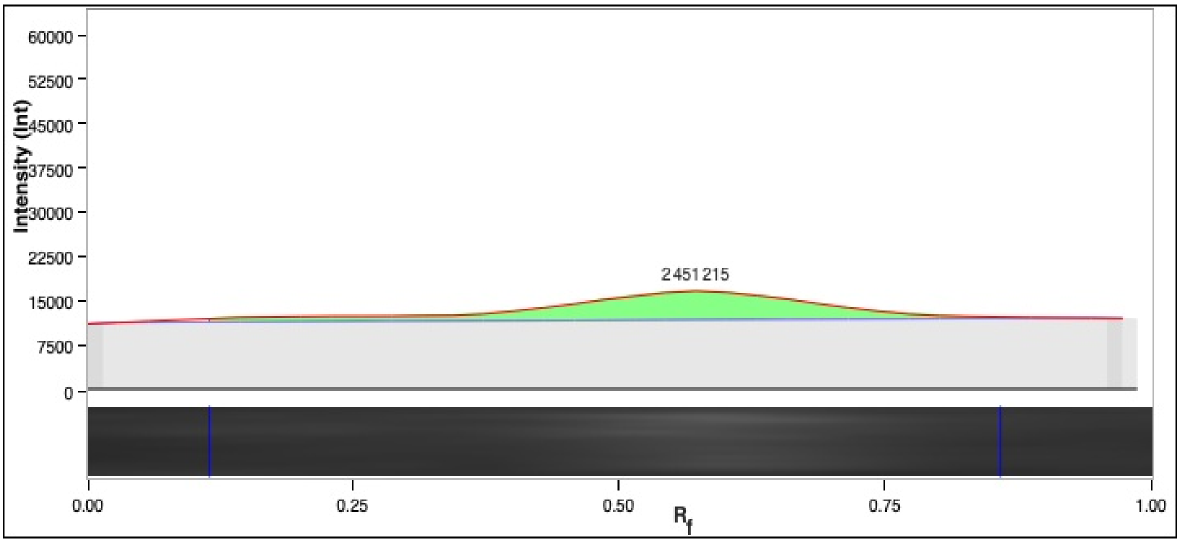

Band No.	Band Label	Mol. Wt. (KDa)	Relative Front	Adj. Volume (Int)	Volume (Int)	Abs. Quant.	Rel. Quant.	Band %	Lane %
1		N/A	0,600	2 451 215	17 308 016	N/A	N/A	100,0	97,6

Band Detection	Automatically detected bands with sensitivity: Low
Lane Background	Lane background subtracted with disk size: 0.1
Lane Width	0.61 mm

Lane 2

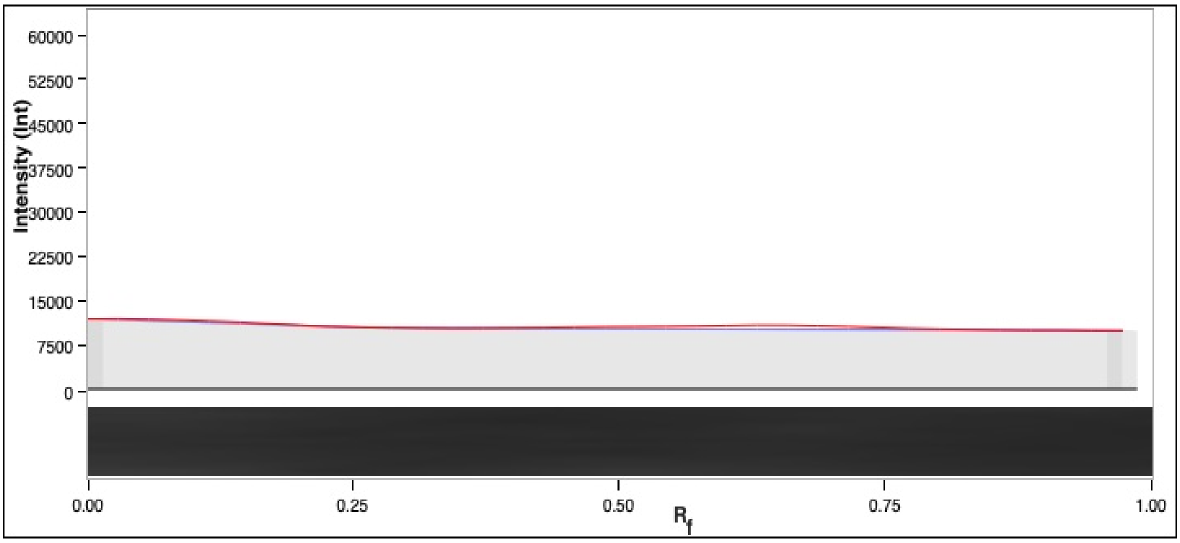

Band No.	Band Label	Mol. Wt. (KDa)	Relative Front	Adj. Volume (Int)	Volume (Int)	Abs. Quant.	Rel. Quant.	Band %	Lane %

Band Detection	Automatically detected bands with sensitivity: Low
Lane Background	Lane background subtracted with disk size: 0.1
Lane Width	0.49 mm

Lane 3

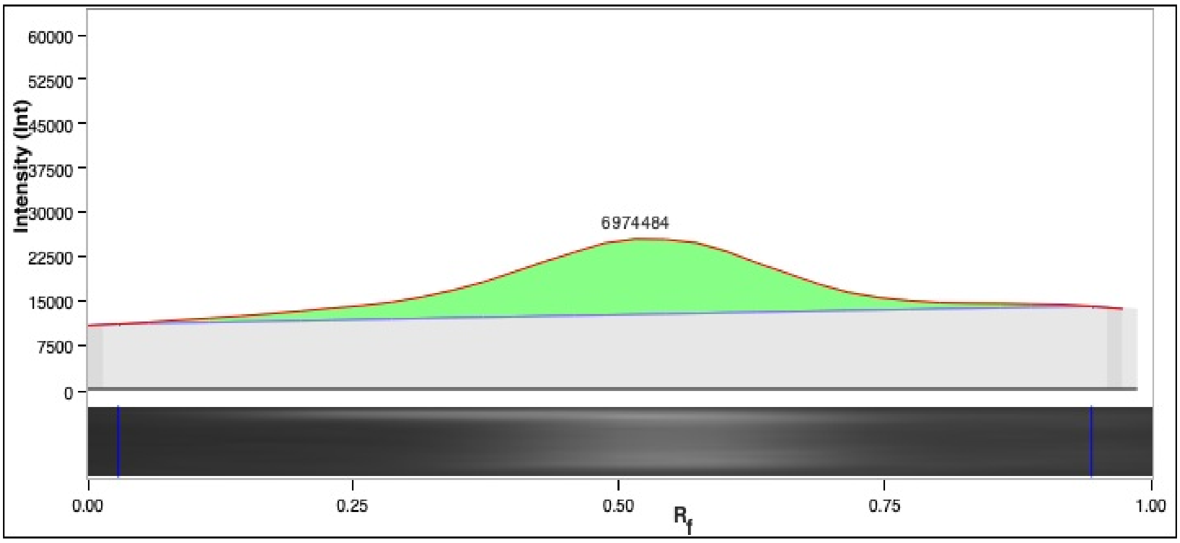

Band No.	Band Label	Mol. Wt. (KDa)	Relative Front	Adj. Volume (Int)	Volume (Int)	Abs. Quant.	Rel. Quant.	Band %	Lane %
1		N/A	0,543	6 974 484	27 026 560	N/A	N/A	100,0	100,0

Band Detection	Automatically detected bands with sensitivity: Low
Lane Background	Lane background subtracted with disk size: 0.1
Lane Width	0.62 mm

Lane 4

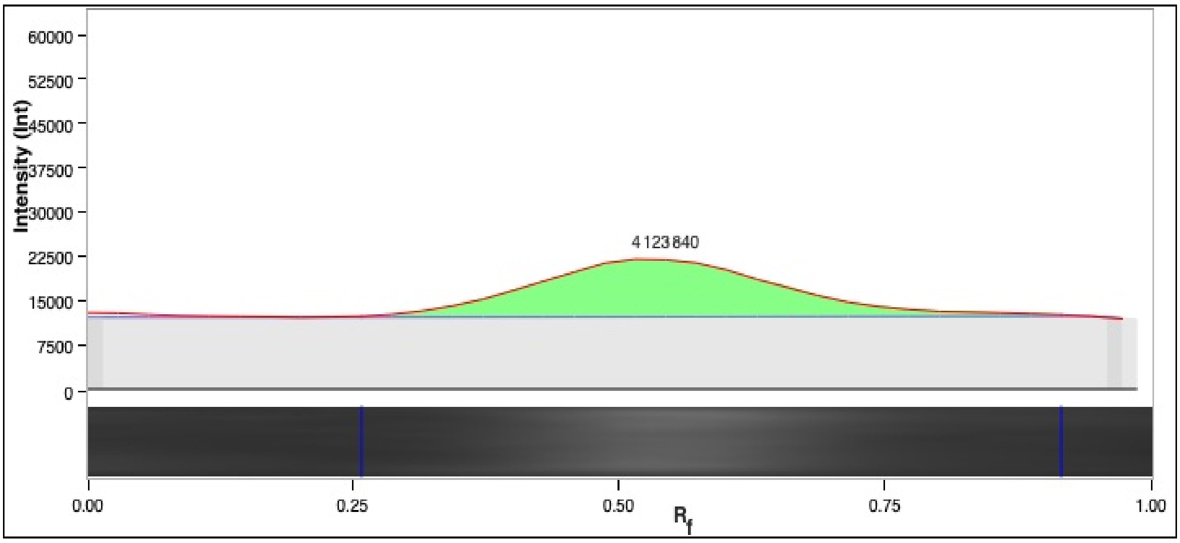

Band No.	Band Label	Mol. Wt. (KDa)	Relative Front	Adj. Volume (Int)	Volume (Int)	Abs. Quant.	Rel. Quant.	Band %	Lane %
1		N/A	0,571	4 123 840	17 532 840	N/A	N/A	100,0	97,1

Band Detection	Automatically detected bands with sensitivity: Low
Lane Background	Lane background subtracted with disk size: 0.1
Lane Width	0.56 mm

Lane 5

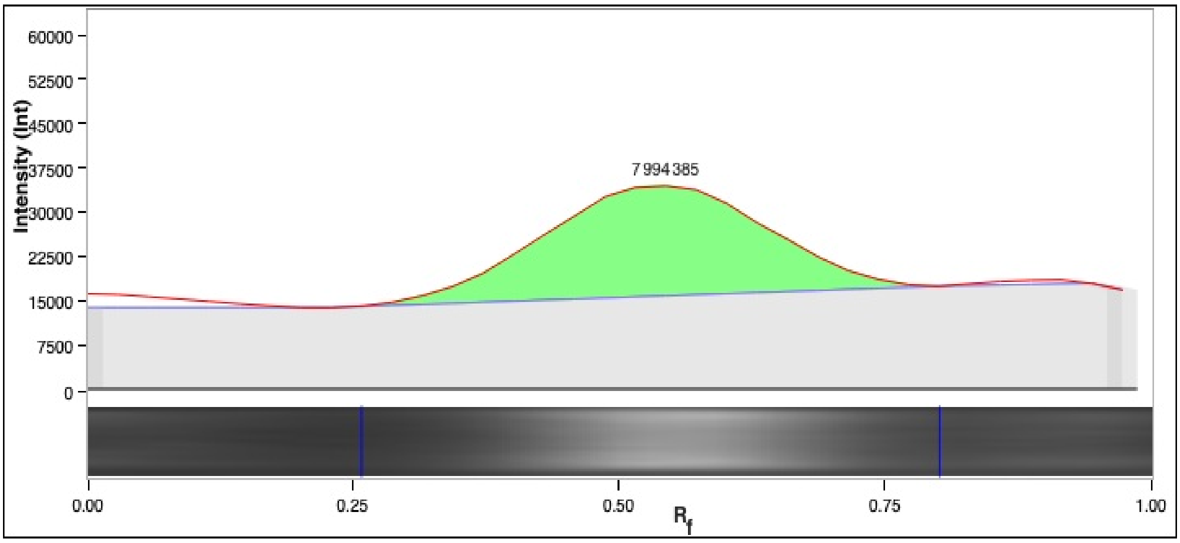

Band No.	Band Label	Mol. Wt. (KDa)	Relative Front	Adj. Volume (Int)	Volume (Int)	Abs. Quant.	Rel. Quant.	Band %	Lane %
1		N/A	0,571	7 994 385	23 484 015	N/A	N/A	100,0	93,0

Band Detection	Automatically detected bands with sensitivity: Low
Lane Background	Lane background subtracted with disk size: 0.1
Lane Width	0.63 mm
