# Supplementary material for: In silico modeling guides identification of novel JAK1 variants associated with immune dysregulation
Source: EMBO Mol Med. 2025 Oct 24;17(12):3275–99. doi: 10.1038/s44321-025-00317-0 (PMC12686074; doi:10.1038/s44321-025-00317-0)
Supplement: Supplementary file 8 — Source data Fig. 3 [file 44321_2025_317_MOESM8_ESM.zip › Figure 3/Replicates Fig.3A/n = 1/GAPDH quantif.pdf]

Image Report: GAPDH quantif

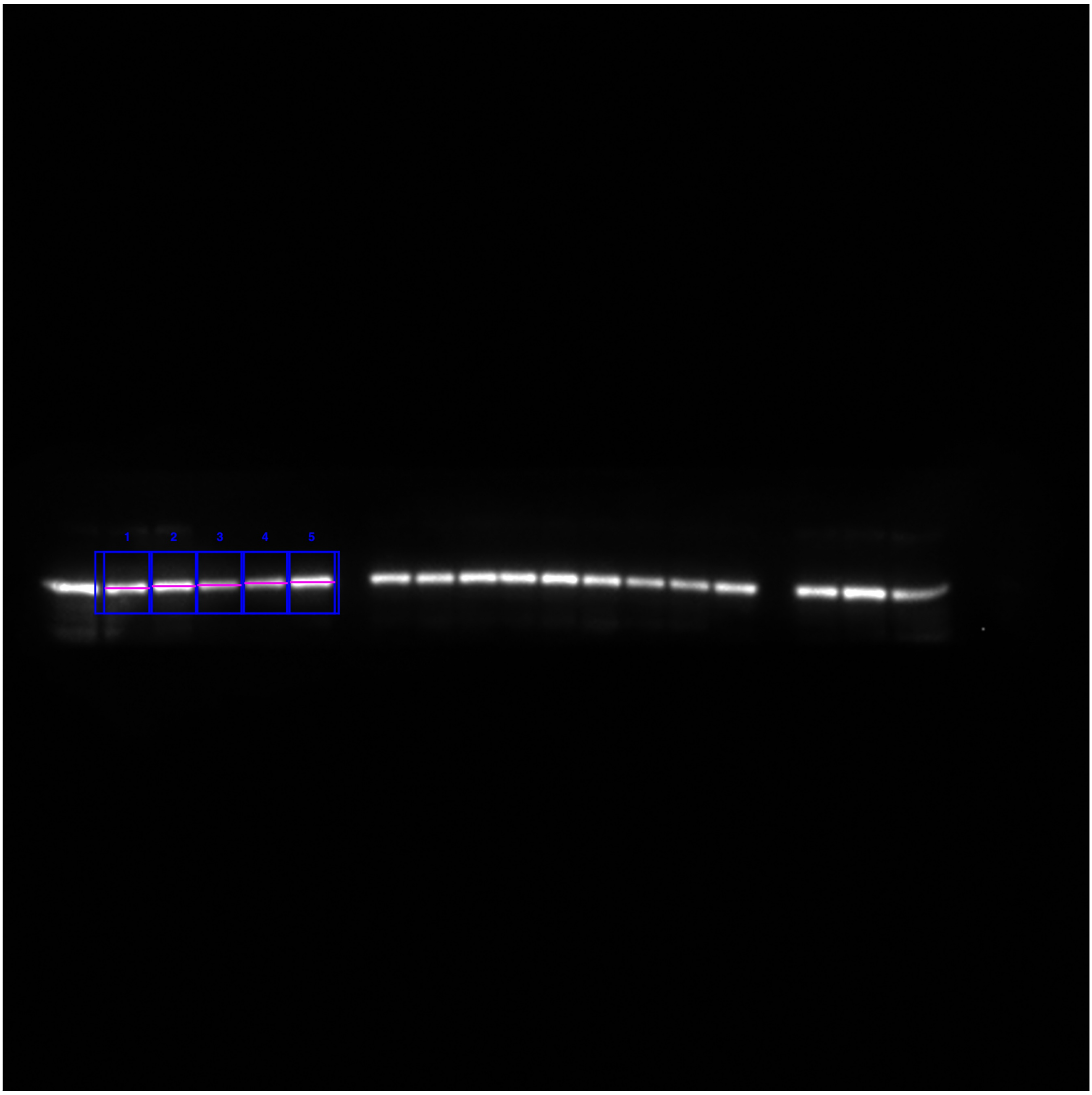

/Volumes/FRL-lab/FRL's Team/Marie Jeanpierre/JAK1/Papier JAK1/Nouvelle submission EMBO/ Source data WB new depot/Quantification Fig.3A/n = 1/GAPDH quantif.scn

Acquisition Information

Image Information

Acquisition Date	unknown
User Name	Marie Jeanpierre

Image Area (mm)	X: 15.2 Y: 15.2
Pixel Size (µm)	X: 14.1 Y: 14.1
Data Range (Int)	130 - 40866

Analysis Settings

Detection	Lane detection:<br>Manually created lanes<br><br>Band detection:<br>Automatically detected bands with sensitivity: Low<br><br>Lane Background Subtraction:<br>Lane background subtracted with disk size: 0.1<br><br>Lane width: Variable

Lane Statistics

Lane No.	Adj. Total Band Vol. (Int)	Total Band Vol. (Int)	Adj. Total Lane Vol. (Int)	Total Lane Vol. (Int)	Bkgd. Vol. (Int)	Norm. Factor
1	11 561 175	13 929 345	12 556 260	17 787 510	5 231 250	N/A
2	12 040 732	13 993 716	13 115 080	17 793 072	4 677 992	N/A
3	7 077 532	8 268 788	7 877 100	11 034 804	3 157 704	N/A
4	7 739 011	8 660 587	8 497 703	10 837 677	2 339 974	N/A
5	13 630 275	14 981 040	14 403 690	17 352 720	2 949 030	N/A

Lane And Band Analysis

Lane 1

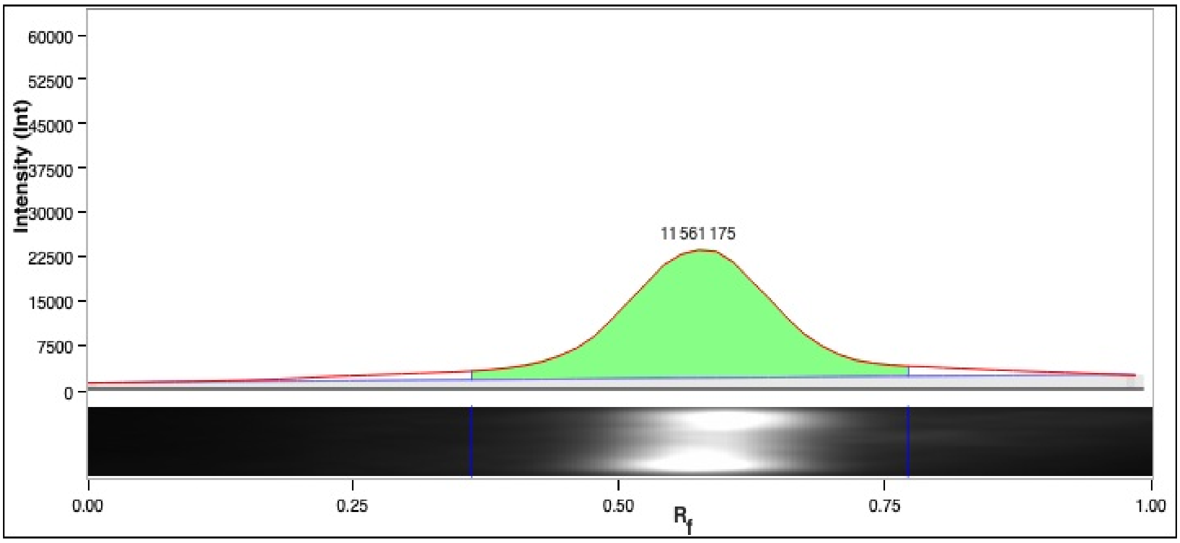

Band No.	Band Label	Mol. Wt. (KDa)	Relative Front	Adj. Volume (Int)	Volume (Int)	Abs. Quant.	Rel. Quant.	Band %	Lane %
1		N/A	0,590	11 561 175	13 929 345	N/A	N/A	100,0	92,1

Band Detection	Automatically detected bands with sensitivity: Low

Lane Background	Lane background subtracted with disk size: 0.1
Lane Width	0.63 mm

Lane 2

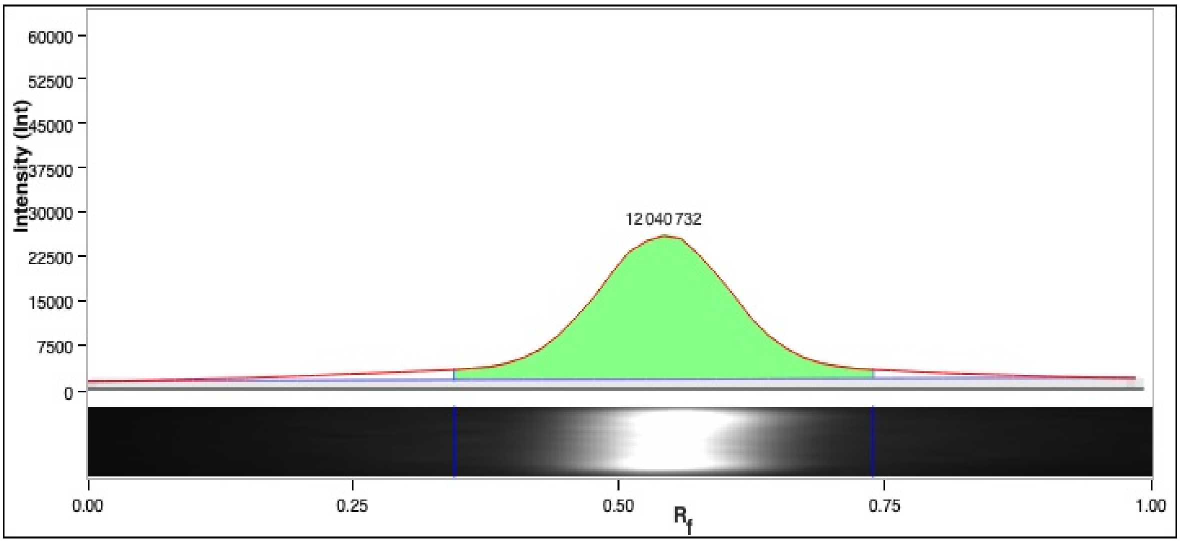

Band No.	Band Label	Mol. Wt. (KDa)	Relative Front	Adj. Volume (Int)	Volume (Int)	Abs. Quant.	Rel. Quant.	Band %	Lane %
1		N/A	0,557	12 040 732	13 993 716	N/A	N/A	100,0	91,8

Band Detection	Automatically detected bands with sensitivity: Low
Lane Background	Lane background subtracted with disk size: 0.1
Lane Width	0.62 mm

Lane 3

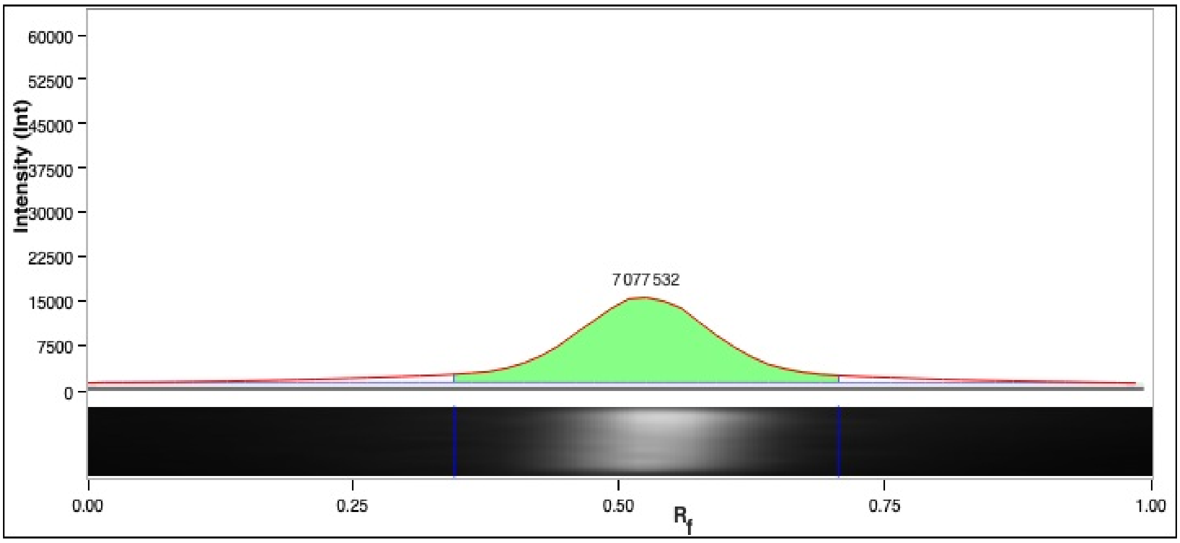

Band No.	Band Label	Mol. Wt. (KDa)	Relative Front	Adj. Volume	Volume (Int)	Abs. Quant.	Rel. Quant.	Band %	Lane %

				(Int)					
1		N/A	0,541	7 077 532	8 268 788	N/A	N/A	100,0	89,8

Band Detection	Automatically detected bands with sensitivity: Low
Lane Background	Lane background subtracted with disk size: 0.1
Lane Width	0.62 mm

Lane 4

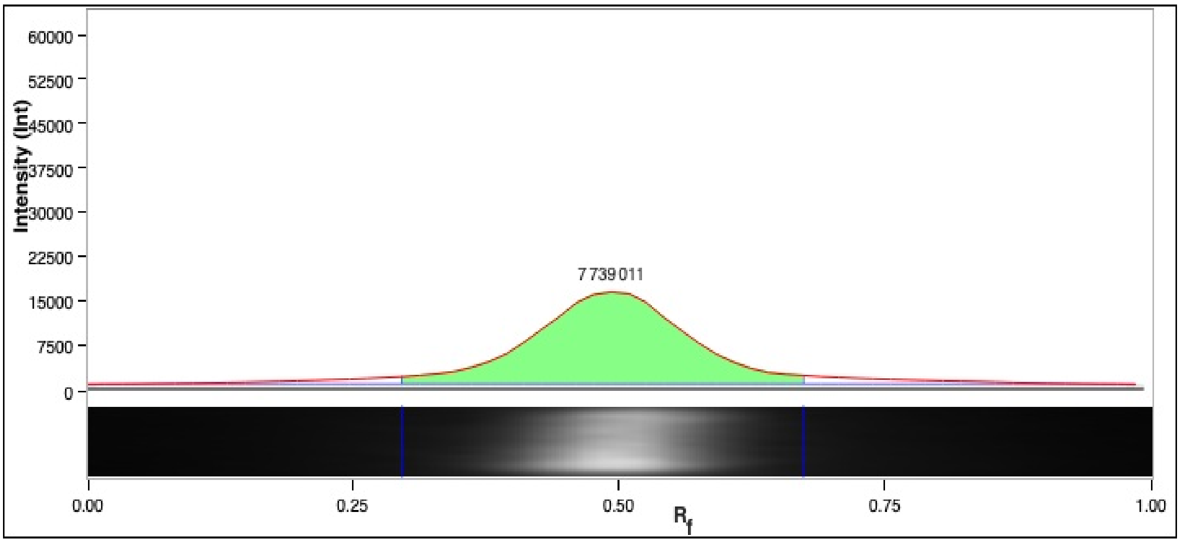

Band No.	Band Label	Mol. Wt. (KDa)	Relative Front	Adj. Volume (Int)	Volume (Int)	Abs. Quant.	Rel. Quant.	Band %	Lane %
1		N/A	0,508	7 739 011	8 660 587	N/A	N/A	100,0	91,1

Band Detection	Automatically detected bands with sensitivity: Low
Lane Background	Lane background subtracted with disk size: 0.1
Lane Width	0.61 mm

Lane 5

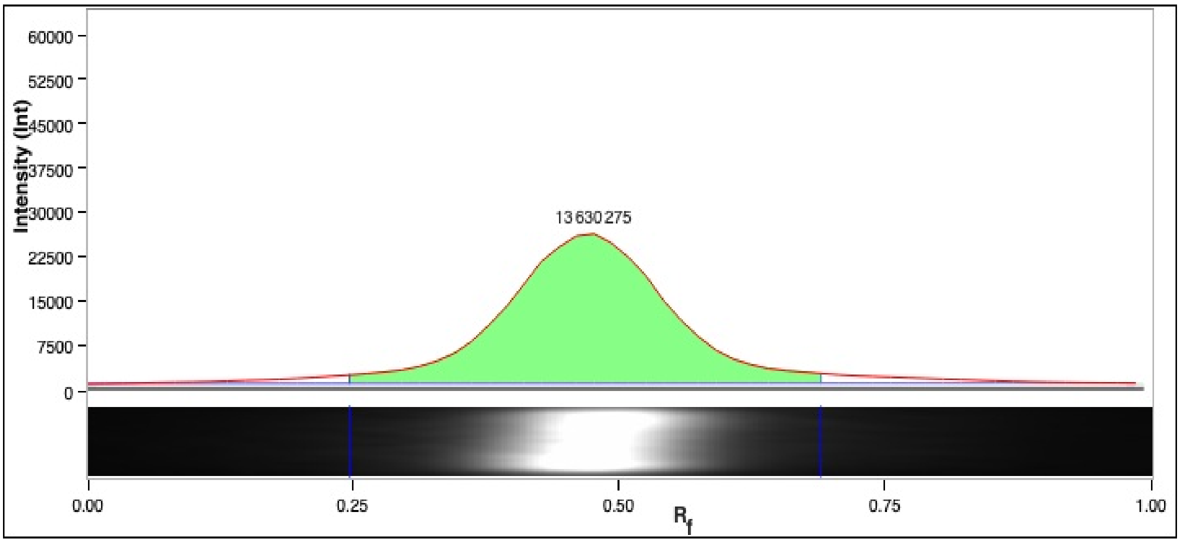

Band No.	Band Label	Mol. Wt. (KDa)	Relative Front	Adj. Volume (Int)	Volume (Int)	Abs. Quant.	Rel. Quant.	Band %	Lane %
1		N/A	0,492	13 630 275	14 981 040	N/A	N/A	100,0	94,6

Band Detection	Automatically detected bands with sensitivity: Low
Lane Background	Lane background subtracted with disk size: 0.1
Lane Width	0.63 mm
