# Supplementary material for: In silico modeling guides identification of novel JAK1 variants associated with immune dysregulation
Source: EMBO Mol Med. 2025 Oct 24;17(12):3275–99. doi: 10.1038/s44321-025-00317-0 (PMC12686074; doi:10.1038/s44321-025-00317-0)
Supplement: Supplementary file 8 — Source data Fig. 3 [file 44321_2025_317_MOESM8_ESM.zip › Figure 3/Replicates Fig.3A/n = 2/GAPDH quantif.pdf]

Image Report: GAPDH quantif

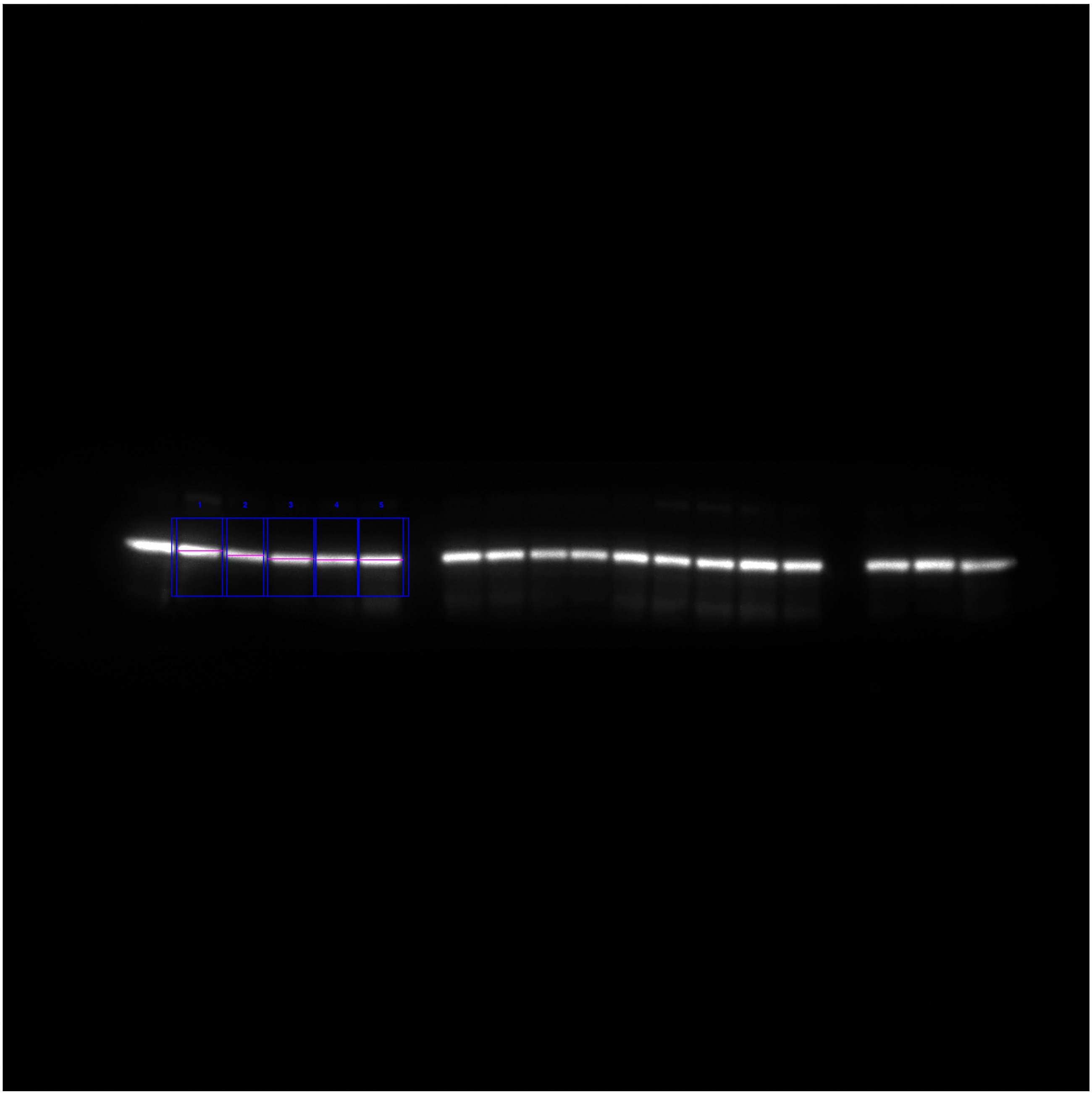

/Volumes/FRL-lab/FRL's Team/Marie Jeanpierre/JAK1/Papier JAK1/Nouvelle submission EMBO/ Source data WB new depot/Quantification Fig.3A/n = 2/GAPDH quantif.scn

Acquisition Information

Image Information

Acquisition Date	unknown
User Name	Marie Jeanpierre

Image Area (mm)	X: 30.5 Y: 30.5
Pixel Size (µm)	X: 14.1 Y: 14.1
Data Range (Int)	130 - 19681

Analysis Settings

Detection	Lane detection:<br>Manually created lanes<br><br>Band detection:<br><br>Manually adjusted bands<br><br>Lane Background Subtraction:<br>Lane background subtracted with disk size: 0.1<br><br>Lane width: Variable

Lane Statistics

Lane No.	Adj. Total Band Vol. (Int)	Total Band Vol. (Int)	Adj. Total Lane Vol. (Int)	Total Lane Vol. (Int)	Bkgd. Vol. (Int)	Norm. Factor
1	24 060 309	26 690 391	28 405 195	38 013 976	9 608 781	N/A
2	13 569 094	15 150 128	15 261 307	21 305 561	6 044 254	N/A
3	16 234 964	18 149 760	18 383 624	25 875 368	7 491 744	N/A
4	16 482 306	18 417 202	18 667 447	26 123 171	7 455 724	N/A
5	18 181 504	21 645 272	19 470 176	31 565 248	12 095 072	N/A

Lane And Band Analysis

Lane 1

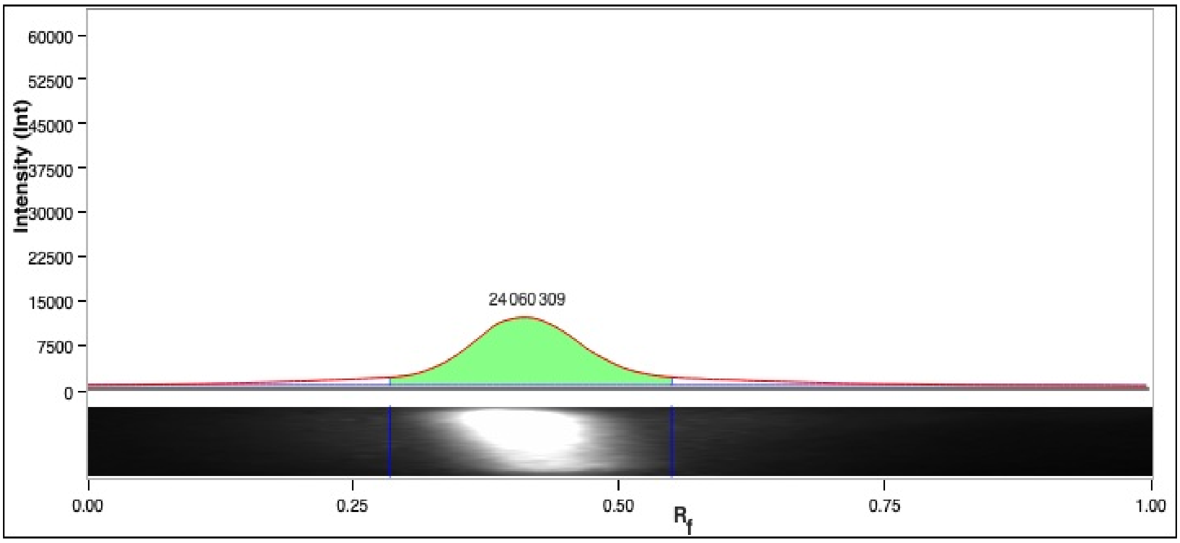

Band No.	Band Label	Mol. Wt. (KDa)	Relative Front	Adj. Volume (Int)	Volume (Int)	Abs. Quant.	Rel. Quant.	Band %	Lane %
1		N/A	0,419	24 060 309	26 690 391	N/A	N/A	100,0	84,7

Lane Background	Lane background subtracted with disk size: 0.1
Lane Width	1.28 mm

Lane 2

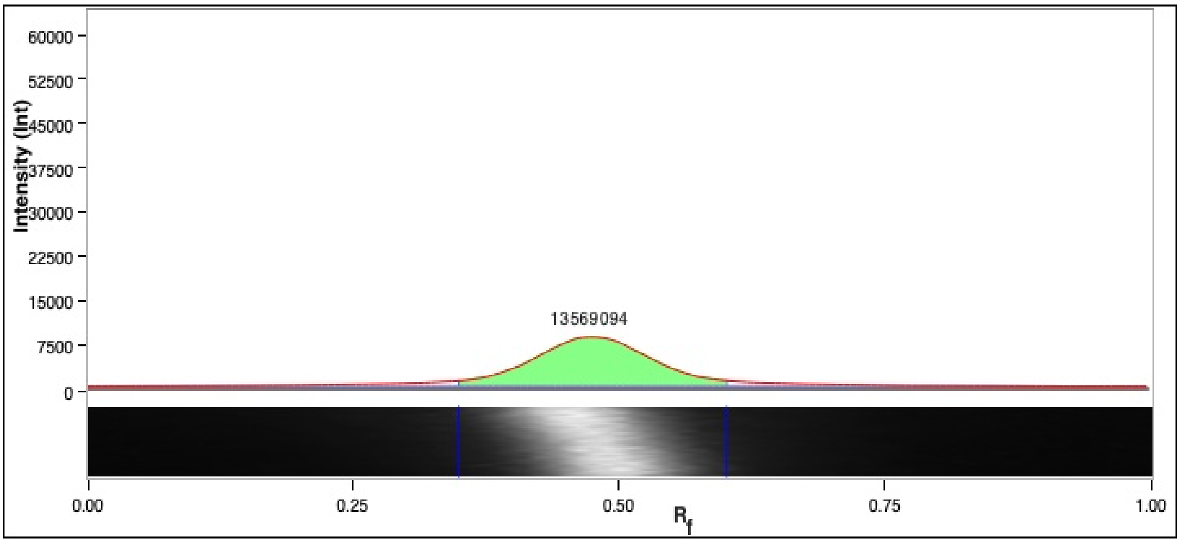

Band No.	Band Label	Mol. Wt. (KDa)	Relative Front	Adj. Volume (Int)	Volume (Int)	Abs. Quant.	Rel. Quant.	Band %	Lane %
1		N/A	0,477	13 569 094	15 150 128	N/A	N/A	100,0	88,9

Lane Background	Lane background subtracted with disk size: 0.1
Lane Width	1.03 mm

Lane 3

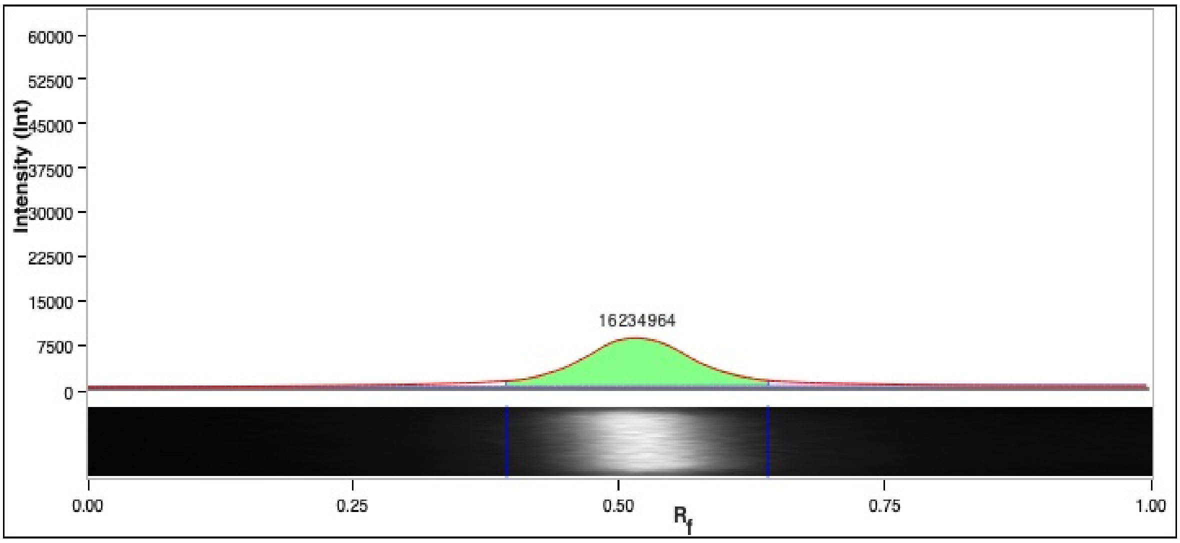

Band No.	Band Label	Mol. Wt. (KDa)	Relative Front	Adj. Volume (Int)	Volume (Int)	Abs. Quant.	Rel. Quant.	Band %	Lane %

1		N/A	0,523	16 234 964	18 149 760	N/A	N/A	100,0	88,3

Lane Background	Lane background subtracted with disk size: 0.1
Lane Width	1.30 mm

Lane 4

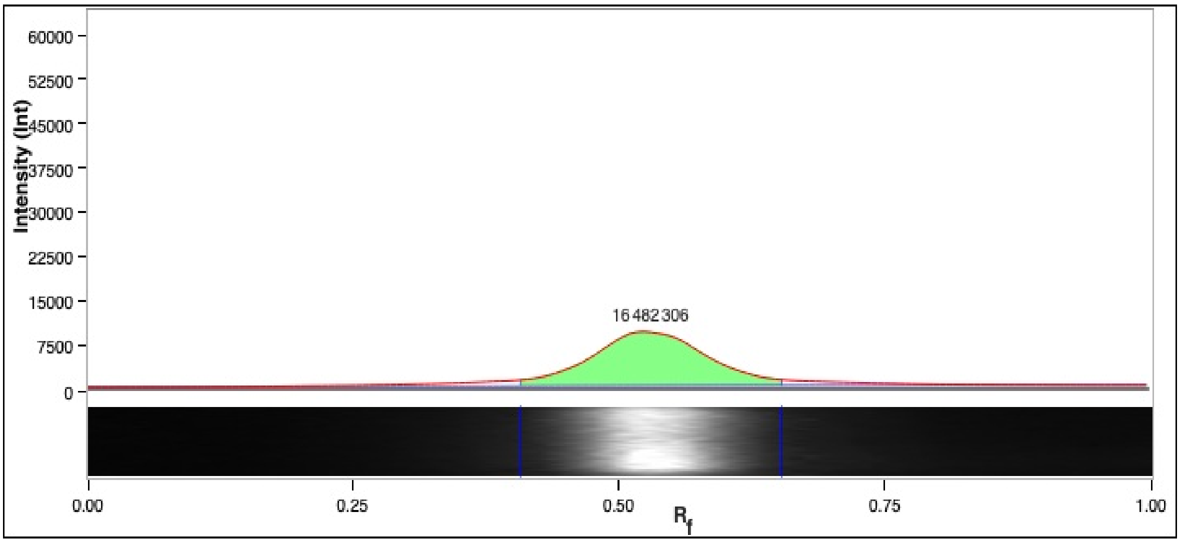

Band No.	Band Label	Mol. Wt. (KDa)	Relative Front	Adj. Volume (Int)	Volume (Int)	Abs. Quant.	Rel. Quant.	Band %	Lane %
1		N/A	0,535	16 482 306	18 417 202	N/A	N/A	100,0	88,3

Lane Background	Lane background subtracted with disk size: 0.1
Lane Width	1.17 mm

Lane 5

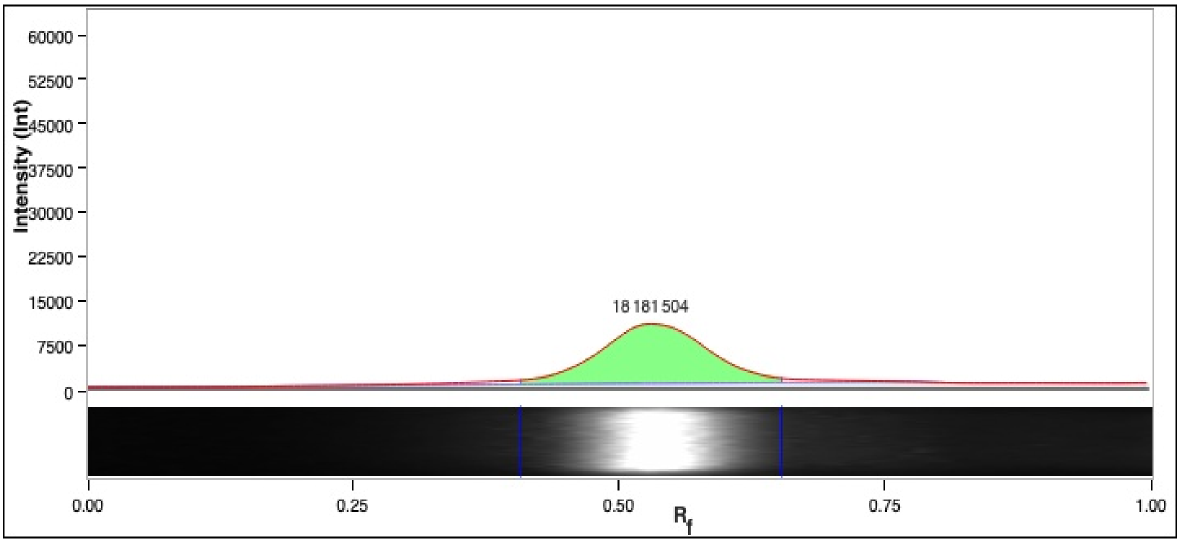

Band No.	Band	Mol. Wt.	Relative	Adj.	Volume	Abs.	Rel.	Band %	Lane %

	Label	(KDa)	Front	Volume (Int)	(Int)	Quant.	Quant.		
1		N/A	0,535	18 181 504	21 645 272	N/A	N/A	100,0	93,4

Lane Background	Lane background subtracted with disk size: 0.1
Lane Width	1.24 mm
