# Supplementary material for: In silico modeling guides identification of novel JAK1 variants associated with immune dysregulation
Source: EMBO Mol Med. 2025 Oct 24;17(12):3275–99. doi: 10.1038/s44321-025-00317-0 (PMC12686074; doi:10.1038/s44321-025-00317-0)
Supplement: Supplementary file 8 — Source data Fig. 3 [file 44321_2025_317_MOESM8_ESM.zip › Figure 3/Replicates Fig.3A/n = 3/GAPDH quantif.pdf]

Image Report: GAPDH quantif

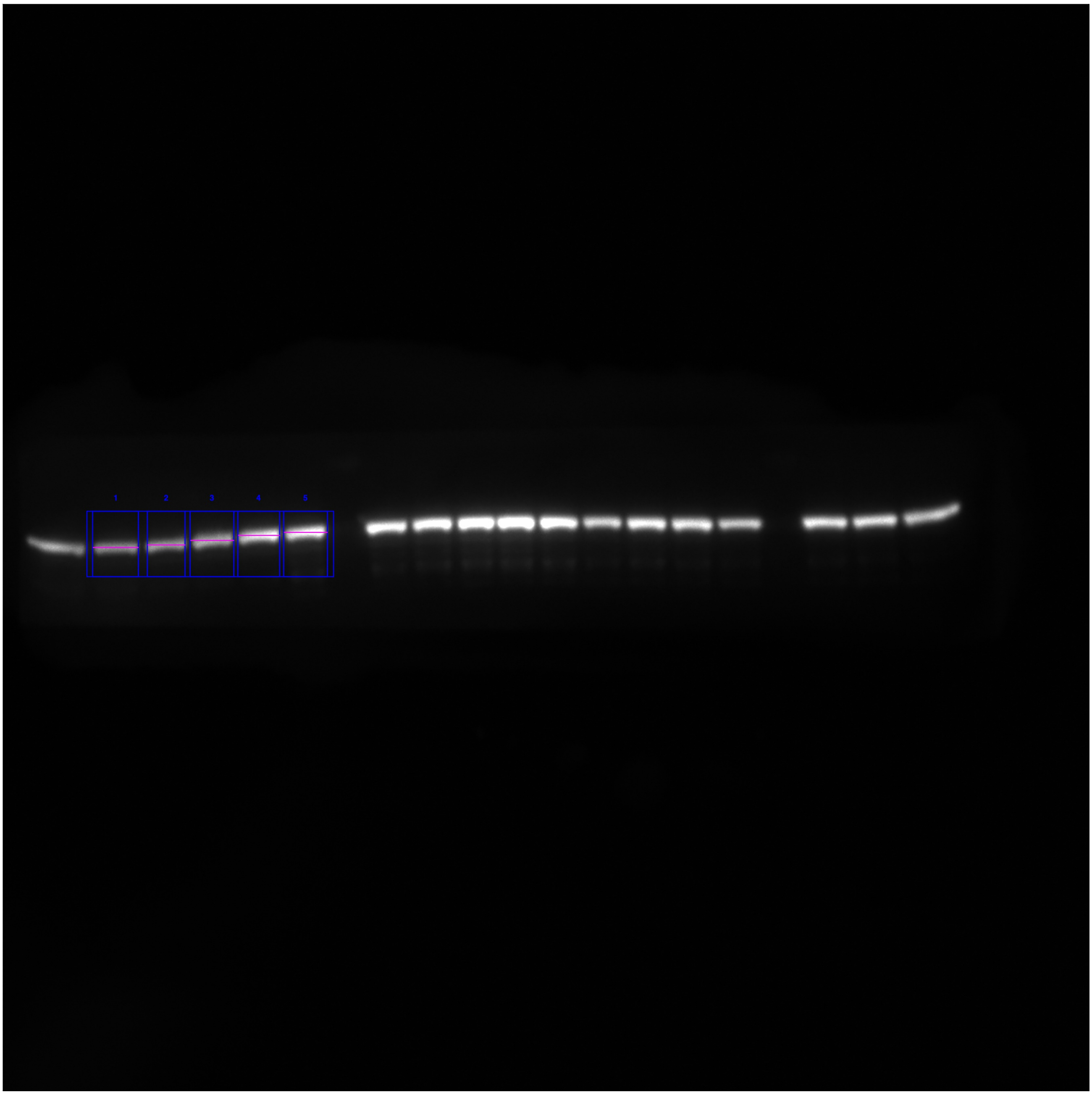

/Volumes/FRL-lab/FRL's Team/Marie Jeanpierre/JAK1/Papier JAK1/Nouvelle submission EMBO/ Source data WB new depot/Quantification Fig.3A/n = 3/GAPDH quantif.scn

Acquisition Information

Image Information

Acquisition Date	unknown
User Name	Marie Jeanpierre

Image Area (mm)	X: 30.5 Y: 30.5
Pixel Size (µm)	X: 14.1 Y: 14.1
Data Range (Int)	142 - 38708

Analysis Settings

Detection	Lane detection:<br>Manually created lanes<br><br>Band detection:<br><br>Manually adjusted bands<br><br>Lane Background Subtraction:<br>Lane background subtracted with disk size: 0.1<br><br>Lane width: Variable

Lane Statistics

Lane No.	Adj. Total Band Vol. (Int)	Total Band Vol. (Int)	Adj. Total Lane Vol. (Int)	Total Lane Vol. (Int)	Bkgd. Vol. (Int)	Norm. Factor
1	22 196 629	29 070 041	24 555 895	46 533 669	21 977 774	N/A
2	20 873 100	27 023 700	22 935 150	42 150 600	19 215 450	N/A
3	30 046 581	37 958 013	32 809 092	54 060 408	21 251 316	N/A
4	40 457 022	48 492 252	43 377 792	64 057 076	20 679 284	N/A
5	41 513 703	50 998 008	44 048 361	70 119 216	26 070 855	N/A

Lane And Band Analysis

Lane 1

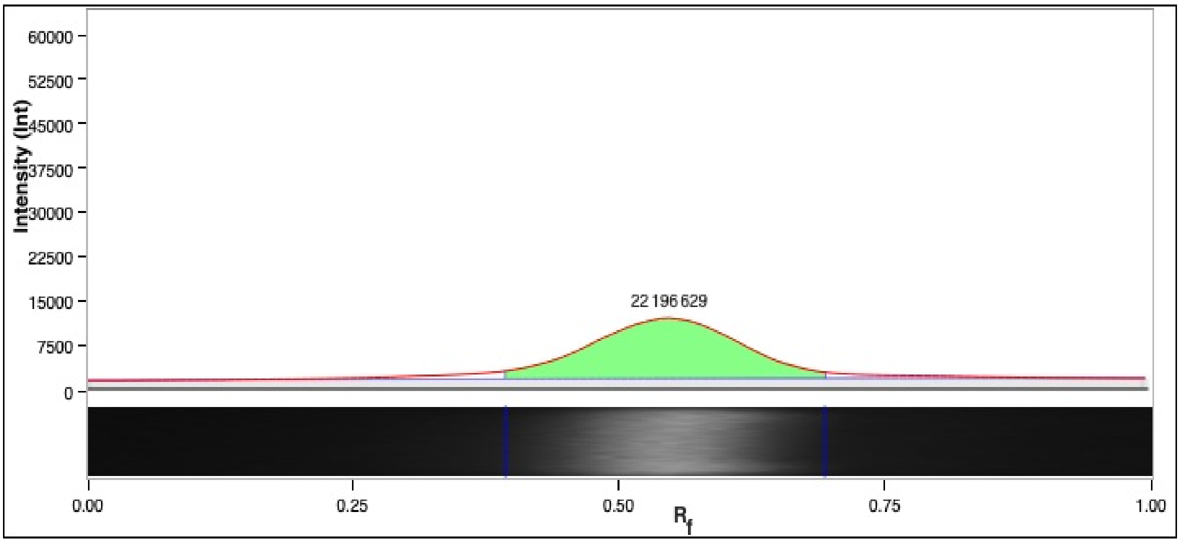

Band No.	Band Label	Mol. Wt. (KDa)	Relative Front	Adj. Volume (Int)	Volume (Int)	Abs. Quant.	Rel. Quant.	Band %	Lane %
1		N/A	0,554	22 196 629	29 070 041	N/A	N/A	100,0	90,4

Lane Background	Lane background subtracted with disk size: 0.1
Lane Width	1.28 mm

Lane 2

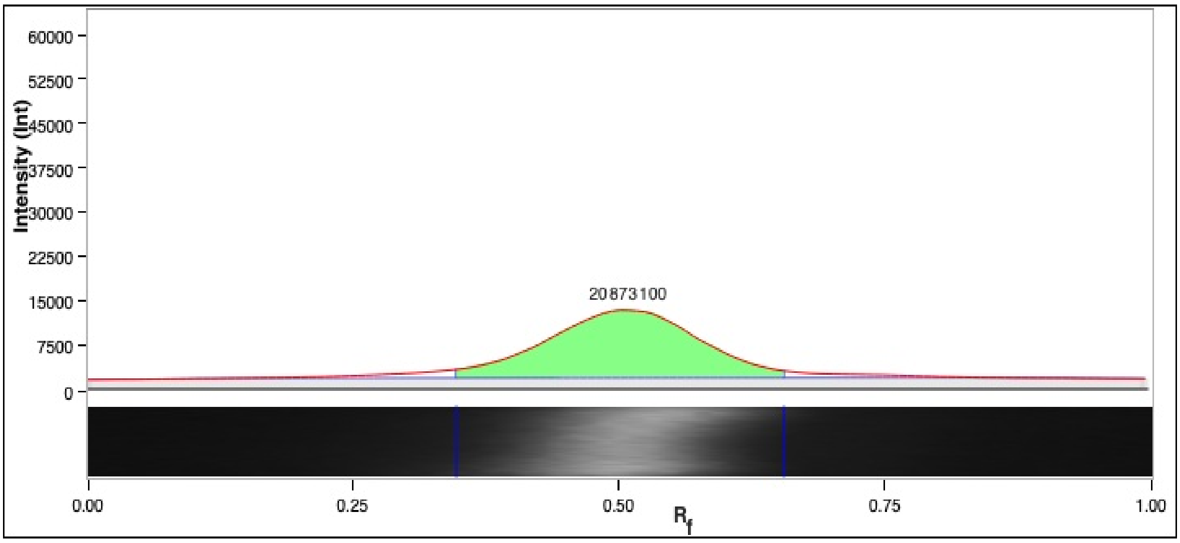

Band No.	Band Label	Mol. Wt. (KDa)	Relative Front	Adj. Volume (Int)	Volume (Int)	Abs. Quant.	Rel. Quant.	Band %	Lane %
1		N/A	0,515	20 873 100	27 023 700	N/A	N/A	100,0	91,0

Lane Background	Lane background subtracted with disk size: 0.1
Lane Width	1.06 mm

Lane 3

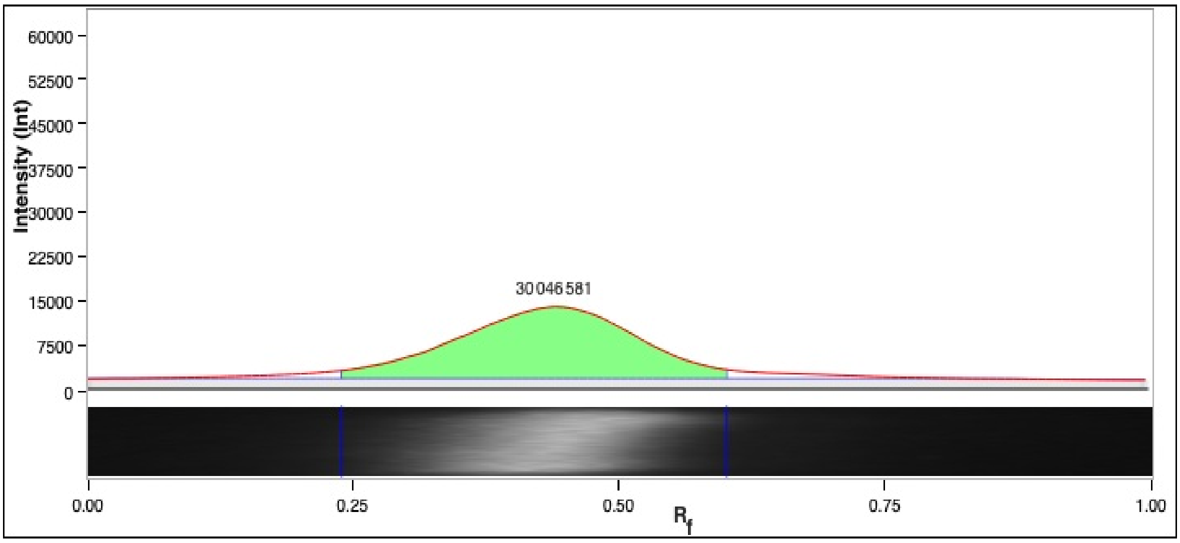

Band No.	Band Label	Mol. Wt. (KDa)	Relative Front	Adj. Volume (Int)	Volume (Int)	Abs. Quant.	Rel. Quant.	Band %	Lane %

1		N/A	0,446	30 046 581	37 958 013	N/A	N/A	100,0	91,6

Lane Background	Lane background subtracted with disk size: 0.1
Lane Width	1.23 mm

Lane 4

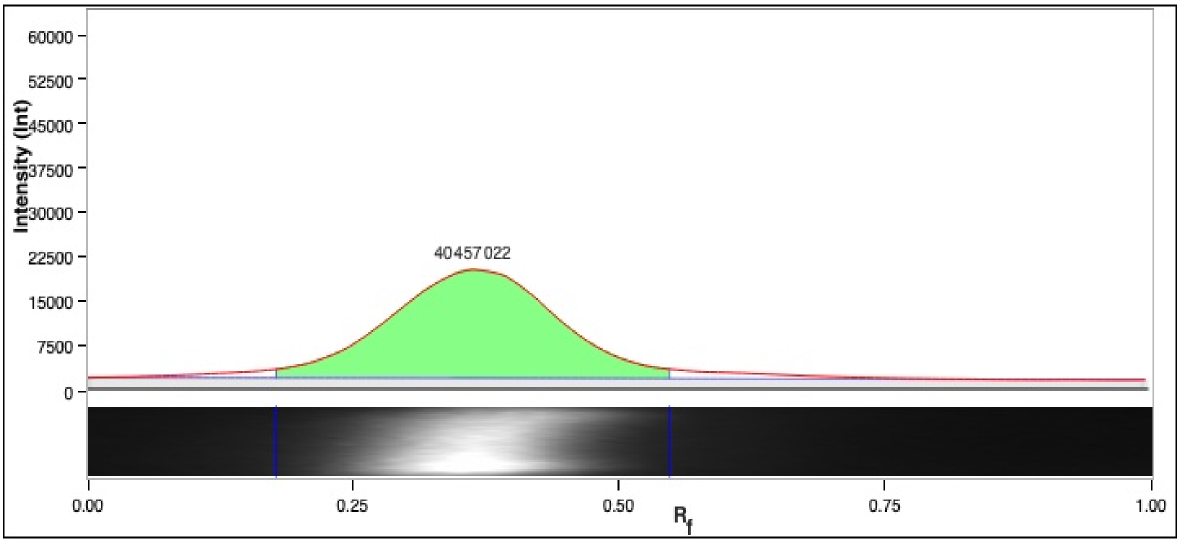

Band No.	Band Label	Mol. Wt. (KDa)	Relative Front	Adj. Volume (Int)	Volume (Int)	Abs. Quant.	Rel. Quant.	Band %	Lane %
1		N/A	0,369	40 457 022	48 492 252	N/A	N/A	100,0	93,3

Lane Background	Lane background subtracted with disk size: 0.1
Lane Width	1.17 mm

Lane 5

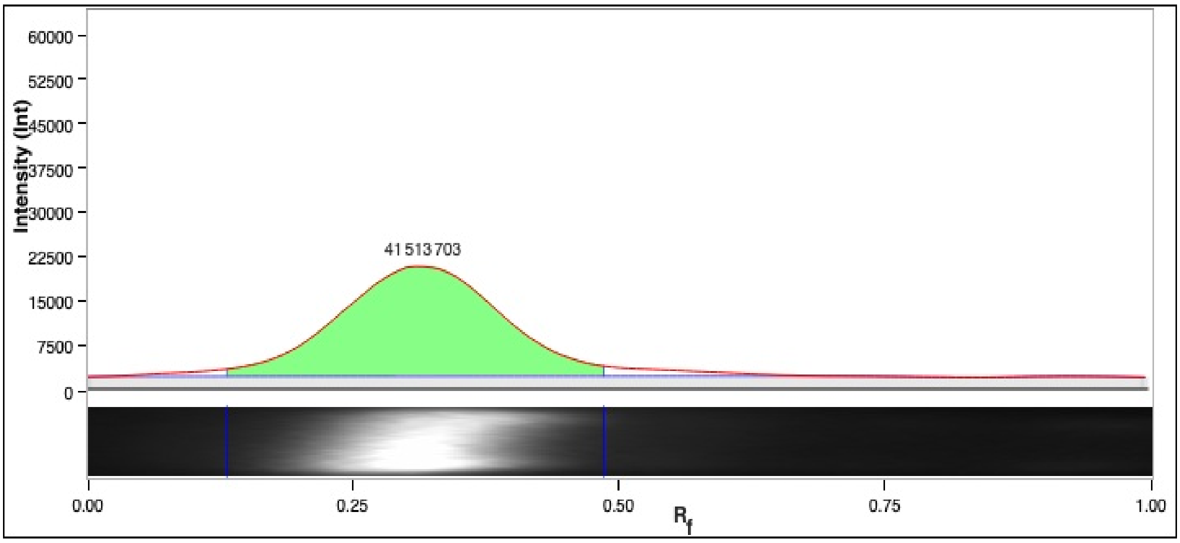

Band No.	Band	Mol. Wt.	Relative	Adj.	Volume	Abs.	Rel.	Band %	Lane %

	Label	(KDa)	Front	Volume (Int)	(Int)	Quant.	Quant.		
1		N/A	0,323	41 513 703	50 998 008	N/A	N/A	100,0	94,2

Lane Background	Lane background subtracted with disk size: 0.1
Lane Width	1.23 mm
