# Supplementary material for: In silico modeling guides identification of novel JAK1 variants associated with immune dysregulation
Source: EMBO Mol Med. 2025 Oct 24;17(12):3275–99. doi: 10.1038/s44321-025-00317-0 (PMC12686074; doi:10.1038/s44321-025-00317-0)
Supplement: Supplementary file 8 — Source data Fig. 3 [file 44321_2025_317_MOESM8_ESM.zip › Figure 3/Replicates Fig.3A/n = 3/Quantif pSTAT1.pdf]

Image Report: Quantif pSTAT1

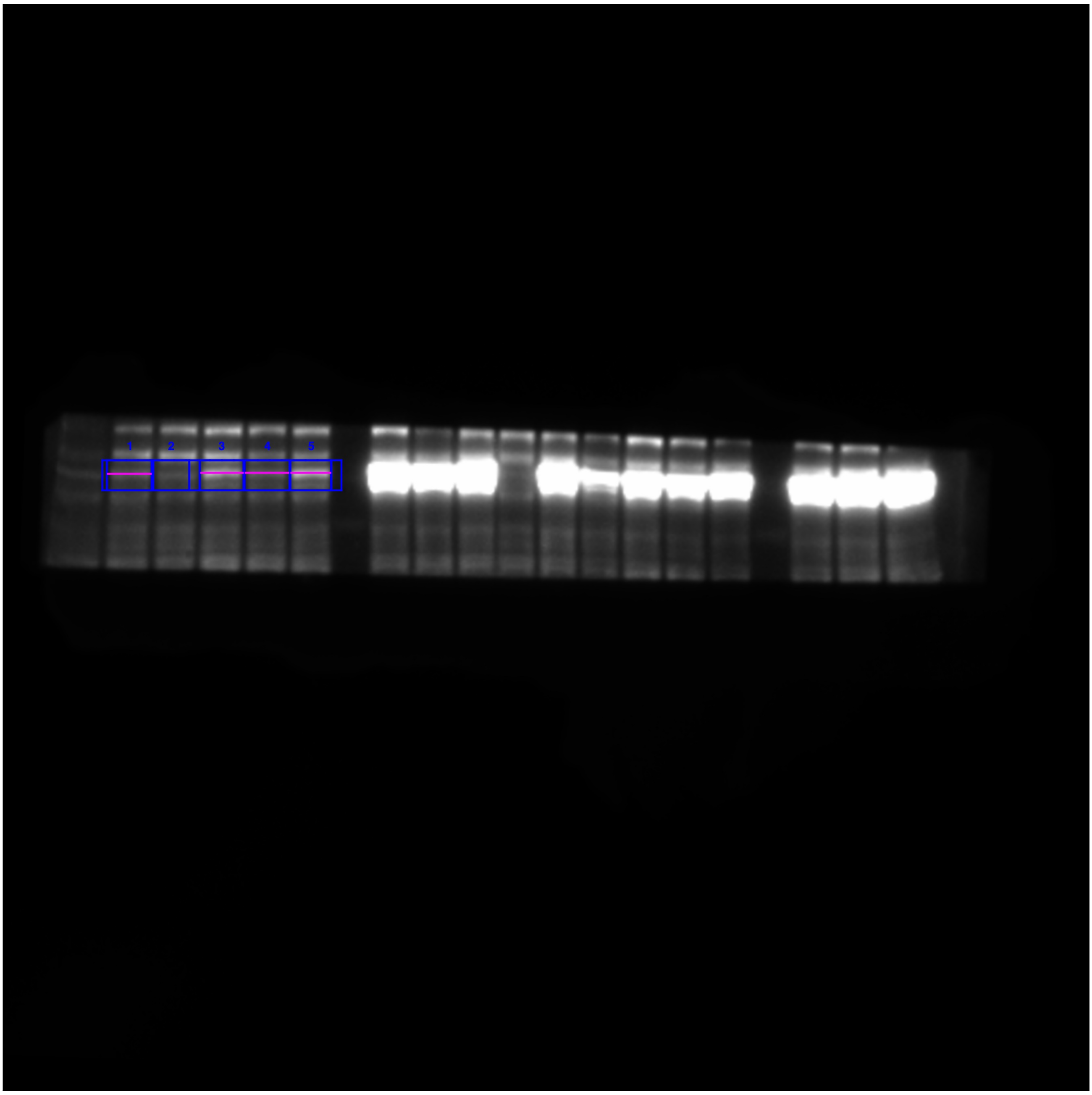

/Volumes/FRL-lab/FRL's Team/Marie Jeanpierre/JAK1/Papier JAK1/Nouvelle submission EMBO/ Source data WB new depot/Quantification Fig.3A/n = 3/Quantif pSTAT1.scn

Acquisition Information

Image Information

Acquisition Date	unknown
User Name	Marie Jeanpierre

Image Area (mm)	X: 15.7 Y: 15.7
Pixel Size (µm)	X: 14.1 Y: 14.1
Data Range (Int)	0 - 65534

Analysis Settings

Detection	Lane detection:<br>Manually created lanes<br><br>Band detection:<br><br>Manually adjusted bands<br><br>Lane Background Subtraction:<br>Lane background subtracted with disk size: 0.1<br><br>Lane width: Variable

Lane Statistics

Lane No.	Adj. Total Band Vol. (Int)	Total Band Vol. (Int)	Adj. Total Lane Vol. (Int)	Total Lane Vol. (Int)	Bkgd. Vol. (Int)	Norm. Factor
1	2 775 364	13 624 648	3 047 592	22 701 000	19 653 408	N/A
2	N/A	N/A	1 674 879	18 447 793	16 772 914	N/A
3	6 075 765	24 455 115	6 534 090	35 839 485	29 305 395	N/A
4	2 421 900	13 562 732	2 672 554	24 205 062	21 532 508	N/A
5	7 733 124	23 444 232	8 235 990	34 778 940	26 542 950	N/A

Lane And Band Analysis

Lane 1

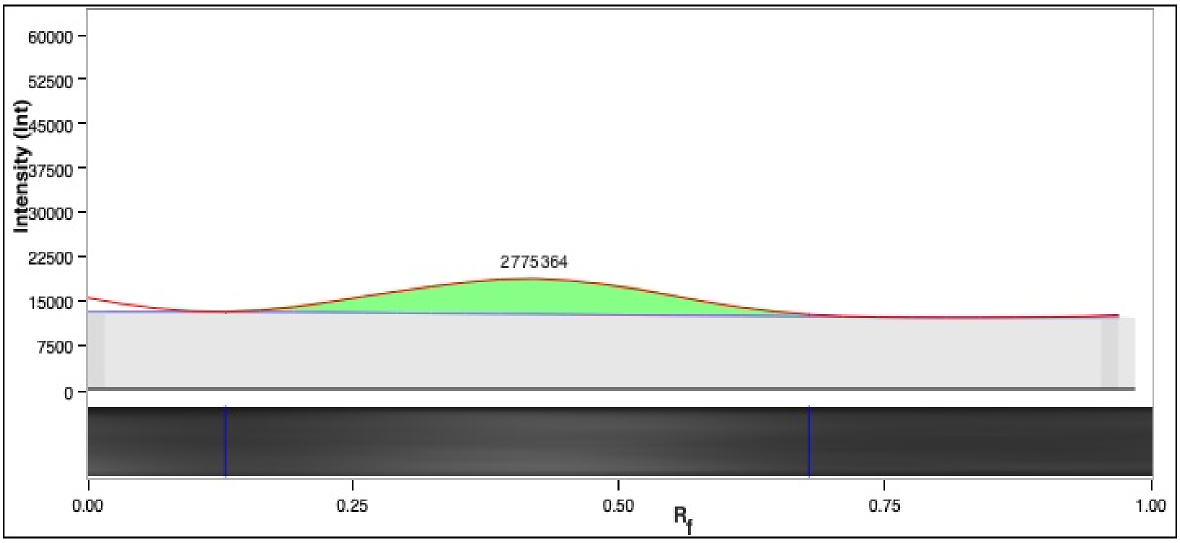

Band No.	Band Label	Mol. Wt. (KDa)	Relative Front	Adj. Volume (Int)	Volume (Int)	Abs. Quant.	Rel. Quant.	Band %	Lane %
1		N/A	0,452	2 775 364	13 624 648	N/A	N/A	100,0	91,1

Lane Background	Lane background subtracted with disk size: 0.1
Lane Width	0.65 mm

Lane 2

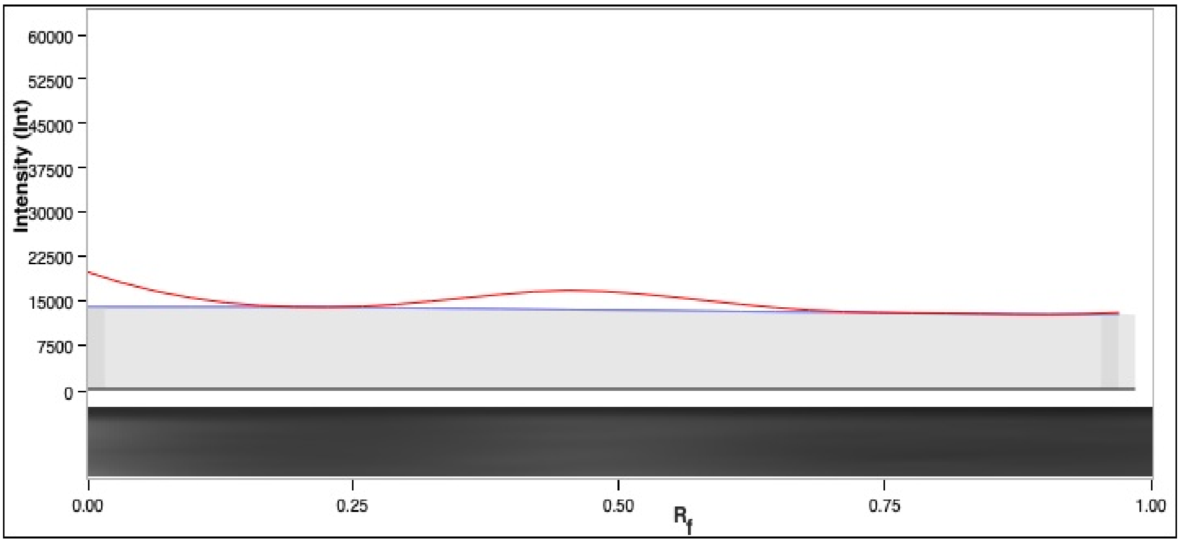

Band No.	Band Label	Mol. Wt. (KDa)	Relative Front	Adj. Volume (Int)	Volume (Int)	Abs. Quant.	Rel. Quant.	Band %	Lane %

Lane Background	Lane background subtracted with disk size: 0.1
Lane Width	0.52 mm

Lane 3

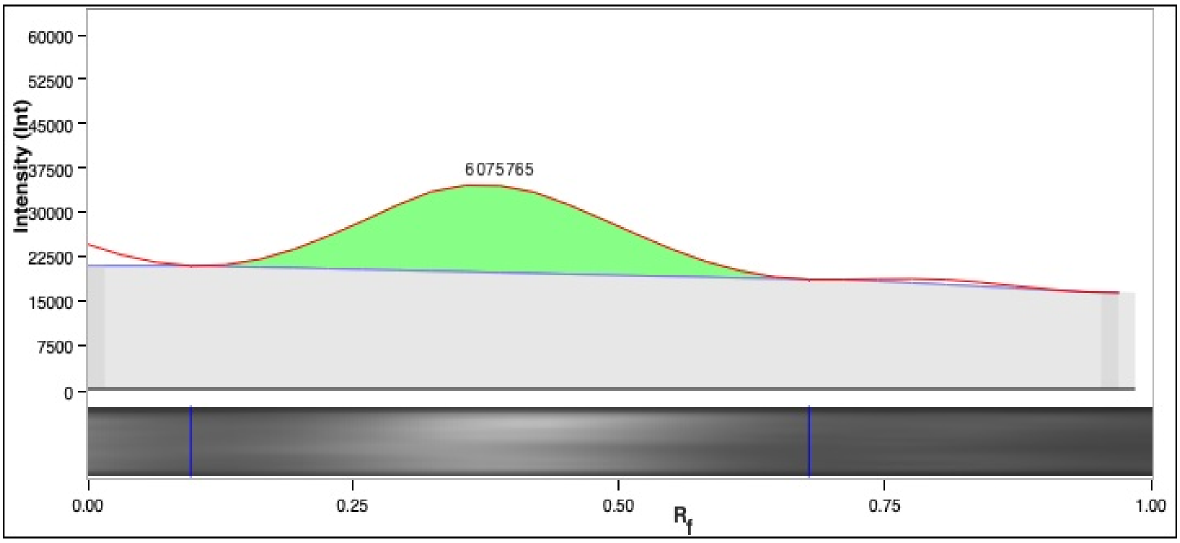

Band No.	Band Label	Mol. Wt. (KDa)	Relative Front	Adj. Volume (Int)	Volume (Int)	Abs. Quant.	Rel. Quant.	Band %	Lane %

1		N/A	0,419	6 075 765	24 455 115	N/A	N/A	100,0	93,0

Lane Background	Lane background subtracted with disk size: 0.1
Lane Width	0.64 mm

Lane 4

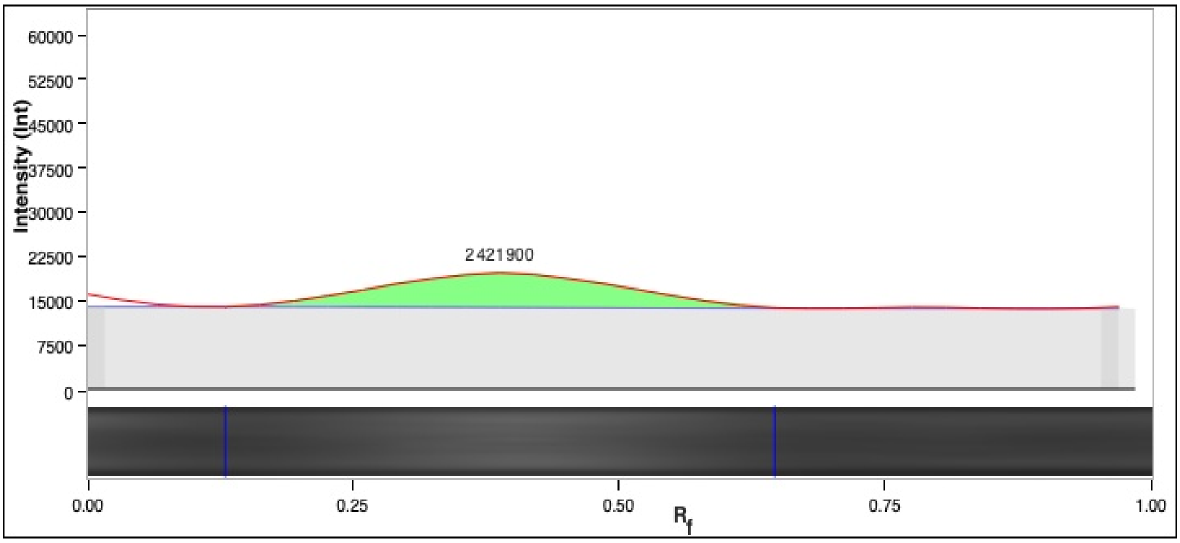

Band No.	Band Label	Mol. Wt. (KDa)	Relative Front	Adj. Volume (Int)	Volume (Int)	Abs. Quant.	Rel. Quant.	Band %	Lane %
1		N/A	0,419	2 421 900	13 562 732	N/A	N/A	100,0	90,6

Lane Background	Lane background subtracted with disk size: 0.1
Lane Width	0.65 mm

Lane 5

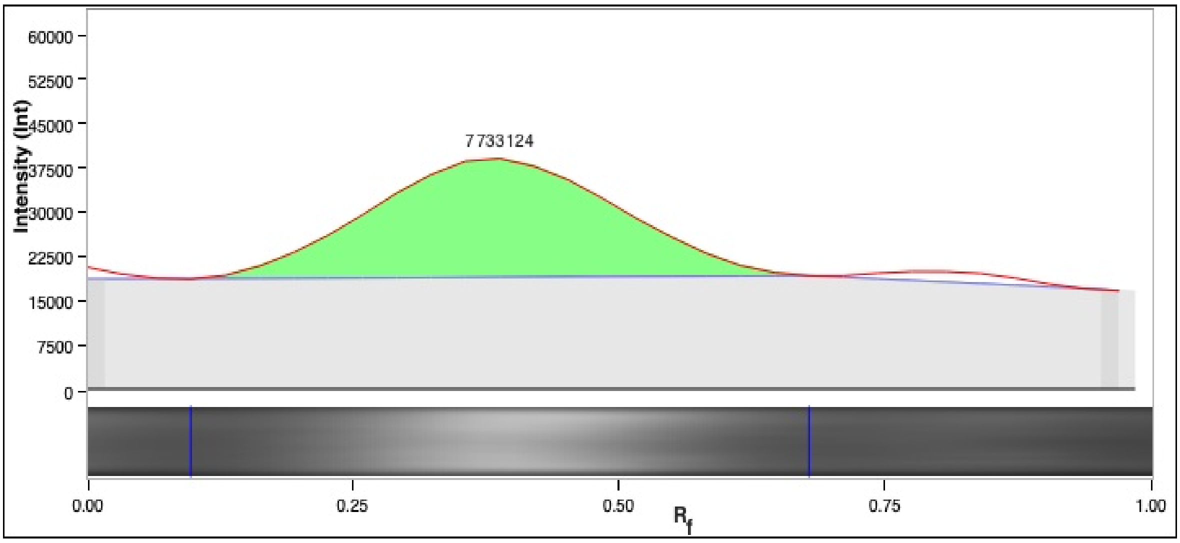

Band No.	Band	Mol. Wt.	Relative	Adj.	Volume	Abs.	Rel.	Band %	Lane %

	Label	(KDa)	Front	Volume (Int)	(Int)	Quant.	Quant.		
1		N/A	0,419	7 733 124	23 444 232	N/A	N/A	100,0	93,9

Lane Background	Lane background subtracted with disk size: 0.1
Lane Width	0.59 mm
