# Supplementary material for: In silico modeling guides identification of novel JAK1 variants associated with immune dysregulation
Source: EMBO Mol Med. 2025 Oct 24;17(12):3275–99. doi: 10.1038/s44321-025-00317-0 (PMC12686074; doi:10.1038/s44321-025-00317-0)
Supplement: Supplementary file 8 — Source data Fig. 3 [file 44321_2025_317_MOESM8_ESM.zip › Figure 3/Replicates Fig.3A/n = 4 & 5/Quantif pSTAT1.pdf]

Image Report: Quantif pSTAT1

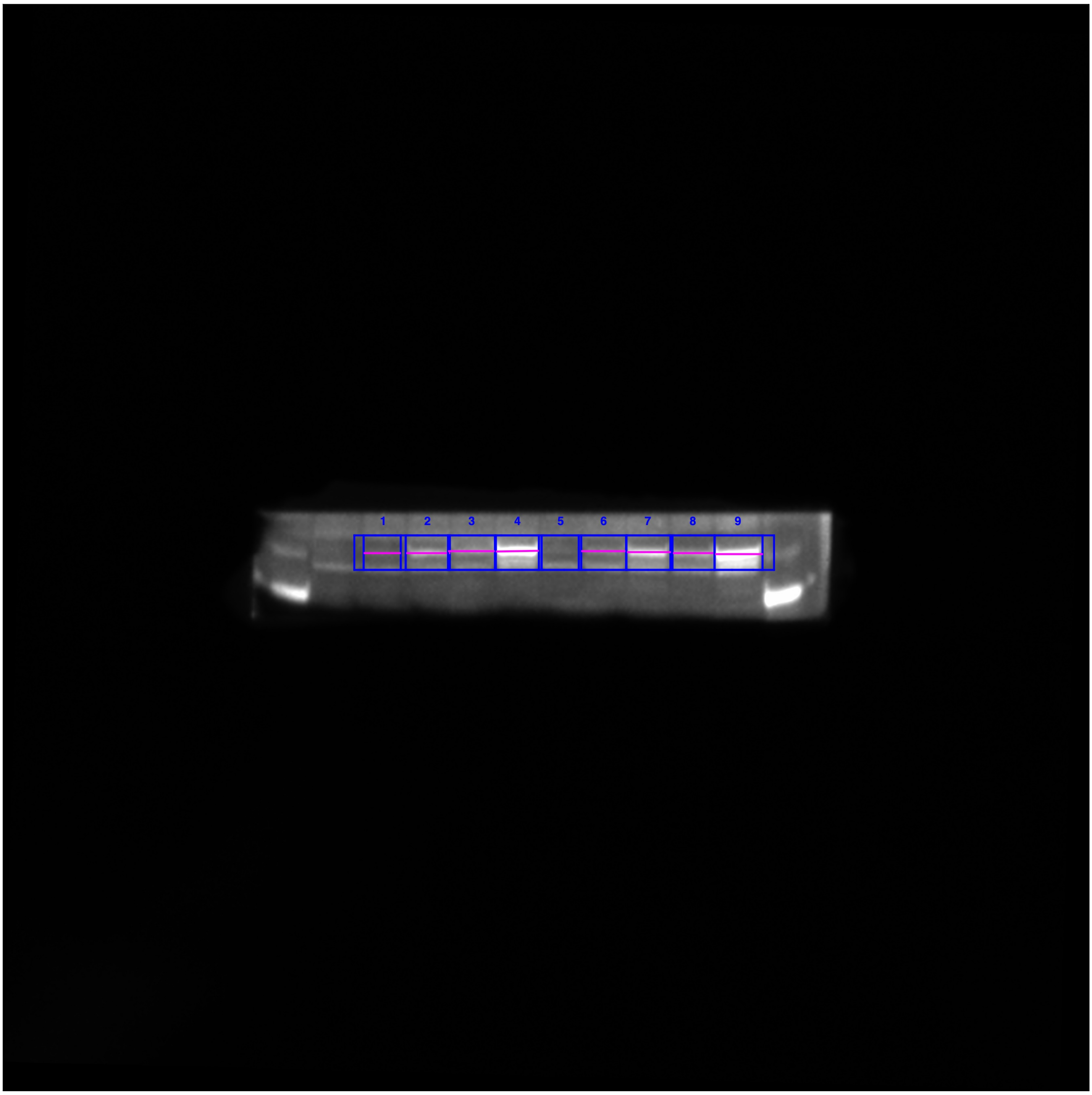

/Volumes/FRL-lab/FRL's Team/Marie Jeanpierre/JAK1/Papier JAK1/Nouvelle submission EMBO/ Source data WB new depot/Quantification Fig.3A/n = 4 & 5/Quantif pSTAT1.scn

Acquisition Information

Image Information

Acquisition Date	unknown
User Name	Marie Jeanpierre

Image Area (mm)	X: 15.7 Y: 15.7
Pixel Size (µm)	X: 14.1 Y: 14.1
Data Range (Int)	0 - 54939

Analysis Settings

Detection	Lane detection:<br>Manually created lanes<br><br>Band detection:<br><br>Manually adjusted bands<br><br>Lane Background Subtraction:<br>Lane background subtracted with disk size: 0.1<br><br>Lane width: Variable

Lane Statistics

Lane No.	Adj. Total Band Vol. (Int)	Total Band Vol. (Int)	Adj. Total Lane Vol. (Int)	Total Lane Vol. (Int)	Bkgd. Vol. (Int)	Norm. Factor
1	540 094	6 554 886	1 144 902	16 811 124	15 666 222	N/A
2	2 744 415	13 208 535	3 175 065	26 397 225	23 222 160	N/A
3	2 009 520	19 140 288	2 379 600	28 169 232	25 789 632	N/A
4	9 080 456	26 803 876	9 712 956	41 254 004	31 541 048	N/A
5	N/A	N/A	1 212 314	18 379 232	17 166 918	N/A
6	1 150 736	11 877 522	1 686 268	24 754 302	23 068 034	N/A
7	4 993 605	20 618 550	5 191 380	31 488 120	26 296 740	N/A
8	2 476 929	14 516 800	2 970 354	24 861 826	21 891 472	N/A
9	12 634 356	41 063 617	12 634 356	45 741 451	33 107 095	N/A

Lane And Band Analysis

Lane 1

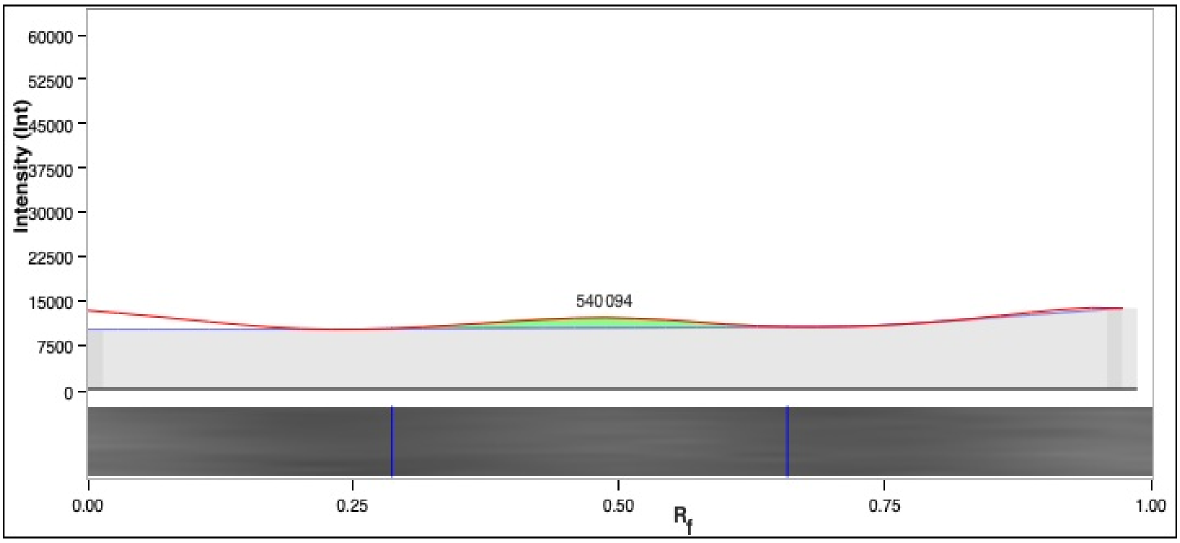

Band No.	Band Label	Mol. Wt. (KDa)	Relative Front	Adj. Volume (Int)	Volume (Int)	Abs. Quant.	Rel. Quant.	Band %	Lane %
1		N/A	0,514	540 094	6 554 886	N/A	N/A	100,0	47,2

Lane Background	Lane background subtracted with disk size: 0.1
Lane Width	0.54 mm

Lane 2

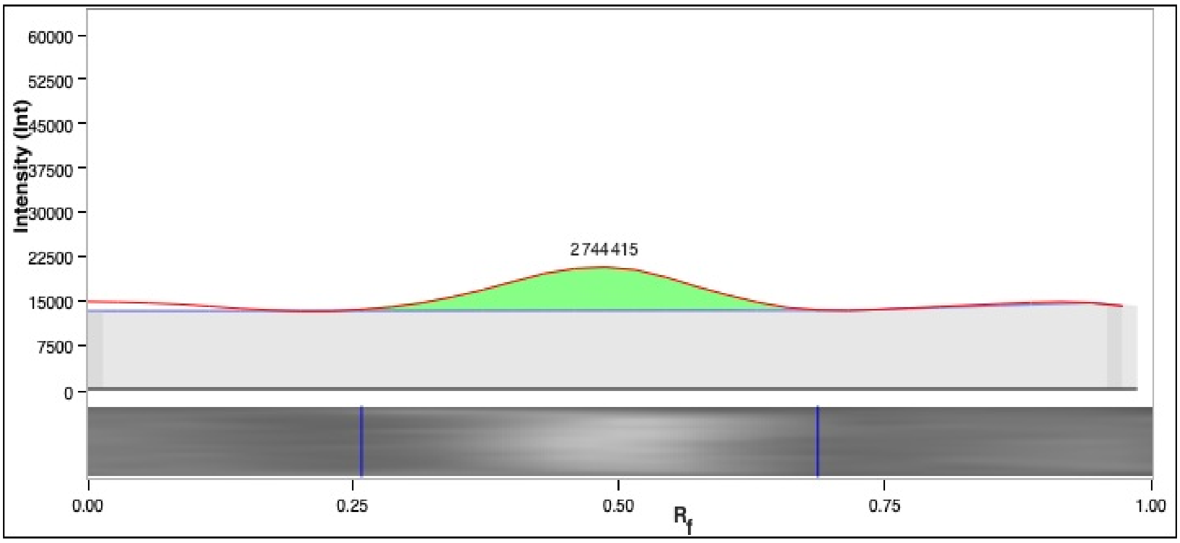

Band No.	Band Label	Mol. Wt. (KDa)	Relative Front	Adj. Volume (Int)	Volume (Int)	Abs. Quant.	Rel. Quant.	Band %	Lane %
1		N/A	0,514	2 744 415	13 208 535	N/A	N/A	100,0	86,4

Lane Background	Lane background subtracted with disk size: 0.1
Lane Width	0.63 mm

Lane 3

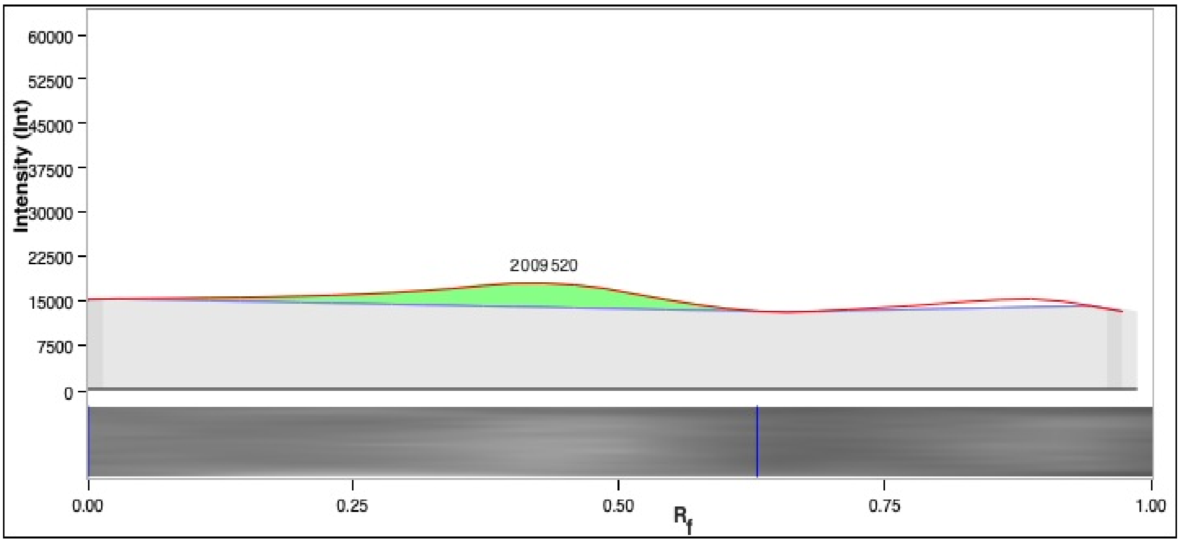

Band No.	Band Label	Mol. Wt. (KDa)	Relative Front	Adj. Volume (Int)	Volume (Int)	Abs. Quant.	Rel. Quant.	Band %	Lane %
1		N/A	0,457	2 009 520	19 140 288	N/A	N/A	100,0	84,4

Lane Background	Lane background subtracted with disk size: 0.1
Lane Width	0.68 mm

Lane 4

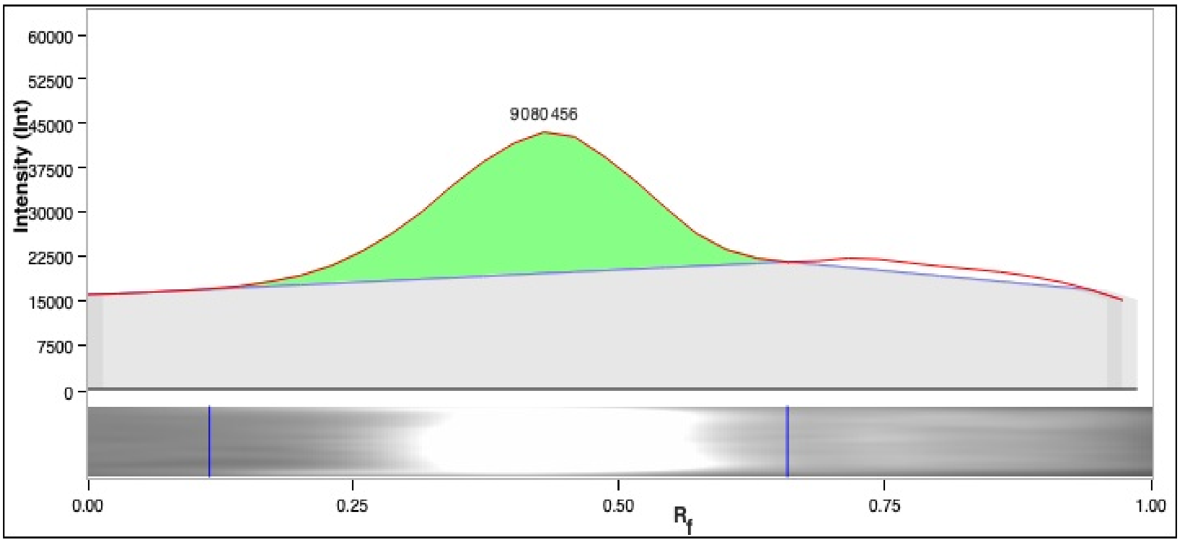

Band No.	Band Label	Mol. Wt. (KDa)	Relative Front	Adj. Volume (Int)	Volume (Int)	Abs. Quant.	Rel. Quant.	Band %	Lane %
1		N/A	0,457	9 080 456	26 803 876	N/A	N/A	100,0	93,5

Lane Background	Lane background subtracted with disk size: 0.1
Lane Width	0.62 mm

Lane 5

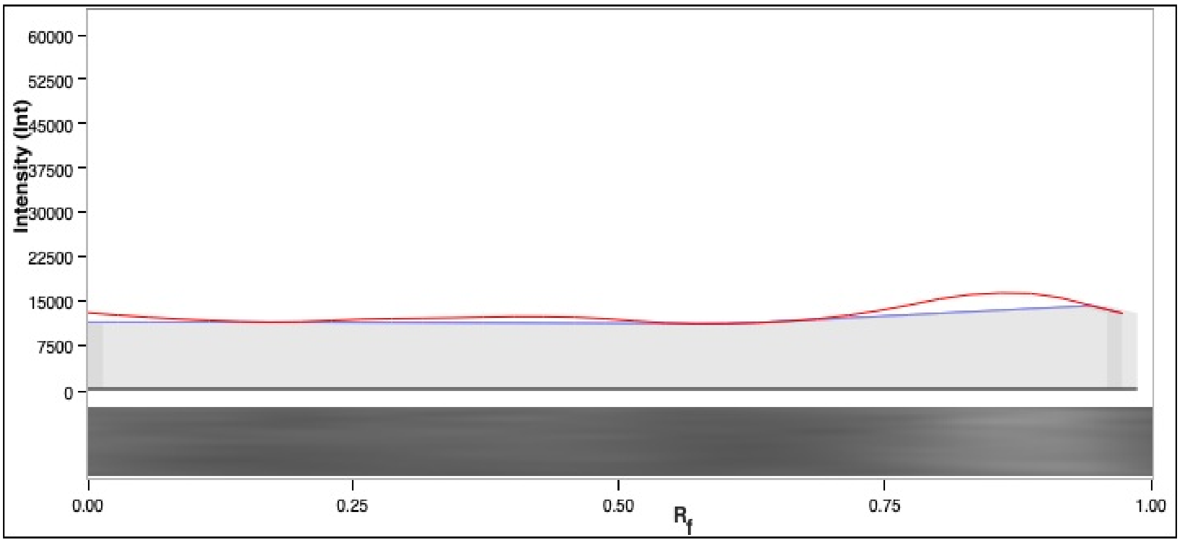

Band No.	Band Label	Mol. Wt. (KDa)	Relative Front	Adj. Volume (Int)	Volume (Int)	Abs. Quant.	Rel. Quant.	Band %	Lane %

Lane Background	Lane background subtracted with disk size: 0.1
Lane Width	0.54 mm

Lane 6

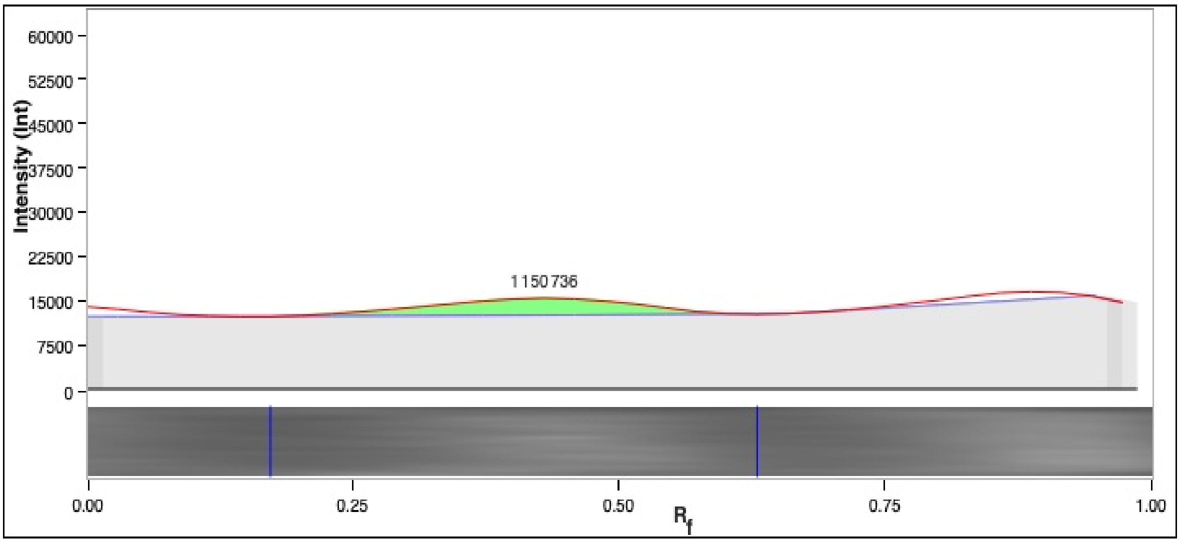

Band No.	Band Label	Mol. Wt. (KDa)	Relative Front	Adj. Volume (Int)	Volume (Int)	Abs. Quant.	Rel. Quant.	Band %	Lane %
1		N/A	0,457	1 150 736	11 877 522	N/A	N/A	100,0	68,2

Lane Background	Lane background subtracted with disk size: 0.1
Lane Width	0.65 mm

Lane 7

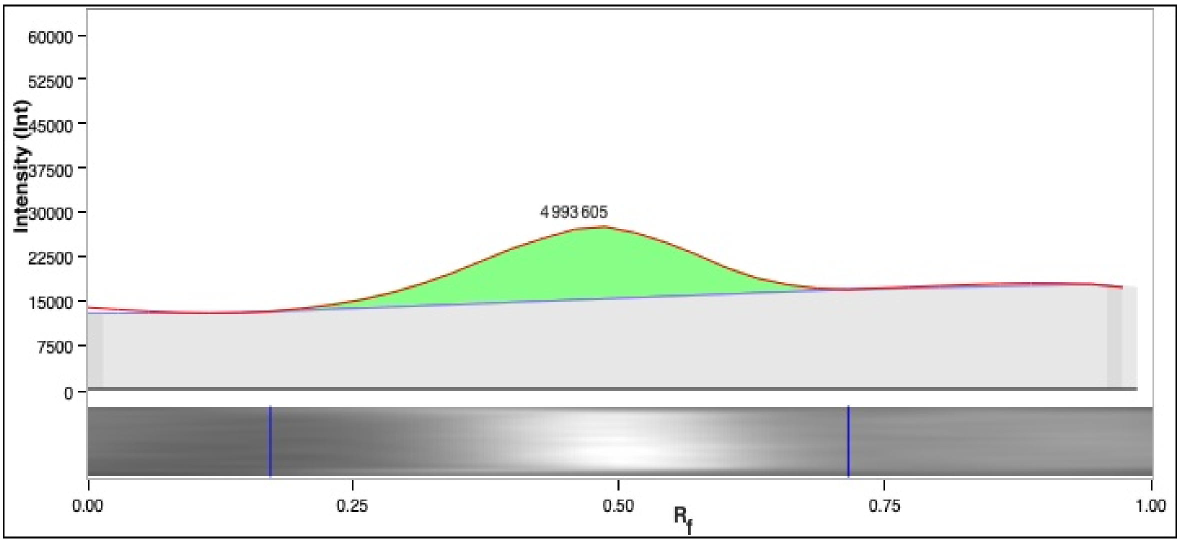

Band No.	Band Label	Mol. Wt. (KDa)	Relative Front	Adj. Volume (Int)	Volume (Int)	Abs. Quant.	Rel. Quant.	Band %	Lane %
1		N/A	0,486	4 993 605	20 618 550	N/A	N/A	100,0	96,2

Lane Background	Lane background subtracted with disk size: 0.1
Lane Width	0.63 mm

Lane 8

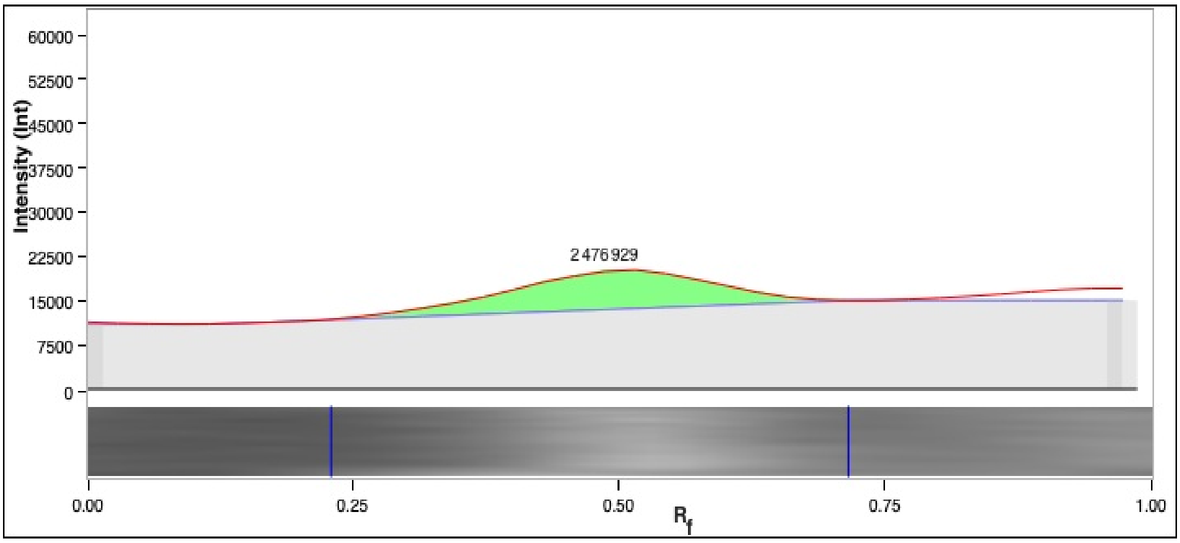

Band No.	Band Label	Mol. Wt. (KDa)	Relative Front	Adj. Volume (Int)	Volume (Int)	Abs. Quant.	Rel. Quant.	Band %	Lane %
1		N/A	0,514	2 476 929	14 516 800	N/A	N/A	100,0	83,4

Lane Background	Lane background subtracted with disk size: 0.1
Lane Width	0.61 mm

Lane 9

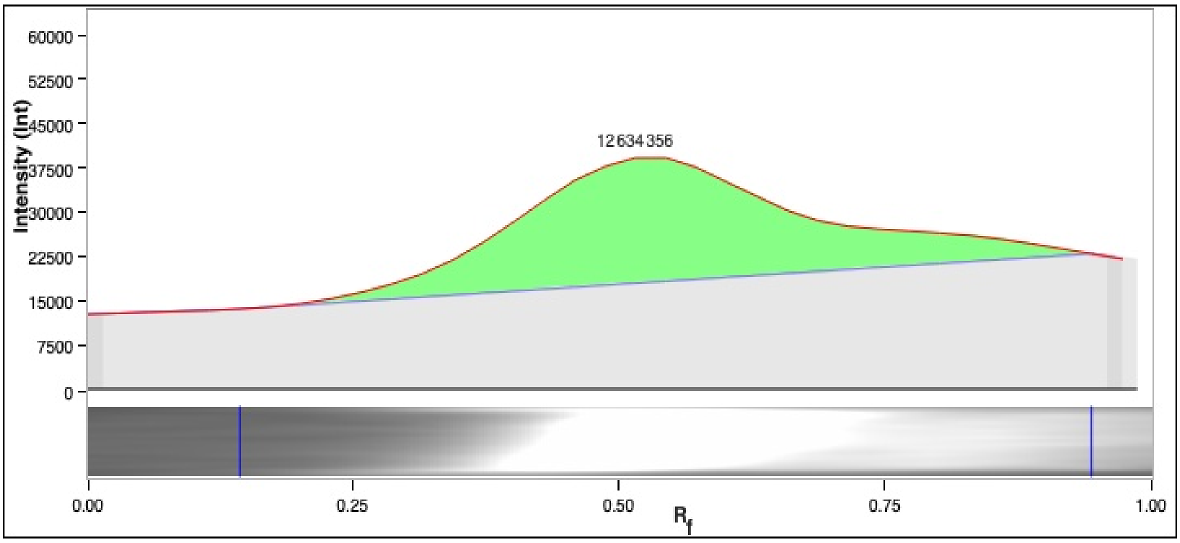

Band No.	Band Label	Mol. Wt. (KDa)	Relative Front	Adj. Volume (Int)	Volume (Int)	Abs. Quant.	Rel. Quant.	Band %	Lane %
1		N/A	0,543	12 634 356	41 063 617	N/A	N/A	100,0	100,0

Lane Background	Lane background subtracted with disk size: 0.1
Lane Width	0.69 mm
