# Supplementary material for: In silico modeling guides identification of novel JAK1 variants associated with immune dysregulation
Source: EMBO Mol Med. 2025 Oct 24;17(12):3275–99. doi: 10.1038/s44321-025-00317-0 (PMC12686074; doi:10.1038/s44321-025-00317-0)
Supplement: Supplementary file 8 — Source data Fig. 3 [file 44321_2025_317_MOESM8_ESM.zip › Figure 3/Replicates Fig.3A/n = 4 & 5/Quantif GAPDH.pdf]

Image Report: Quantif GAPDH

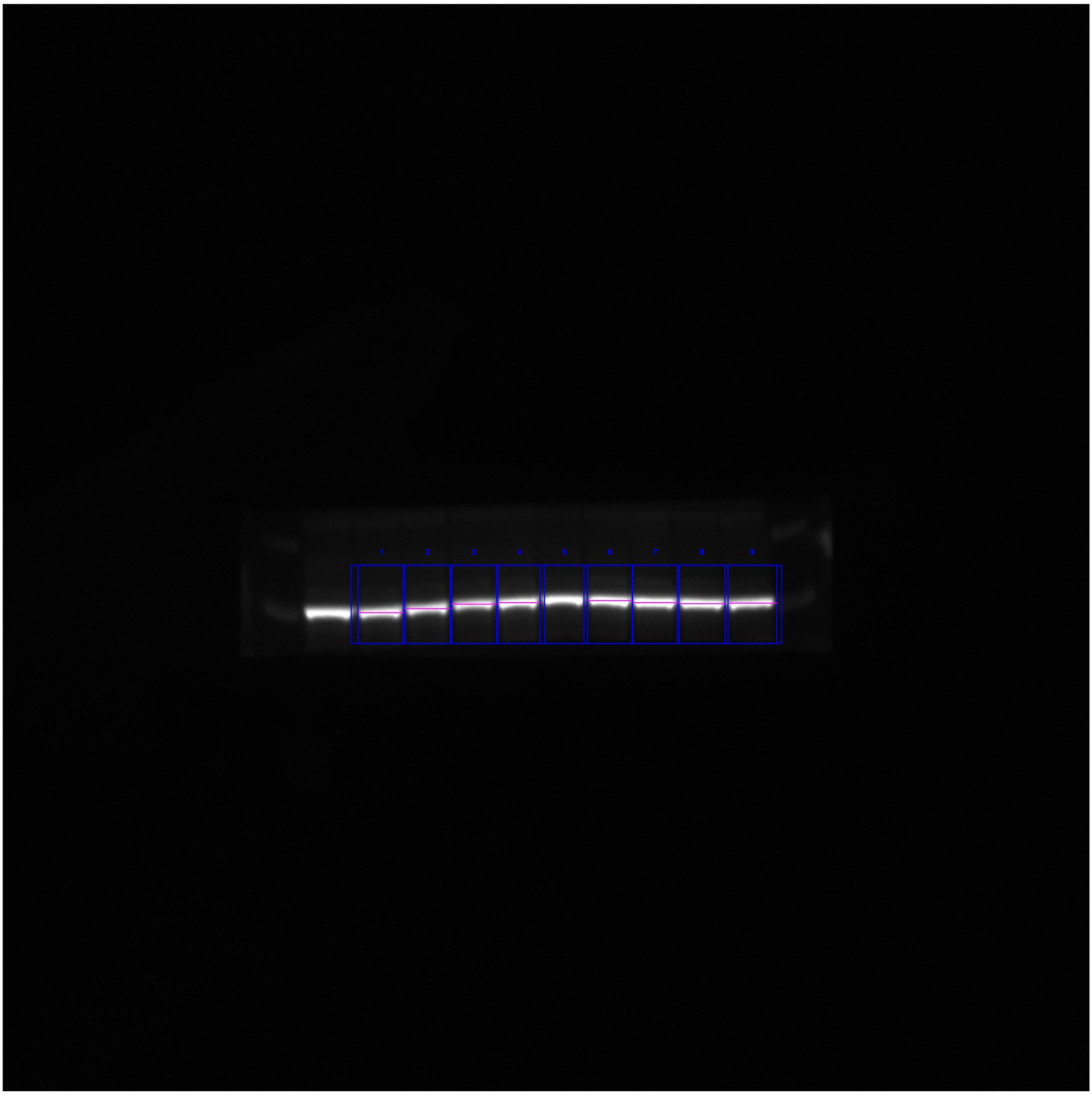

/Volumes/FRL-lab/FRL's Team/Marie Jeanpierre/JAK1/Papier JAK1/Nouvelle submission EMBO/ Source data WB new depot/Quantification Fig.3A/n = 4 & 5/Quantif GAPDH.scn

Acquisition Information

Image Information

Acquisition Date	unknown
User Name	Marie Jeanpierre

Image Area (mm)	X: 30.5 Y: 30.5
Pixel Size (µm)	X: 14.1 Y: 14.1
Data Range (Int)	156 - 15997

Analysis Settings

Detection	Lane detection:<br>Manually created lanes<br><br>Band detection:<br><br>Manually adjusted bands<br><br>Lane Background Subtraction:<br>Lane background subtracted with disk size: 0.1<br><br>Lane width: Variable

Lane Statistics

Lane No.	Adj. Total Band Vol. (Int)	Total Band Vol. (Int)	Adj. Total Lane Vol. (Int)	Total Lane Vol. (Int)	Bkgd. Vol. (Int)	Norm. Factor
1	17 673 210	21 795 840	18 726 390	33 027 030	14 300 640	N/A
2	15 971 580	20 382 930	16 859 610	31 723 470	14 863 860	N/A
3	14 512 429	18 690 534	15 316 633	30 219 505	14 902 872	N/A
4	17 306 856	21 846 888	18 202 380	33 328 764	15 126 384	N/A
5	N/A	N/A	15 943 356	28 884 960	12 941 604	N/A
6	19 200 148	23 856 717	20 222 402	35 492 577	15 270 175	N/A
7	21 015 837	25 537 393	22 136 614	36 730 834	14 594 220	N/A
8	19 804 421	23 844 366	20 770 204	34 308 183	13 537 979	N/A
9	17 714 334	21 424 778	18 434 074	31 216 831	12 782 757	N/A

Lane And Band Analysis

Lane 1

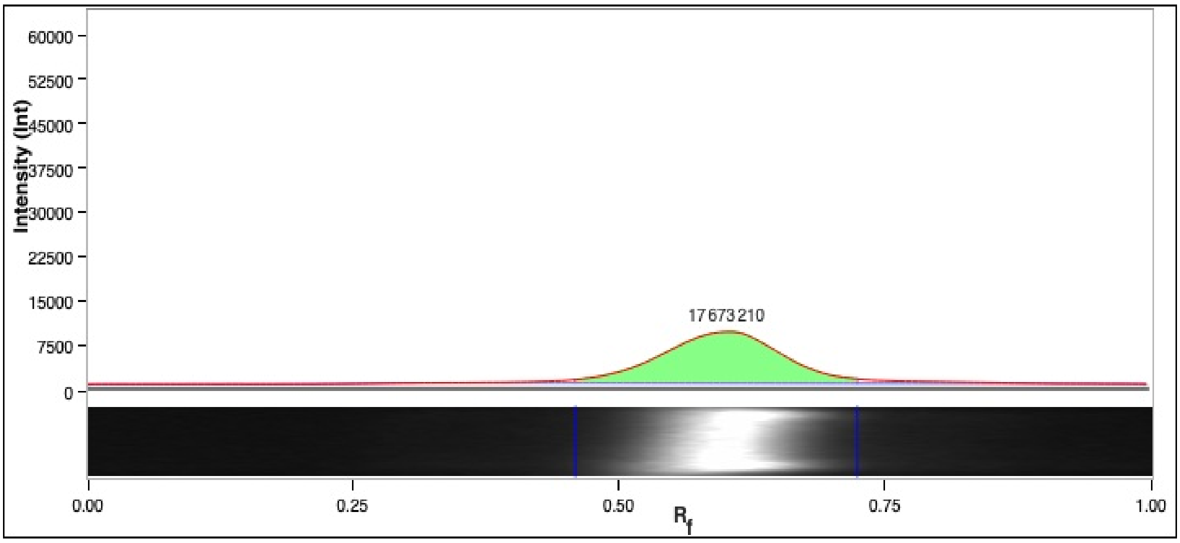

Band No.	Band Label	Mol. Wt. (KDa)	Relative Front	Adj. Volume (Int)	Volume (Int)	Abs. Quant.	Rel. Quant.	Band %	Lane %
1		N/A	0,606	17 673 210	21 795 840	N/A	N/A	100,0	94,4

Lane Background	Lane background subtracted with disk size: 0.1
Lane Width	1.27 mm

Lane 2

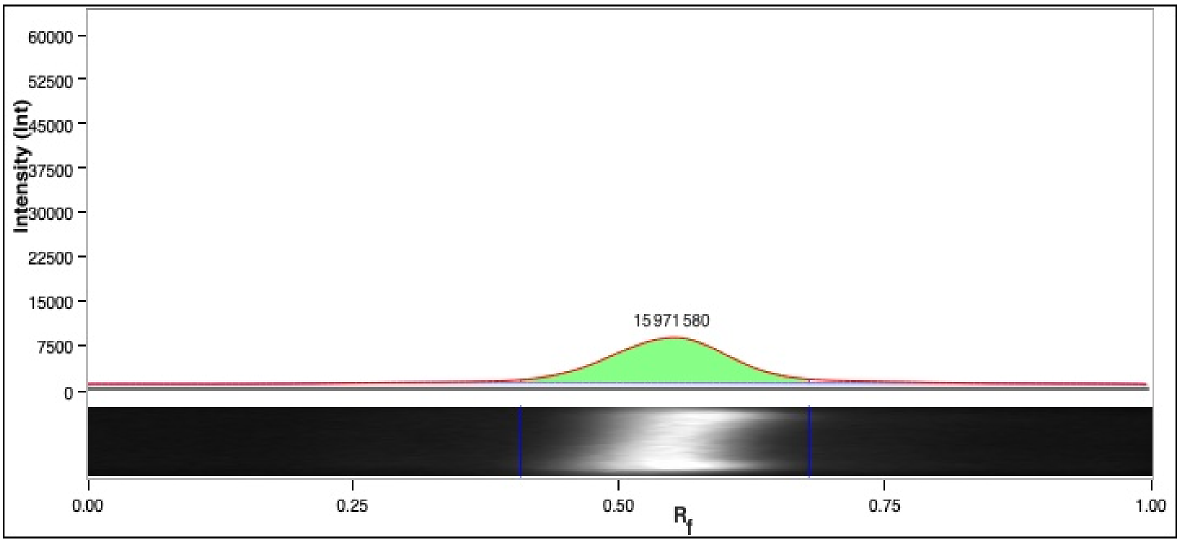

Band No.	Band Label	Mol. Wt. (KDa)	Relative Front	Adj. Volume (Int)	Volume (Int)	Abs. Quant.	Rel. Quant.	Band %	Lane %
1		N/A	0,555	15 971 580	20 382 930	N/A	N/A	100,0	94,7

Lane Background	Lane background subtracted with disk size: 0.1
Lane Width	1.27 mm

Lane 3

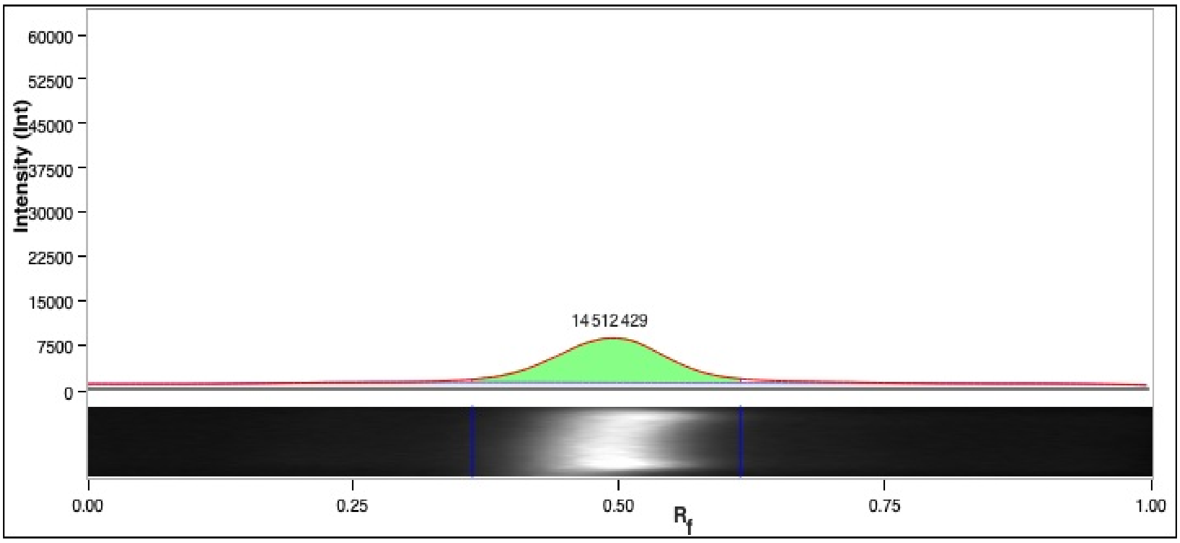

Band No.	Band Label	Mol. Wt. (KDa)	Relative Front	Adj. Volume (Int)	Volume (Int)	Abs. Quant.	Rel. Quant.	Band %	Lane %
1		N/A	0,497	14 512 429	18 690 534	N/A	N/A	100,0	94,7

Lane Background	Lane background subtracted with disk size: 0.1
Lane Width	1.26 mm

Lane 4

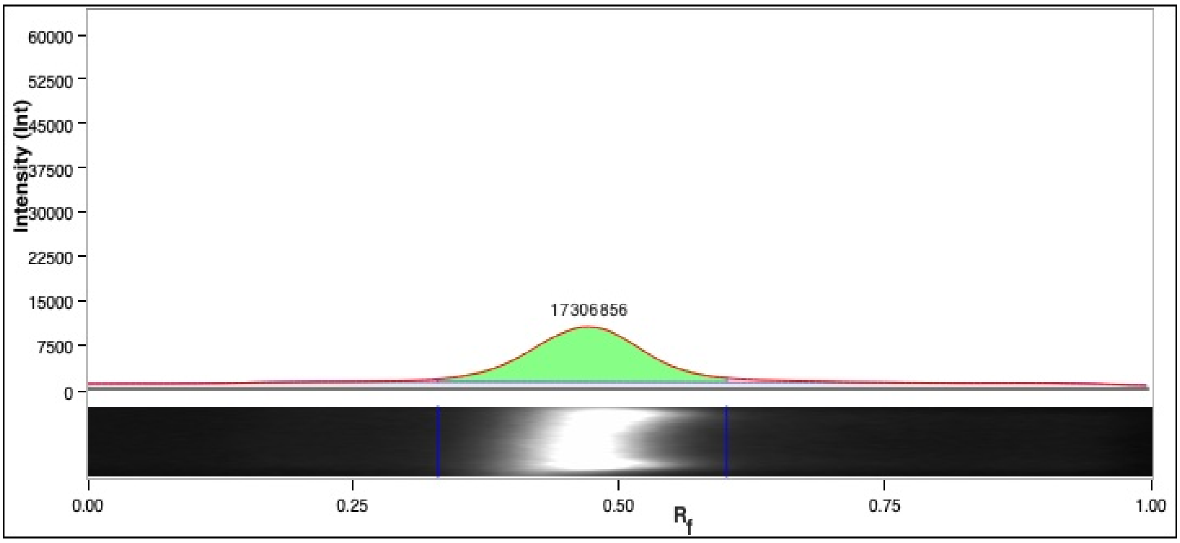

Band No.	Band Label	Mol. Wt. (KDa)	Relative Front	Adj. Volume (Int)	Volume (Int)	Abs. Quant.	Rel. Quant.	Band %	Lane %
1		N/A	0,477	17 306 856	21 846 888	N/A	N/A	100,0	95,1

Lane Background	Lane background subtracted with disk size: 0.1
Lane Width	1.19 mm

Lane 5

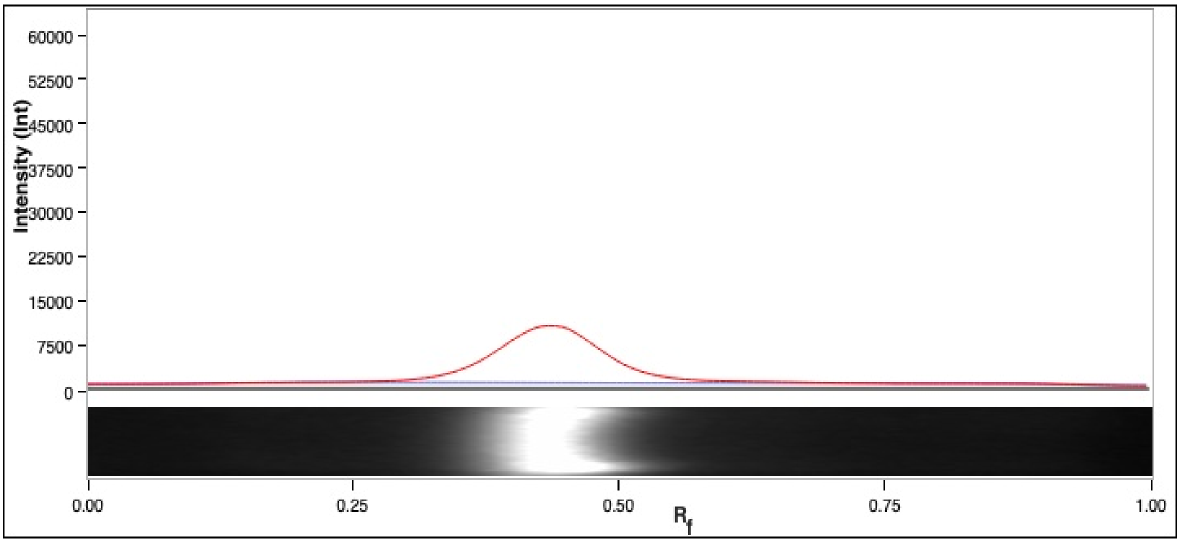

Band No.	Band Label	Mol. Wt. (KDa)	Relative Front	Adj. Volume (Int)	Volume (Int)	Abs. Quant.	Rel. Quant.	Band %	Lane %

Lane Background	Lane background subtracted with disk size: 0.1
Lane Width	1.10 mm

Lane 6

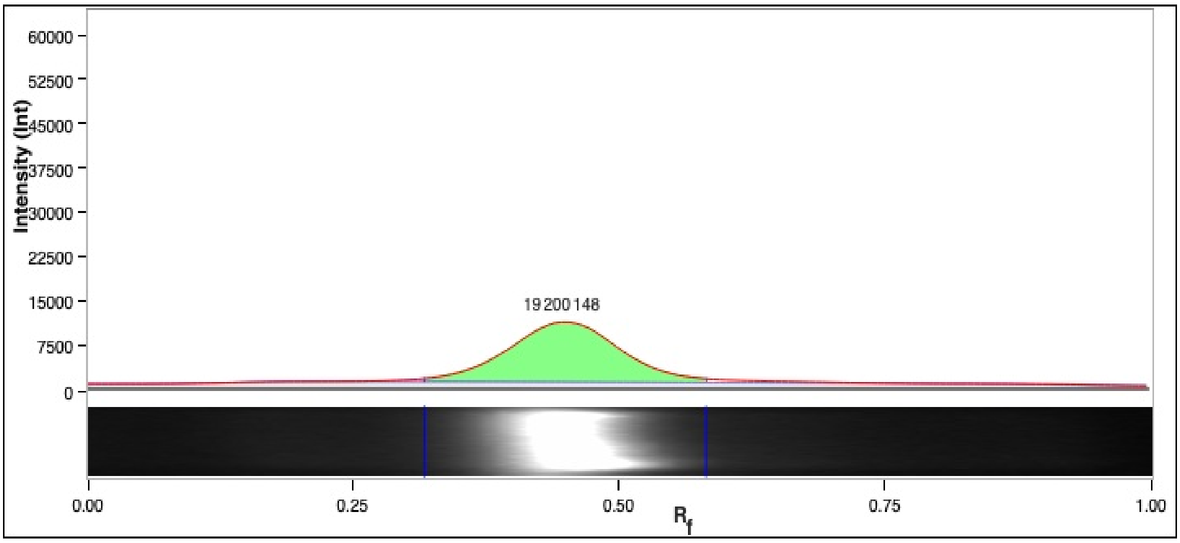

Band No.	Band Label	Mol. Wt. (KDa)	Relative Front	Adj. Volume (Int)	Volume (Int)	Abs. Quant.	Rel. Quant.	Band %	Lane %
1		N/A	0,452	19 200 148	23 856 717	N/A	N/A	100,0	94,9

Lane Background	Lane background subtracted with disk size: 0.1
Lane Width	1.26 mm

Lane 7

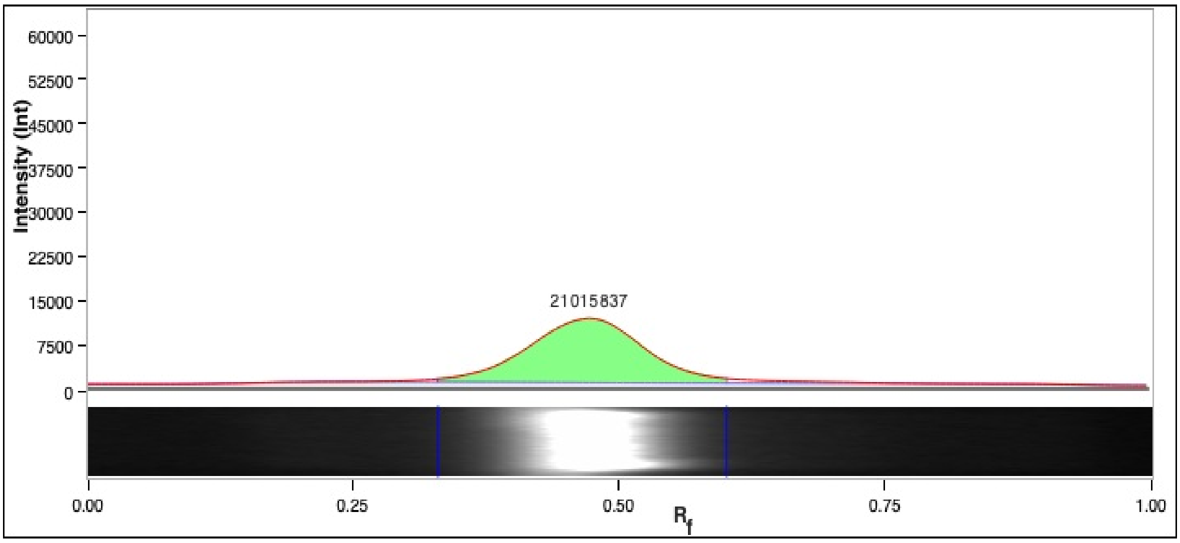

Band No.	Band Label	Mol. Wt. (KDa)	Relative Front	Adj. Volume (Int)	Volume (Int)	Abs. Quant.	Rel. Quant.	Band %	Lane %
1		N/A	0,477	21 015 837	25 537 393	N/A	N/A	100,0	94,9

Lane Background	Lane background subtracted with disk size: 0.1
Lane Width	1.26 mm

Lane 8

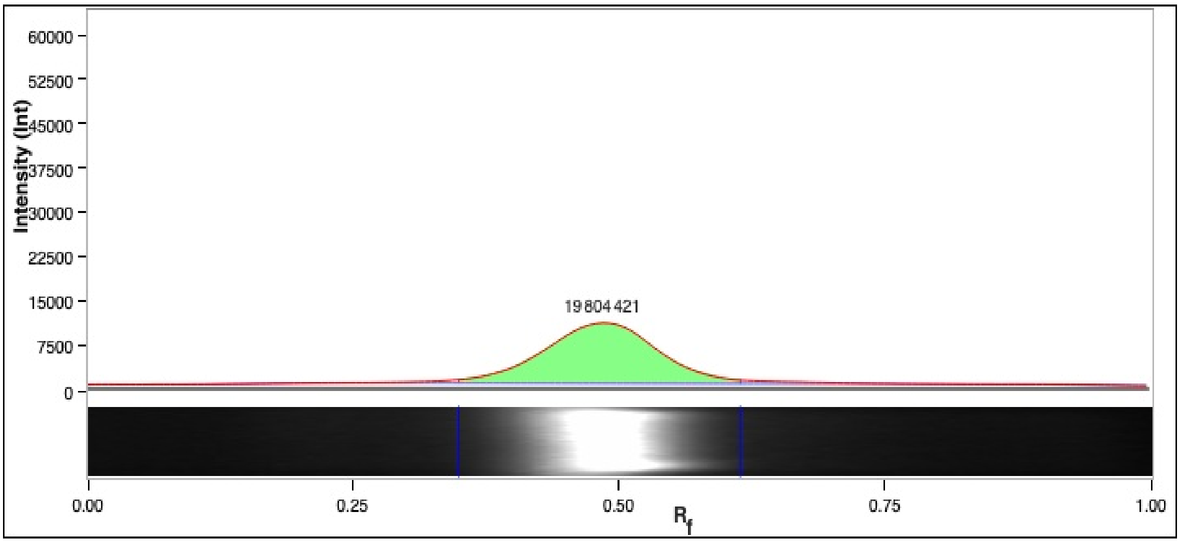

Band No.	Band Label	Mol. Wt. (KDa)	Relative Front	Adj. Volume (Int)	Volume (Int)	Abs. Quant.	Rel. Quant.	Band %	Lane %
1		N/A	0,490	19 804 421	23 844 366	N/A	N/A	100,0	95,4

Lane Background	Lane background subtracted with disk size: 0.1
Lane Width	1.28 mm

Lane 9

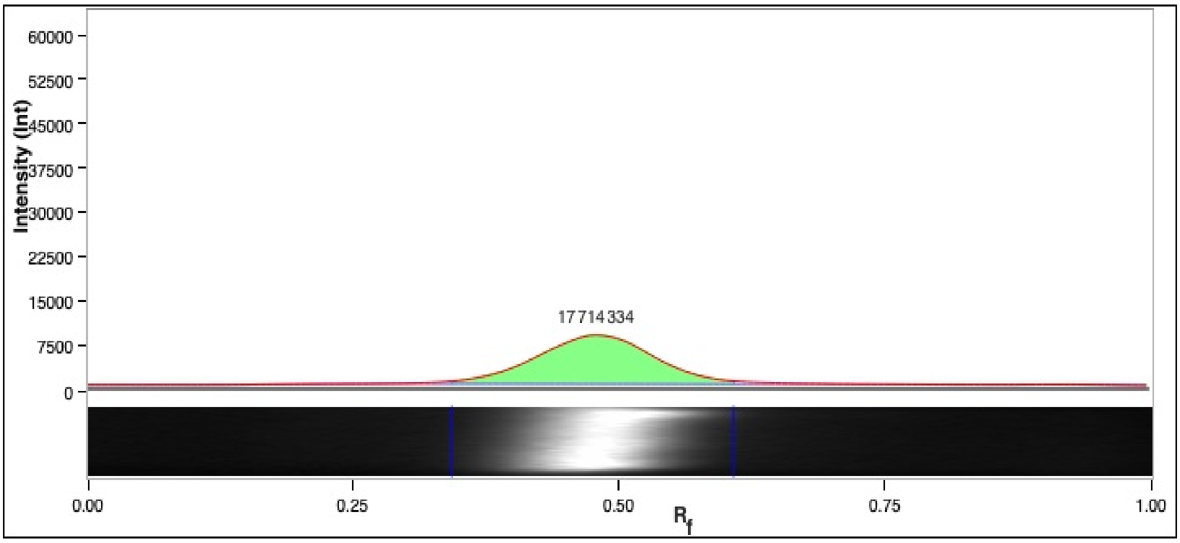

Band No.	Band Label	Mol. Wt. (KDa)	Relative Front	Adj. Volume (Int)	Volume (Int)	Abs. Quant.	Rel. Quant.	Band %	Lane %
1		N/A	0,484	17 714 334	21 424 778	N/A	N/A	100,0	96,1

Lane Background	Lane background subtracted with disk size: 0.1
Lane Width	1.37 mm
