# Supplementary material for: In silico modeling guides identification of novel JAK1 variants associated with immune dysregulation
Source: EMBO Mol Med. 2025 Oct 24;17(12):3275–99. doi: 10.1038/s44321-025-00317-0 (PMC12686074; doi:10.1038/s44321-025-00317-0)
Supplement: Supplementary file 8 — Source data Fig. 3 [file 44321_2025_317_MOESM8_ESM.zip › Figure 3/Replicates Fig.3A/n = 6/pSTAT1 quantif.pdf]

Image Report: IM005215_05Sum2-2 (glissées)

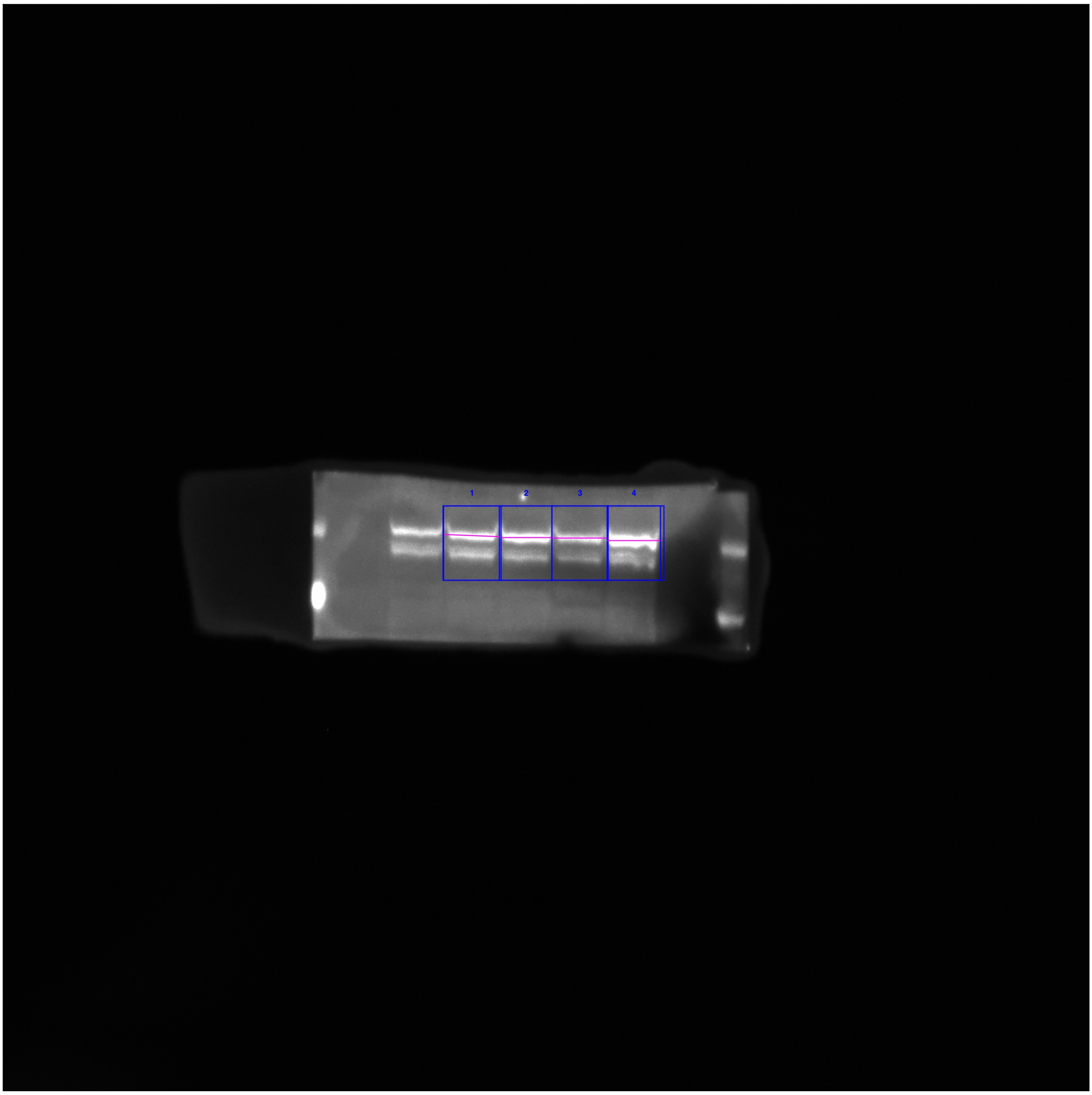

/Users/mariejeanpierre/Desktop/IM005215_05Sum2-2 (glissées).tiff

Acquisition Information

Image Information

Acquisition Date	unknown
User Name	Marie Jeanpierre
Image Area (mm)	X: 30.5 Y: 30.5

Pixel Size (µm)	X: 14.1 Y: 14.1
Data Range (Int)	145 - 60306

Analysis Settings

Detection	Lane detection:<br>Manually created lanes<br><br>Band detection:<br><br>Manually adjusted bands<br><br>Lane Background Subtraction:<br>Lane background subtracted with disk size: 0.1<br><br>Lane width: Variable

Lane Statistics

Lane No.	Adj. Total Band Vol. (Int)	Total Band Vol. (Int)	Adj. Total Lane Vol. (Int)	Total Lane Vol. (Int)	Bkgd. Vol. (Int)	Norm. Factor
1	15 127 635	51 771 177	24 048 927	139 938 033	115 889 106	N/A
2	21 604 708	57 515 561	28 097 796	127 217 580	99 119 784	N/A
3	15 055 824	59 683 344	19 803 952	120 636 544	100 832 592	N/A
4	28 220 698	71 737 514	29 653 606	143 640 282	113 986 676	N/A

Lane And Band Analysis

Lane 1

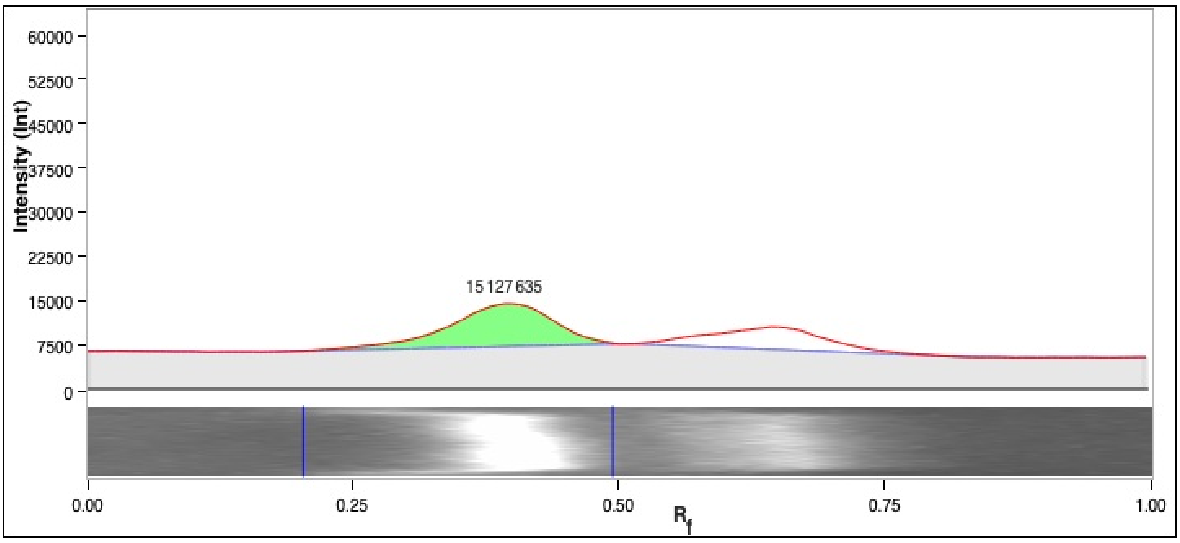

Band No.	Band Label	Mol. Wt. (KDa)	Relative Front	Adj. Volume (Int)	Volume (Int)	Abs. Quant.	Rel. Quant.	Band %	Lane %
1		N/A	0,399	15 127 635	51 771 177	N/A	N/A	100,0	62,9

Lane Background	Lane background subtracted with disk size: 0.1
Lane Width	1.57 mm

Lane 2

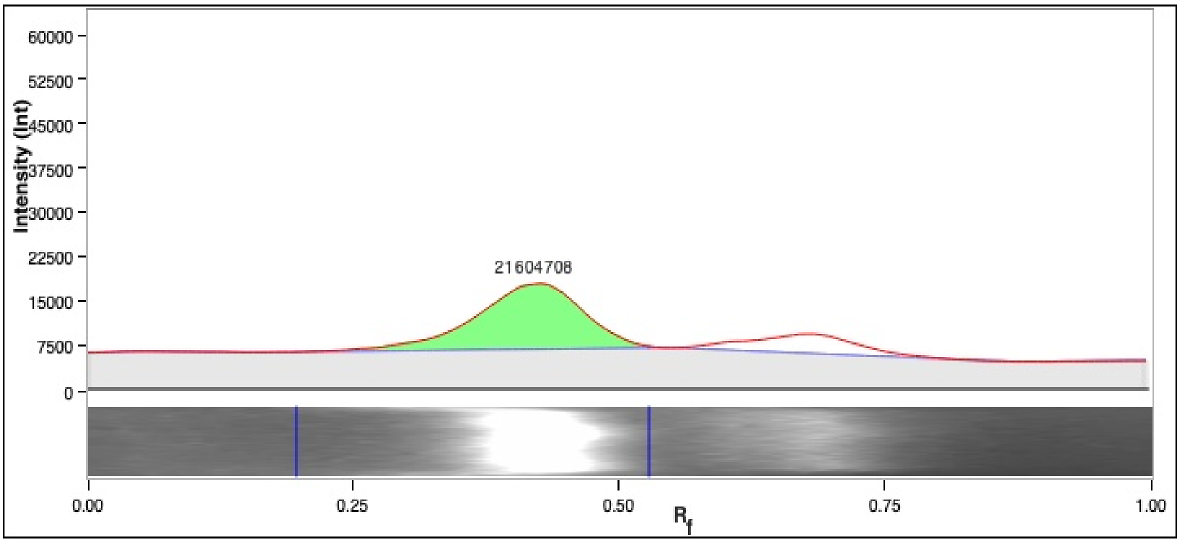

Band No.	Band Label	Mol. Wt. (KDa)	Relative Front	Adj. Volume (Int)	Volume (Int)	Abs. Quant.	Rel. Quant.	Band %	Lane %
1		N/A	0,426	21 604 708	57 515 561	N/A	N/A	100,0	76,9

Lane Background	Lane background subtracted with disk size: 0.1
Lane Width	1.43 mm

Lane 3

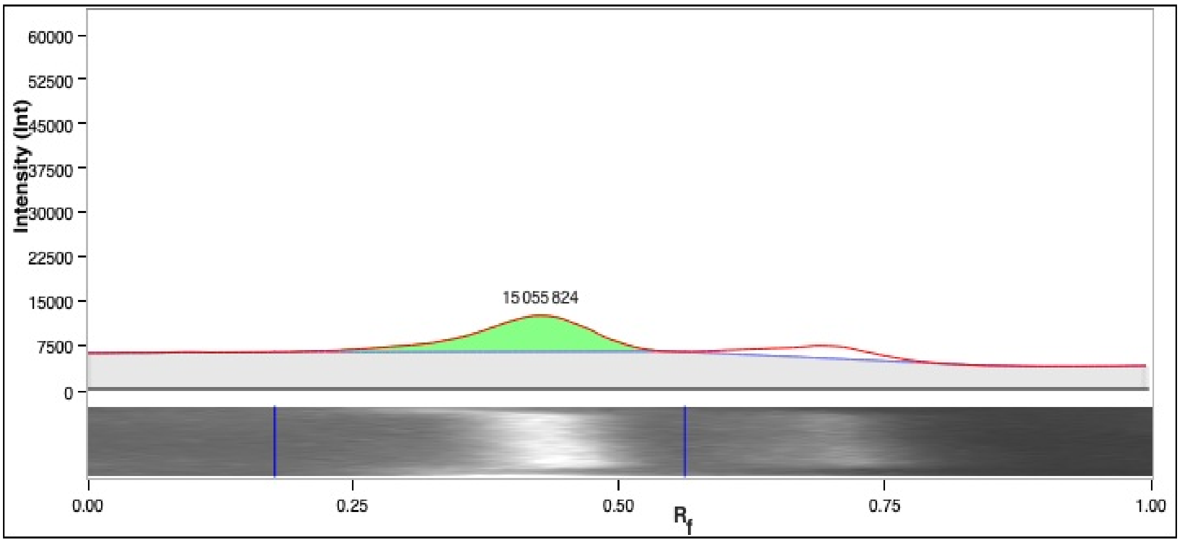

Band No.	Band Label	Mol. Wt. (KDa)	Relative Front	Adj. Volume (Int)	Volume (Int)	Abs. Quant.	Rel. Quant.	Band %	Lane %
1		N/A	0,432	15 055 824	59 683 344	N/A	N/A	100,0	76,0

Lane Background	Lane background subtracted with disk size: 0.1

Lane Width	1.58 mm

Lane 4

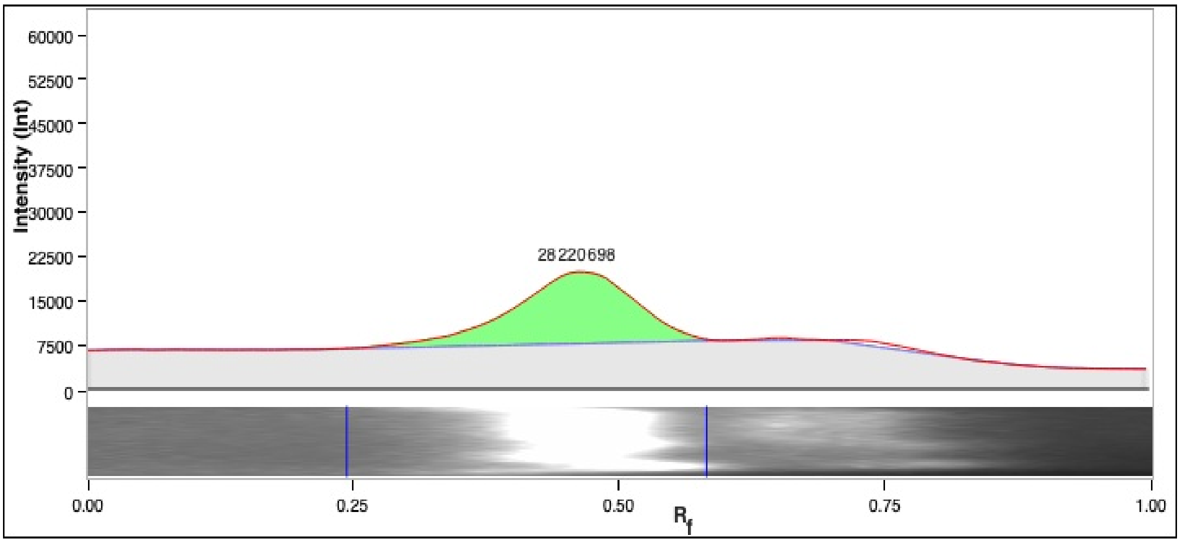

Band No.	Band Label	Mol. Wt. (KDa)	Relative Front	Adj. Volume (Int)	Volume (Int)	Abs. Quant.	Rel. Quant.	Band %	Lane %
1		N/A	0,466	28 220 698	71 737 514	N/A	N/A	100,0	95,2

Lane Background	Lane background subtracted with disk size: 0.1
Lane Width	1.50 mm
