# Supplementary material for: In silico modeling guides identification of novel JAK1 variants associated with immune dysregulation
Source: EMBO Mol Med. 2025 Oct 24;17(12):3275–99. doi: 10.1038/s44321-025-00317-0 (PMC12686074; doi:10.1038/s44321-025-00317-0)
Supplement: Supplementary file 8 — Source data Fig. 3 [file 44321_2025_317_MOESM8_ESM.zip › Figure 3/Replicates Fig.3A/n = 6/GAPDH quantif.pdf]

Image Report: IM005222_02Sum-2 (glissées)

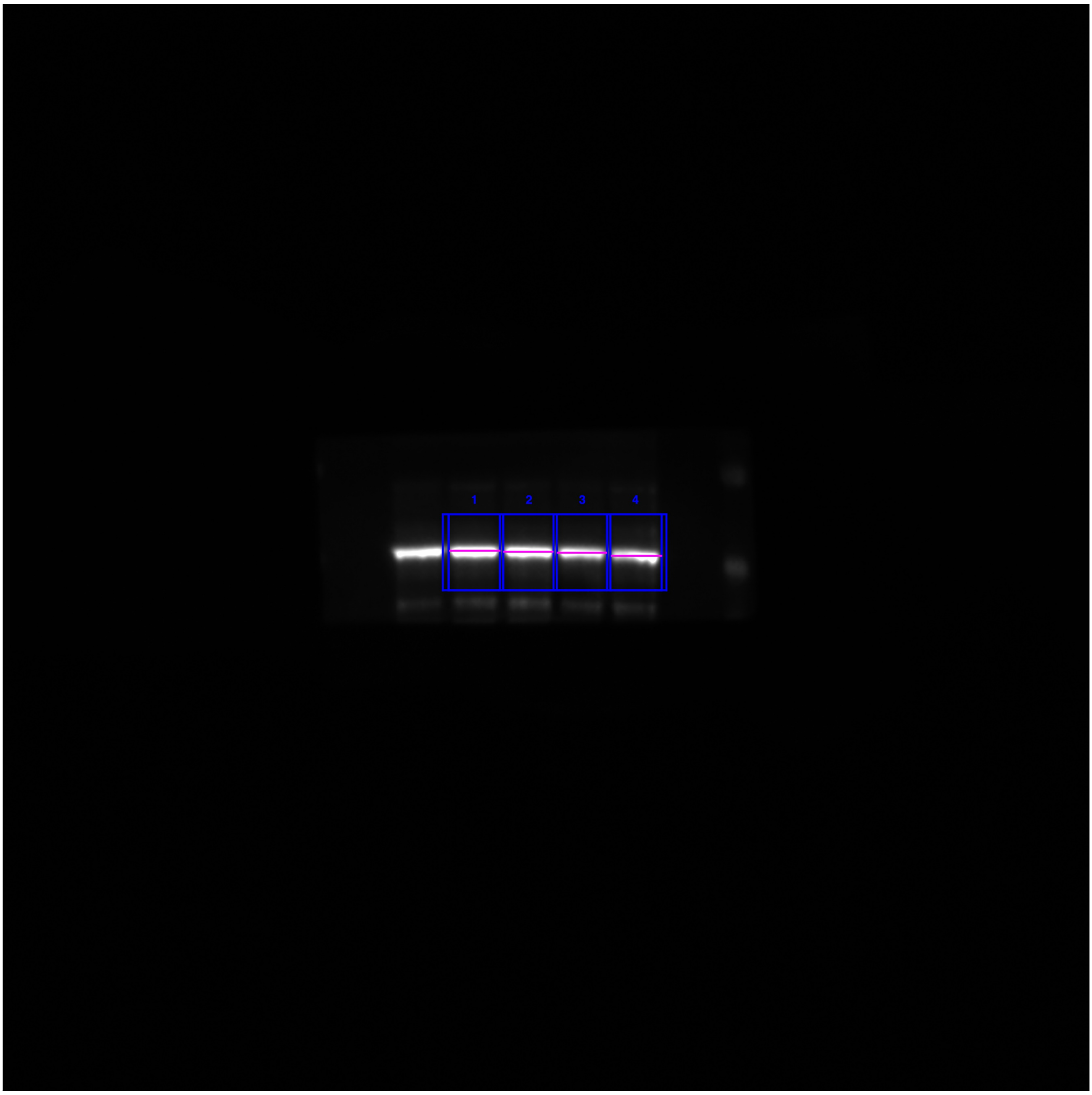

/Users/mariejeanpierre/Desktop/IM005222_02Sum-2 (glissées).tiff

Acquisition Information

Image Information

Acquisition Date	unknown
User Name	Marie Jeanpierre
Image Area (mm)	X: 15.2 Y: 15.2

Pixel Size (µm)	X: 14.1 Y: 14.1
Data Range (Int)	130 - 52235

Analysis Settings

Detection	Lane detection:<br>Manually created lanes<br><br>Band detection:<br><br>Manually adjusted bands<br><br>Lane Background Subtraction:<br>Lane background subtracted with disk size: 0.1<br><br>Lane width: Variable

Lane Statistics

Lane No.	Adj. Total Band Vol. (Int)	Total Band Vol. (Int)	Adj. Total Lane Vol. (Int)	Total Lane Vol. (Int)	Bkgd. Vol. (Int)	Norm. Factor
1	24 582 969	27 499 608	26 275 710	33 834 012	7 558 302	N/A
2	22 818 300	25 769 750	24 534 250	32 193 050	7 658 800	N/A
3	17 831 850	20 070 850	19 604 850	25 823 200	6 218 350	N/A
4	17 196 180	20 774 391	18 099 747	27 221 046	9 121 299	N/A

Lane And Band Analysis

Lane 1

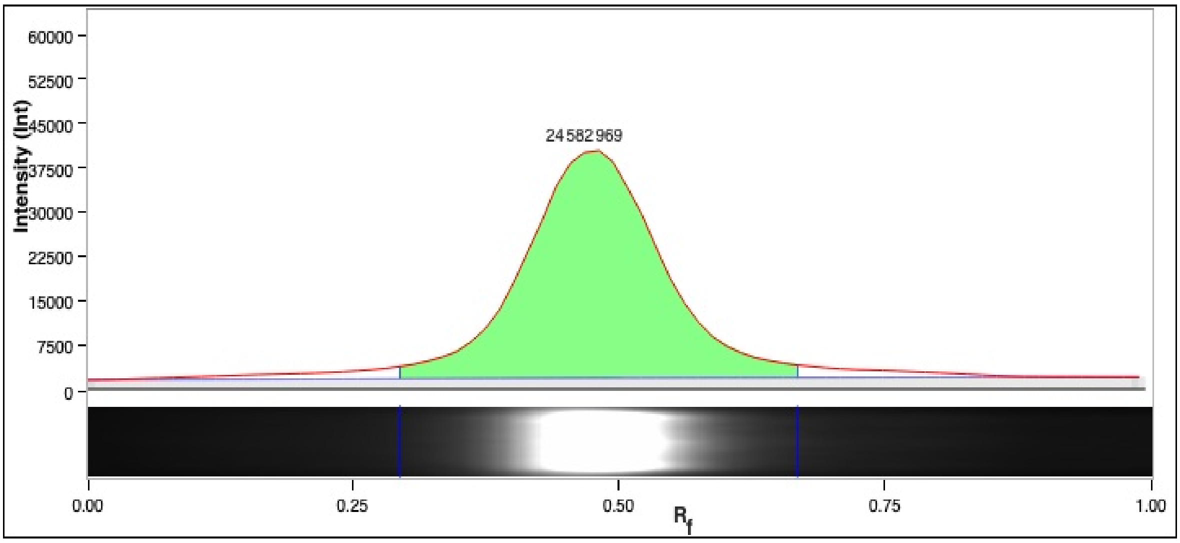

Band No.	Band Label	Mol. Wt. (KDa)	Relative Front	Adj. Volume (Int)	Volume (Int)	Abs. Quant.	Rel. Quant.	Band %	Lane %
1		N/A	0,480	24 582 969	27 499 608	N/A	N/A	100,0	93,6

Lane Background	Lane background subtracted with disk size: 0.1
Lane Width	0.72 mm

Lane 2

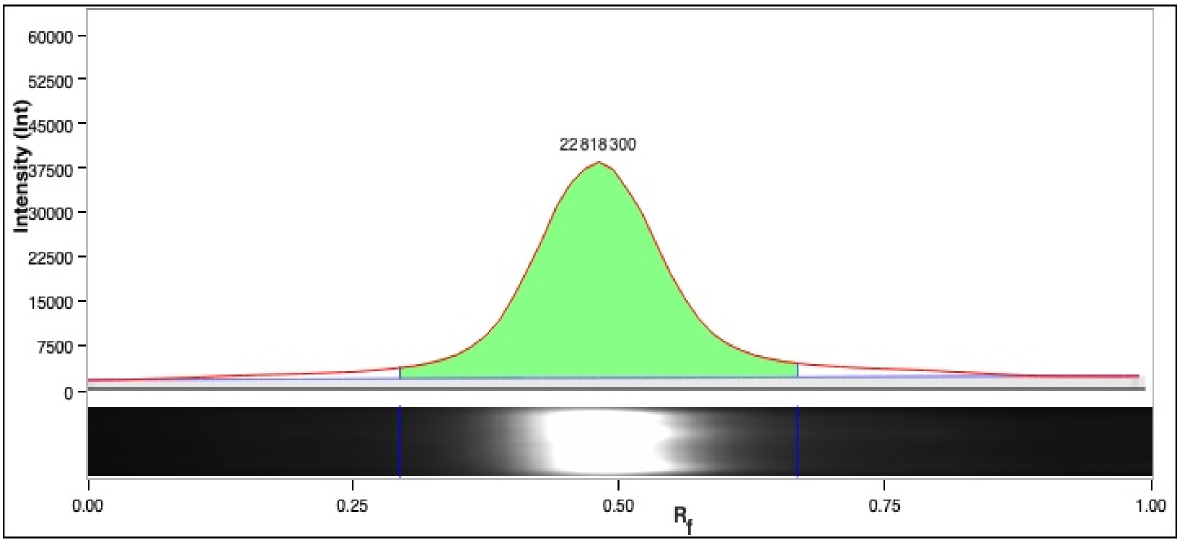

Band No.	Band Label	Mol. Wt. (KDa)	Relative Front	Adj. Volume (Int)	Volume (Int)	Abs. Quant.	Rel. Quant.	Band %	Lane %
1		N/A	0,493	22 818 300	25 769 750	N/A	N/A	100,0	93,0

Lane Background	Lane background subtracted with disk size: 0.1
Lane Width	0.71 mm

Lane 3

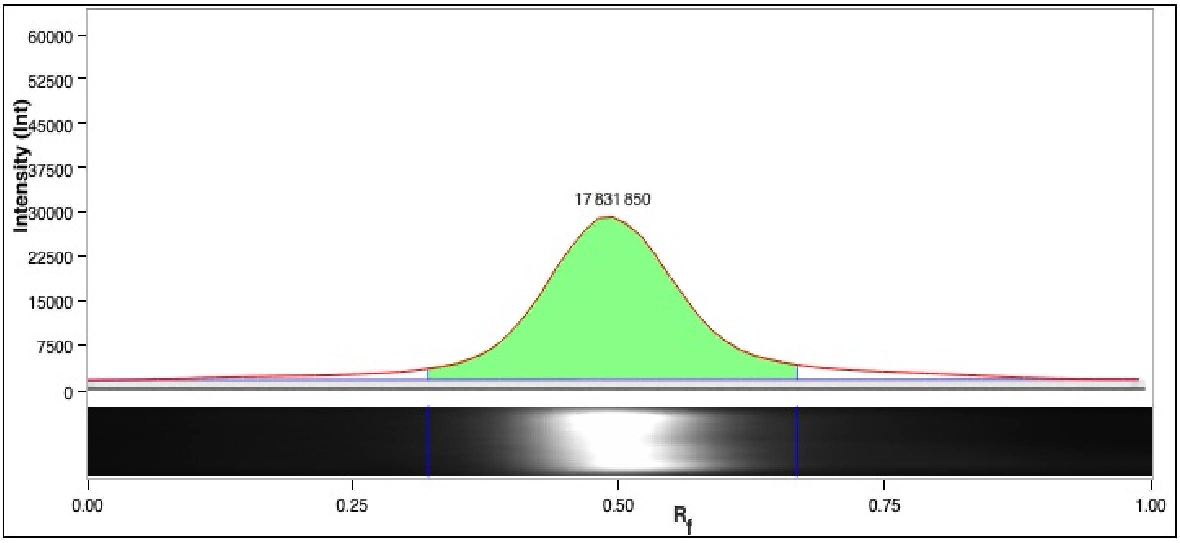

Band No.	Band Label	Mol. Wt. (KDa)	Relative Front	Adj. Volume (Int)	Volume (Int)	Abs. Quant.	Rel. Quant.	Band %	Lane %
1		N/A	0,507	17 831 850	20 070 850	N/A	N/A	100,0	91,0

Lane Background	Lane background subtracted with disk size: 0.1

Lane Width	0.71 mm

Lane 4

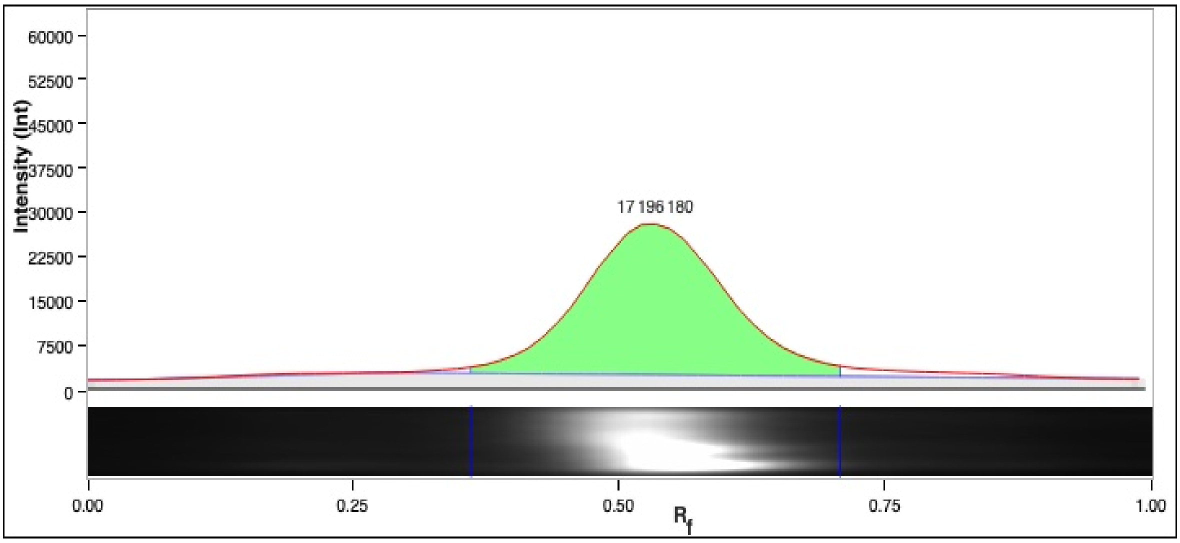

Band No.	Band Label	Mol. Wt. (KDa)	Relative Front	Adj. Volume (Int)	Volume (Int)	Abs. Quant.	Rel. Quant.	Band %	Lane %
1		N/A	0,547	17 196 180	20 774 391	N/A	N/A	100,0	95,0

Lane Background	Lane background subtracted with disk size: 0.1
Lane Width	0.72 mm
