# Supplementary material for: In silico modeling guides identification of novel JAK1 variants associated with immune dysregulation
Source: EMBO Mol Med. 2025 Oct 24;17(12):3275–99. doi: 10.1038/s44321-025-00317-0 (PMC12686074; doi:10.1038/s44321-025-00317-0)
Supplement: Supplementary file 8 — Source data Fig. 3 [file 44321_2025_317_MOESM8_ESM.zip › Figure 3/Replicates Fig.3A/wb 1/20241205 GAPDH.pdf]

Image Report: GAPDH

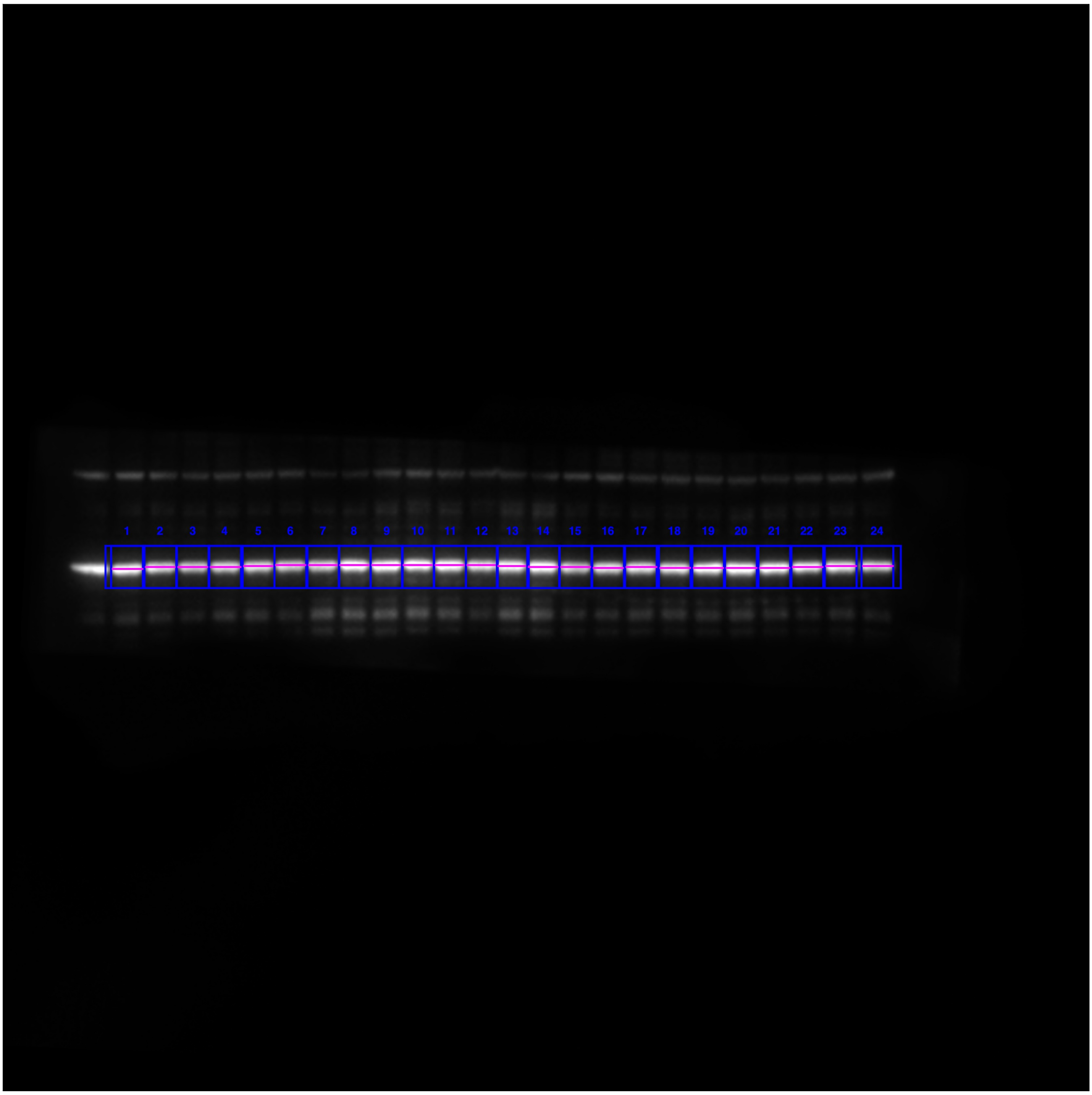

/Volumes/FRL-lab/FRL's Team/Marie Jeanpierre/JAK1/Papier JAK1/Nouvelle submission EMBO/ Source data/Figure 3/3A/Western n1 /Membrane 1/Quantification /GAPDH.scn

---

Acquisition Information

Image Information

Acquisition Date	unknown
User Name	Marie Jeanpierre

Image Area (mm)	X: 15.9 Y: 15.9
Pixel Size (µm)	X: 14.1 Y: 14.1
Data Range (Int)	0 - 54085

Analysis Settings

Detection	Lane detection:<br>Manually created lanes<br><br>Band detection:<br><br>Manually adjusted bands<br><br>Lane Background Subtraction:<br>Lane background subtracted with disk size: 0.1<br><br>Lane width: 0.47 mm

Lane Statistics

Lane No.	Adj. Total Band Vol. (Int)	Total Band Vol. (Int)	Adj. Total Lane Vol. (Int)	Total Lane Vol. (Int)	Bkgd. Vol. (Int)	Norm. Factor
1	15 130 665	19 607 478	15 636 027	22 957 935	7 321 908	N/A
2	12 686 850	16 714 302	13 094 697	19 894 116	6 799 419	N/A
3	11 398 101	14 882 043	11 854 887	17 977 509	6 122 622	N/A
4	12 664 773	16 617 513	13 065 789	19 503 726	6 437 937	N/A
5	13 467 630	17 533 890	13 824 954	20 025 258	6 200 304	N/A
6	12 578 973	16 241 247	12 979 692	18 740 997	5 761 305	N/A
7	12 999 129	17 492 871	13 393 446	20 231 310	6 837 864	N/A
8	15 910 983	21 548 307	16 474 194	24 757 722	8 283 528	N/A
9	16 449 576	22 986 546	16 929 198	26 216 949	9 287 751	N/A
10	15 467 199	21 770 760	16 078 689	25 299 846	9 221 157	N/A
11	15 766 311	21 277 707	16 478 286	24 821 841	8 343 555	N/A
12	13 877 523	18 701 892	14 412 222	21 786 270	7 374 048	N/A
13	15 290 055	21 166 101	15 680 115	24 412 806	8 732 691	N/A
14	15 015 759	21 774 984	15 077 997	25 061 751	9 983 754	N/A
15	12 183 336	16 074 300	12 418 461	18 521 976	6 103 515	N/A
16	14 131 953	17 732 583	14 336 256	19 603 221	5 266 965	N/A
17	14 441 856	18 174 156	14 667 576	20 127 657	5 460 081	N/A
18	14 233 692	18 521 646	14 499 441	20 965 527	6 466 086	N/A
19	16 327 872	20 779 374	16 511 847	22 792 077	6 280 230	N/A
20	17 343 018	22 153 329	17 510 295	24 287 637	6 777 342	N/A
21	16 607 316	20 349 516	16 842 903	22 319 187	5 476 284	N/A
22	15 090 966	17 965 200	15 318 171	19 521 579	4 203 408	N/A
23	15 340 875	18 310 116	15 659 688	20 162 274	4 502 586	N/A
24	13 319 526	16 077 270	13 518 681	17 678 463	4 159 782	N/A

Lane And Band Analysis

Lane 1

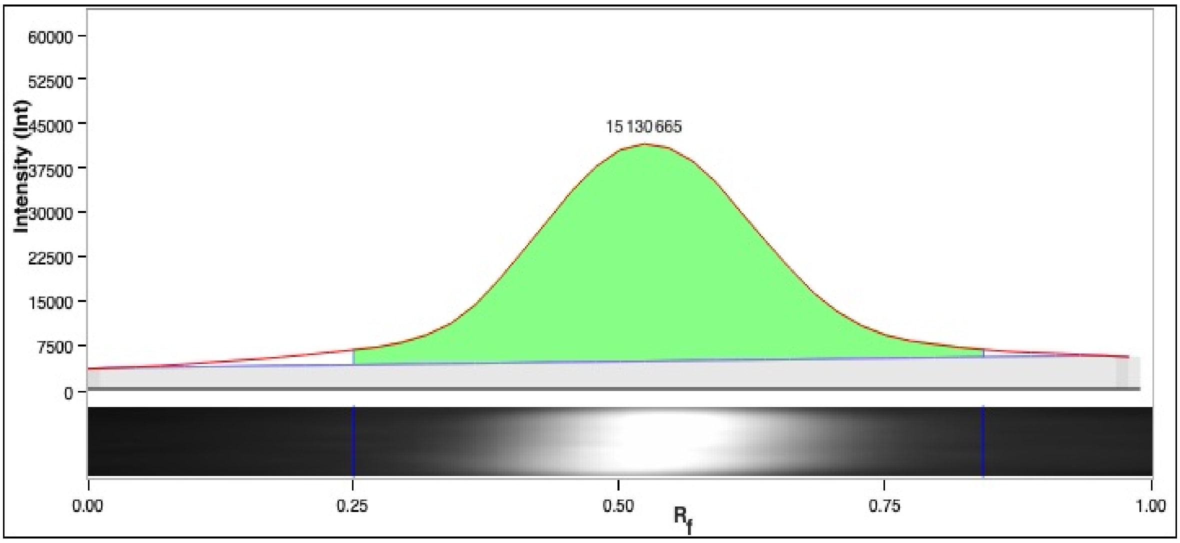

Band No.	Band Label	Mol. Wt. (KDa)	Relative Front	Adj. Volume (Int)	Volume (Int)	Abs. Quant.	Rel. Quant.	Band %	Lane %
1		N/A	0,545	15 130 665	19 607 478	N/A	N/A	100,0	96,8

Lane Background	Lane background subtracted with disk size: 0.1
Lane Width	0.47 mm

Lane 2

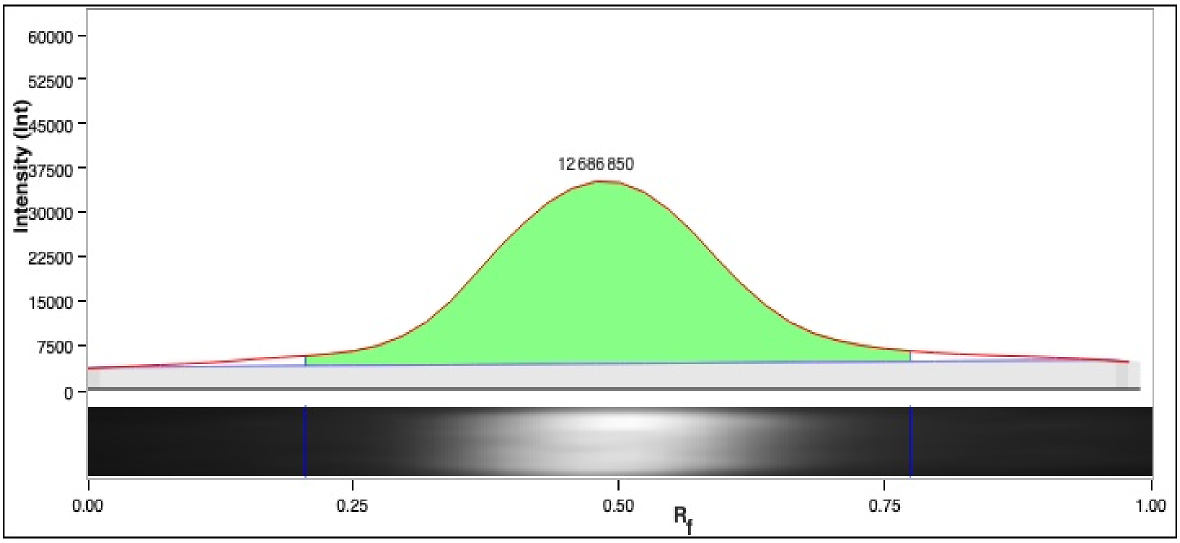

Band No.	Band Label	Mol. Wt. (KDa)	Relative Front	Adj. Volume (Int)	Volume (Int)	Abs. Quant.	Rel. Quant.	Band %	Lane %
1		N/A	0,500	12 686 850	16 714 302	N/A	N/A	100,0	96,9

Lane Background	Lane background subtracted with disk size: 0.1
Lane Width	0.47 mm

Lane 3

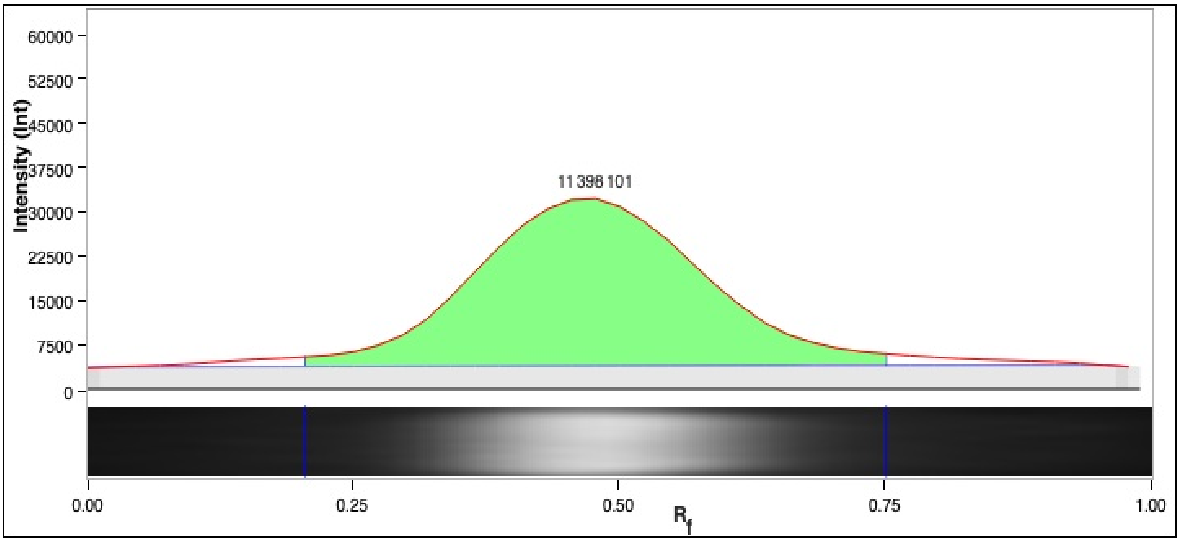

Band No.	Band Label	Mol. Wt. (KDa)	Relative Front	Adj. Volume (Int)	Volume (Int)	Abs. Quant.	Rel. Quant.	Band %	Lane %
1		N/A	0,500	11 398 101	14 882 043	N/A	N/A	100,0	96,1

Lane Background	Lane background subtracted with disk size: 0.1
Lane Width	0.47 mm

Lane 4

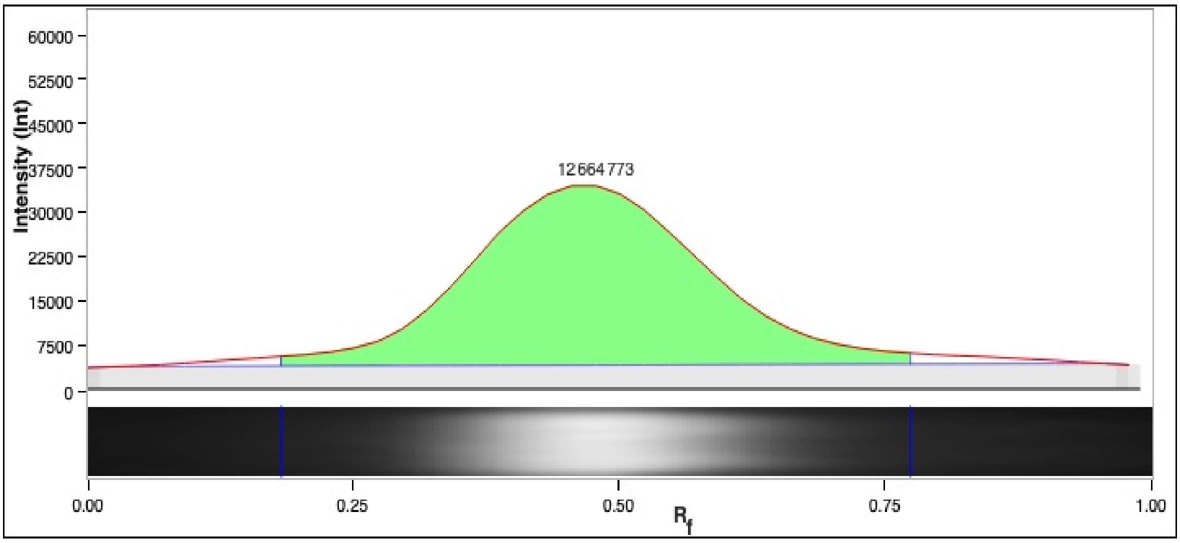

Band No.	Band Label	Mol. Wt. (KDa)	Relative Front	Adj. Volume (Int)	Volume (Int)	Abs. Quant.	Rel. Quant.	Band %	Lane %
1		N/A	0,500	12 664 773	16 617 513	N/A	N/A	100,0	96,9

Lane Background	Lane background subtracted with disk size: 0.1
Lane Width	0.47 mm

Lane 5

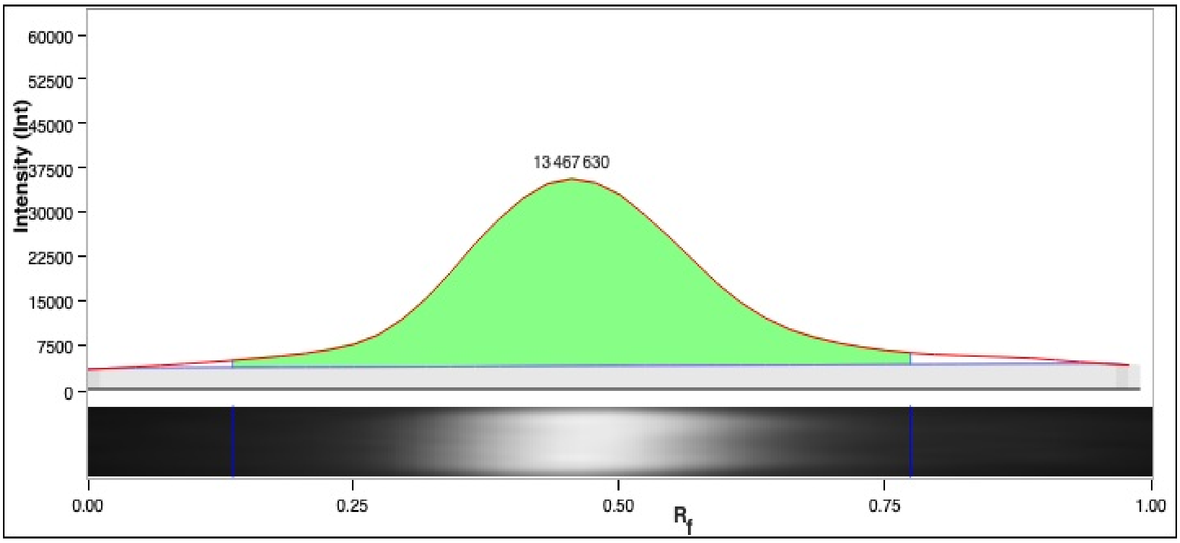

Band No.	Band Label	Mol. Wt. (KDa)	Relative Front	Adj. Volume (Int)	Volume (Int)	Abs. Quant.	Rel. Quant.	Band %	Lane %
1		N/A	0,477	13 467 630	17 533 890	N/A	N/A	100,0	97,4

Lane Background	Lane background subtracted with disk size: 0.1
Lane Width	0.47 mm

Lane 6

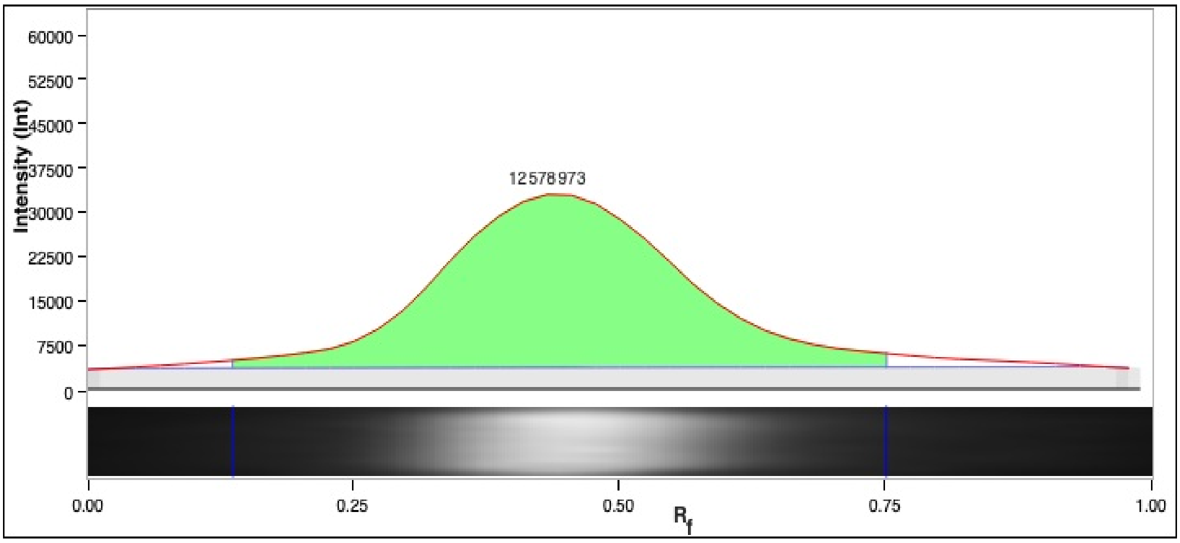

Band No.	Band Label	Mol. Wt. (KDa)	Relative Front	Adj. Volume (Int)	Volume (Int)	Abs. Quant.	Rel. Quant.	Band %	Lane %
1		N/A	0,455	12 578 973	16 241 247	N/A	N/A	100,0	96,9

Lane Background	Lane background subtracted with disk size: 0.1

Lane Width	0.47 mm

Lane 7

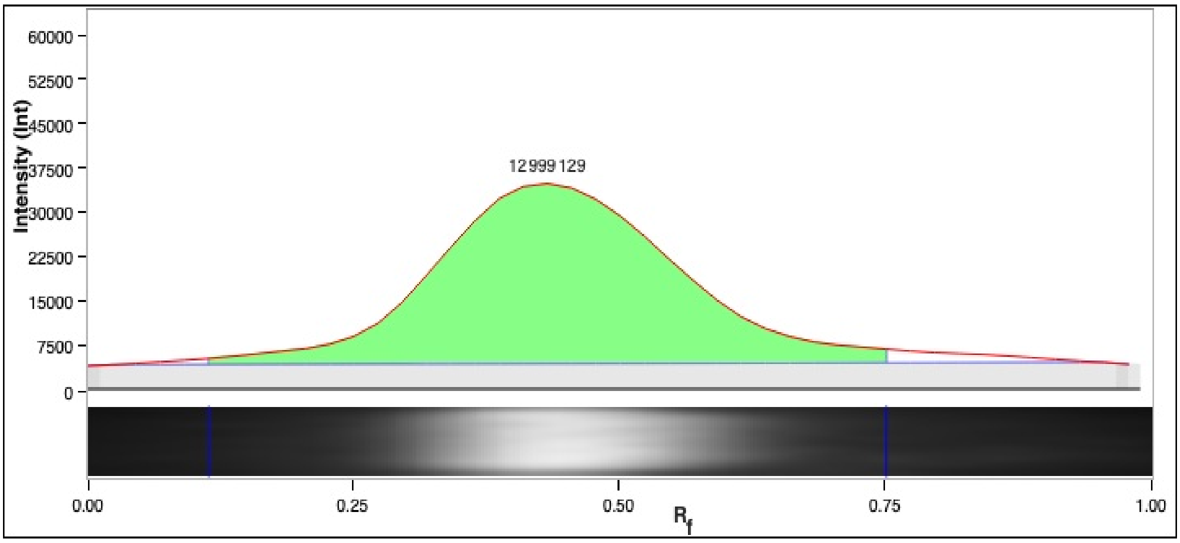

Band No.	Band Label	Mol. Wt. (KDa)	Relative Front	Adj. Volume (Int)	Volume (Int)	Abs. Quant.	Rel. Quant.	Band %	Lane %
1		N/A	0,455	12 999 129	17 492 871	N/A	N/A	100,0	97,1

Lane Background	Lane background subtracted with disk size: 0.1
Lane Width	0.47 mm

Lane 8

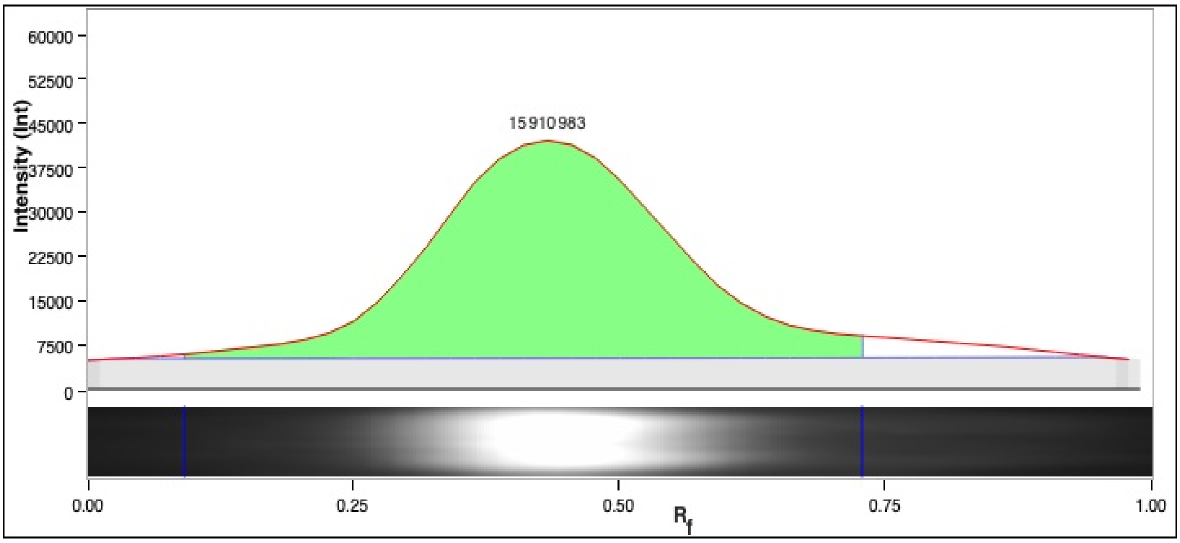

Band No.	Band Label	Mol. Wt. (KDa)	Relative Front	Adj. Volume (Int)	Volume (Int)	Abs. Quant.	Rel. Quant.	Band %	Lane %
1		N/A	0,455	15 910 983	21 548 307	N/A	N/A	100,0	96,6

Lane Background	Lane background subtracted with disk size: 0.1
Lane Width	0.47 mm

Lane 9

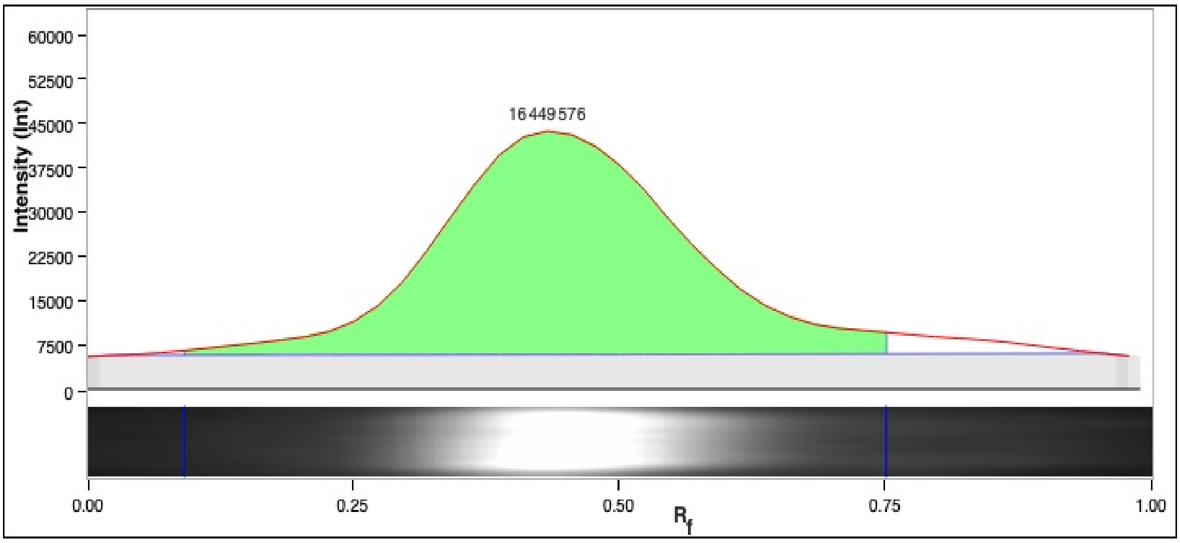

Band No.	Band Label	Mol. Wt. (KDa)	Relative Front	Adj. Volume (Int)	Volume (Int)	Abs. Quant.	Rel. Quant.	Band %	Lane %
1		N/A	0,455	16 449 576	22 986 546	N/A	N/A	100,0	97,2

Lane Background	Lane background subtracted with disk size: 0.1
Lane Width	0.47 mm

Lane 10

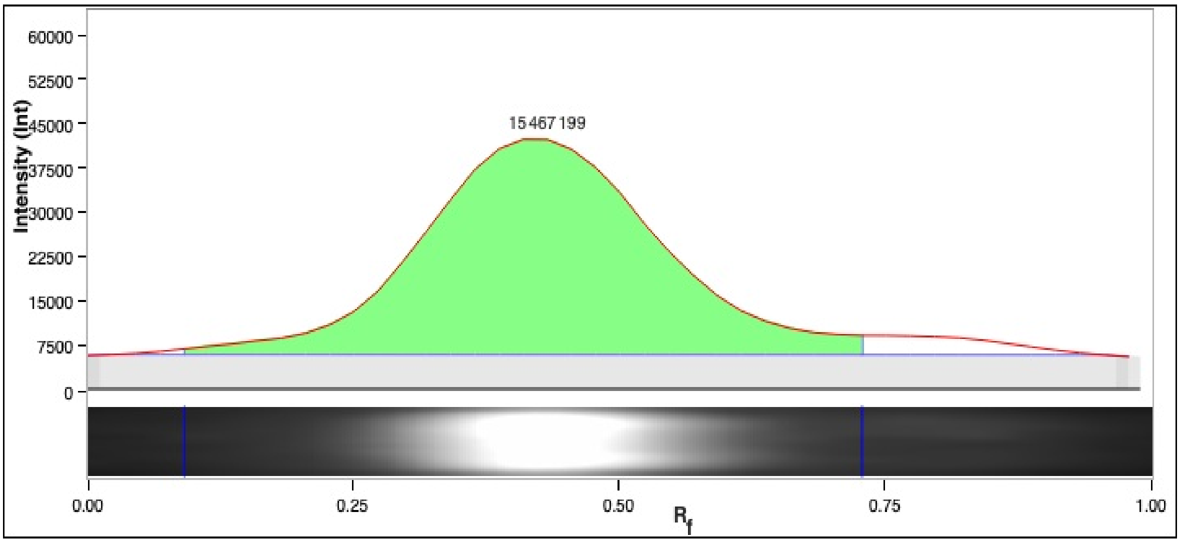

Band No.	Band Label	Mol. Wt. (KDa)	Relative Front	Adj. Volume (Int)	Volume (Int)	Abs. Quant.	Rel. Quant.	Band %	Lane %

1		N/A	0,455	15 467 199	21 770 760	N/A	N/A	100,0	96,2

Lane Background	Lane background subtracted with disk size: 0.1
Lane Width	0.47 mm

Lane 11

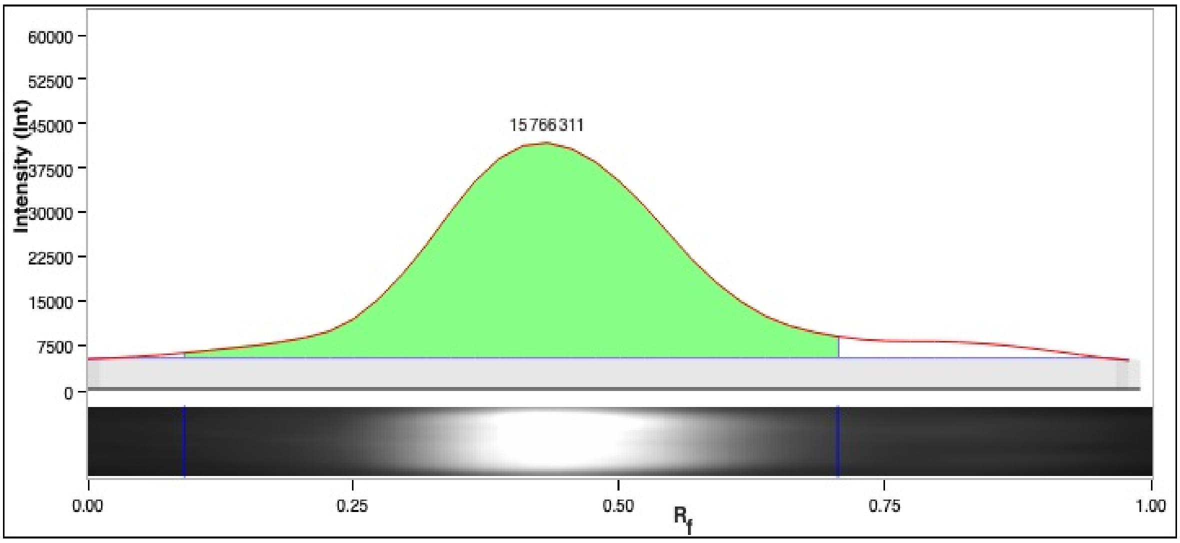

Band No.	Band Label	Mol. Wt. (KDa)	Relative Front	Adj. Volume (Int)	Volume (Int)	Abs. Quant.	Rel. Quant.	Band %	Lane %
1		N/A	0,455	15 766 311	21 277 707	N/A	N/A	100,0	95,7

Lane Background	Lane background subtracted with disk size: 0.1
Lane Width	0.47 mm

Lane 12

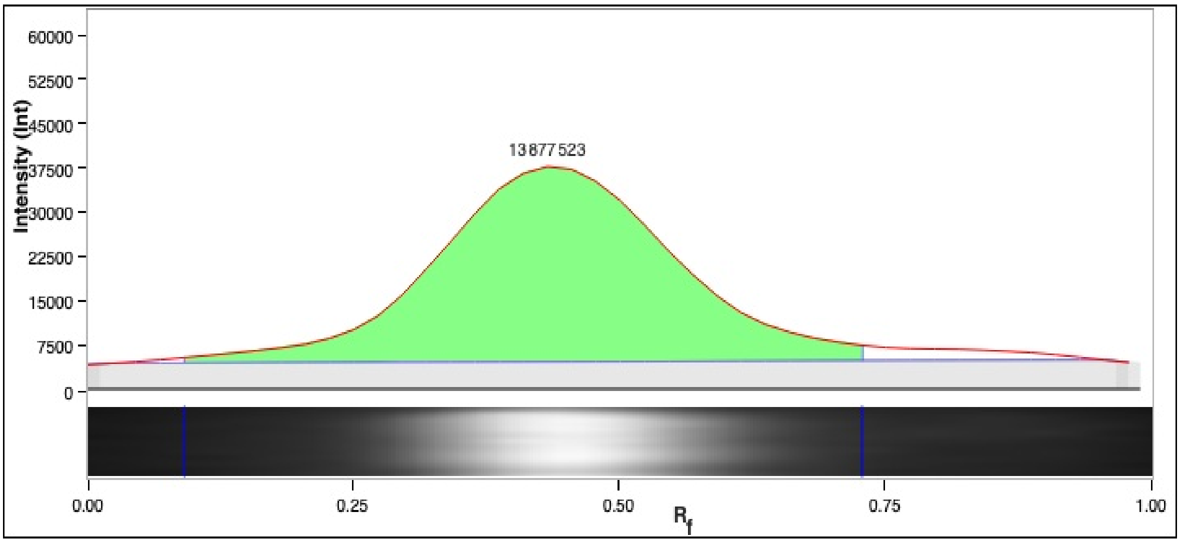

Band No.	Band	Mol. Wt.	Relative	Adj.	Volume	Abs.	Rel.	Band %	Lane %

	Label	(KDa)	Front	Volume (Int)	(Int)	Quant.	Quant.		
1		N/A	0,455	13 877 523	18 701 892	N/A	N/A	100,0	96,3

Lane Background	Lane background subtracted with disk size: 0.1
Lane Width	0.47 mm

Lane 13

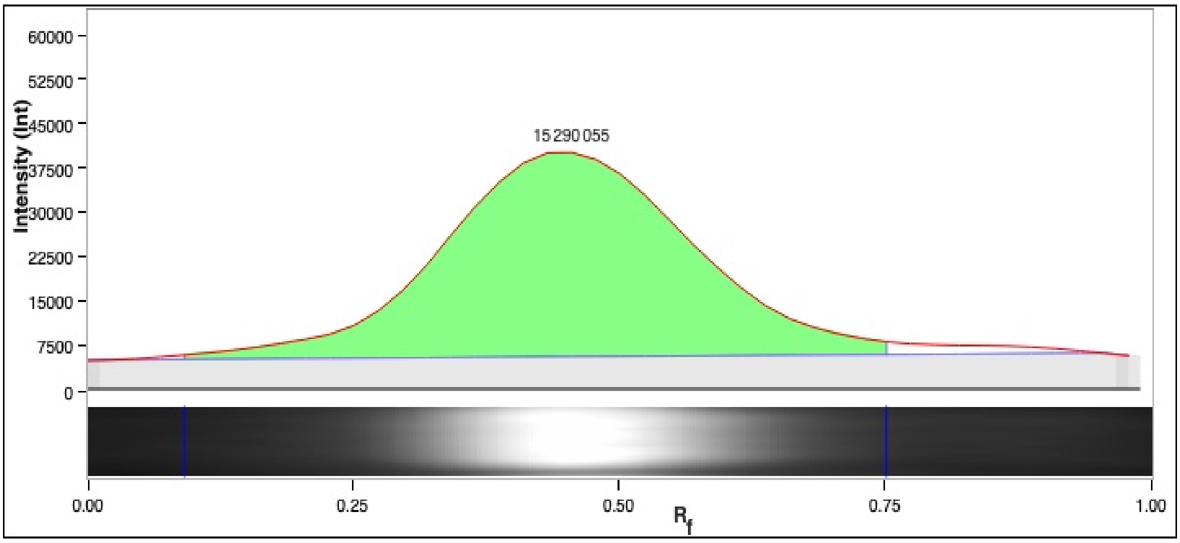

Band No.	Band Label	Mol. Wt. (KDa)	Relative Front	Adj. Volume (Int)	Volume (Int)	Abs. Quant.	Rel. Quant.	Band %	Lane %
1		N/A	0,477	15 290 055	21 166 101	N/A	N/A	100,0	97,5

Lane Background	Lane background subtracted with disk size: 0.1
Lane Width	0.47 mm

Lane 14

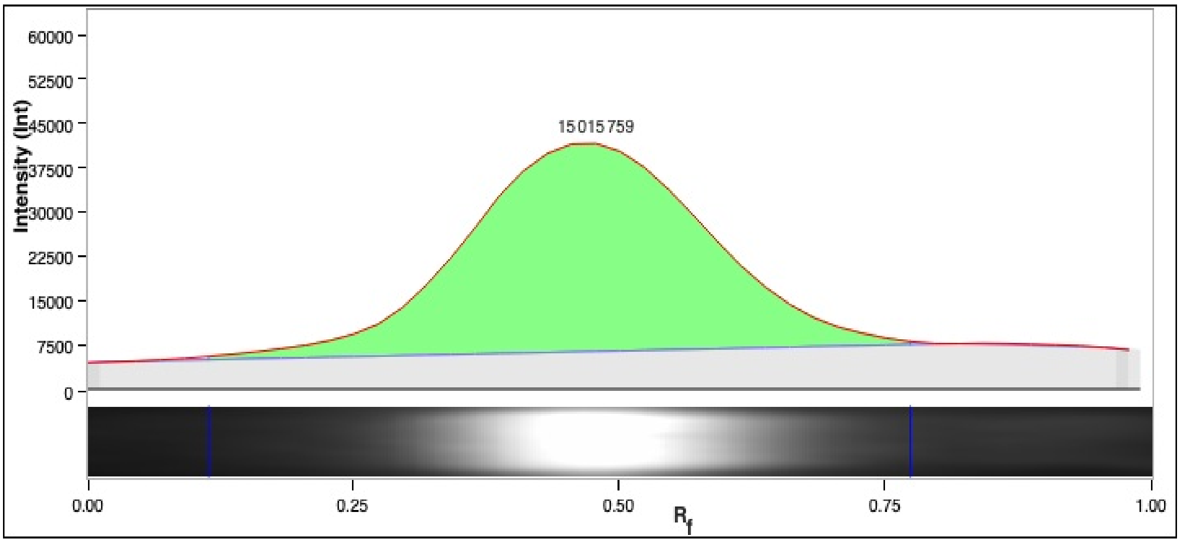

Band No.	Band Label	Mol. Wt. (KDa)	Relative Front	Adj. Volume (Int)	Volume (Int)	Abs. Quant.	Rel. Quant.	Band %	Lane %
1		N/A	0,500	15 015 759	21 774 984	N/A	N/A	100,0	99,6

Lane Background	Lane background subtracted with disk size: 0.1
Lane Width	0.47 mm

Lane 15

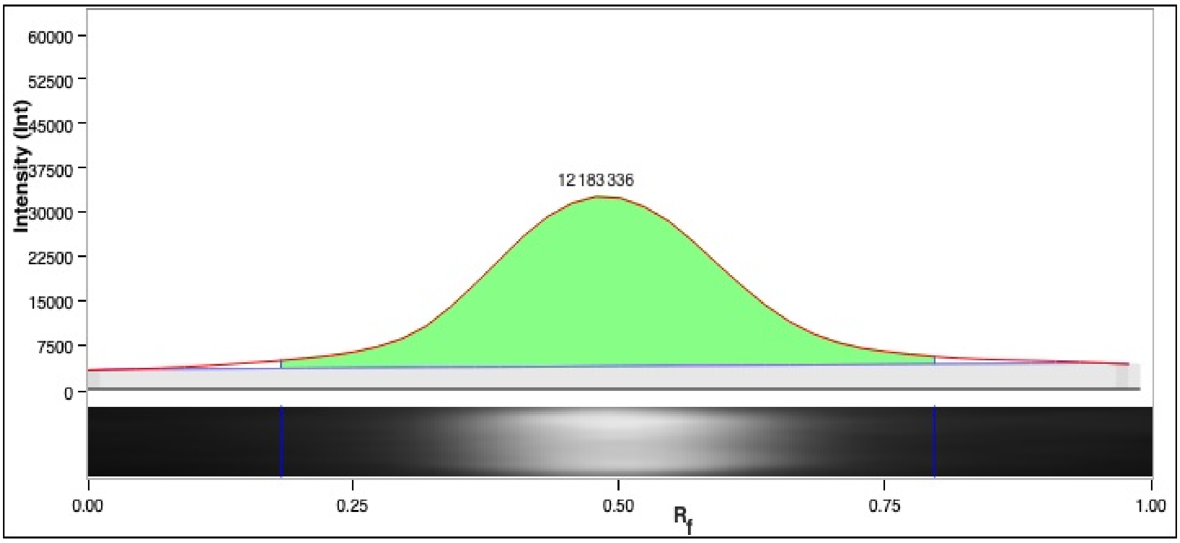

Band No.	Band Label	Mol. Wt. (KDa)	Relative Front	Adj. Volume (Int)	Volume (Int)	Abs. Quant.	Rel. Quant.	Band %	Lane %
1		N/A	0,500	12 183 336	16 074 300	N/A	N/A	100,0	98,1

Lane Background	Lane background subtracted with disk size: 0.1
Lane Width	0.47 mm

Lane 16

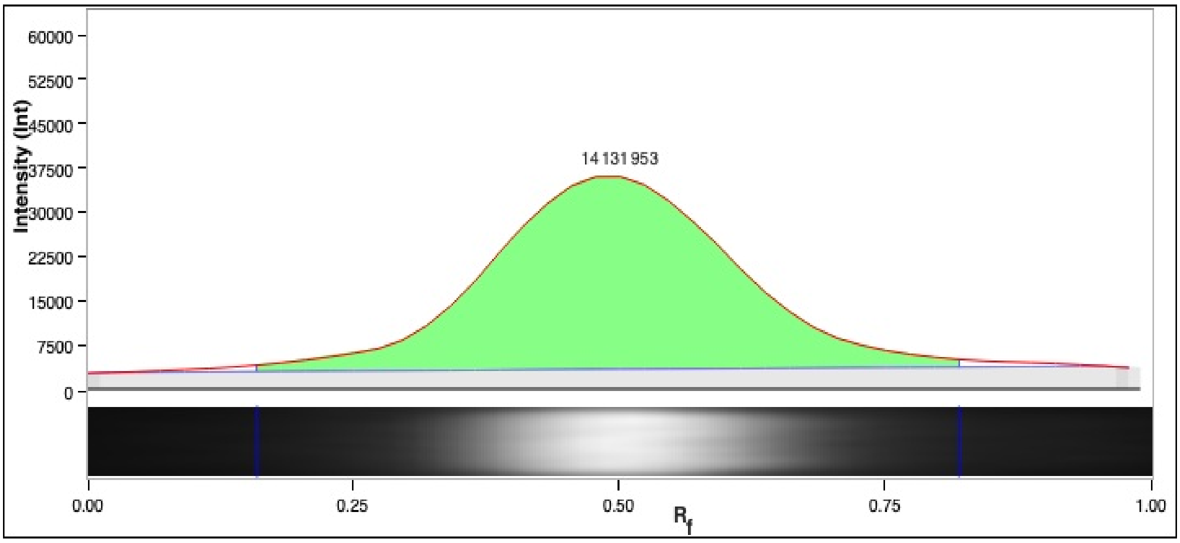

Band No.	Band Label	Mol. Wt. (KDa)	Relative Front	Adj. Volume (Int)	Volume (Int)	Abs. Quant.	Rel. Quant.	Band %	Lane %
1		N/A	0,523	14 131 953	17 732 583	N/A	N/A	100,0	98,6

Lane Background	Lane background subtracted with disk size: 0.1
Lane Width	0.47 mm

Lane 17

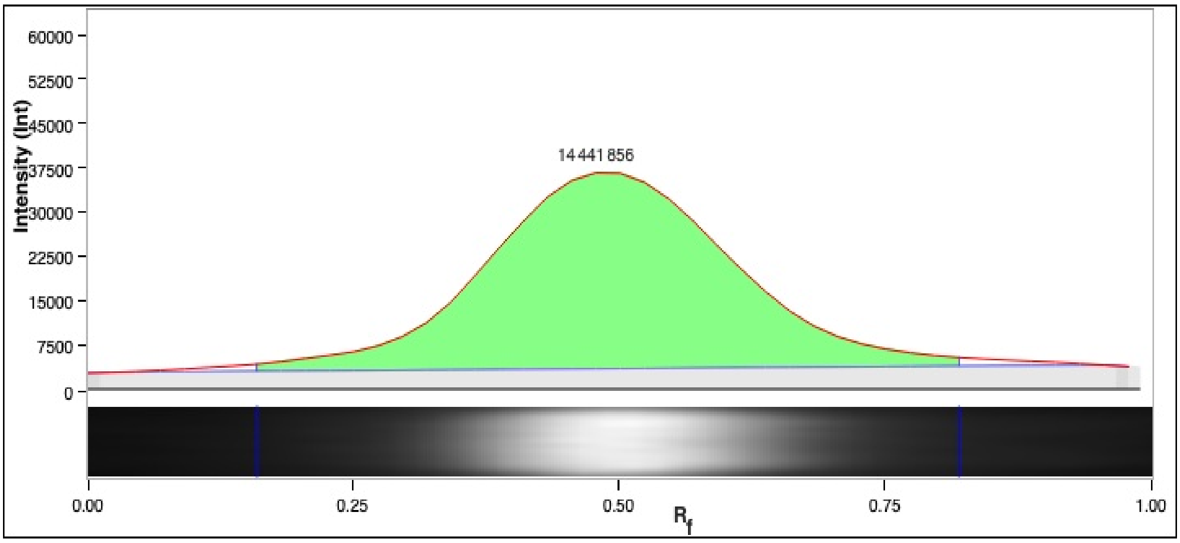

Band No.	Band Label	Mol. Wt. (KDa)	Relative Front	Adj. Volume (Int)	Volume (Int)	Abs. Quant.	Rel. Quant.	Band %	Lane %
1		N/A	0,500	14 441 856	18 174 156	N/A	N/A	100,0	98,5

Lane Background	Lane background subtracted with disk size: 0.1
Lane Width	0.47 mm

Lane 18

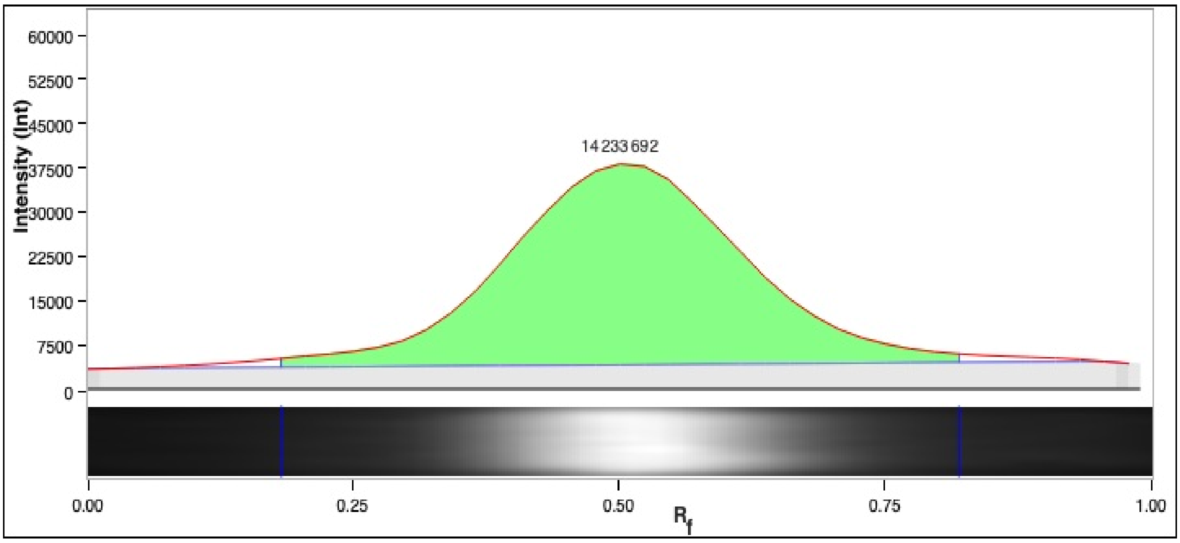

Band No.	Band Label	Mol. Wt. (KDa)	Relative Front	Adj. Volume (Int)	Volume (Int)	Abs. Quant.	Rel. Quant.	Band %	Lane %
1		N/A	0,523	14 233 692	18 521 646	N/A	N/A	100,0	98,2

Lane Background	Lane background subtracted with disk size: 0.1
Lane Width	0.47 mm

Lane 19

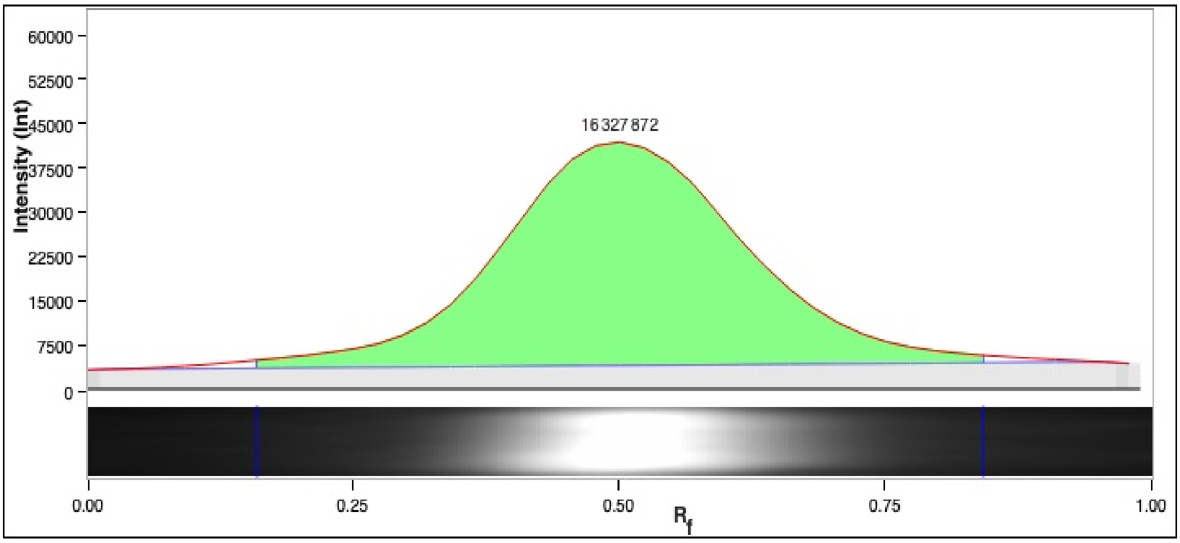

Band No.	Band Label	Mol. Wt. (KDa)	Relative Front	Adj. Volume (Int)	Volume (Int)	Abs. Quant.	Rel. Quant.	Band %	Lane %
1		N/A	0,523	16 327 872	20 779 374	N/A	N/A	100,0	98,9

Lane Background	Lane background subtracted with disk size: 0.1
Lane Width	0.47 mm

Lane 20

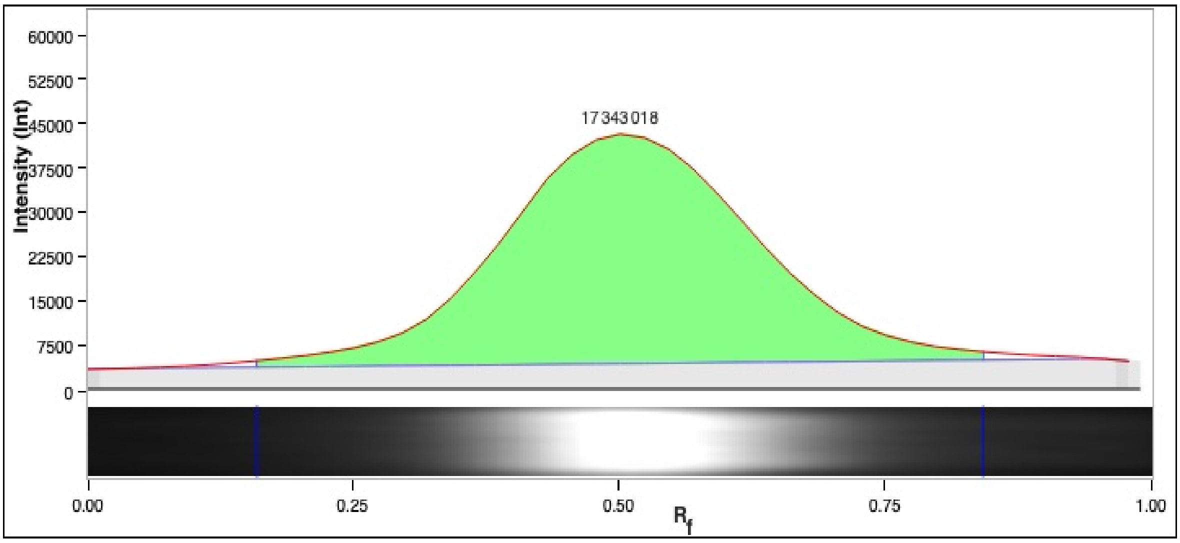

Band No.	Band Label	Mol. Wt. (KDa)	Relative Front	Adj. Volume (Int)	Volume (Int)	Abs. Quant.	Rel. Quant.	Band %	Lane %
1		N/A	0,523	17 343 018	22 153 329	N/A	N/A	100,0	99,0

Lane Background	Lane background subtracted with disk size: 0.1
Lane Width	0.47 mm

Lane 21

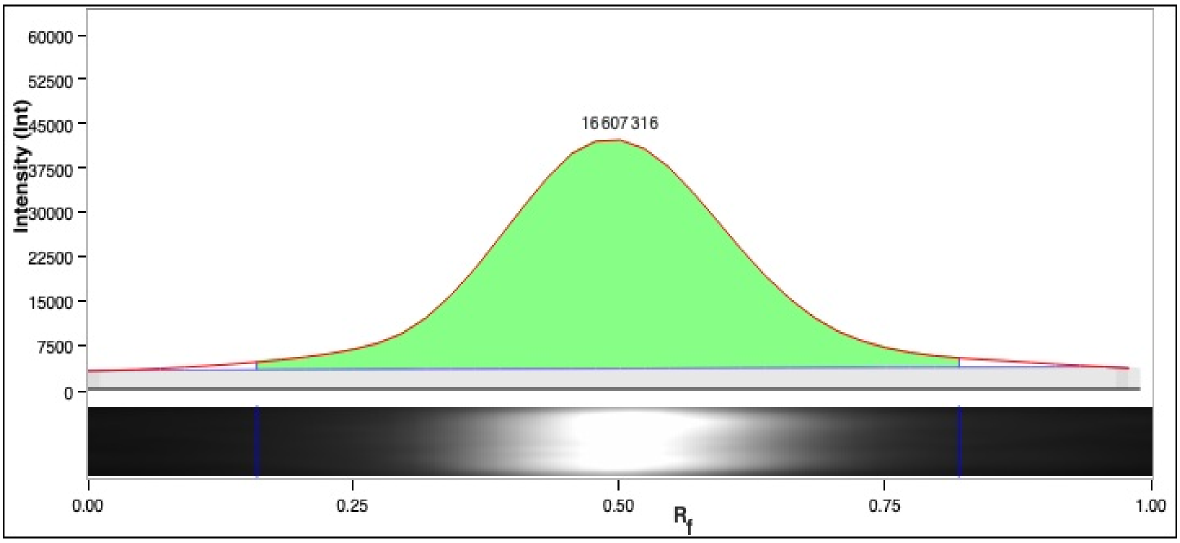

Band No.	Band Label	Mol. Wt. (KDa)	Relative Front	Adj. Volume (Int)	Volume (Int)	Abs. Quant.	Rel. Quant.	Band %	Lane %
1		N/A	0,523	16 607 316	20 349 516	N/A	N/A	100,0	98,6

Lane Background	Lane background subtracted with disk size: 0.1
Lane Width	0.47 mm

Lane 22

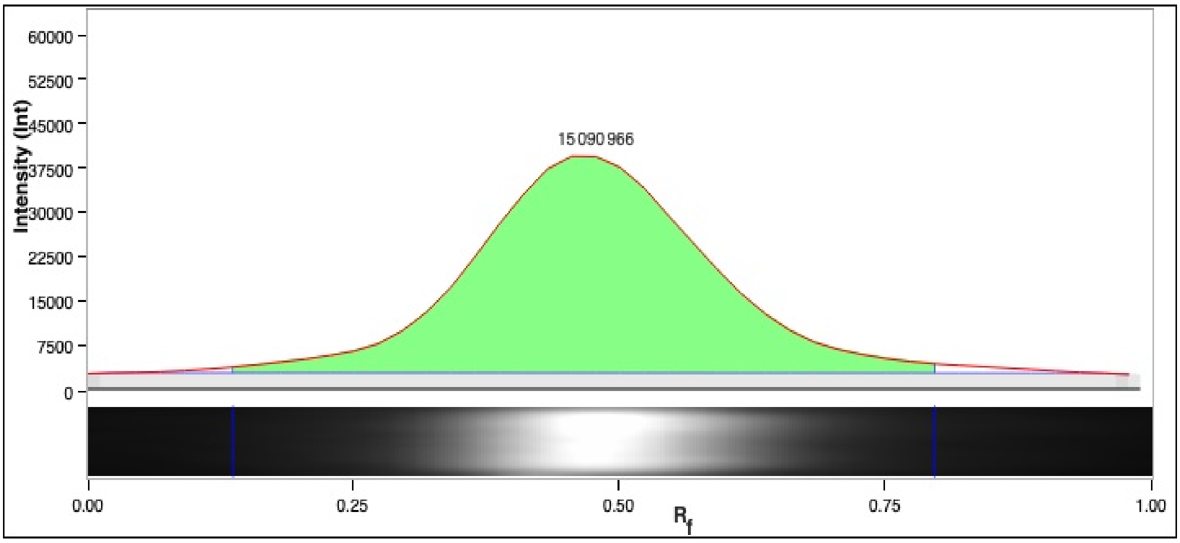

Band No.	Band Label	Mol. Wt. (KDa)	Relative Front	Adj. Volume (Int)	Volume (Int)	Abs. Quant.	Rel. Quant.	Band %	Lane %
1		N/A	0,500	15 090 966	17 965 200	N/A	N/A	100,0	98,5

Lane Background	Lane background subtracted with disk size: 0.1
Lane Width	0.47 mm

Lane 23

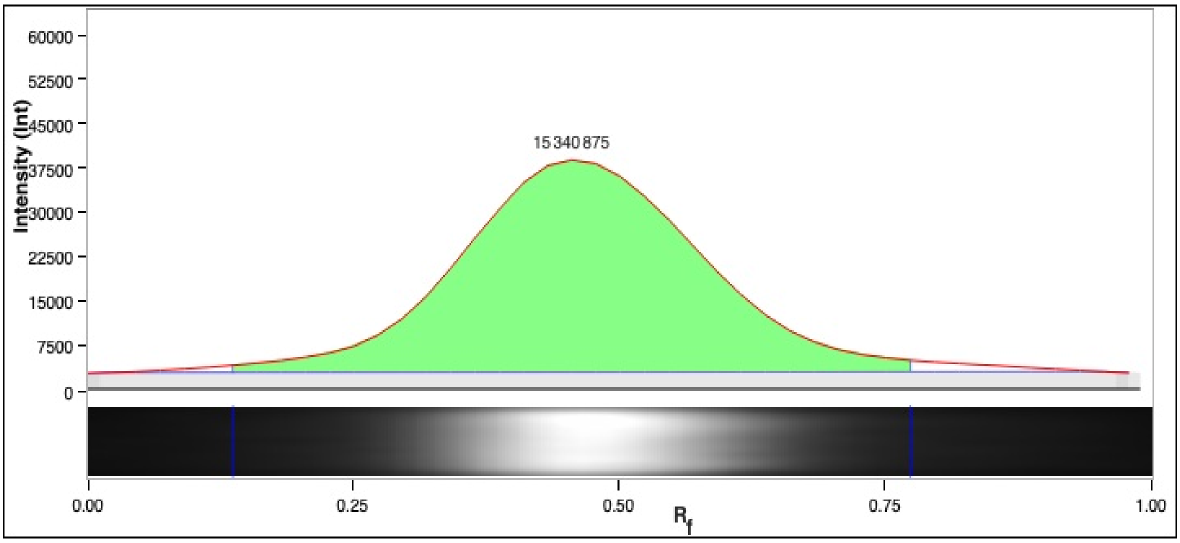

Band No.	Band Label	Mol. Wt. (KDa)	Relative Front	Adj. Volume (Int)	Volume (Int)	Abs. Quant.	Rel. Quant.	Band %	Lane %
1		N/A	0,477	15 340 875	18 310 116	N/A	N/A	100,0	98,0

Lane Background	Lane background subtracted with disk size: 0.1

Lane Width	0.47 mm

Lane 24

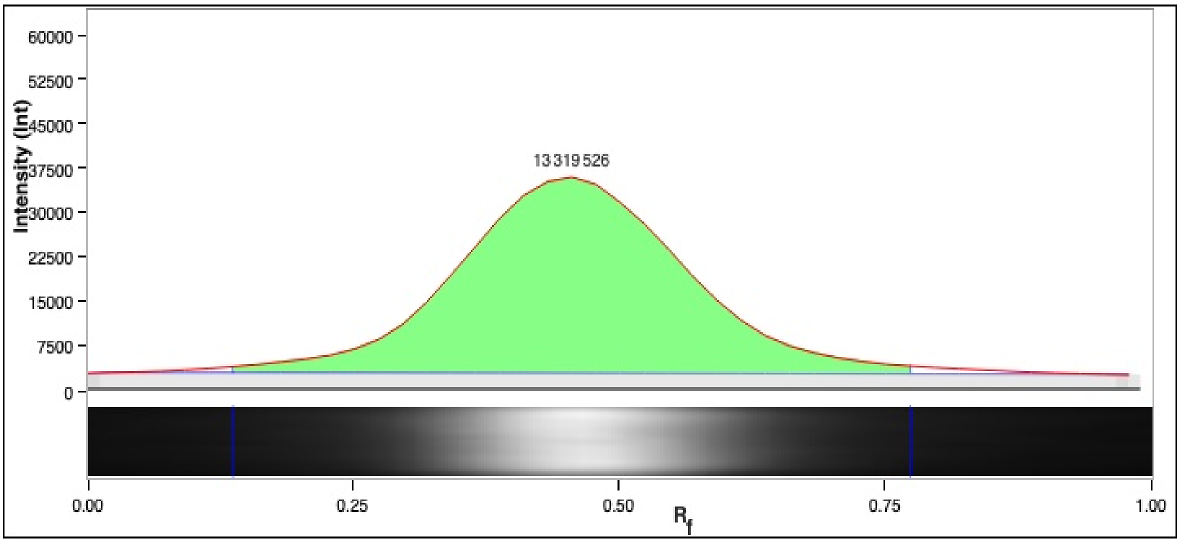

Band No.	Band Label	Mol. Wt. (KDa)	Relative Front	Adj. Volume (Int)	Volume (Int)	Abs. Quant.	Rel. Quant.	Band %	Lane %
1		N/A	0,477	13 319 526	16 077 270	N/A	N/A	100,0	98,5

Lane Background	Lane background subtracted with disk size: 0.1
Lane Width	0.47 mm
