# Supplementary material for: In silico modeling guides identification of novel JAK1 variants associated with immune dysregulation
Source: EMBO Mol Med. 2025 Oct 24;17(12):3275–99. doi: 10.1038/s44321-025-00317-0 (PMC12686074; doi:10.1038/s44321-025-00317-0)
Supplement: Supplementary file 8 — Source data Fig. 3 [file 44321_2025_317_MOESM8_ESM.zip › Figure 3/Replicates Fig.3A/wb 1/20241205 pSTAT1 quantif NS.pdf]

Image Report: IM004002_05Sum-2 (glissées)

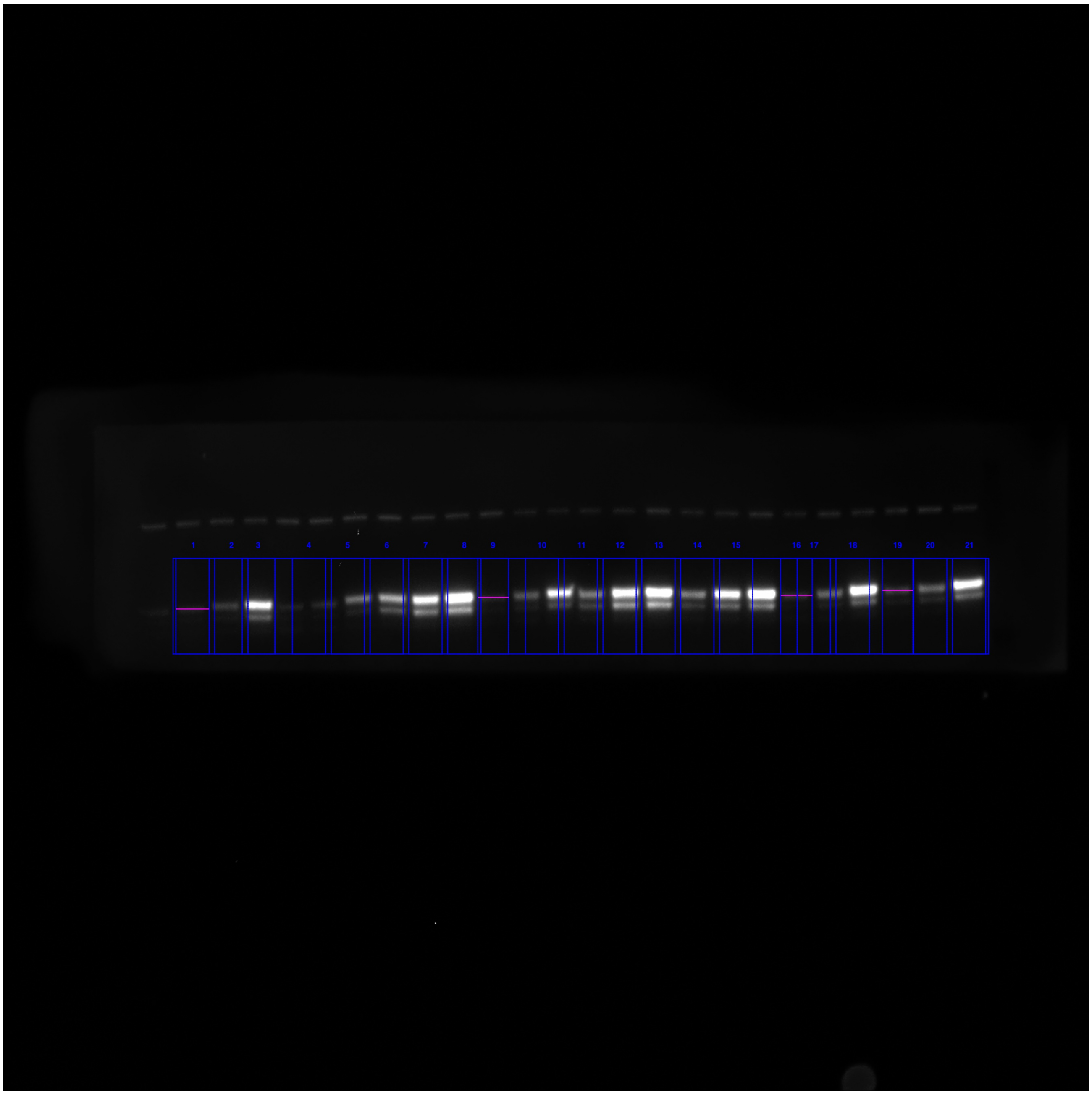

/Users/mariejeanpierre/Desktop/IM004002_05Sum-2 (glissées).tiff

Acquisition Information

Image Information

Acquisition Date	unknown
User Name	Marie Jeanpierre
Image Area (mm)	X: 30.5 Y: 30.5

Pixel Size (µm)	X: 14.1 Y: 14.1
Data Range (Int)	130 - 40363

Analysis Settings

Detection	Lane detection:<br>Manually created lanes<br><br>Band detection:<br><br>Manually adjusted bands<br><br>Lane Background Subtraction:<br>Lane background subtracted with disk size: 0.1<br><br>Lane width: Variable

Lane Statistics

Lane No.	Adj. Total Band Vol. (Int)	Total Band Vol. (Int)	Adj. Total Lane Vol. (Int)	Total Lane Vol. (Int)	Bkgd. Vol. (Int)	Norm. Factor
1	919 578	3 211 560	1 032 372	18 478 218	17 445 846	N/A
2	N/A	N/A	4 362 996	24 063 204	19 700 208	N/A
3	N/A	N/A	17 911 400	47 037 445	29 126 045	N/A
4	N/A	N/A	1 411 542	17 267 184	15 855 642	N/A
5	N/A	N/A	5 068 074	23 257 476	18 189 402	N/A
6	N/A	N/A	10 344 576	35 529 714	25 185 138	N/A
7	N/A	N/A	18 441 258	53 470 098	35 028 840	N/A
8	N/A	N/A	25 660 074	66 593 340	40 933 266	N/A
9	1 402 695	4 038 993	1 569 957	17 228 169	15 658 212	N/A
10	N/A	N/A	10 128 096	32 733 162	22 605 066	N/A
11	N/A	N/A	10 757 010	35 017 554	24 260 544	N/A
12	N/A	N/A	19 982 754	54 543 390	34 560 636	N/A
13	N/A	N/A	25 982 088	66 321 816	40 339 728	N/A
14	N/A	N/A	7 550 400	29 742 570	22 192 170	N/A
15	N/A	N/A	20 264 376	50 684 700	30 420 324	N/A
16	2 069 865	4 875 633	2 373 399	17 409 924	15 036 525	N/A
17	N/A	N/A	3 920 400	20 823 726	16 903 326	N/A
18	N/A	N/A	19 260 780	46 569 732	27 308 952	N/A
19	2 558 645	5 821 718	2 698 152	18 063 564	15 365 412	N/A
20	N/A	N/A	5 821 002	23 991 594	18 170 592	N/A
21	N/A	N/A	20 765 844	50 141 982	29 376 138	N/A

Lane And Band Analysis

Lane 1

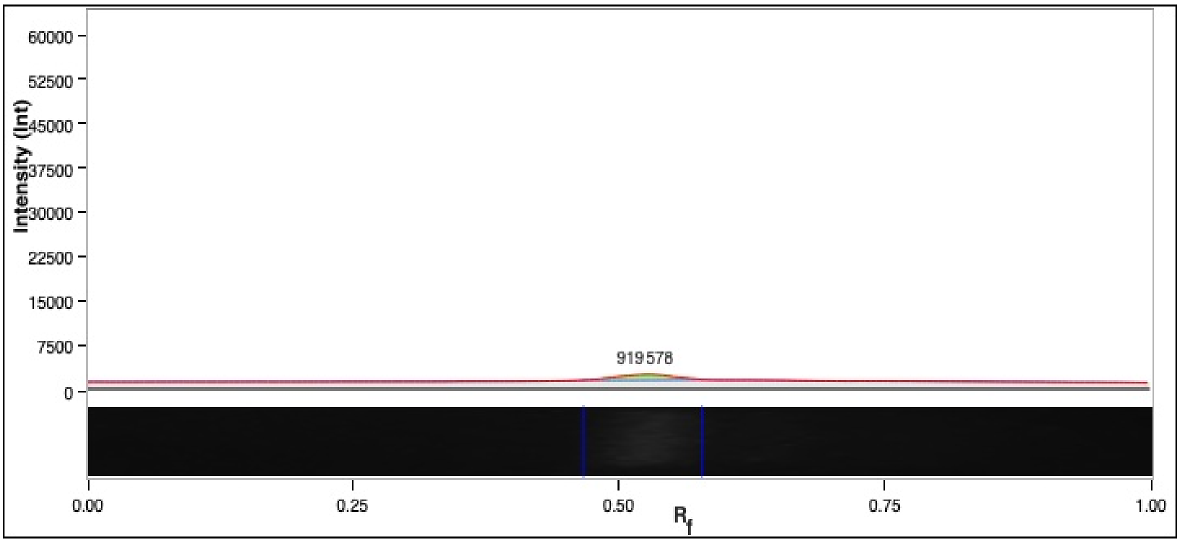

Band No.	Band Label	Mol. Wt. (KDa)	Relative Front	Adj. Volume (Int)	Volume (Int)	Abs. Quant.	Rel. Quant.	Band %	Lane %
1		N/A	0,529	919 578	3 211 560	N/A	N/A	100,0	89,1

Lane Background	Lane background subtracted with disk size: 0.1
Lane Width	0.93 mm

Lane 2

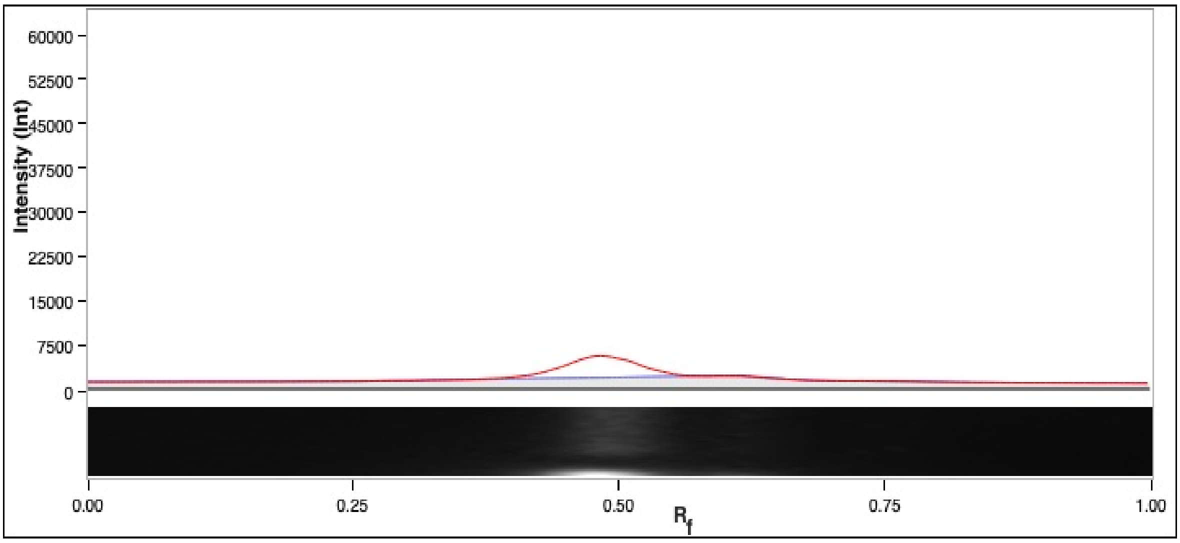

Band No.	Band Label	Mol. Wt. (KDa)	Relative Front	Adj. Volume (Int)	Volume (Int)	Abs. Quant.	Rel. Quant.	Band %	Lane %

Lane Background	Lane background subtracted with disk size: 0.1
Lane Width	0.93 mm

Lane 3

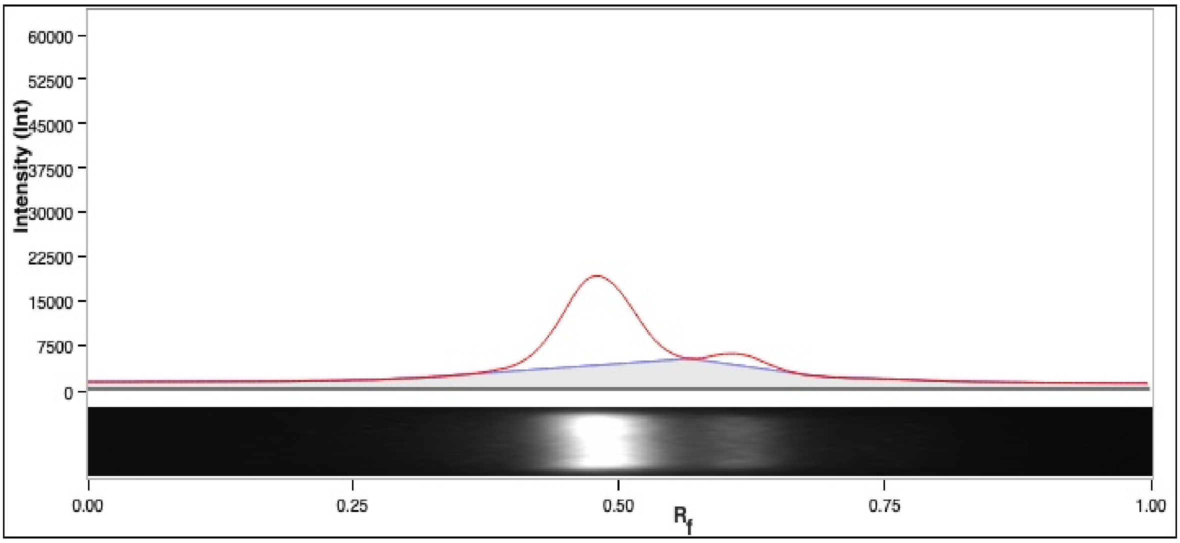

Band No.	Band Label	Mol. Wt. (KDa)	Relative Front	Adj. Volume (Int)	Volume (Int)	Abs. Quant.	Rel. Quant.	Band %	Lane %

Lane Background	Lane background subtracted with disk size: 0.1
Lane Width	0.92 mm

Lane 4

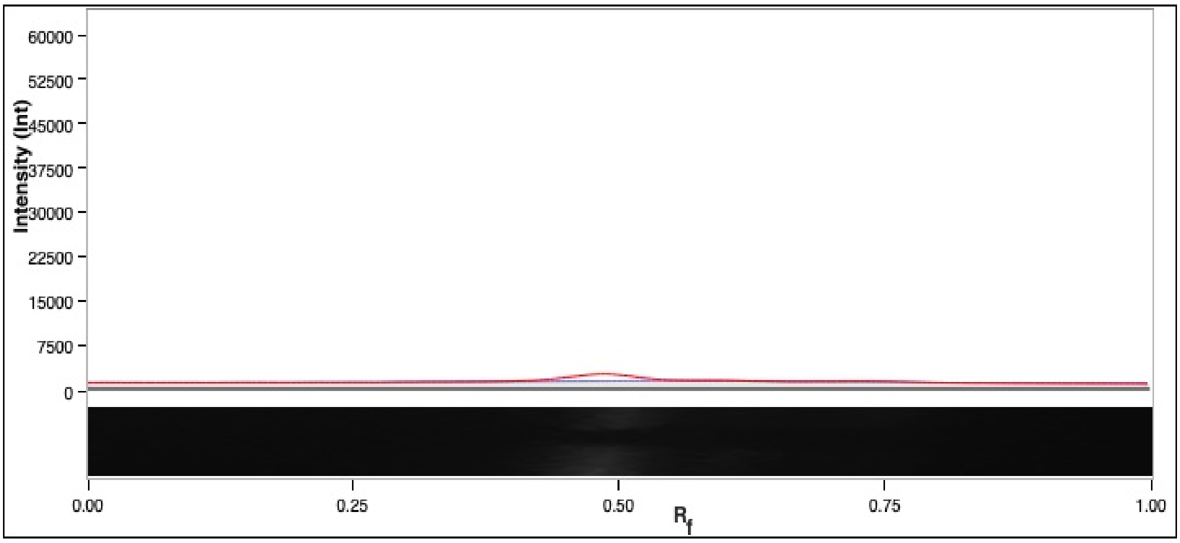

Band No.	Band Label	Mol. Wt. (KDa)	Relative Front	Adj. Volume (Int)	Volume (Int)	Abs. Quant.	Rel. Quant.	Band %	Lane %

Lane Background	Lane background subtracted with disk size: 0.1
Lane Width	0.93 mm

Lane 5

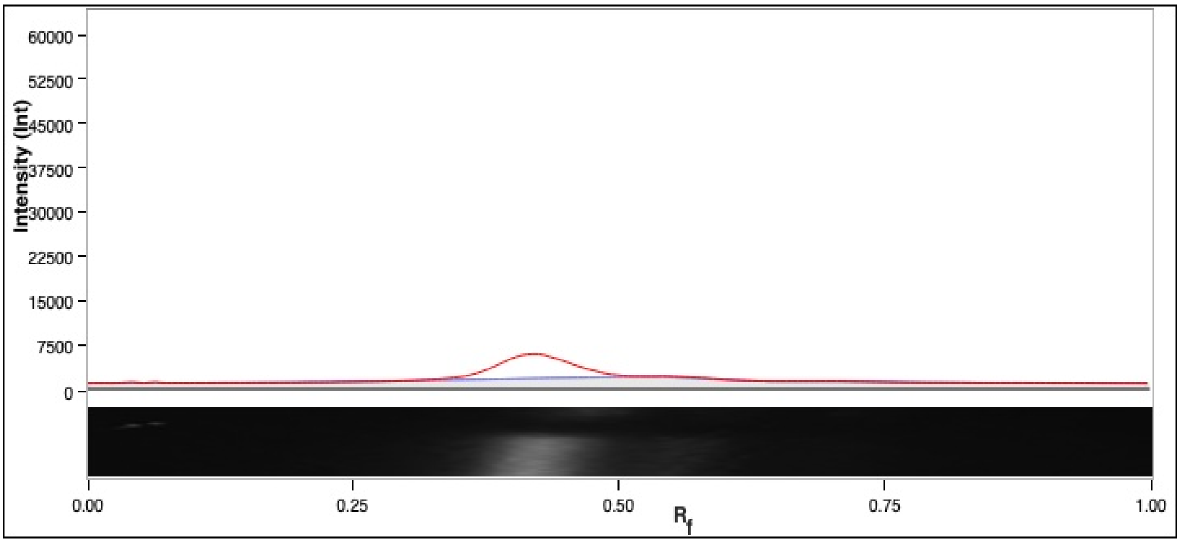

Band No.	Band Label	Mol. Wt. (KDa)	Relative Front	Adj. Volume (Int)	Volume (Int)	Abs. Quant.	Rel. Quant.	Band %	Lane %

Lane Background	Lane background subtracted with disk size: 0.1
Lane Width	0.93 mm

Lane 6

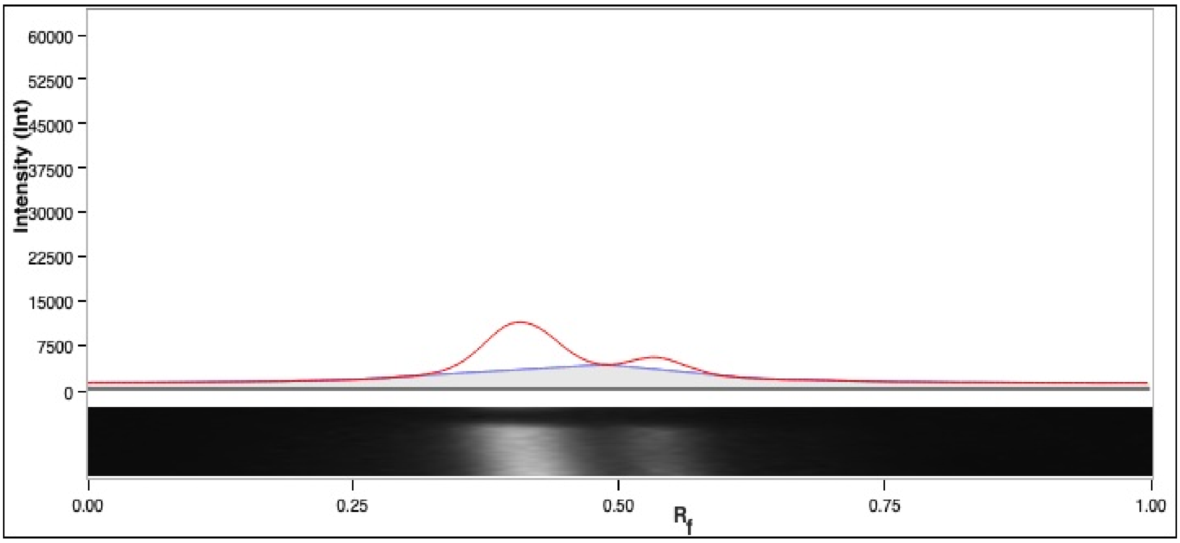

Band No.	Band Label	Mol. Wt. (KDa)	Relative Front	Adj. Volume (Int)	Volume (Int)	Abs. Quant.	Rel. Quant.	Band %	Lane %

Lane Background	Lane background subtracted with disk size: 0.1

Lane Width	0.93 mm

Lane 7

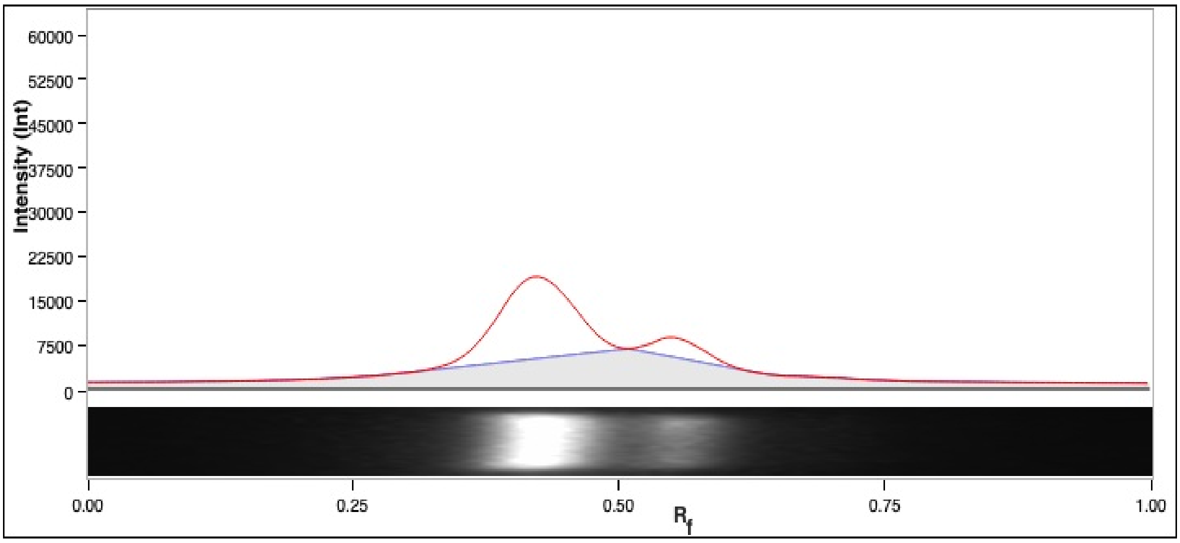

Band No.	Band Label	Mol. Wt. (KDa)	Relative Front	Adj. Volume (Int)	Volume (Int)	Abs. Quant.	Rel. Quant.	Band %	Lane %

Lane Background	Lane background subtracted with disk size: 0.1
Lane Width	0.93 mm

Lane 8

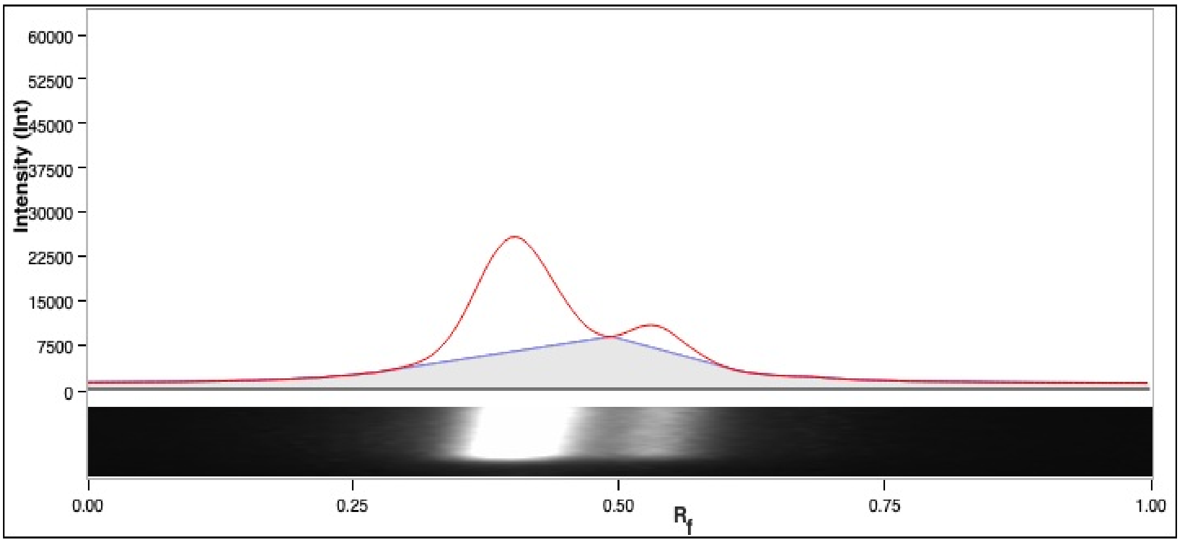

Band No.	Band Label	Mol. Wt. (KDa)	Relative Front	Adj. Volume (Int)	Volume (Int)	Abs. Quant.	Rel. Quant.	Band %	Lane %

Lane Background	Lane background subtracted with disk size: 0.1
Lane Width	0.93 mm

Lane 9

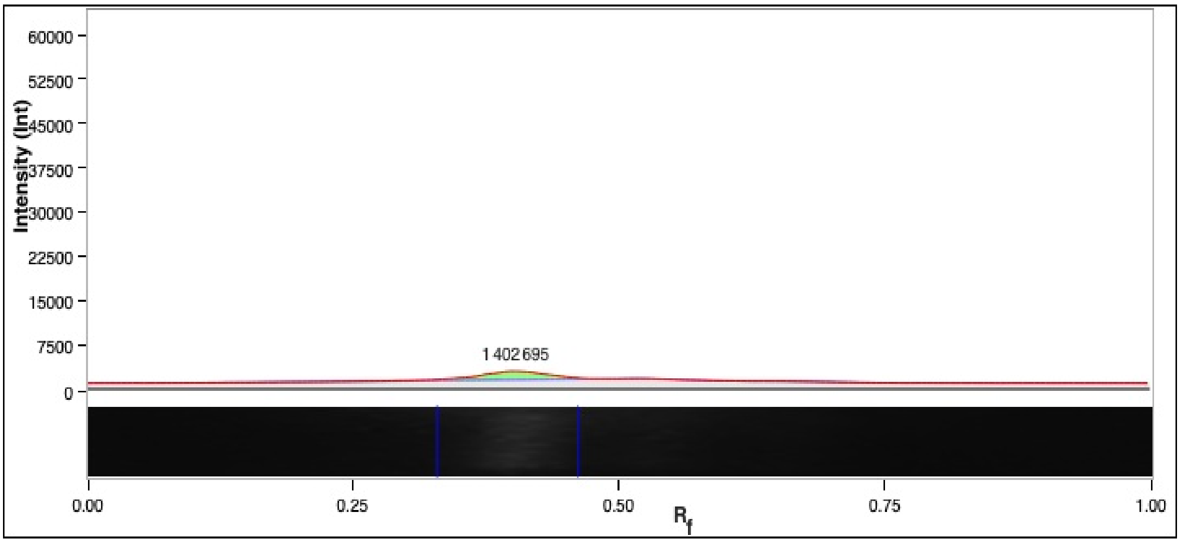

Band No.	Band Label	Mol. Wt. (KDa)	Relative Front	Adj. Volume (Int)	Volume (Int)	Abs. Quant.	Rel. Quant.	Band %	Lane %
1		N/A	0,407	1 402 695	4 038 993	N/A	N/A	100,0	89,3

Lane Background	Lane background subtracted with disk size: 0.1
Lane Width	0.86 mm

Lane 10

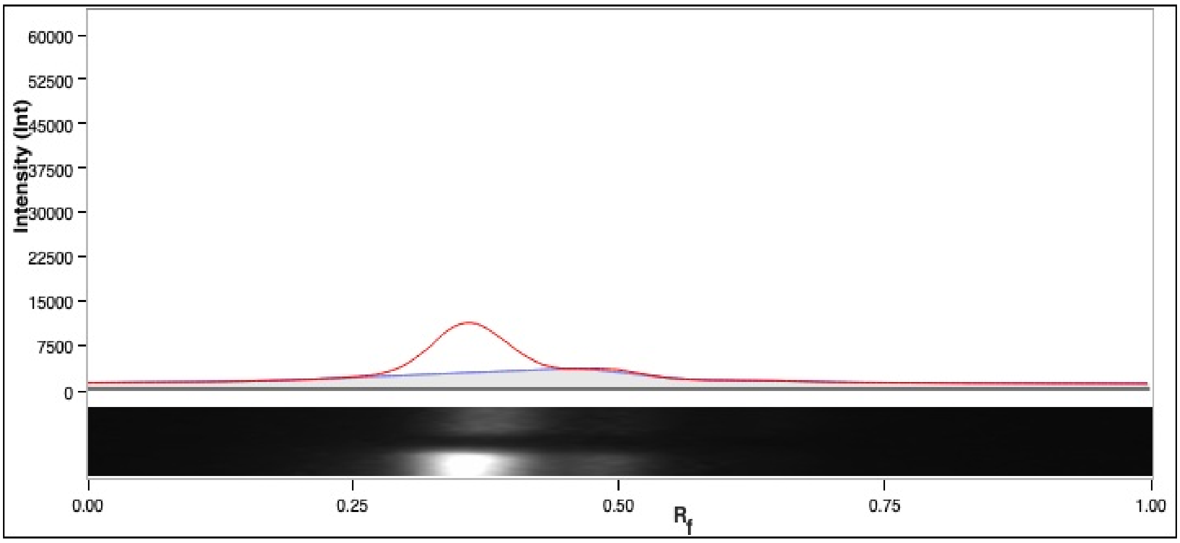

Band No.	Band Label	Mol. Wt. (KDa)	Relative Front	Adj. Volume (Int)	Volume (Int)	Abs. Quant.	Rel. Quant.	Band %	Lane %

Lane Background	Lane background subtracted with disk size: 0.1
Lane Width	0.93 mm

Lane 11

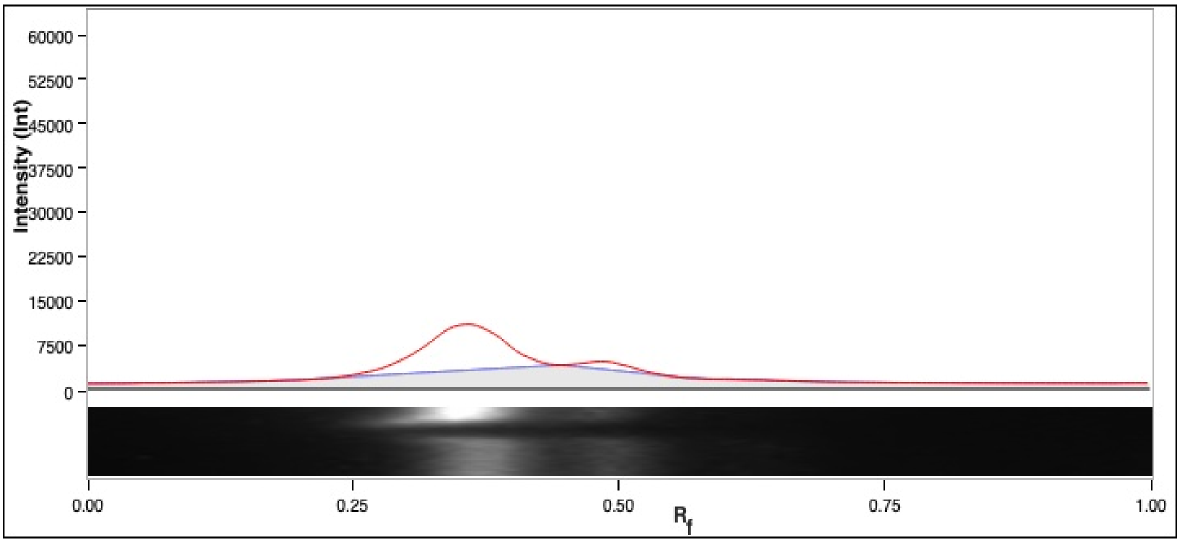

Band No.	Band Label	Mol. Wt. (KDa)	Relative Front	Adj. Volume (Int)	Volume (Int)	Abs. Quant.	Rel. Quant.	Band %	Lane %

Lane Background	Lane background subtracted with disk size: 0.1
Lane Width	0.93 mm

Lane 12

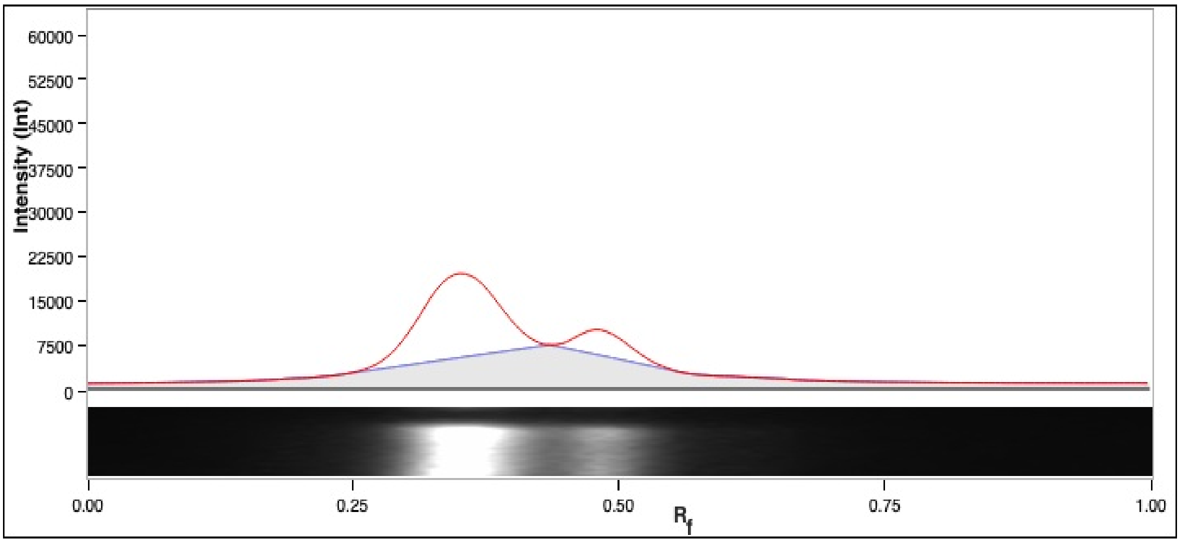

Band No.	Band	Mol. Wt.	Relative	Adj.	Volume	Abs.	Rel.	Band %	Lane %

	Label	(KDa)	Front	Volume (Int)	(Int)	Quant.	Quant.		

Lane Background	Lane background subtracted with disk size: 0.1
Lane Width	0.93 mm

Lane 13

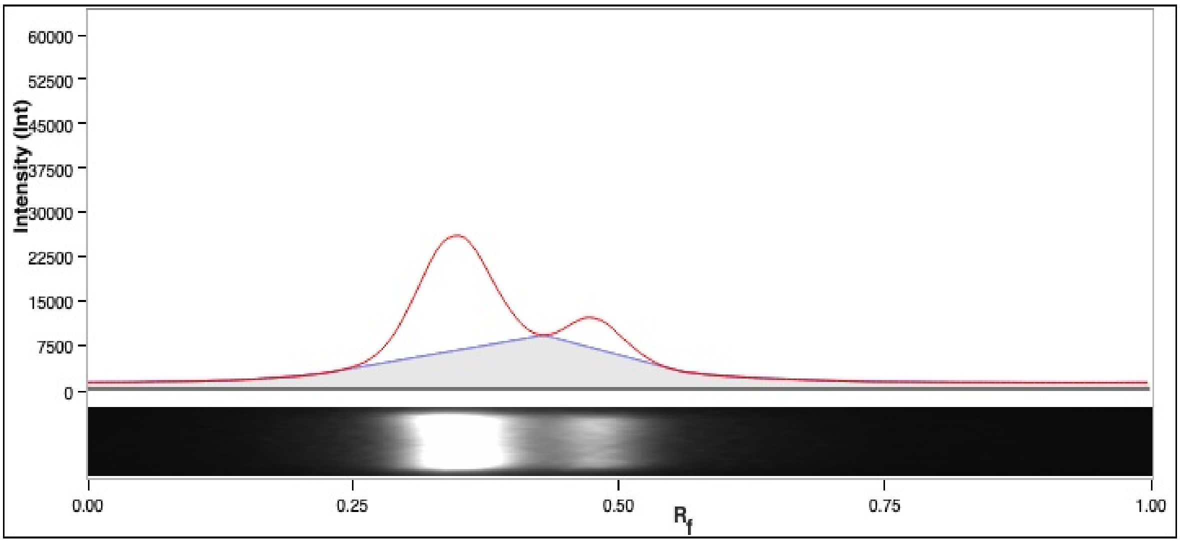

Band No.	Band Label	Mol. Wt. (KDa)	Relative Front	Adj. Volume (Int)	Volume (Int)	Abs. Quant.	Rel. Quant.	Band %	Lane %

Lane Background	Lane background subtracted with disk size: 0.1
Lane Width	0.93 mm

Lane 14

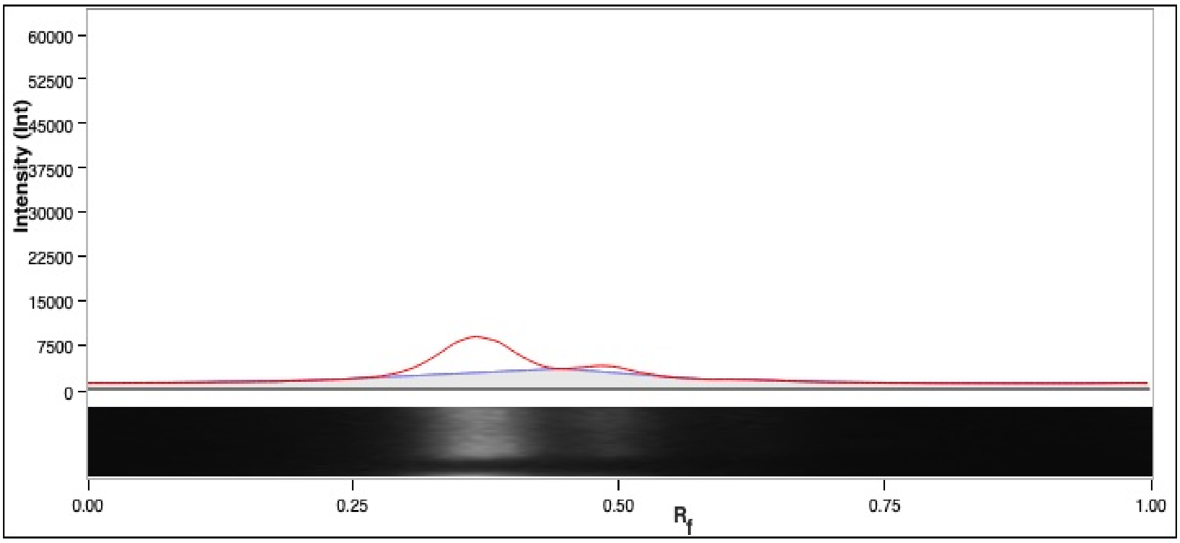

Band No.	Band Label	Mol. Wt. (KDa)	Relative Front	Adj. Volume (Int)	Volume (Int)	Abs. Quant.	Rel. Quant.	Band %	Lane %

Lane Background	Lane background subtracted with disk size: 0.1
Lane Width	0.93 mm

Lane 15

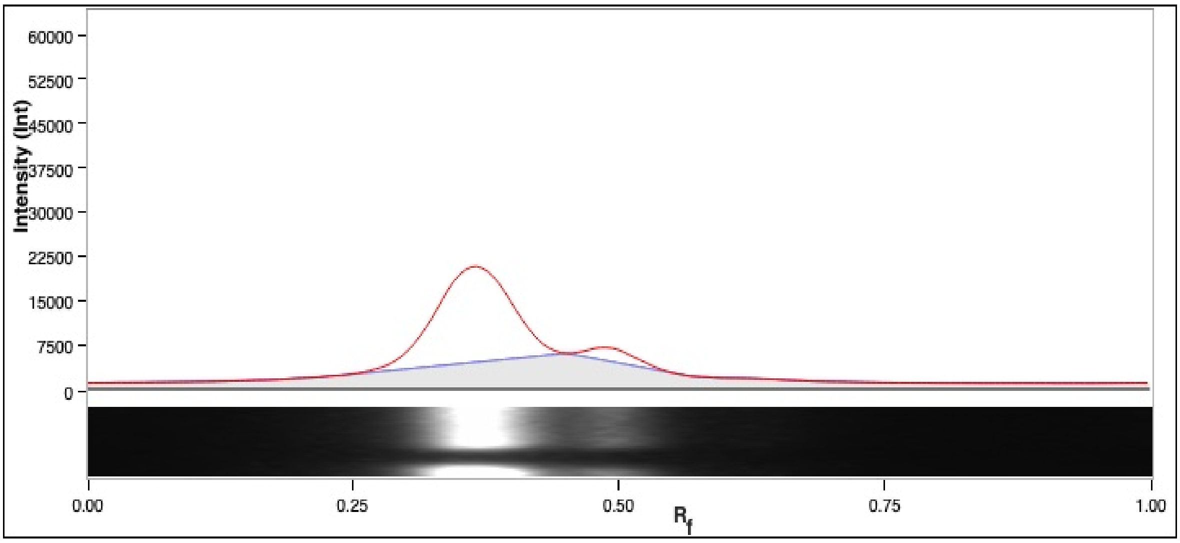

Band No.	Band Label	Mol. Wt. (KDa)	Relative Front	Adj. Volume (Int)	Volume (Int)	Abs. Quant.	Rel. Quant.	Band %	Lane %

Lane Background	Lane background subtracted with disk size: 0.1
Lane Width	0.93 mm

Lane 16

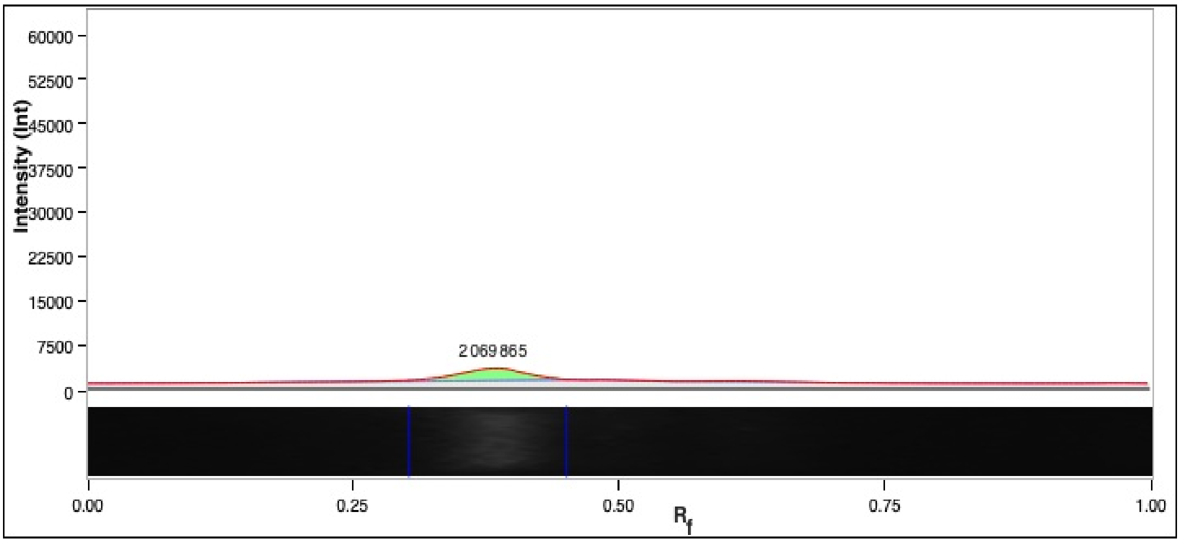

Band No.	Band Label	Mol. Wt. (KDa)	Relative Front	Adj. Volume (Int)	Volume (Int)	Abs. Quant.	Rel. Quant.	Band %	Lane %
1		N/A	0,386	2 069 865	4 875 633	N/A	N/A	100,0	87,2

Lane Background	Lane background subtracted with disk size: 0.1
Lane Width	0.89 mm

Lane 17

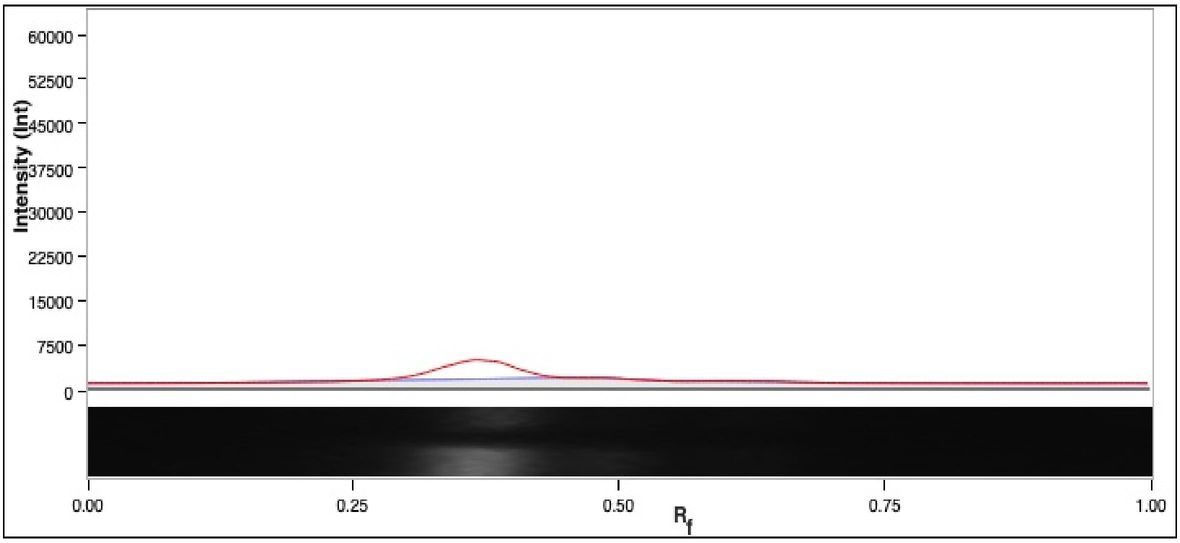

Band No.	Band Label	Mol. Wt. (KDa)	Relative Front	Adj. Volume (Int)	Volume (Int)	Abs. Quant.	Rel. Quant.	Band %	Lane %

Lane Background	Lane background subtracted with disk size: 0.1
Lane Width	0.93 mm

Lane 18

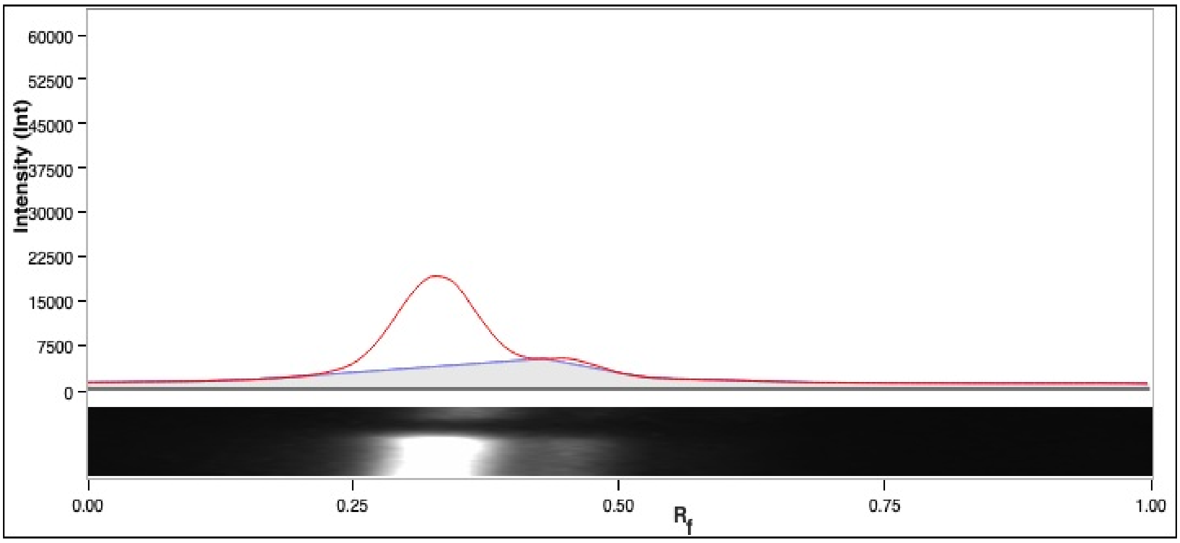

Band No.	Band Label	Mol. Wt. (KDa)	Relative Front	Adj. Volume (Int)	Volume (Int)	Abs. Quant.	Rel. Quant.	Band %	Lane %

Lane Background	Lane background subtracted with disk size: 0.1
Lane Width	0.93 mm

Lane 19

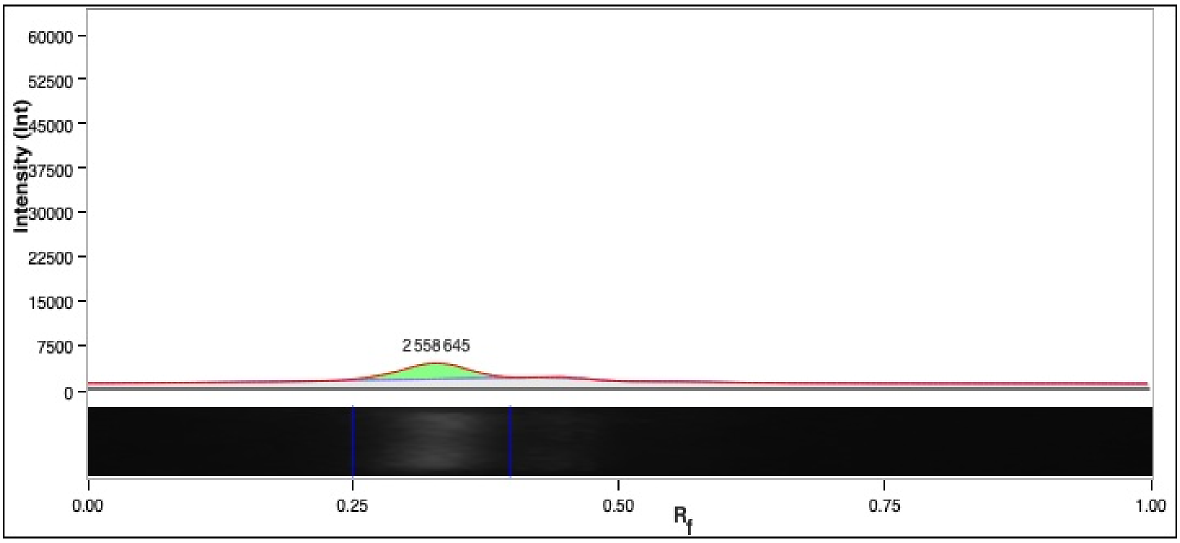

Band No.	Band Label	Mol. Wt. (KDa)	Relative Front	Adj. Volume (Int)	Volume (Int)	Abs. Quant.	Rel. Quant.	Band %	Lane %
1		N/A	0,333	2 558 645	5 821 718	N/A	N/A	100,0	94,8

Lane Background	Lane background subtracted with disk size: 0.1
Lane Width	0.86 mm

Lane 20

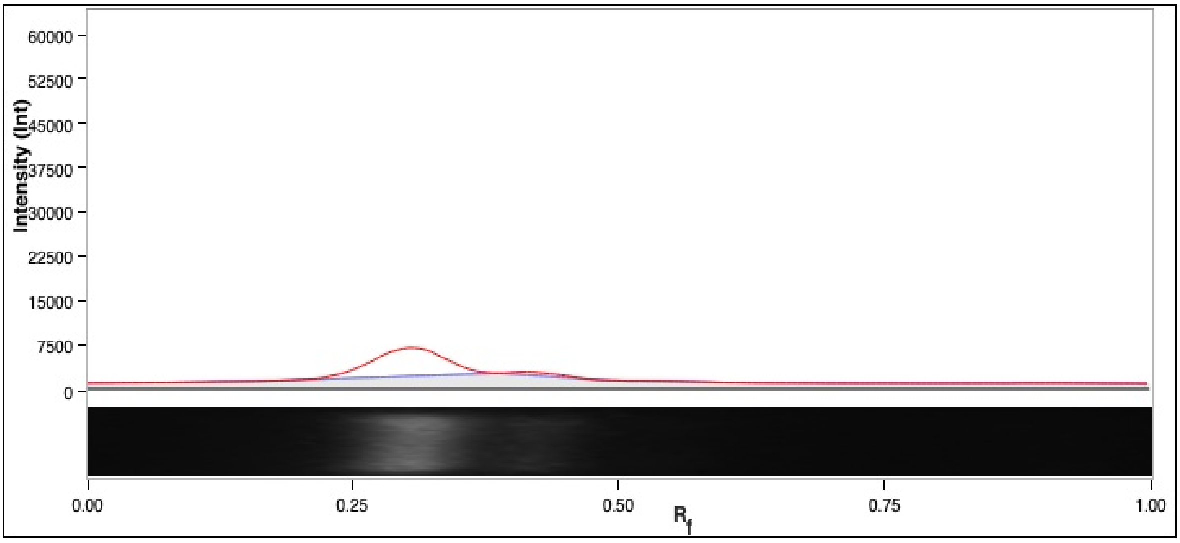

Band No.	Band Label	Mol. Wt. (KDa)	Relative Front	Adj. Volume (Int)	Volume (Int)	Abs. Quant.	Rel. Quant.	Band %	Lane %

Lane Background	Lane background subtracted with disk size: 0.1
Lane Width	0.93 mm

Lane 21

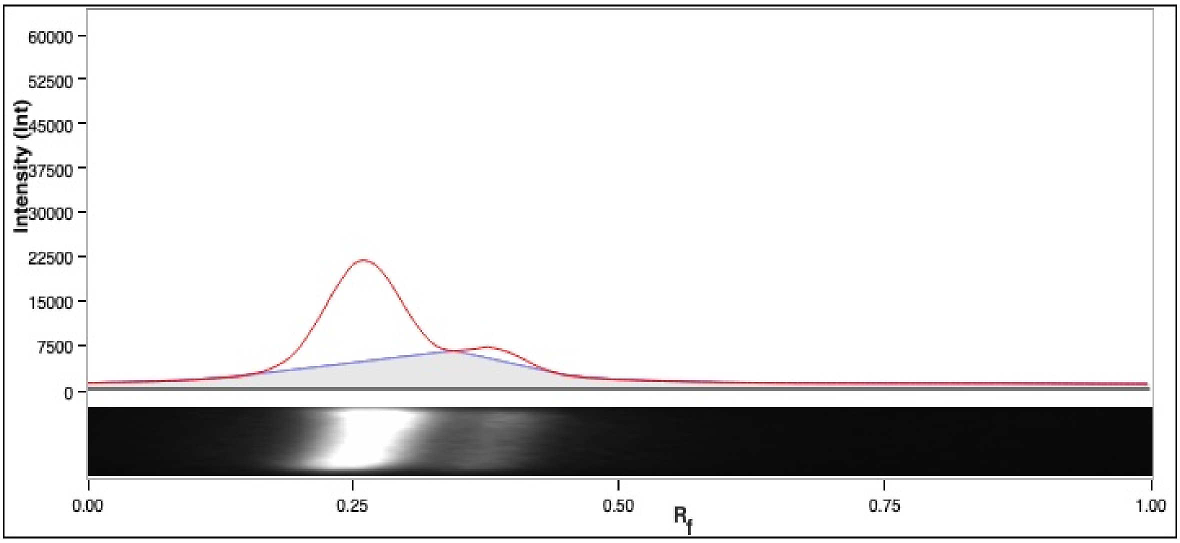

Band No.	Band Label	Mol. Wt. (KDa)	Relative Front	Adj. Volume (Int)	Volume (Int)	Abs. Quant.	Rel. Quant.	Band %	Lane %

Lane Background	Lane background subtracted with disk size: 0.1
Lane Width	0.93 mm
