# Supplementary material for: In silico modeling guides identification of novel JAK1 variants associated with immune dysregulation
Source: EMBO Mol Med. 2025 Oct 24;17(12):3275–99. doi: 10.1038/s44321-025-00317-0 (PMC12686074; doi:10.1038/s44321-025-00317-0)
Supplement: Supplementary file 8 — Source data Fig. 3 [file 44321_2025_317_MOESM8_ESM.zip › Figure 3/Replicates Fig.3A/wb 5/pSTAT1 true quantif .pdf]

Image Report: IM004419_03-2 (glissées)

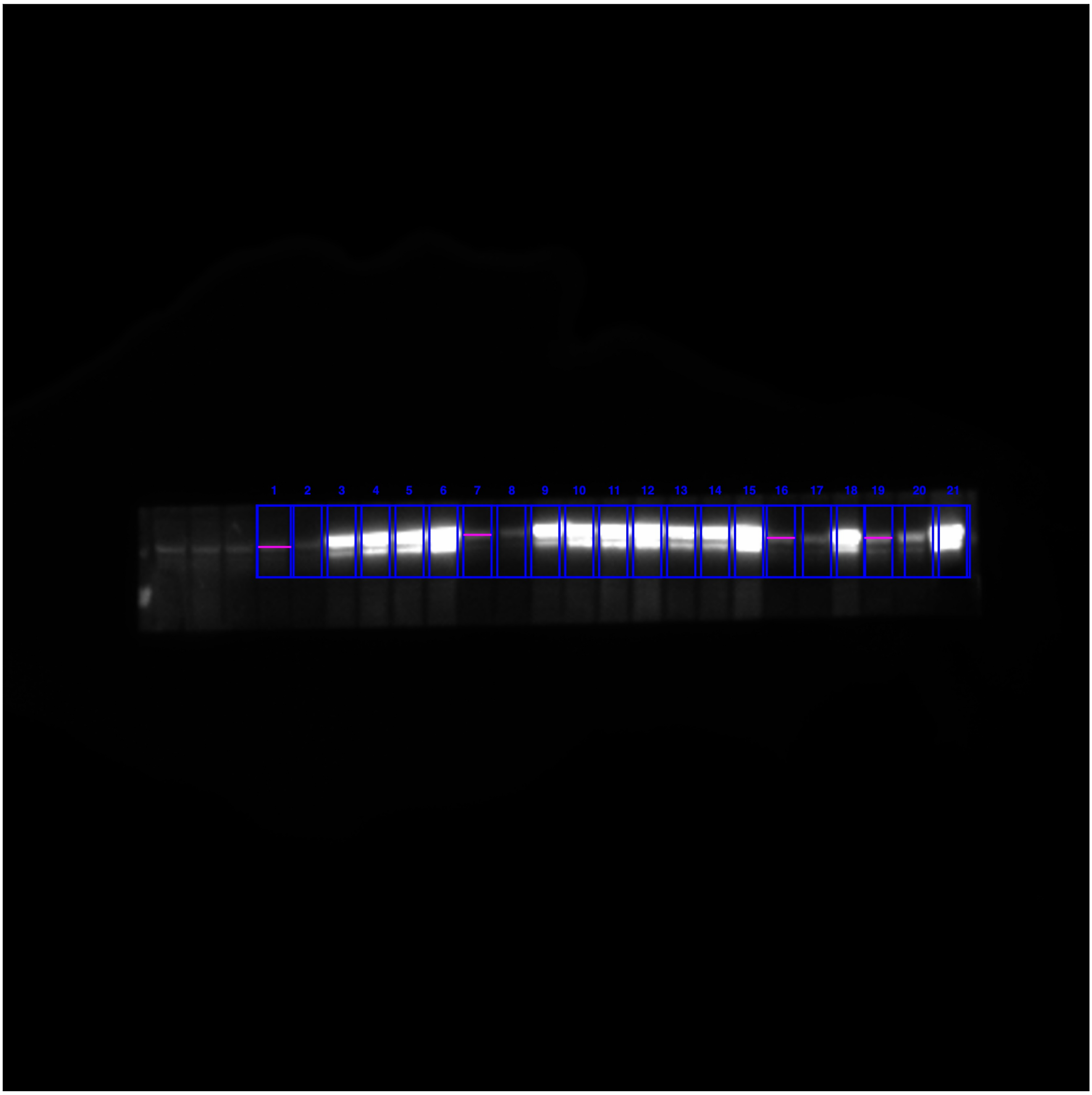

/Users/mariejeanpierre/Desktop/IM004419_03-2 (glissées).tiff

Acquisition Information

Image Information

Acquisition Date	unknown
User Name	Marie Jeanpierre
Image Area (mm)	X: 15.2 Y: 15.2

Pixel Size (μm)	X: 14.1 Y: 14.1
Data Range (Int)	130 - 65534

Analysis Settings

Detection	Lane detection:<br>Manually created lanes<br><br>Band detection:<br><br>Manually adjusted bands<br><br>Lane Background Subtraction:<br>Lane background subtracted with disk size: 0.1<br><br>Lane width: Variable

Lane Statistics

Lane No.	Adj. Total Band Vol. (Int)	Total Band Vol. (Int)	Adj. Total Lane Vol. (Int)	Total Lane Vol. (Int)	Bkgd. Vol. (Int)	Norm. Factor
1	1 871 859	6 268 350	2 110 350	13 886 796	11 776 446	N/A
2	N/A	N/A	1 876 756	12 356 596	10 479 840	N/A
3	N/A	N/A	15 433 684	44 991 352	29 557 668	N/A
4	N/A	N/A	13 071 940	55 514 760	42 442 820	N/A
5	N/A	N/A	12 417 384	56 916 664	44 499 280	N/A
6	N/A	N/A	10 408 384	64 544 144	54 135 760	N/A
7	3 369 744	8 661 380	3 401 384	14 769 076	11 367 692	N/A
8	N/A	N/A	2 240 336	12 143 572	9 903 236	N/A
9	N/A	N/A	14 948 948	48 977 628	34 028 680	N/A
10	N/A	N/A	11 396 504	57 696 968	46 300 464	N/A
11	N/A	N/A	5 808 824	59 090 668	53 281 844	N/A
12	N/A	N/A	11 482 996	63 232 036	51 749 040	N/A
13	N/A	N/A	5 940 508	48 736 604	42 796 096	N/A
14	N/A	N/A	15 230 544	51 033 388	35 802 844	N/A
15	N/A	N/A	11 894 988	65 871 176	53 976 188	N/A
16	3 519 824	9 018 688	3 656 408	15 269 884	11 613 476	N/A
17	N/A	N/A	3 870 748	15 544 900	11 674 152	N/A
18	N/A	N/A	39 356 716	49 543 368	10 186 652	N/A
19	4 389 616	10 953 936	4 804 464	20 059 536	15 255 072	N/A
20	N/A	N/A	17 371 228	25 593 036	8 221 808	N/A
21	N/A	N/A	10 322 144	53 866 344	43 544 200	N/A

Lane And Band Analysis

Lane 1

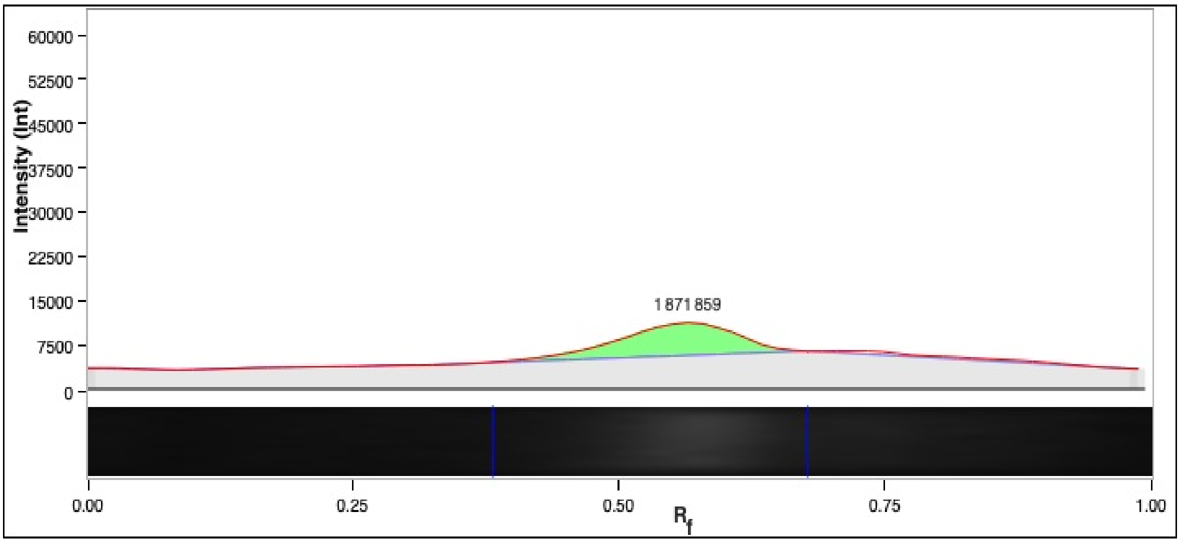

Band No.	Band Label	Mol. Wt. (KDa)	Relative Front	Adj. Volume (Int)	Volume (Int)	Abs. Quant.	Rel. Quant.	Band %	Lane %
1		N/A	0,577	1 871 859	6 268 350	N/A	N/A	100,0	88,7

Lane Background	Lane background subtracted with disk size: 0.1
Lane Width	0.47 mm

Lane 2

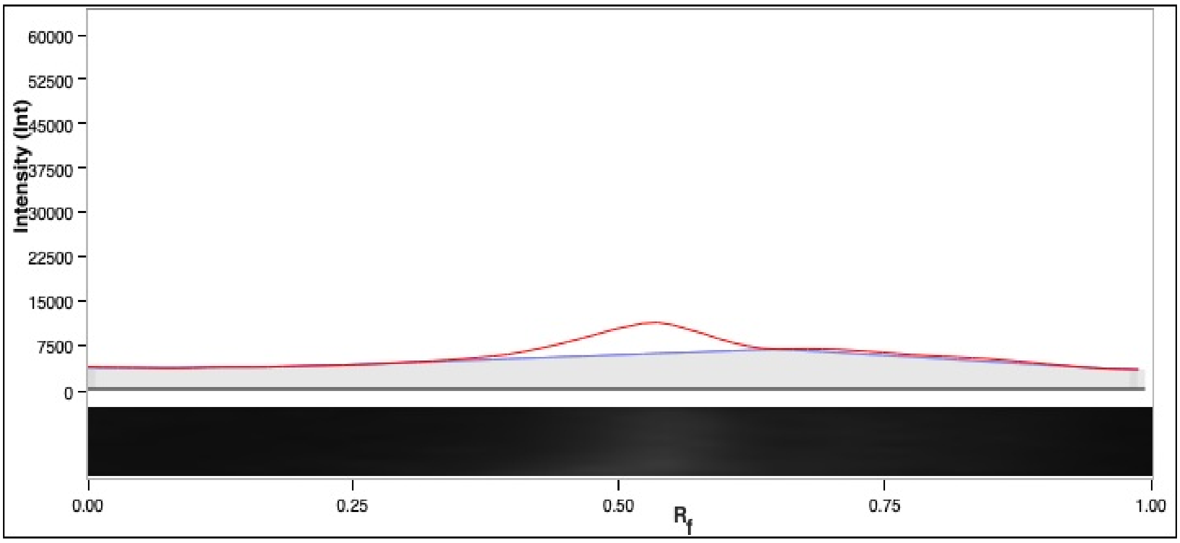

Band No.	Band Label	Mol. Wt. (KDa)	Relative Front	Adj. Volume (Int)	Volume (Int)	Abs. Quant.	Rel. Quant.	Band %	Lane %

Lane Background	Lane background subtracted with disk size: 0.1
Lane Width	0.40 mm

Lane 3

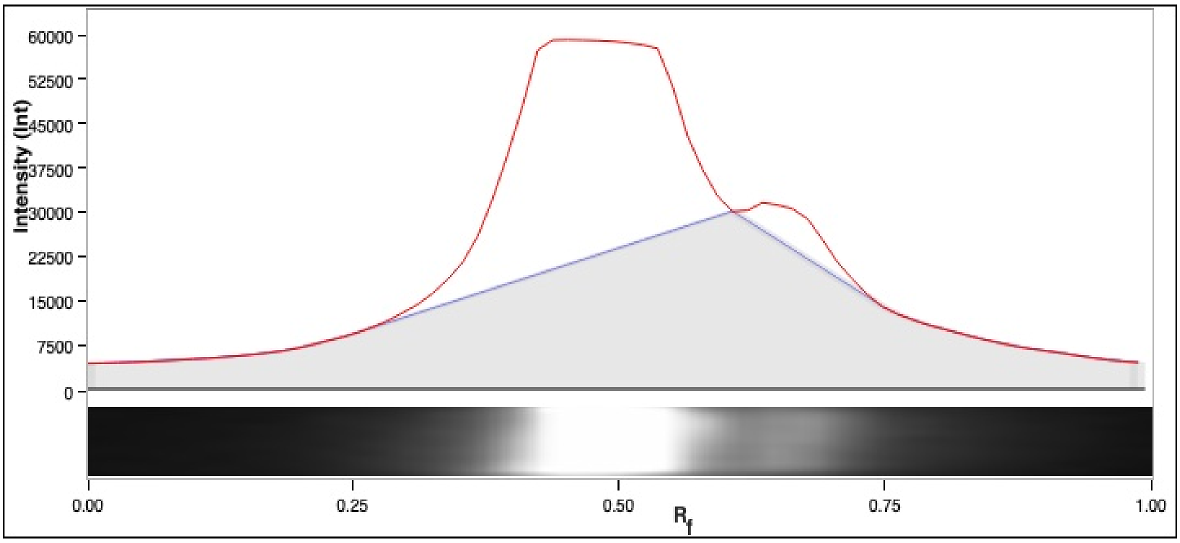

Band No.	Band Label	Mol. Wt. (KDa)	Relative Front	Adj. Volume (Int)	Volume (Int)	Abs. Quant.	Rel. Quant.	Band %	Lane %

Lane Background	Lane background subtracted with disk size: 0.1
Lane Width	0.40 mm

Lane 4

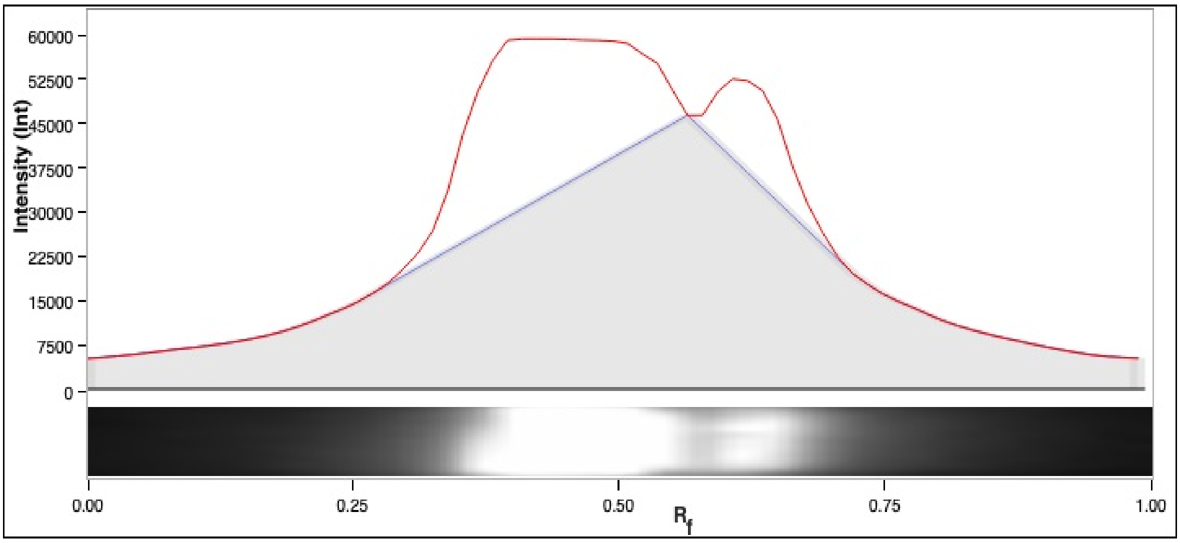

Band No.	Band Label	Mol. Wt. (KDa)	Relative Front	Adj. Volume (Int)	Volume (Int)	Abs. Quant.	Rel. Quant.	Band %	Lane %

Lane Background	Lane background subtracted with disk size: 0.1
Lane Width	0.40 mm

Lane 5

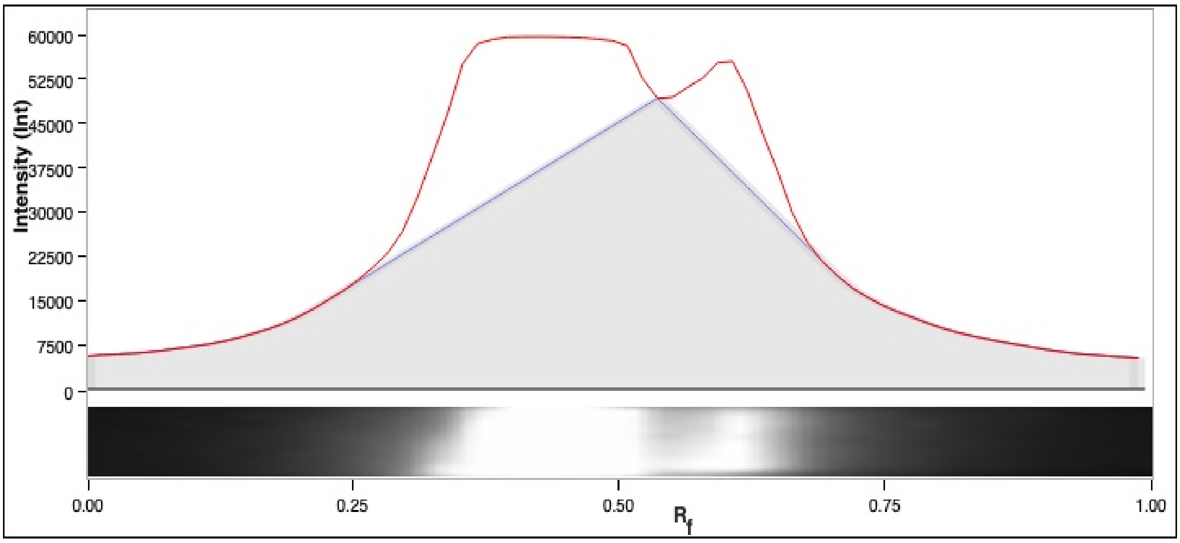

Band No.	Band Label	Mol. Wt. (KDa)	Relative Front	Adj. Volume (Int)	Volume (Int)	Abs. Quant.	Rel. Quant.	Band %	Lane %

Lane Background	Lane background subtracted with disk size: 0.1
Lane Width	0.40 mm

Lane 6

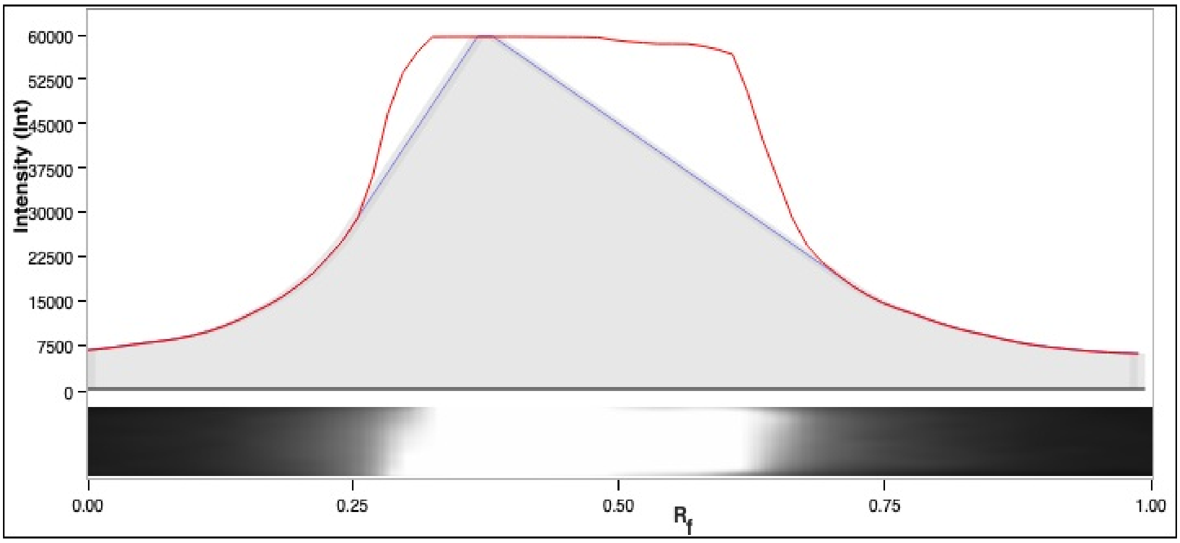

Band No.	Band Label	Mol. Wt. (KDa)	Relative Front	Adj. Volume (Int)	Volume (Int)	Abs. Quant.	Rel. Quant.	Band %	Lane %

Lane Background	Lane background subtracted with disk size: 0.1

Lane Width	0.40 mm

Lane 7

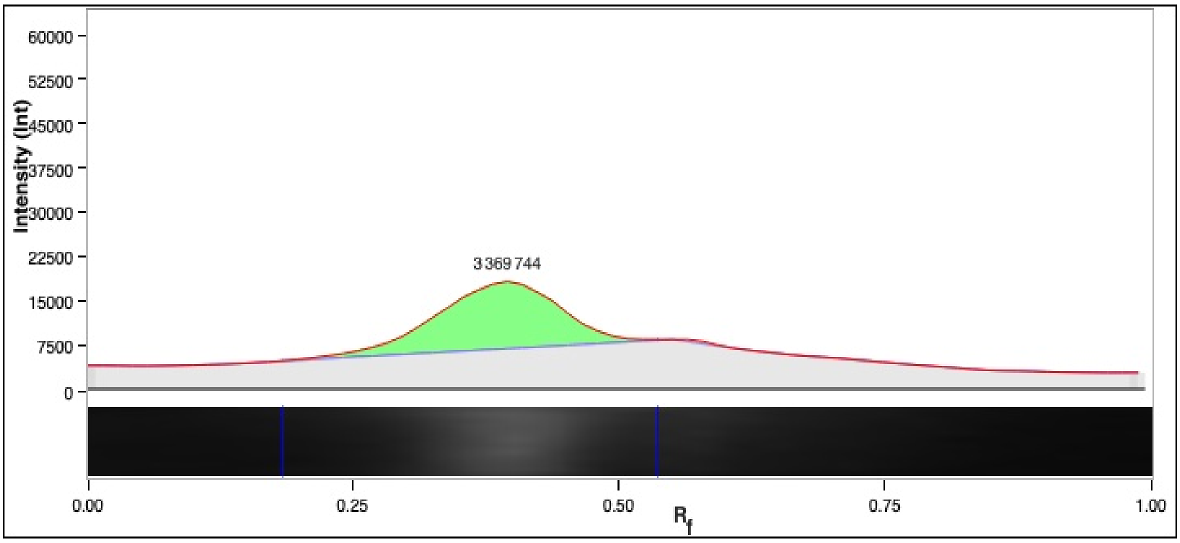

Band No.	Band Label	Mol. Wt. (KDa)	Relative Front	Adj. Volume (Int)	Volume (Int)	Abs. Quant.	Rel. Quant.	Band %	Lane %
1		N/A	0,408	3 369 744	8 661 380	N/A	N/A	100,0	99,1

Lane Background	Lane background subtracted with disk size: 0.1
Lane Width	0.40 mm

Lane 8

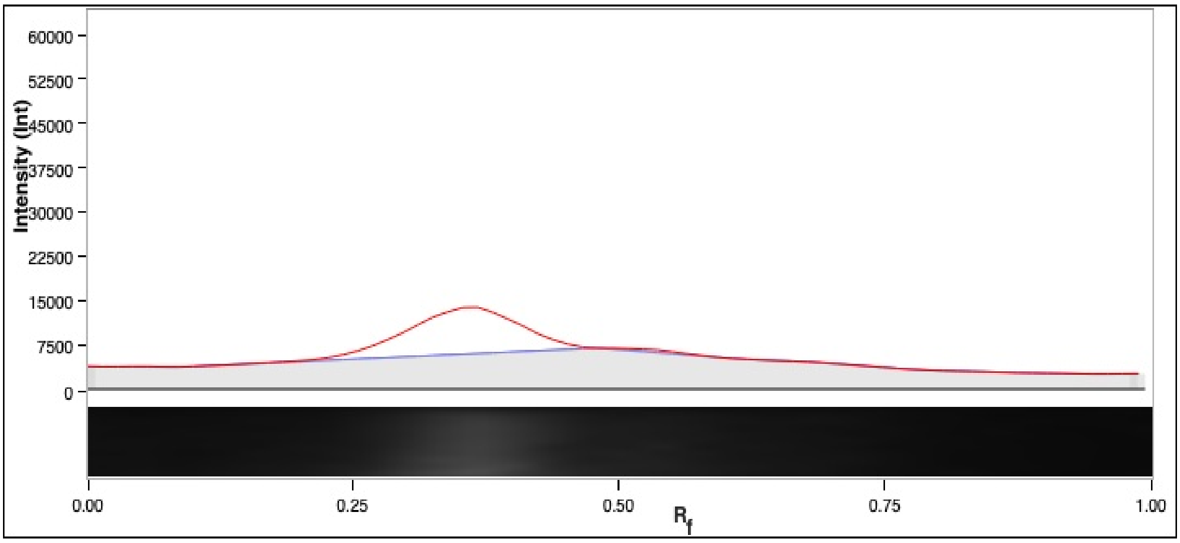

Band No.	Band Label	Mol. Wt. (KDa)	Relative Front	Adj. Volume (Int)	Volume (Int)	Abs. Quant.	Rel. Quant.	Band %	Lane %

Lane Background	Lane background subtracted with disk size: 0.1
Lane Width	0.40 mm

Lane 9

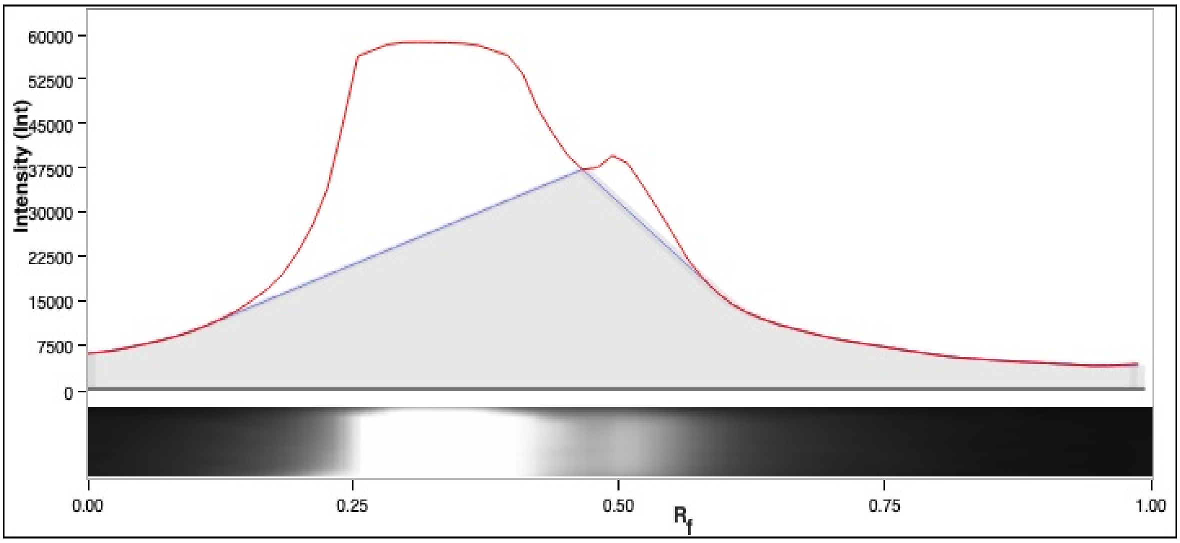

Band No.	Band Label	Mol. Wt. (KDa)	Relative Front	Adj. Volume (Int)	Volume (Int)	Abs. Quant.	Rel. Quant.	Band %	Lane %

Lane Background	Lane background subtracted with disk size: 0.1
Lane Width	0.40 mm

Lane 10

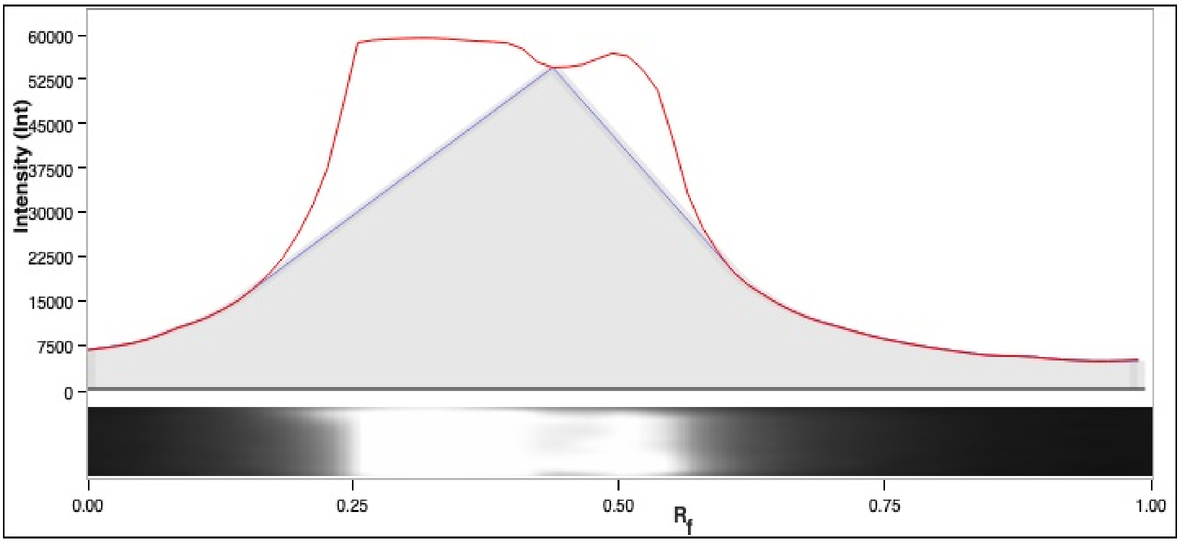

Band No.	Band Label	Mol. Wt. (KDa)	Relative Front	Adj. Volume (Int)	Volume (Int)	Abs. Quant.	Rel. Quant.	Band %	Lane %

Lane Background	Lane background subtracted with disk size: 0.1
Lane Width	0.40 mm

Lane 11

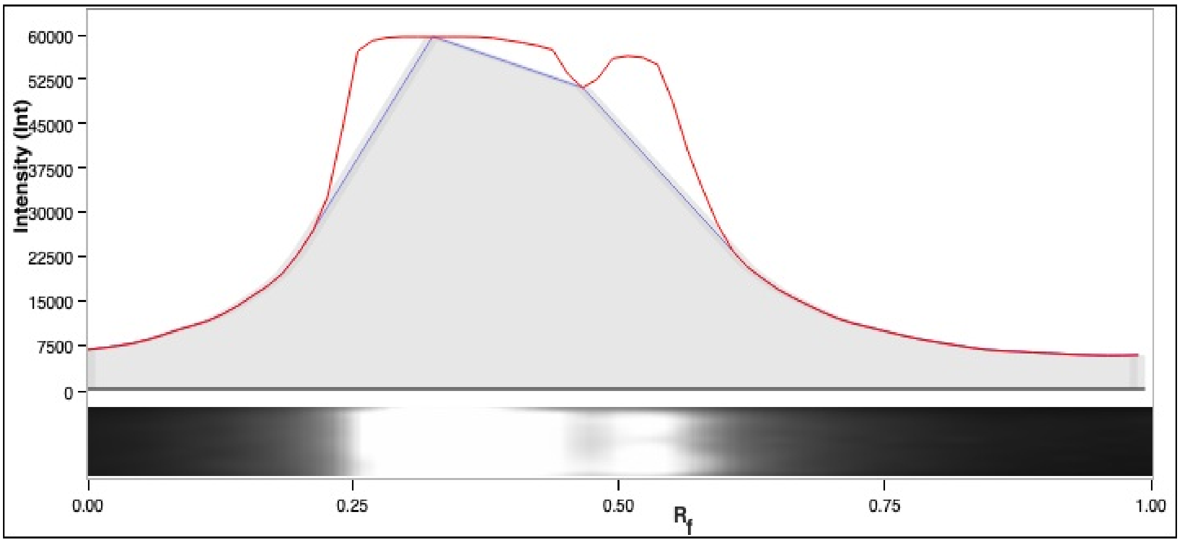

Band No.	Band Label	Mol. Wt. (KDa)	Relative Front	Adj. Volume (Int)	Volume (Int)	Abs. Quant.	Rel. Quant.	Band %	Lane %

Lane Background	Lane background subtracted with disk size: 0.1
Lane Width	0.40 mm

Lane 12

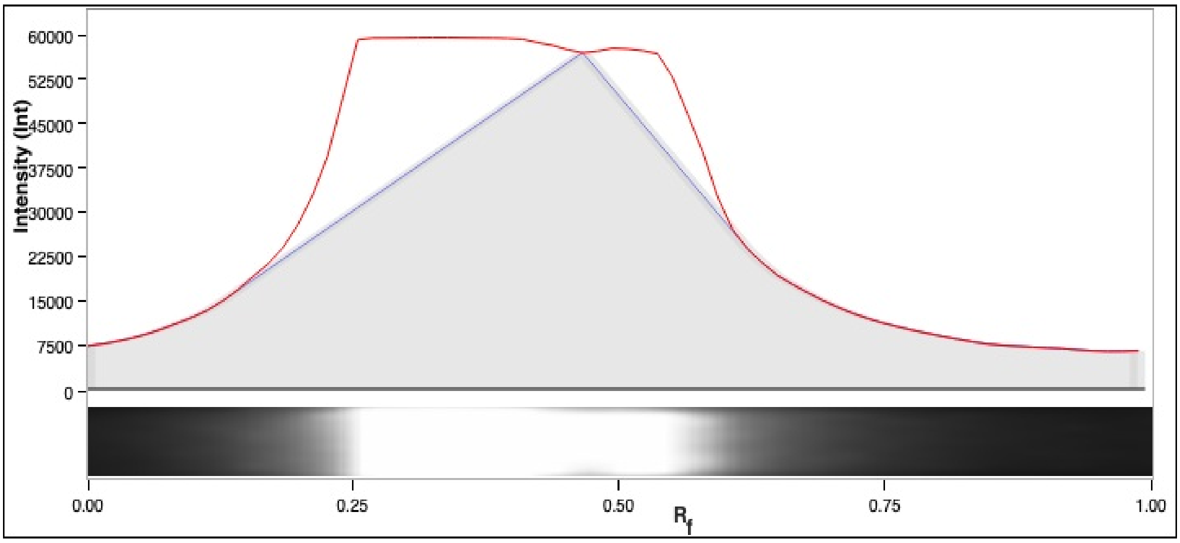

Band No.	Band	Mol. Wt.	Relative	Adj.	Volume	Abs.	Rel.	Band %	Lane %

	Label	(KDa)	Front	Volume (Int)	(Int)	Quant.	Quant.		

Lane Background	Lane background subtracted with disk size: 0.1
Lane Width	0.40 mm

Lane 13

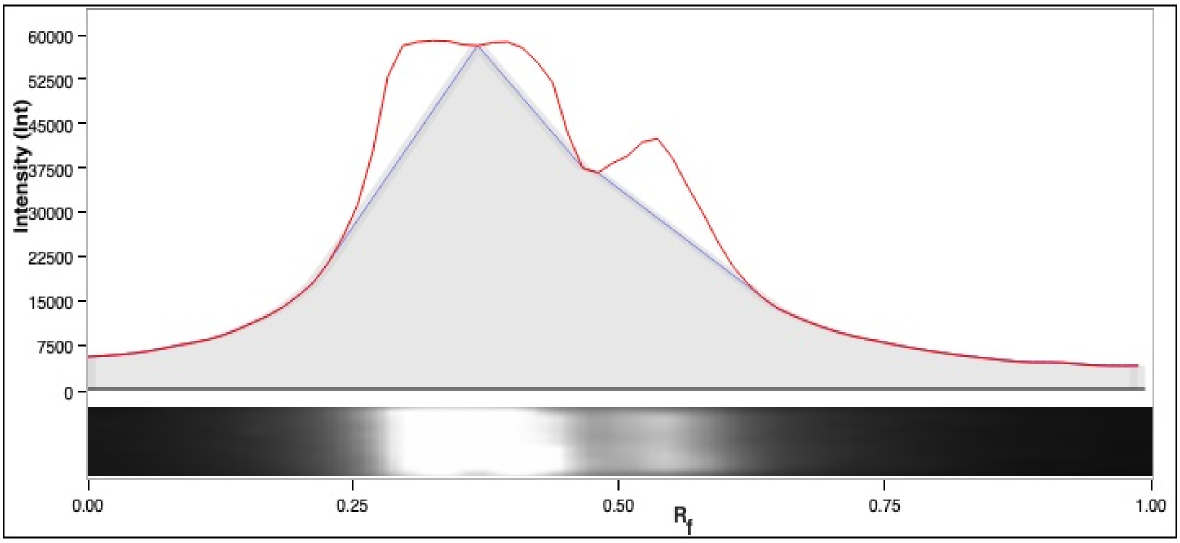

Band No.	Band Label	Mol. Wt. (KDa)	Relative Front	Adj. Volume (Int)	Volume (Int)	Abs. Quant.	Rel. Quant.	Band %	Lane %

Lane Background	Lane background subtracted with disk size: 0.1
Lane Width	0.40 mm

Lane 14

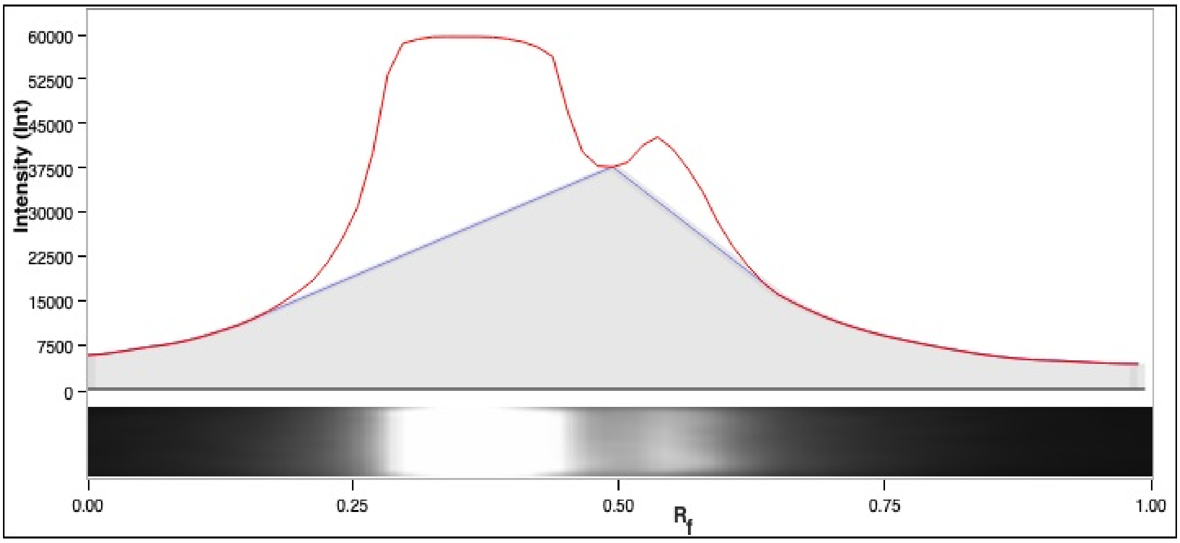

Band No.	Band Label	Mol. Wt. (KDa)	Relative Front	Adj. Volume (Int)	Volume (Int)	Abs. Quant.	Rel. Quant.	Band %	Lane %

Lane Background	Lane background subtracted with disk size: 0.1
Lane Width	0.40 mm

Lane 15

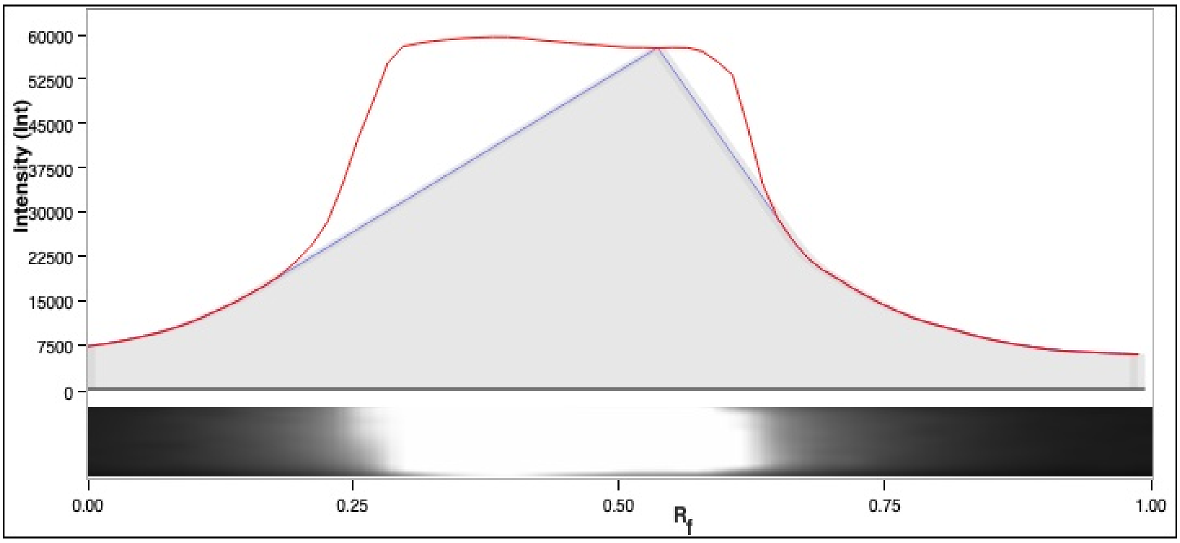

Band No.	Band Label	Mol. Wt. (KDa)	Relative Front	Adj. Volume (Int)	Volume (Int)	Abs. Quant.	Rel. Quant.	Band %	Lane %

Lane Background	Lane background subtracted with disk size: 0.1
Lane Width	0.40 mm

Lane 16

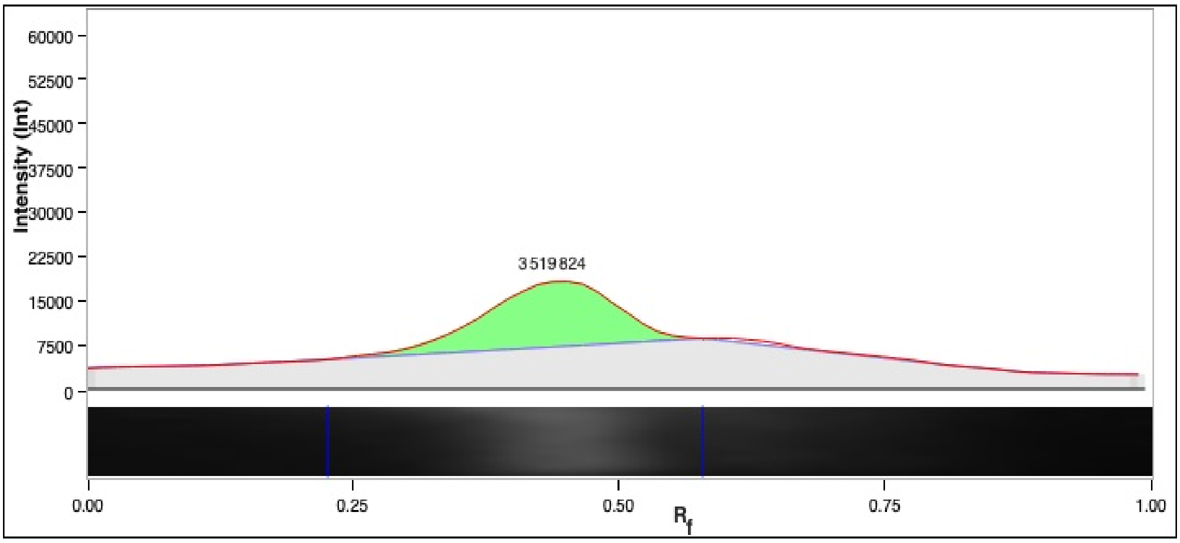

Band No.	Band Label	Mol. Wt. (KDa)	Relative Front	Adj. Volume (Int)	Volume (Int)	Abs. Quant.	Rel. Quant.	Band %	Lane %
1		N/A	0,451	3 519 824	9 018 688	N/A	N/A	100,0	96,3

Lane Background	Lane background subtracted with disk size: 0.1
Lane Width	0.40 mm

Lane 17

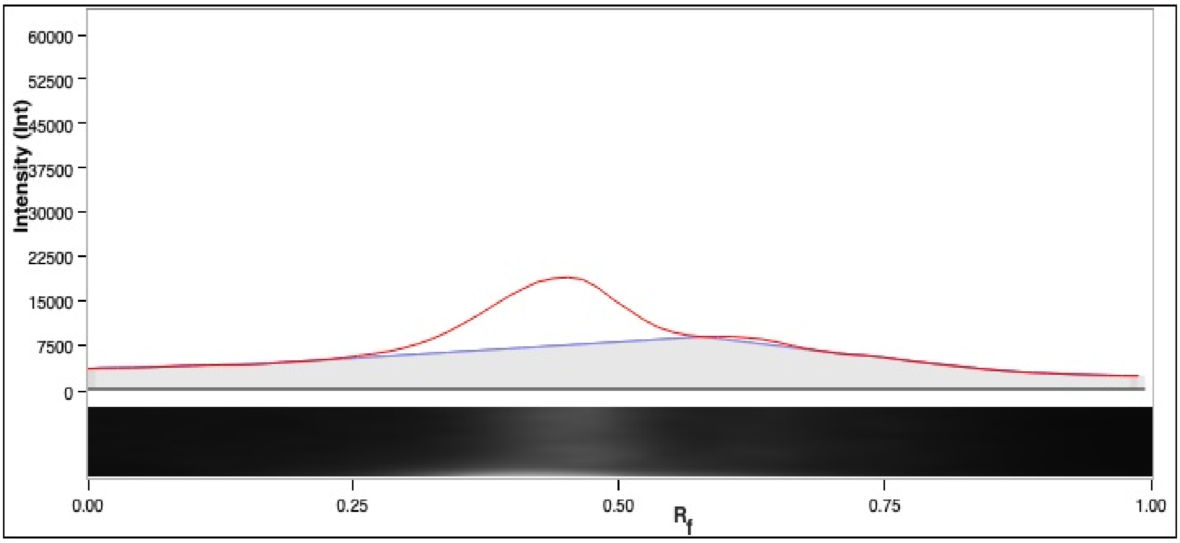

Band No.	Band Label	Mol. Wt. (KDa)	Relative Front	Adj. Volume (Int)	Volume (Int)	Abs. Quant.	Rel. Quant.	Band %	Lane %

Lane Background	Lane background subtracted with disk size: 0.1
Lane Width	0.40 mm

Lane 18

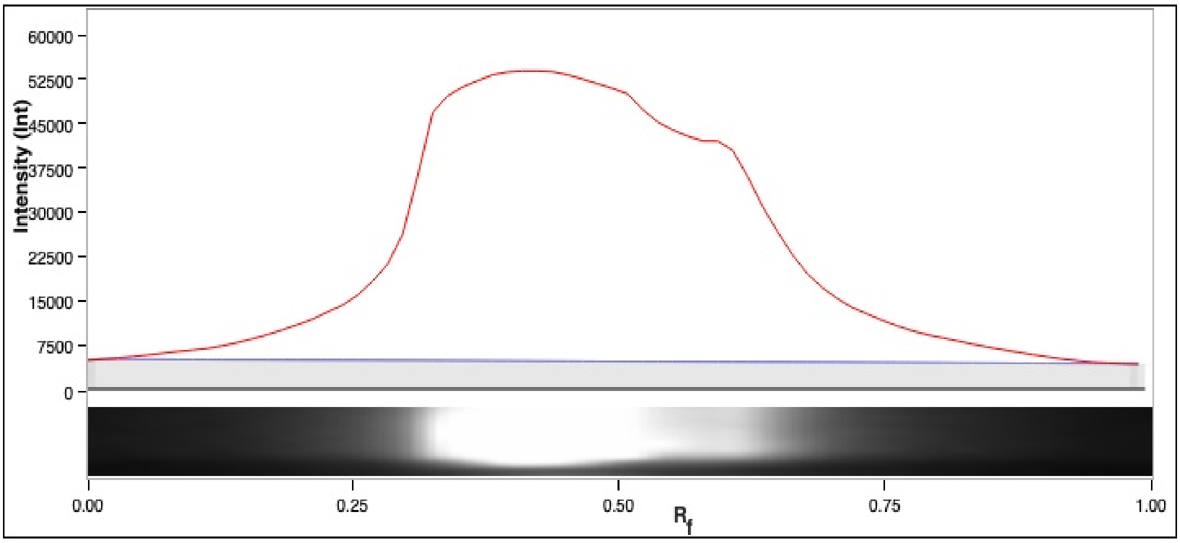

Band No.	Band Label	Mol. Wt. (KDa)	Relative Front	Adj. Volume (Int)	Volume (Int)	Abs. Quant.	Rel. Quant.	Band %	Lane %

Lane Background	Lane background subtracted with disk size: 0.1
Lane Width	0.40 mm

Lane 19

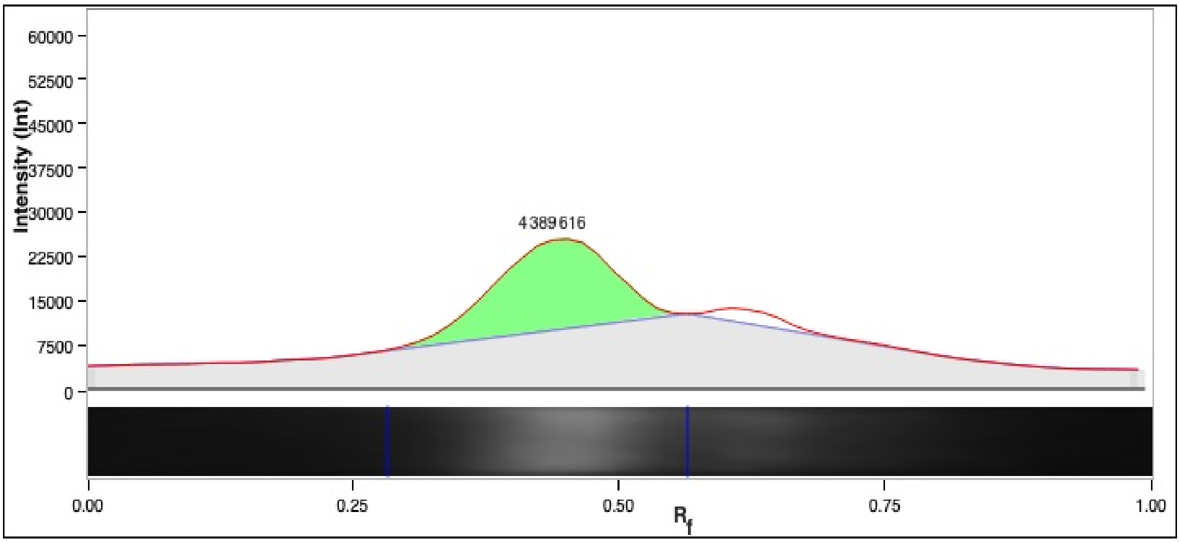

Band No.	Band Label	Mol. Wt. (KDa)	Relative Front	Adj. Volume (Int)	Volume (Int)	Abs. Quant.	Rel. Quant.	Band %	Lane %
1		N/A	0,451	4 389 616	10 953 936	N/A	N/A	100,0	91,4

Lane Background	Lane background subtracted with disk size: 0.1
Lane Width	0.40 mm

Lane 20

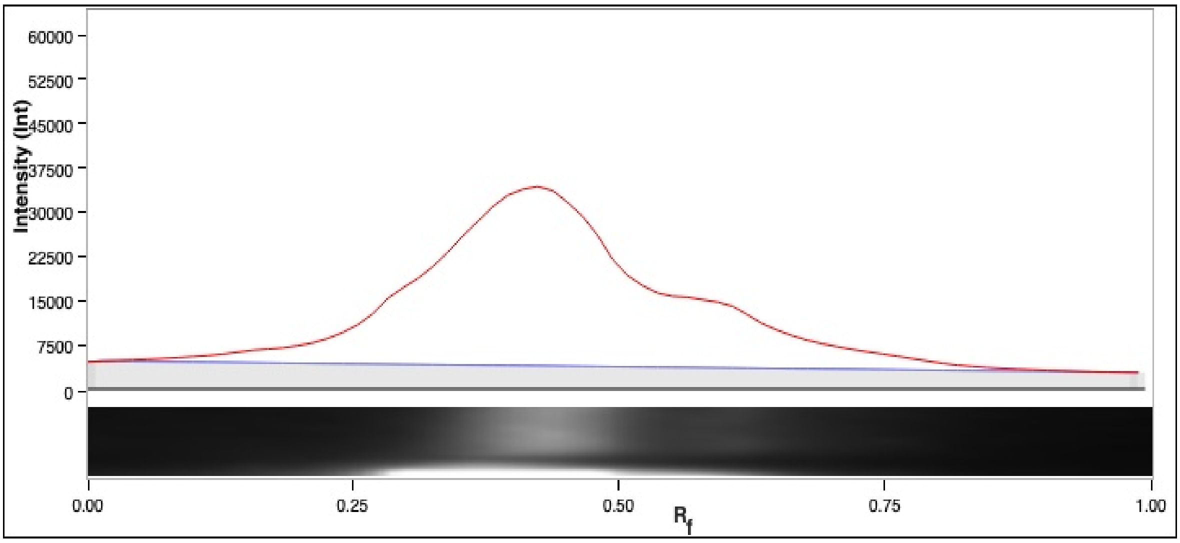

Band No.	Band Label	Mol. Wt. (KDa)	Relative Front	Adj. Volume (Int)	Volume (Int)	Abs. Quant.	Rel. Quant.	Band %	Lane %

Lane Background	Lane background subtracted with disk size: 0.1
Lane Width	0.40 mm

Lane 21

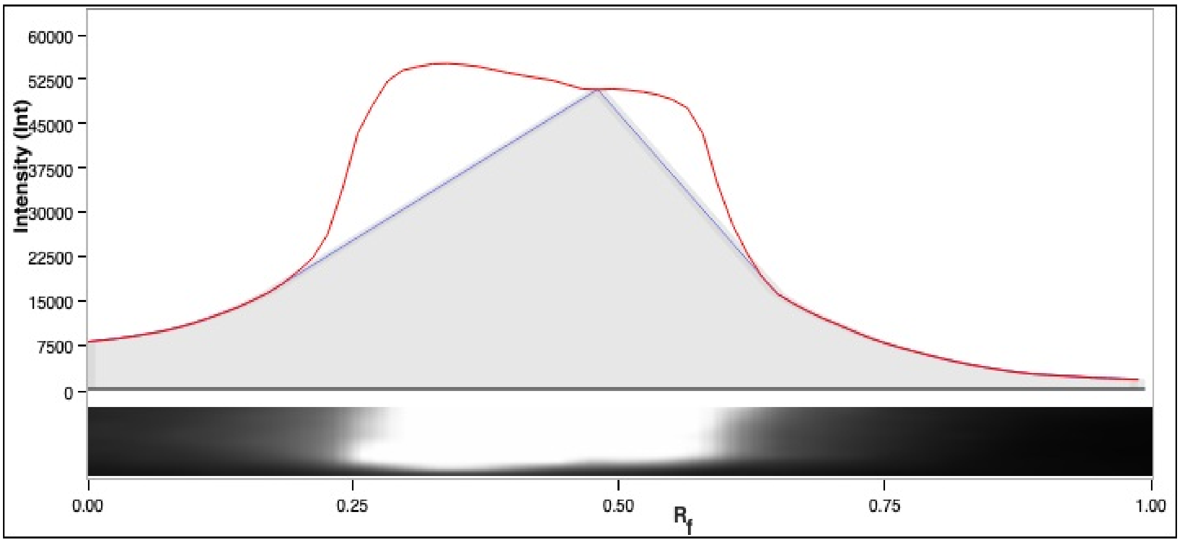

Band No.	Band Label	Mol. Wt. (KDa)	Relative Front	Adj. Volume (Int)	Volume (Int)	Abs. Quant.	Rel. Quant.	Band %	Lane %

Lane Background	Lane background subtracted with disk size: 0.1
Lane Width	0.40 mm
