# Supplementary material for: In silico modeling guides identification of novel JAK1 variants associated with immune dysregulation
Source: EMBO Mol Med. 2025 Oct 24;17(12):3275–99. doi: 10.1038/s44321-025-00317-0 (PMC12686074; doi:10.1038/s44321-025-00317-0)
Supplement: Supplementary file 8 — Source data Fig. 3 [file 44321_2025_317_MOESM8_ESM.zip › Figure 3/Replicates Fig.3A/wb 5/GAPDH membrane 1.pdf]

Image Report: GAPDH membrane 1

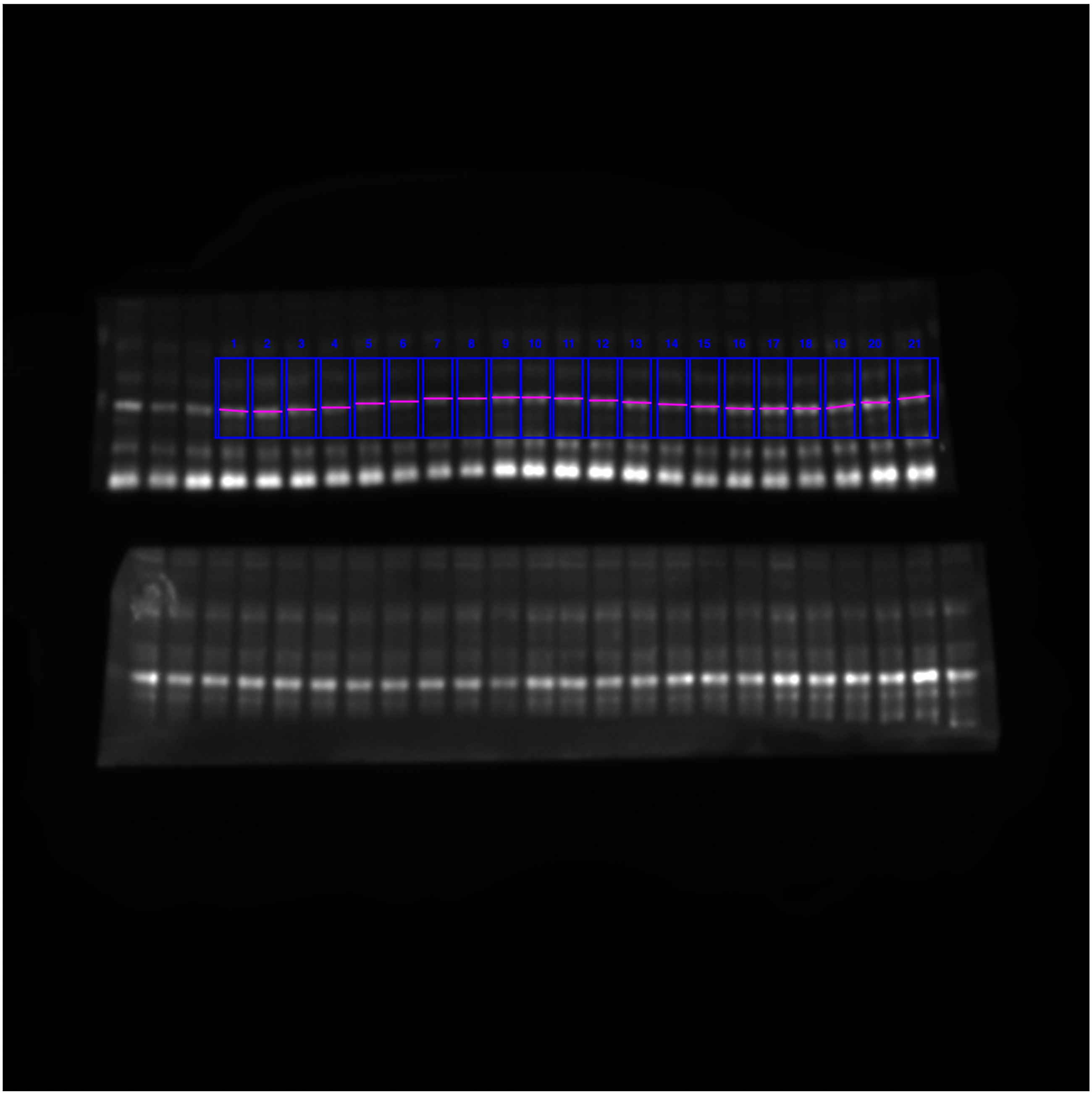

/Volumes/FRL-lab/FRL's Team/Marie Jeanpierre/JAK1/Papier JAK1/Nouvelle submission EMBO/ Source data/Figure 3/3A/Western 5/GAPDH membrane 1.scn

---

Acquisition Information

Image Information

Acquisition Date	unknown
User Name	Marie Jeanpierre

Image Area (mm)	X: 15.2 Y: 15.2
Pixel Size (µm)	X: 14.1 Y: 14.1
Data Range (Int)	157 - 65534

Analysis Settings

Detection	Lane detection:<br>Manually created lanes<br><br>Band detection:<br><br>Manually adjusted bands<br><br>Lane Background Subtraction:<br>Lane background subtracted with disk size: 0.1<br><br>Lane width: Variable

Lane Statistics

Lane No.	Adj. Total Band Vol. (Int)	Total Band Vol. (Int)	Adj. Total Lane Vol. (Int)	Total Lane Vol. (Int)	Bkgd. Vol. (Int)	Norm. Factor
1	3 283 873	7 587 038	4 441 466	19 576 102	15 134 636	N/A
2	3 859 059	8 786 014	5 607 730	21 754 495	16 146 765	N/A
3	3 048 016	7 828 318	4 335 442	20 400 572	16 065 130	N/A
4	2 469 785	6 747 053	3 357 997	17 823 661	14 465 664	N/A
5	2 353 814	6 304 223	3 289 151	17 060 294	13 771 143	N/A
6	2 129 035	5 183 605	3 207 487	15 106 448	11 898 961	N/A
7	2 325 742	5 235 892	3 540 465	14 969 771	11 429 306	N/A
8	1 754 413	4 852 831	2 790 380	15 557 862	12 767 482	N/A
9	3 303 100	8 219 905	4 909 091	20 772 062	15 862 971	N/A
10	3 188 260	7 643 820	5 217 506	21 151 933	15 934 427	N/A
11	3 455 640	7 745 552	5 402 845	20 642 722	15 239 877	N/A
12	3 029 920	6 806 793	4 633 388	18 725 126	14 091 738	N/A
13	3 706 548	7 598 667	5 312 481	19 646 891	14 334 410	N/A
14	2 951 678	6 619 047	4 192 472	17 480 852	13 288 380	N/A
15	3 414 779	7 060 949	4 599 371	17 147 294	12 547 923	N/A
16	4 097 091	7 934 980	5 544 945	18 354 680	12 809 735	N/A
17	4 601 894	9 106 522	6 085 998	20 636 197	14 550 199	N/A
18	5 226 699	10 050 849	6 621 773	21 612 192	14 990 419	N/A
19	4 371 982	9 459 394	5 595 869	21 424 069	15 828 200	N/A
20	6 035 480	10 968 322	7 800 971	23 567 807	15 766 836	N/A
21	4 214 272	8 814 272	6 455 808	22 219 264	15 763 456	N/A

Lane And Band Analysis

Lane 1

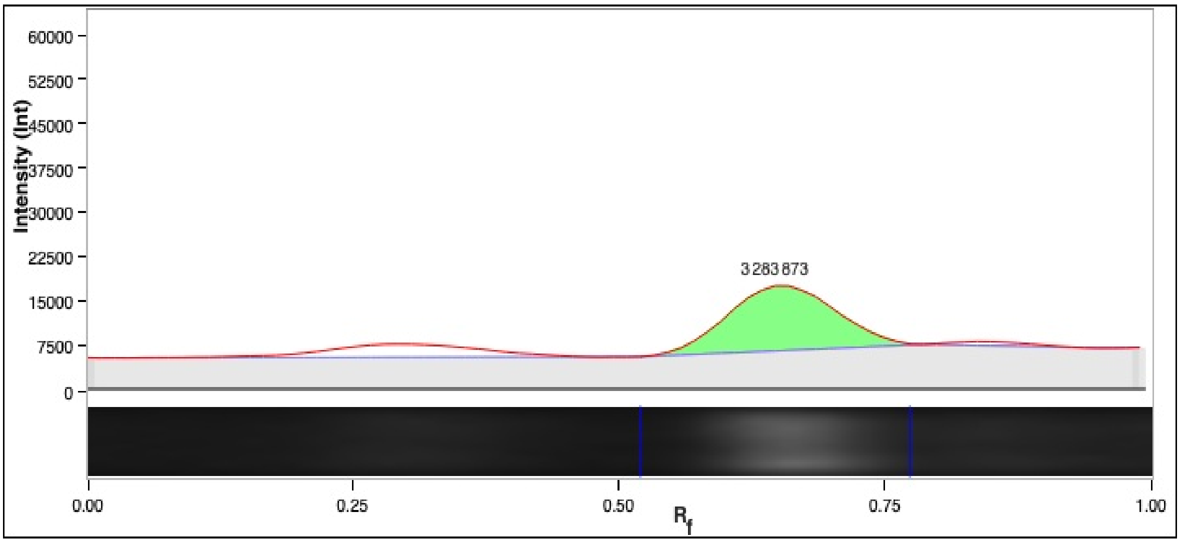

Band No.	Band Label	Mol. Wt. (KDa)	Relative Front	Adj. Volume (Int)	Volume (Int)	Abs. Quant.	Rel. Quant.	Band %	Lane %
1		N/A	0,658	3 283 873	7 587 038	N/A	N/A	100,0	73,9

Lane Background	Lane background subtracted with disk size: 0.1
Lane Width	0.41 mm

Lane 2

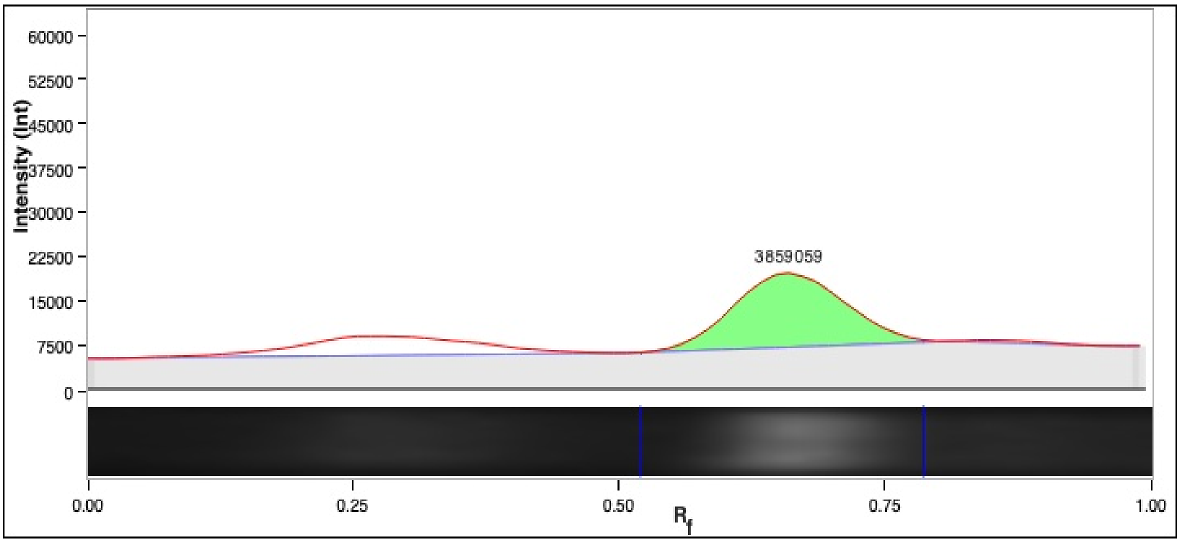

Band No.	Band Label	Mol. Wt. (KDa)	Relative Front	Adj. Volume (Int)	Volume (Int)	Abs. Quant.	Rel. Quant.	Band %	Lane %
1		N/A	0,671	3 859 059	8 786 014	N/A	N/A	100,0	68,8

Lane Background	Lane background subtracted with disk size: 0.1
Lane Width	0.41 mm

Lane 3

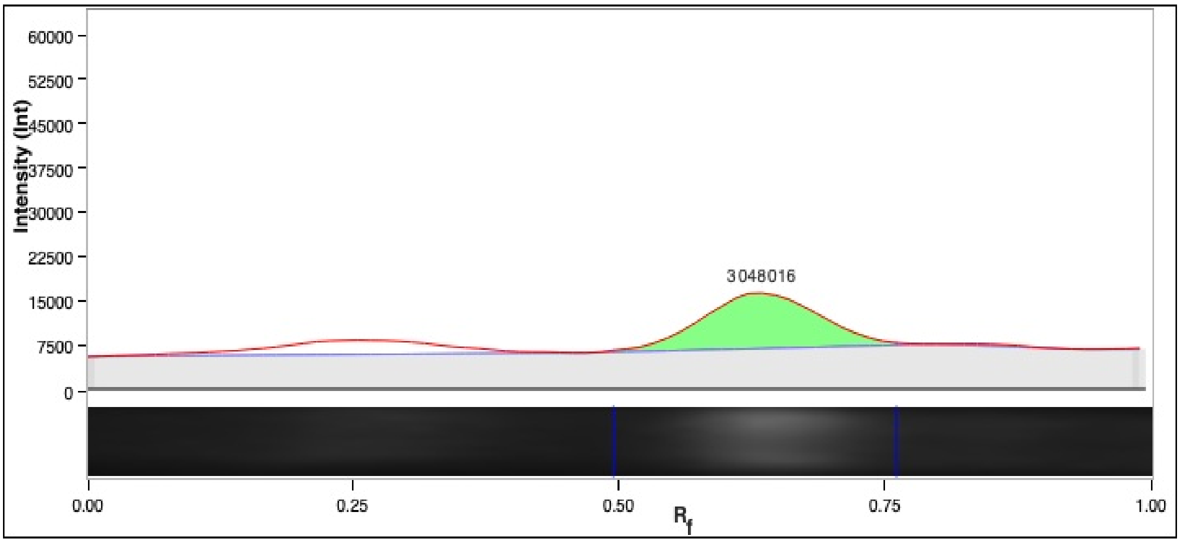

Band No.	Band Label	Mol. Wt. (KDa)	Relative Front	Adj. Volume (Int)	Volume (Int)	Abs. Quant.	Rel. Quant.	Band %	Lane %
1		N/A	0,646	3 048 016	7 828 318	N/A	N/A	100,0	70,3

Lane Background	Lane background subtracted with disk size: 0.1
Lane Width	0.41 mm

Lane 4

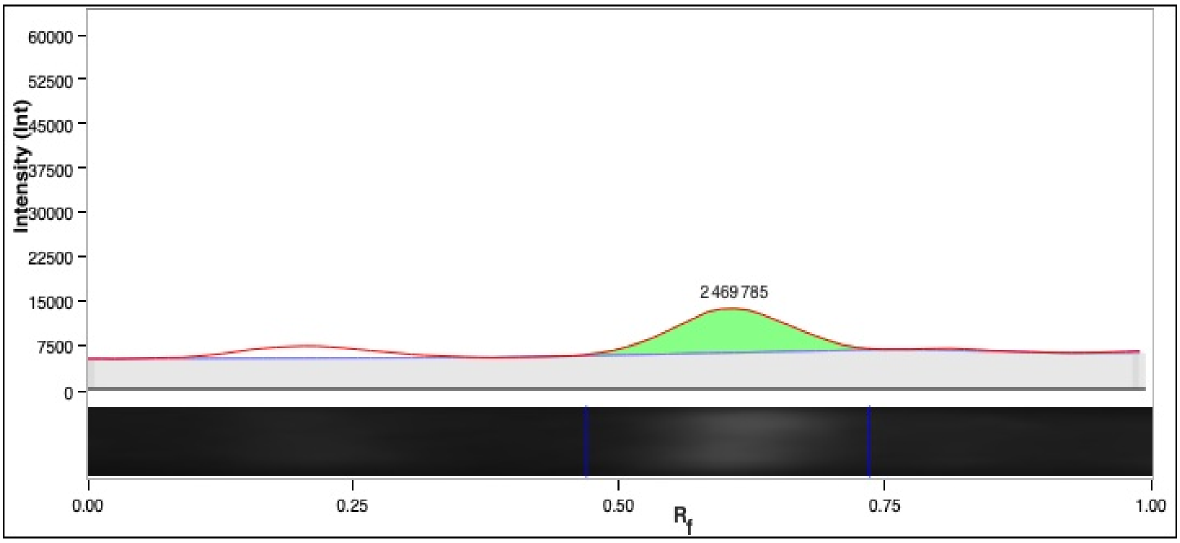

Band No.	Band Label	Mol. Wt. (KDa)	Relative Front	Adj. Volume (Int)	Volume (Int)	Abs. Quant.	Rel. Quant.	Band %	Lane %
1		N/A	0,620	2 469 785	6 747 053	N/A	N/A	100,0	73,5

Lane Background	Lane background subtracted with disk size: 0.1
Lane Width	0.41 mm

Lane 5

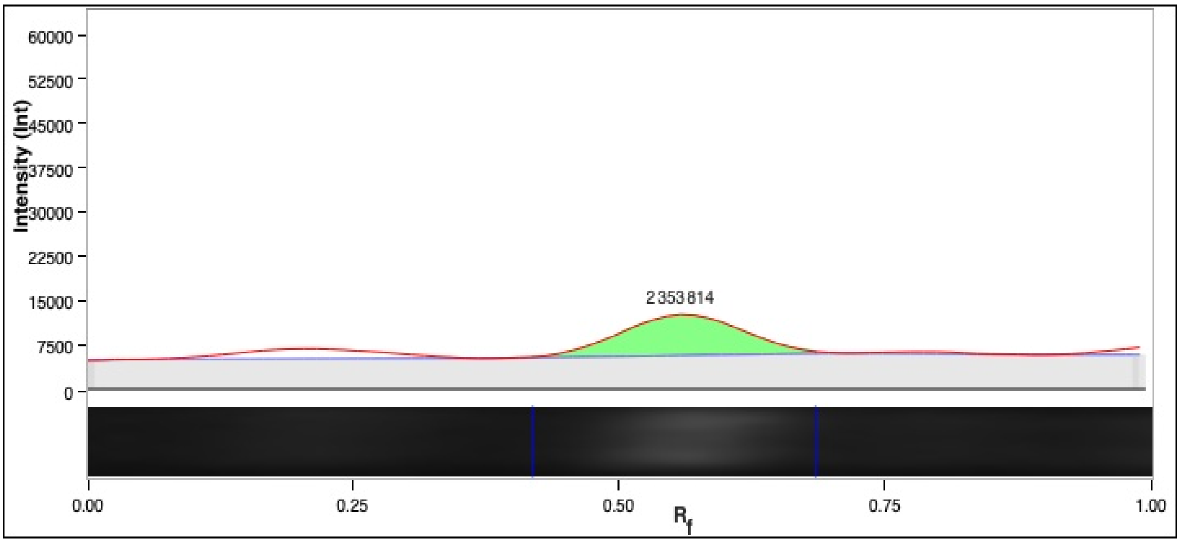

Band No.	Band Label	Mol. Wt. (KDa)	Relative Front	Adj. Volume (Int)	Volume (Int)	Abs. Quant.	Rel. Quant.	Band %	Lane %
1		N/A	0,570	2 353 814	6 304 223	N/A	N/A	100,0	71,6

Lane Background	Lane background subtracted with disk size: 0.1
Lane Width	0.41 mm

Lane 6

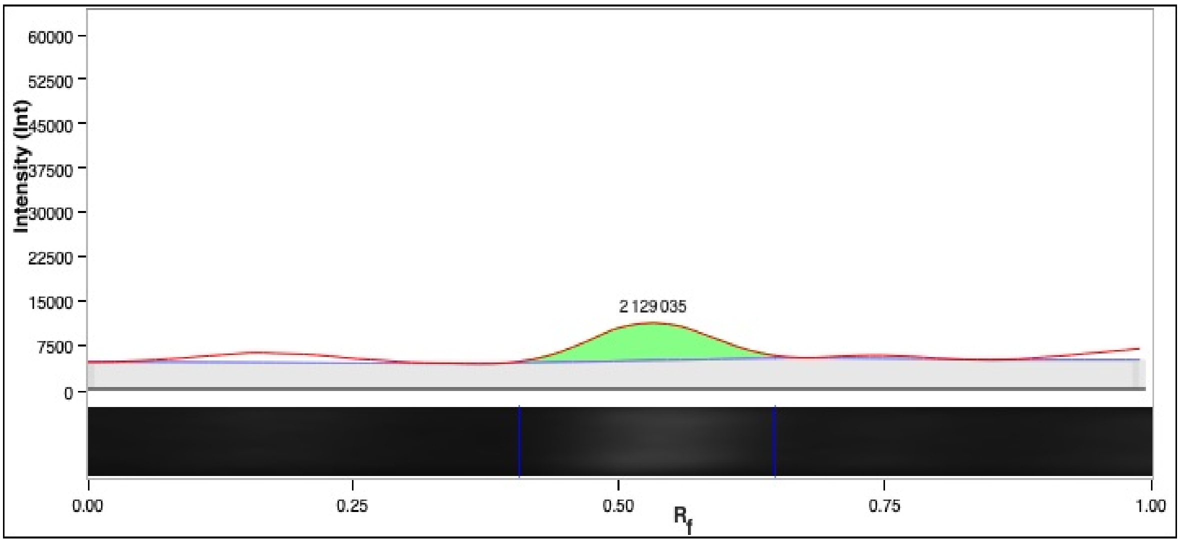

Band No.	Band Label	Mol. Wt. (KDa)	Relative Front	Adj. Volume (Int)	Volume (Int)	Abs. Quant.	Rel. Quant.	Band %	Lane %
1		N/A	0,544	2 129 035	5 183 605	N/A	N/A	100,0	66,4

Lane Background	Lane background subtracted with disk size: 0.1

Lane Width	0.41 mm

Lane 7

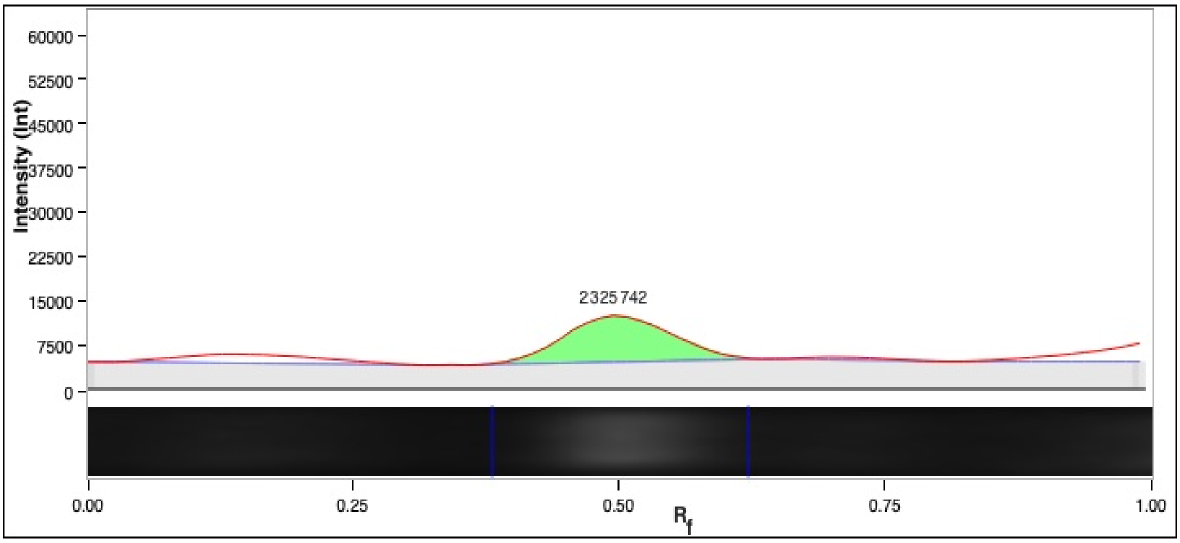

Band No.	Band Label	Mol. Wt. (KDa)	Relative Front	Adj. Volume (Int)	Volume (Int)	Abs. Quant.	Rel. Quant.	Band %	Lane %
1		N/A	0,506	2 325 742	5 235 892	N/A	N/A	100,0	65,7

Lane Background	Lane background subtracted with disk size: 0.1
Lane Width	0.41 mm

Lane 8

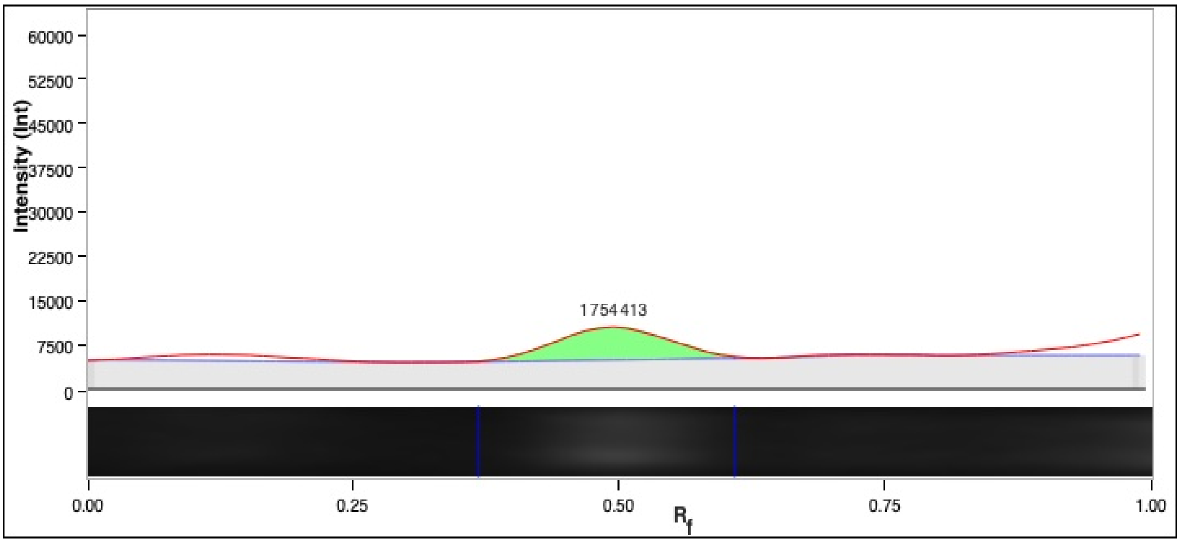

Band No.	Band Label	Mol. Wt. (KDa)	Relative Front	Adj. Volume (Int)	Volume (Int)	Abs. Quant.	Rel. Quant.	Band %	Lane %
1		N/A	0,506	1 754 413	4 852 831	N/A	N/A	100,0	62,9

Lane Background	Lane background subtracted with disk size: 0.1
Lane Width	0.41 mm

Lane 9

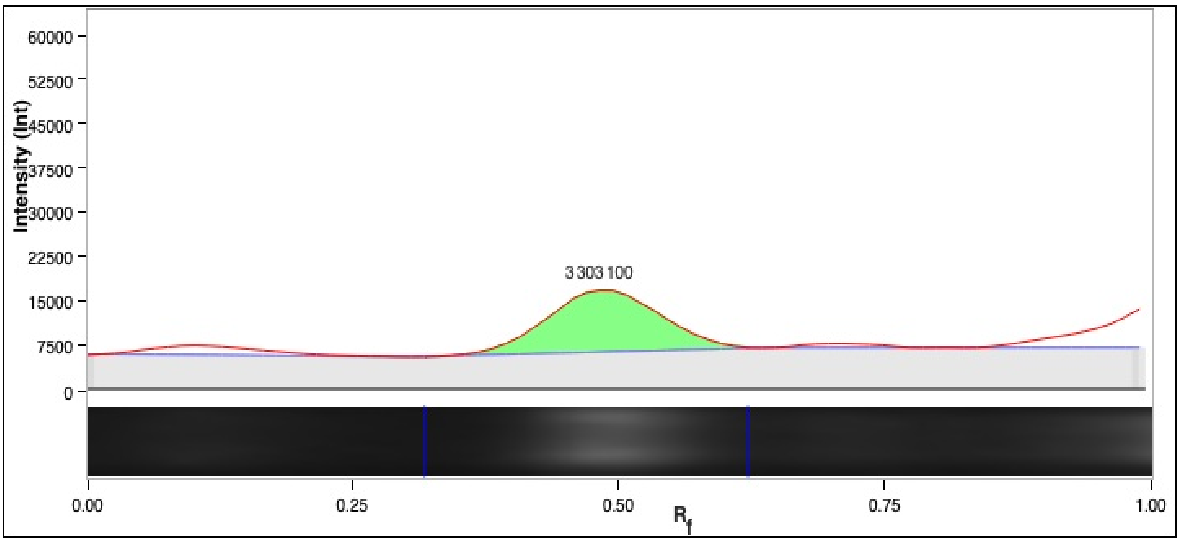

Band No.	Band Label	Mol. Wt. (KDa)	Relative Front	Adj. Volume (Int)	Volume (Int)	Abs. Quant.	Rel. Quant.	Band %	Lane %
1		N/A	0,494	3 303 100	8 219 905	N/A	N/A	100,0	67,3

Lane Background	Lane background subtracted with disk size: 0.1
Lane Width	0.41 mm

Lane 10

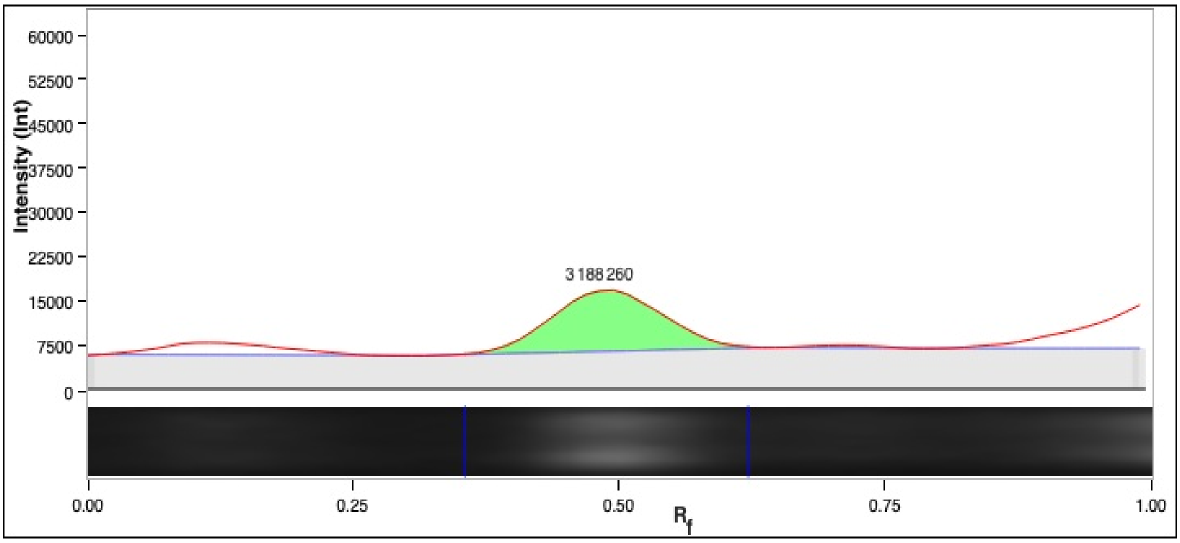

Band No.	Band Label	Mol. Wt. (KDa)	Relative Front	Adj. Volume (Int)	Volume (Int)	Abs. Quant.	Rel. Quant.	Band %	Lane %

1		N/A	0,494	3 188 260	7 643 820	N/A	N/A	100,0	61,1

Lane Background	Lane background subtracted with disk size: 0.1
Lane Width	0.41 mm

Lane 11

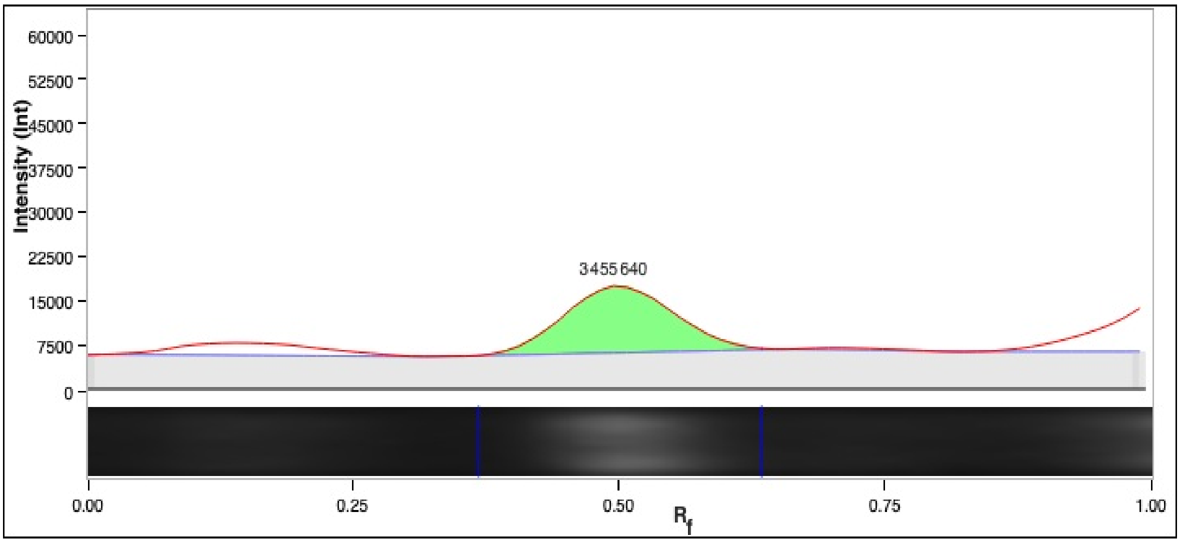

Band No.	Band Label	Mol. Wt. (KDa)	Relative Front	Adj. Volume (Int)	Volume (Int)	Abs. Quant.	Rel. Quant.	Band %	Lane %
1		N/A	0,506	3 455 640	7 745 552	N/A	N/A	100,0	64,0

Lane Background	Lane background subtracted with disk size: 0.1
Lane Width	0.41 mm

Lane 12

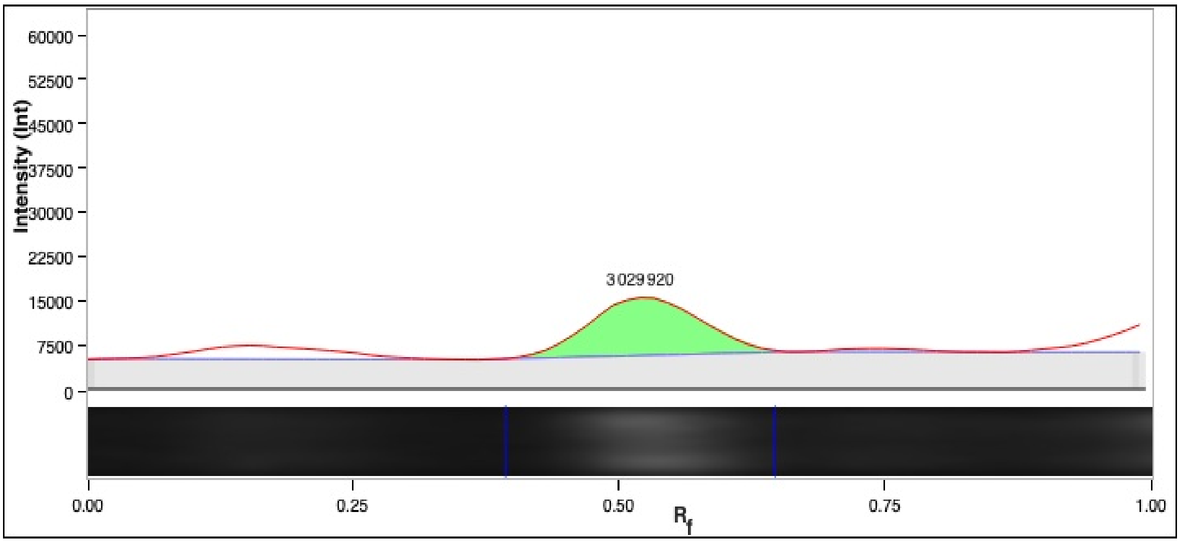

Band No.	Band	Mol. Wt.	Relative	Adj.	Volume	Abs.	Rel.	Band %	Lane %

	Label	(KDa)	Front	Volume (Int)	(Int)	Quant.	Quant.		
1		N/A	0,532	3 029 920	6 806 793	N/A	N/A	100,0	65,4

Lane Background	Lane background subtracted with disk size: 0.1
Lane Width	0.41 mm

Lane 13

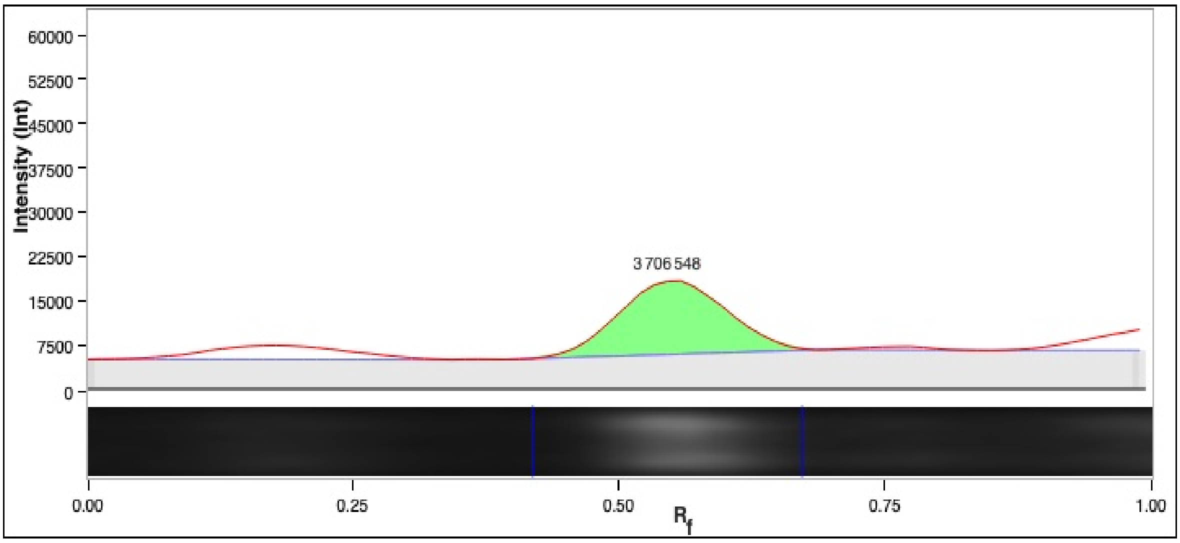

Band No.	Band Label	Mol. Wt. (KDa)	Relative Front	Adj. Volume (Int)	Volume (Int)	Abs. Quant.	Rel. Quant.	Band %	Lane %
1		N/A	0,557	3 706 548	7 598 667	N/A	N/A	100,0	69,8

Lane Background	Lane background subtracted with disk size: 0.1
Lane Width	0.41 mm

Lane 14

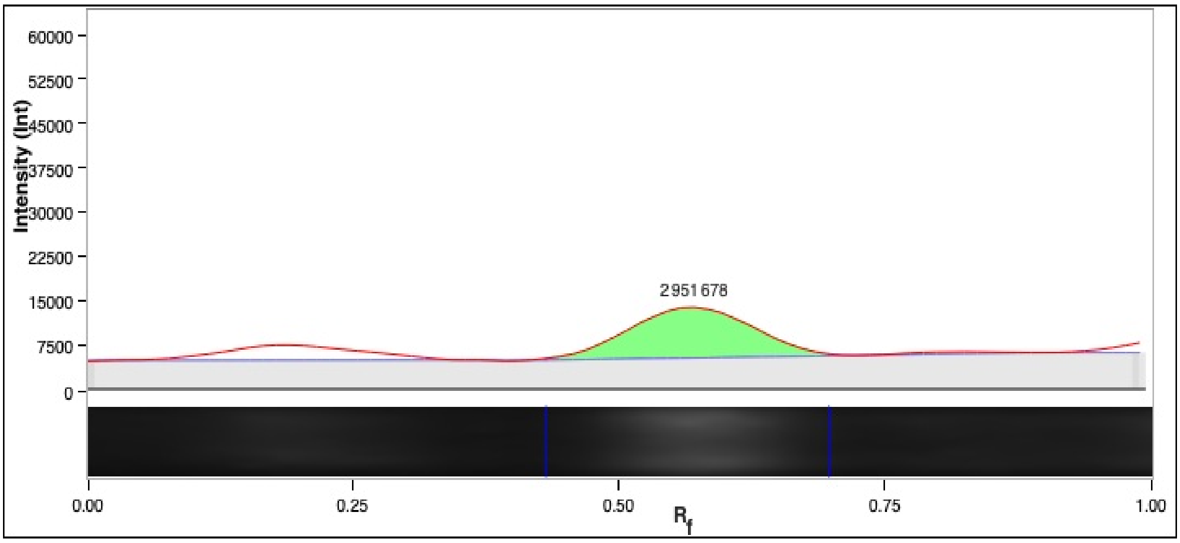

Band No.	Band Label	Mol. Wt. (KDa)	Relative Front	Adj. Volume (Int)	Volume (Int)	Abs. Quant.	Rel. Quant.	Band %	Lane %
1		N/A	0,582	2 951 678	6 619 047	N/A	N/A	100,0	70,4

Lane Background	Lane background subtracted with disk size: 0.1
Lane Width	0.41 mm

Lane 15

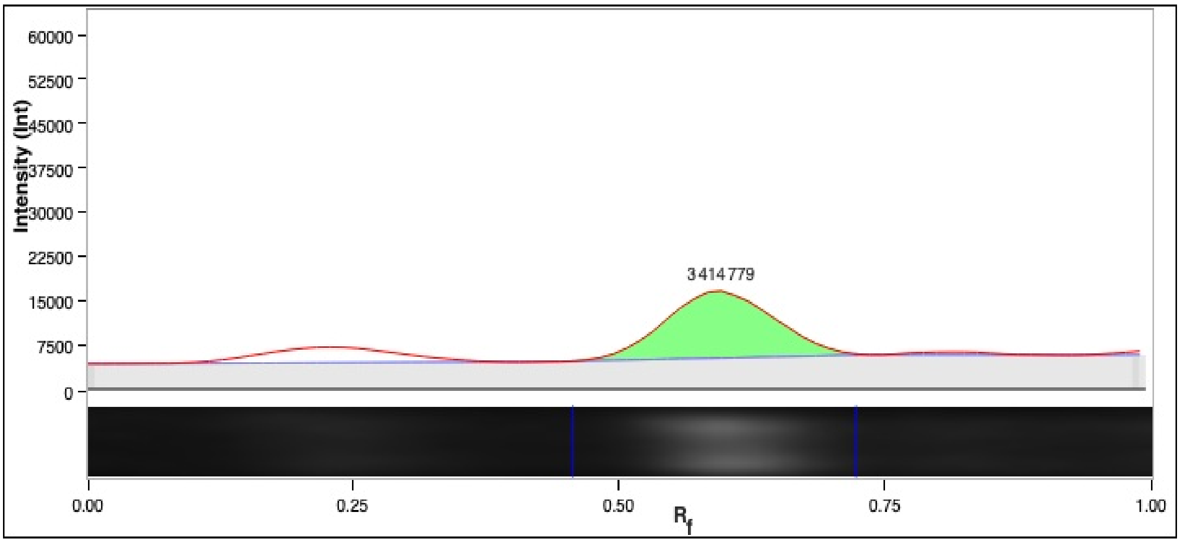

Band No.	Band Label	Mol. Wt. (KDa)	Relative Front	Adj. Volume (Int)	Volume (Int)	Abs. Quant.	Rel. Quant.	Band %	Lane %
1		N/A	0,608	3 414 779	7 060 949	N/A	N/A	100,0	74,2

Lane Background	Lane background subtracted with disk size: 0.1
Lane Width	0.41 mm

Lane 16

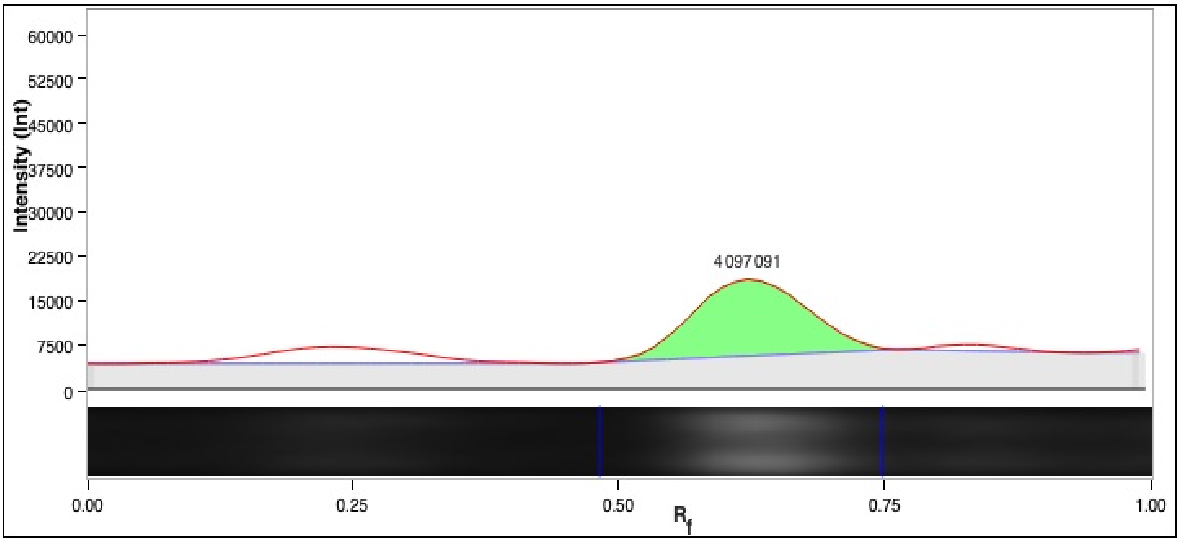

Band No.	Band Label	Mol. Wt. (KDa)	Relative Front	Adj. Volume (Int)	Volume (Int)	Abs. Quant.	Rel. Quant.	Band %	Lane %
1		N/A	0,633	4 097 091	7 934 980	N/A	N/A	100,0	73,9

Lane Background	Lane background subtracted with disk size: 0.1
Lane Width	0.41 mm

Lane 17

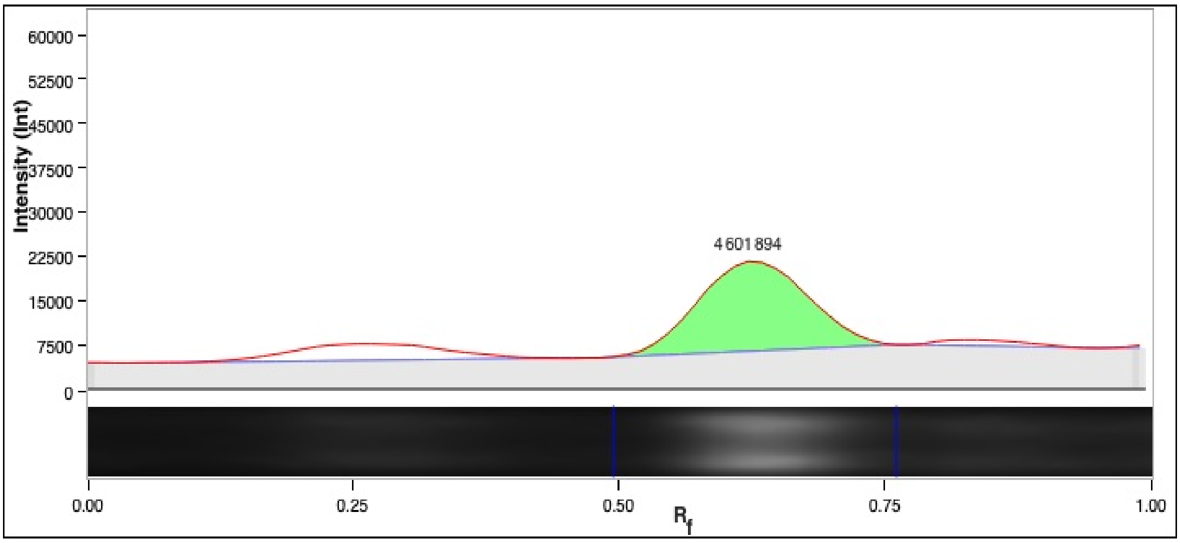

Band No.	Band Label	Mol. Wt. (KDa)	Relative Front	Adj. Volume (Int)	Volume (Int)	Abs. Quant.	Rel. Quant.	Band %	Lane %
1		N/A	0,633	4 601 894	9 106 522	N/A	N/A	100,0	75,6

Lane Background	Lane background subtracted with disk size: 0.1
Lane Width	0.41 mm

Lane 18

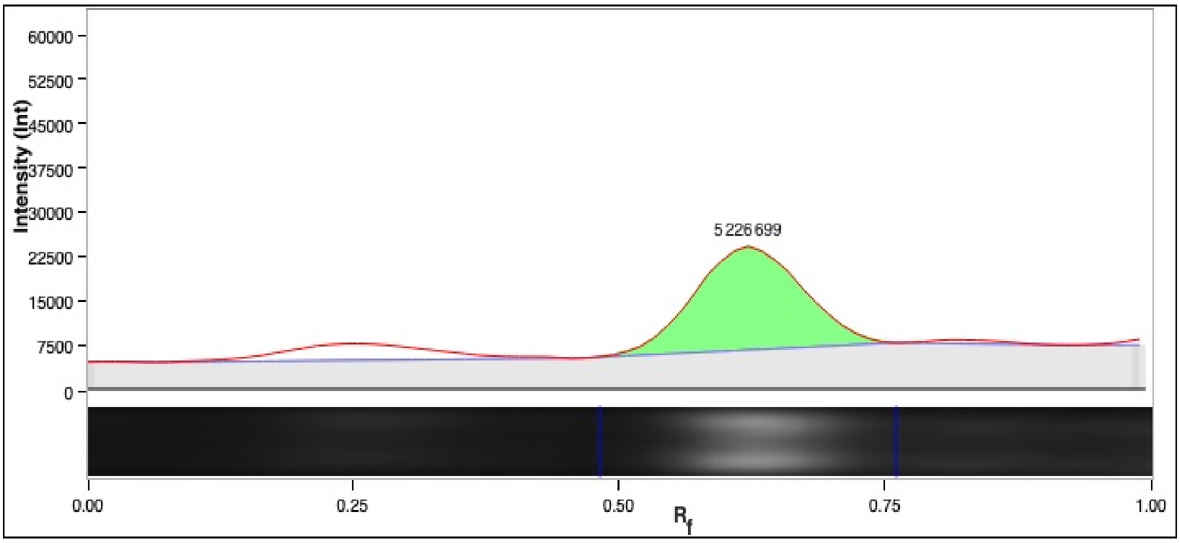

Band No.	Band Label	Mol. Wt. (KDa)	Relative Front	Adj. Volume (Int)	Volume (Int)	Abs. Quant.	Rel. Quant.	Band %	Lane %
1		N/A	0,633	5 226 699	10 050 849	N/A	N/A	100,0	78,9

Lane Background	Lane background subtracted with disk size: 0.1
Lane Width	0.41 mm

Lane 19

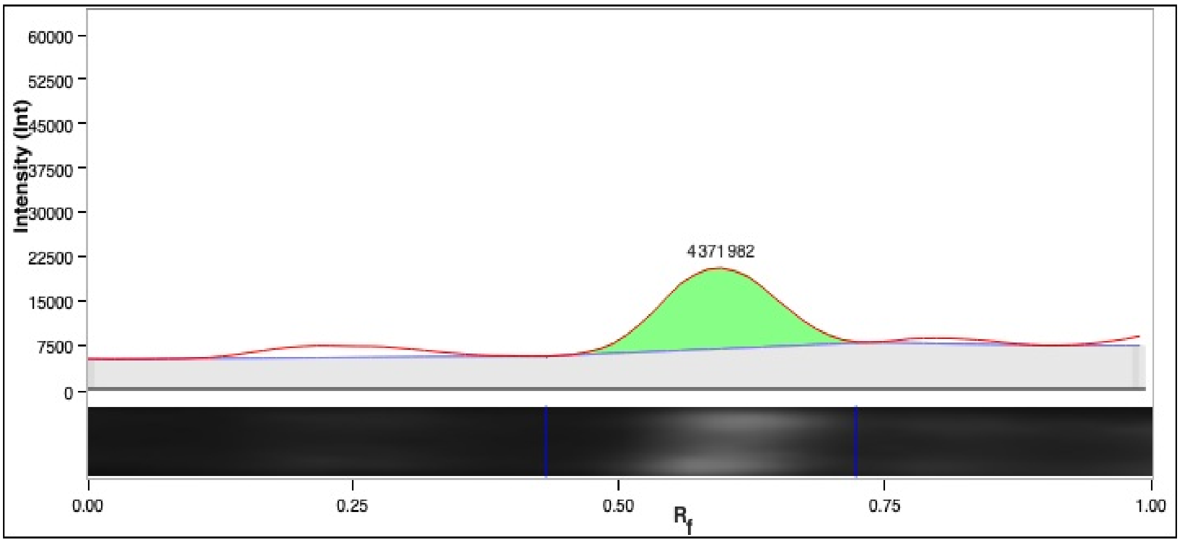

Band No.	Band Label	Mol. Wt. (KDa)	Relative Front	Adj. Volume (Int)	Volume (Int)	Abs. Quant.	Rel. Quant.	Band %	Lane %
1		N/A	0,608	4 371 982	9 459 394	N/A	N/A	100,0	78,1

Lane Background	Lane background subtracted with disk size: 0.1
Lane Width	0.41 mm

Lane 20

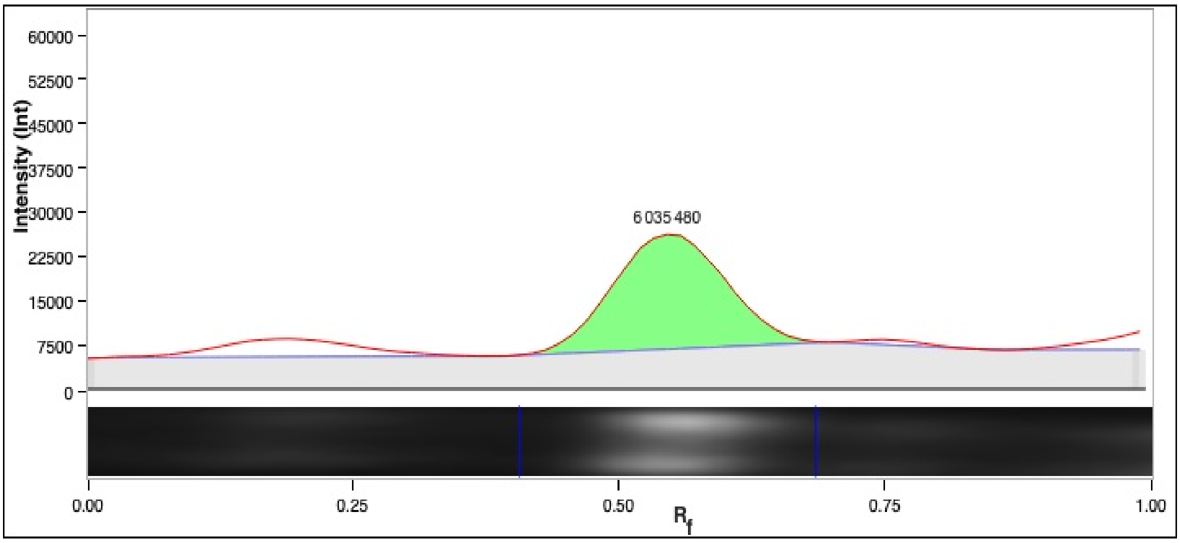

Band No.	Band Label	Mol. Wt. (KDa)	Relative Front	Adj. Volume (Int)	Volume (Int)	Abs. Quant.	Rel. Quant.	Band %	Lane %
1		N/A	0,557	6 035 480	10 968 322	N/A	N/A	100,0	77,4

Lane Background	Lane background subtracted with disk size: 0.1
Lane Width	0.41 mm

Lane 21

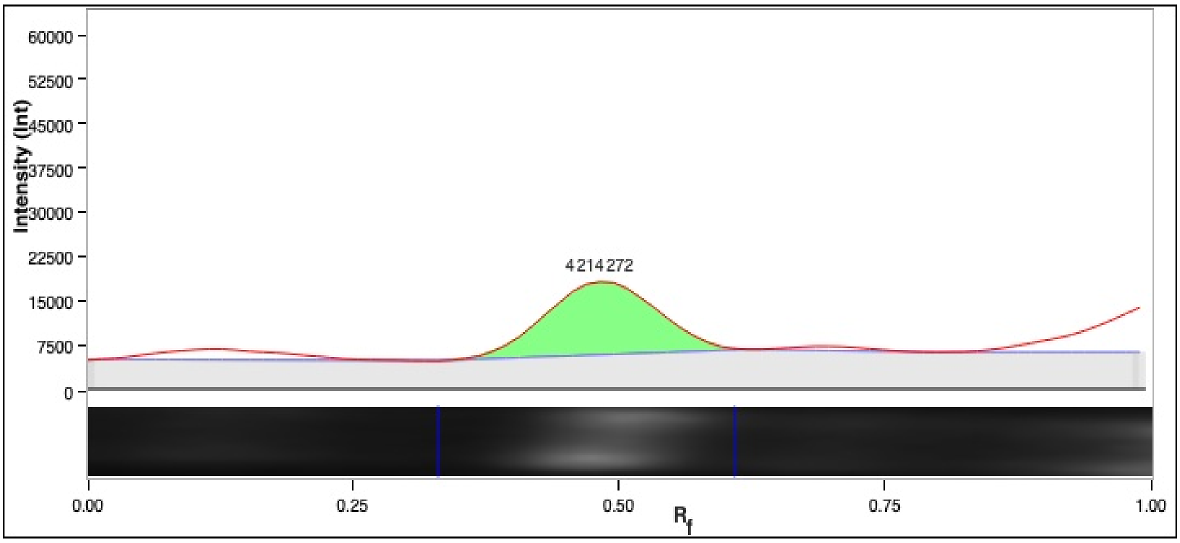

Band No.	Band Label	Mol. Wt. (KDa)	Relative Front	Adj. Volume (Int)	Volume (Int)	Abs. Quant.	Rel. Quant.	Band %	Lane %
1		N/A	0,494	4 214 272	8 814 272	N/A	N/A	100,0	65,3

Lane Background	Lane background subtracted with disk size: 0.1
Lane Width	0.45 mm
