# Supplementary figures and images for: In silico modeling guides identification of novel JAK1 variants associated with immune dysregulation
Source: EMBO Mol Med. 2025 Oct 24;17(12):3275–99. doi: 10.1038/s44321-025-00317-0 (PMC12686074; doi:10.1038/s44321-025-00317-0)

## Slide 1
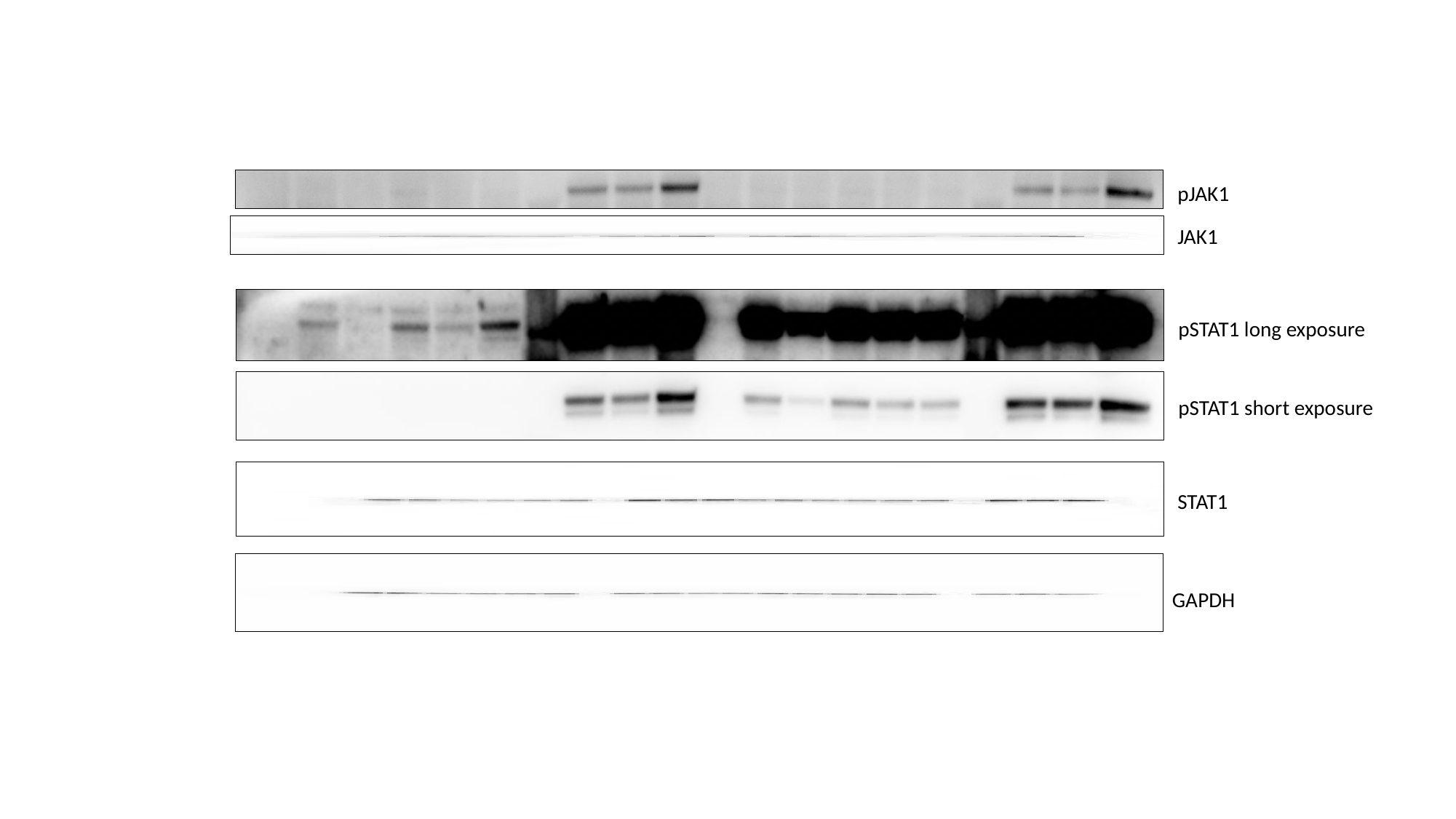

pJAK1
JAK1
pSTAT1 long exposure
pSTAT1 short exposure
STAT1
GAPDH

Supplement: Supplementary file 8 — Source data Fig. 3 [file 44321_2025_317_MOESM8_ESM.zip › Figure 3/Replicates Fig.3A/n = 2/n2.pptx]

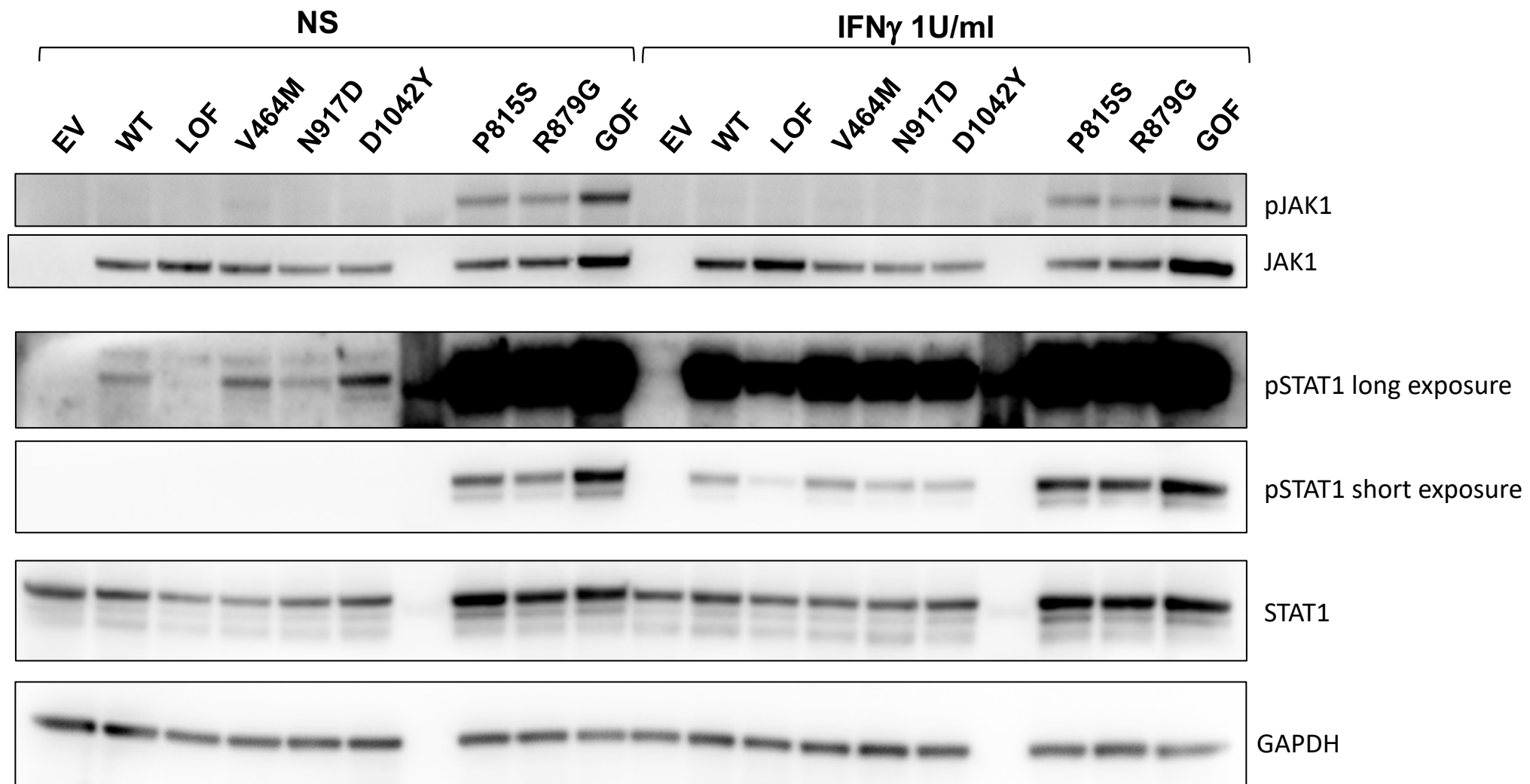

Supplement: Supplementary file 8 — Source data Fig. 3 [file 44321_2025_317_MOESM8_ESM.zip › Figure 3/Replicates Fig.3A/n = 2/n2.pdf]

## Slide 1
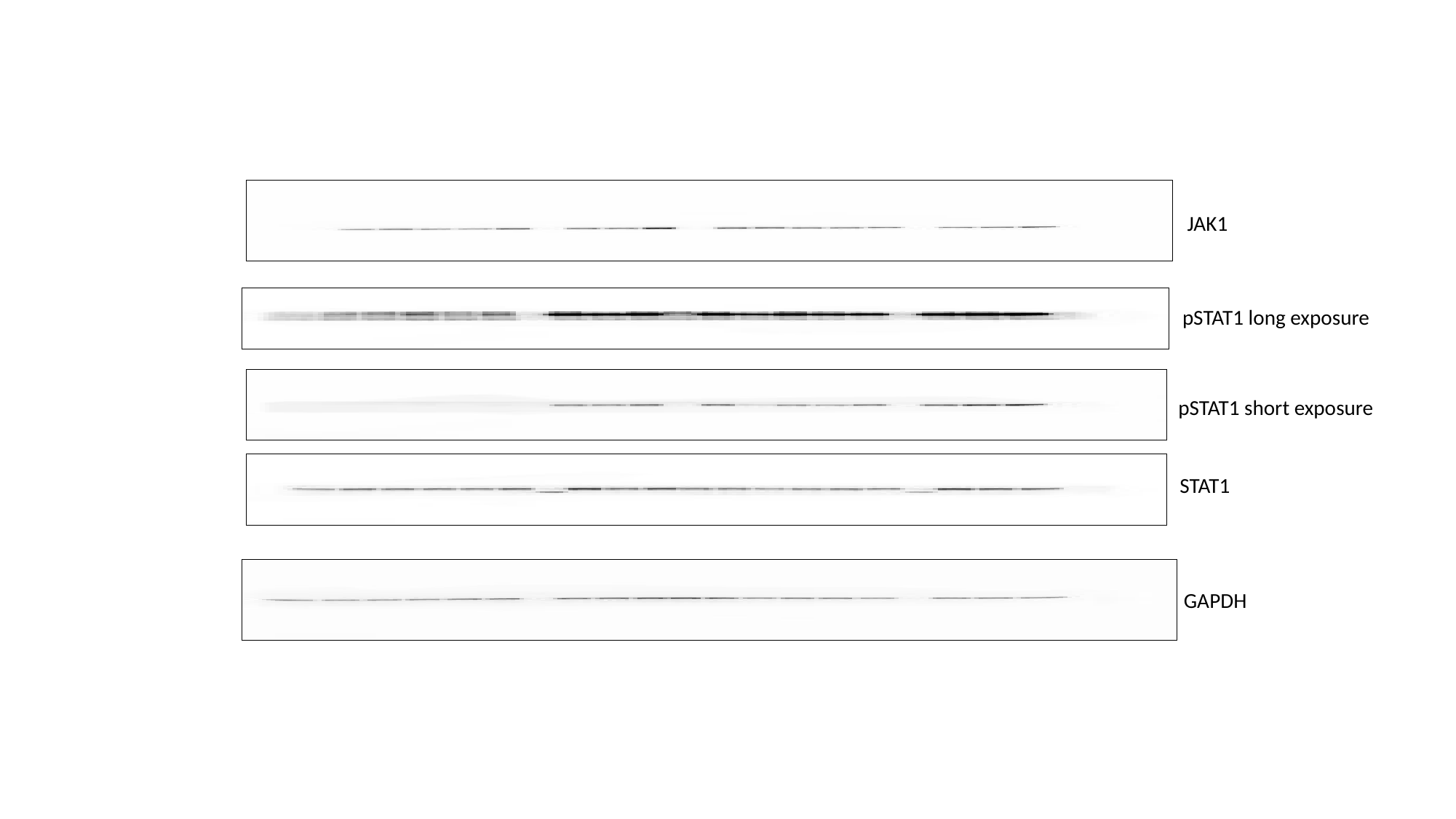

JAK1
pSTAT1 long exposure
pSTAT1 short exposure
STAT1
GAPDH

Supplement: Supplementary file 8 — Source data Fig. 3 [file 44321_2025_317_MOESM8_ESM.zip › Figure 3/Replicates Fig.3A/n = 3/n3.pptx]

## Slide 1
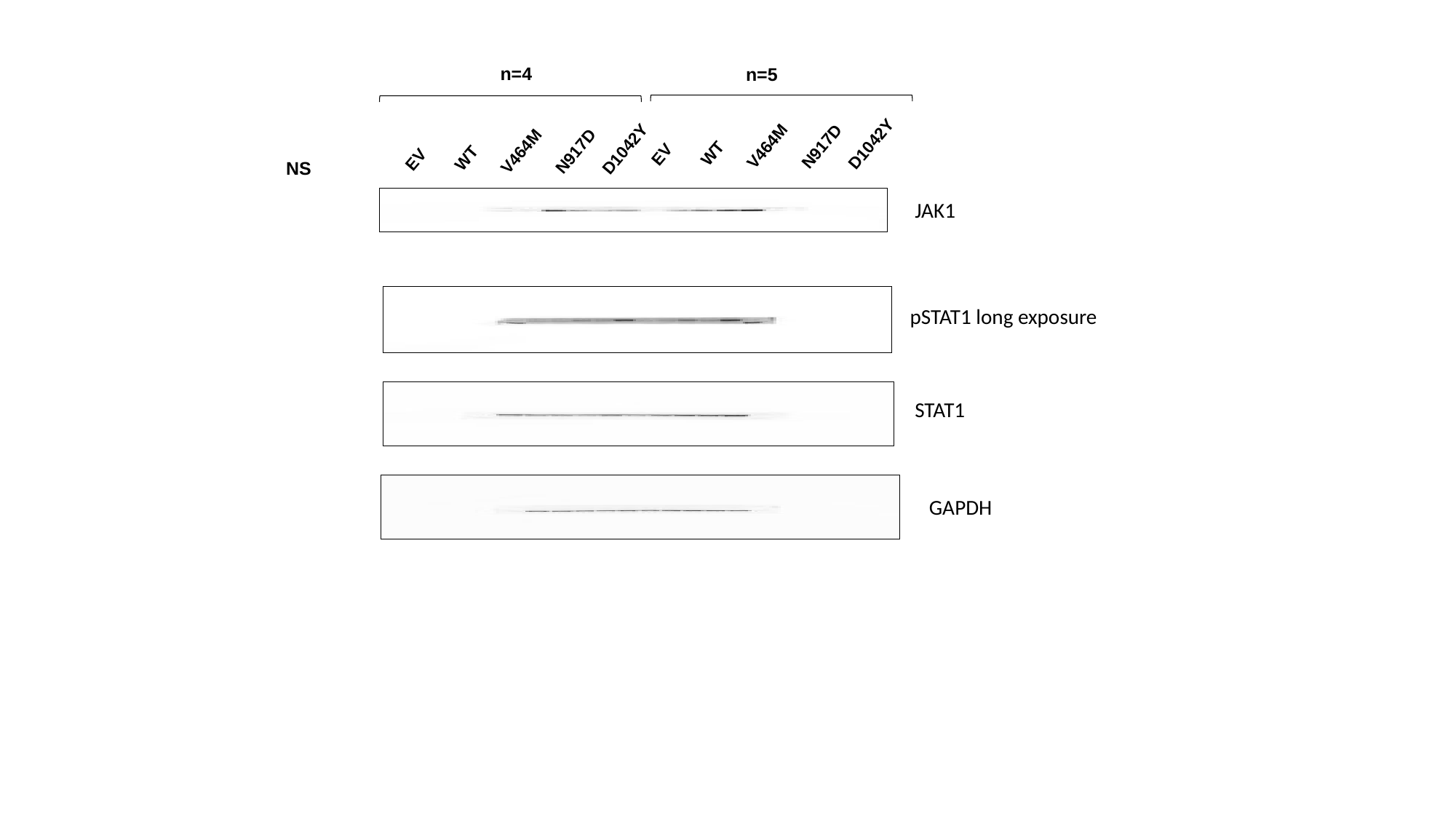

n=4
n=5
D1042Y
V464M
N917D
D1042Y
V464M
N917D
WT
EV
WT
EV
NS
JAK1
pSTAT1 long exposure
STAT1
GAPDH

Supplement: Supplementary file 8 — Source data Fig. 3 [file 44321_2025_317_MOESM8_ESM.zip › Figure 3/Replicates Fig.3A/n = 4 & 5/n4 & n5.pptx]

NS

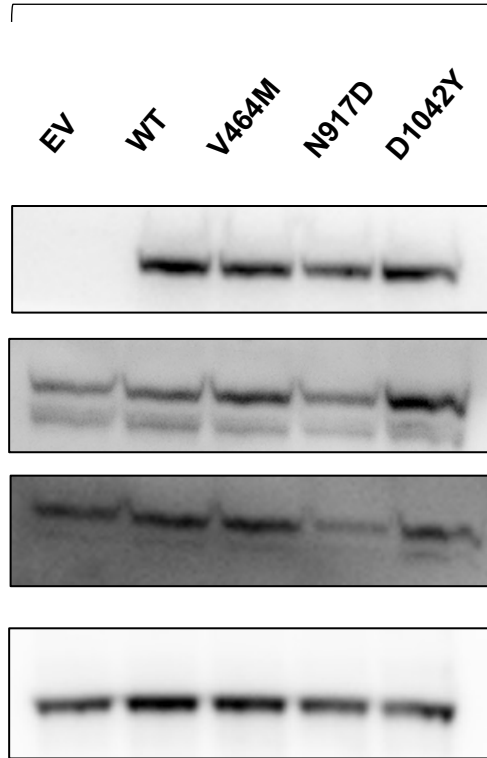

JAK1

pSTAT1 long exposure

STAT1

GAPDH

Supplement: Supplementary file 8 — Source data Fig. 3 [file 44321_2025_317_MOESM8_ESM.zip › Figure 3/Replicates Fig.3A/n = 6/n6.pdf]

## Slide 1
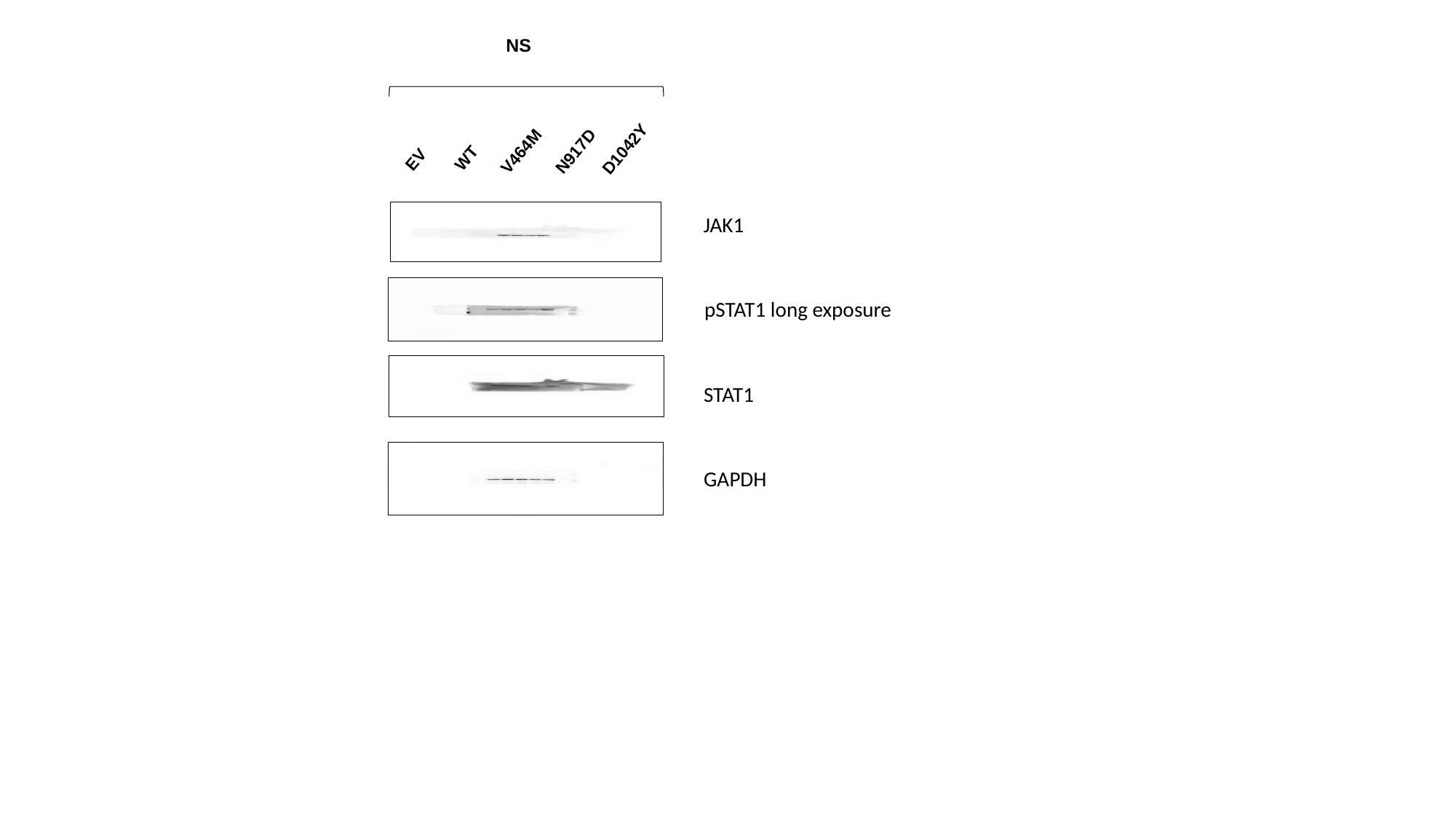

NS
D1042Y
V464M
N917D
WT
EV
JAK1
pSTAT1 long exposure
STAT1
GAPDH

Supplement: Supplementary file 8 — Source data Fig. 3 [file 44321_2025_317_MOESM8_ESM.zip › Figure 3/Replicates Fig.3A/n = 6/n6.pptx]

## Slide 1
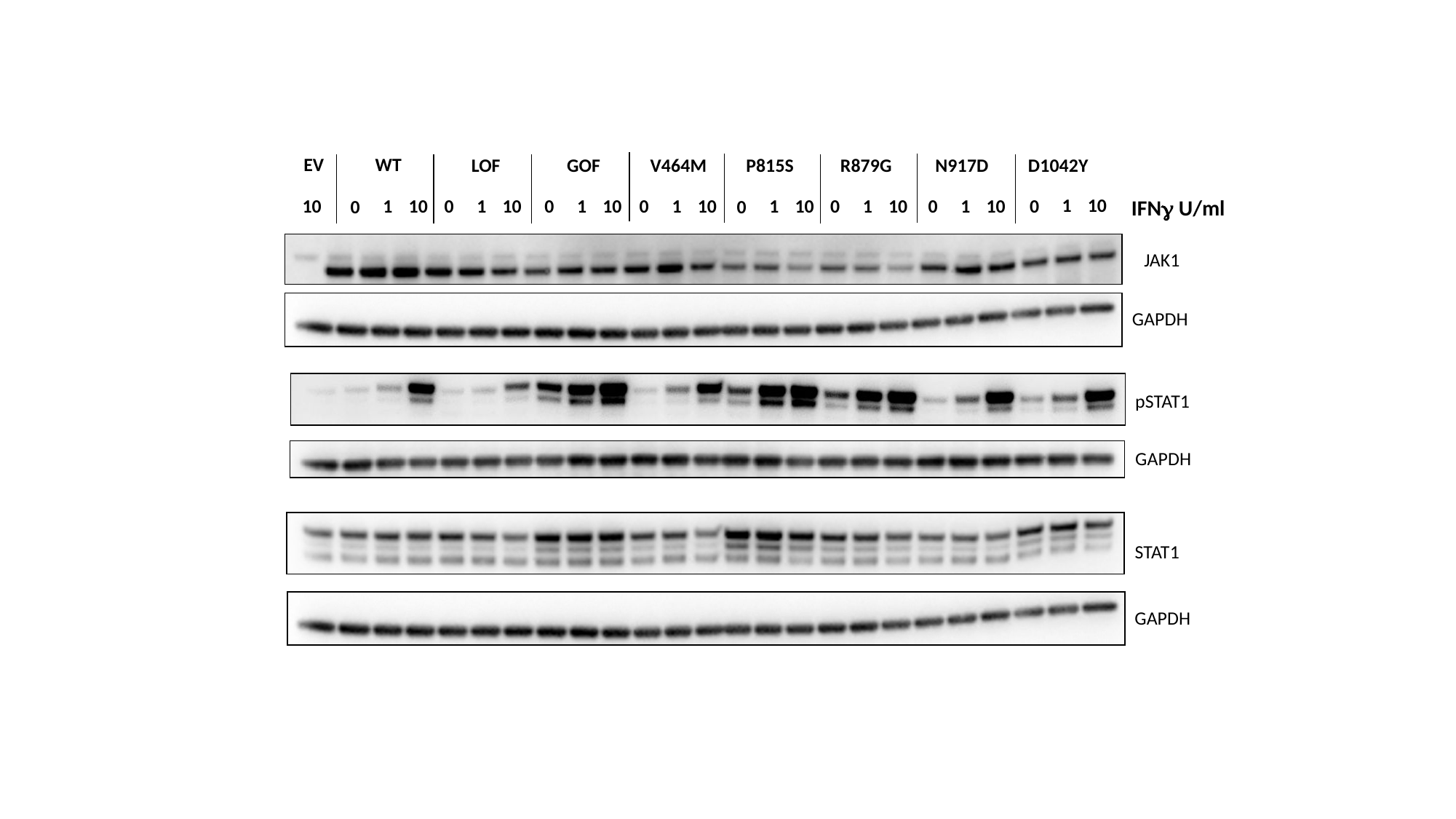

EV
WT
V464M
N917D
LOF
P815S
GOF
R879G
D1042Y
1
10
0
1
10
1
10
1
10
1
10
1
10
0
10
1
10
0
0
0
1
10
0
IFNγ U/ml
0
0
JAK1
GAPDH
pSTAT1
GAPDH
STAT1
GAPDH

Supplement: Supplementary file 8 — Source data Fig. 3 [file 44321_2025_317_MOESM8_ESM.zip › Figure 3/Replicates Fig.3A/wb 1/Western 1.pptx]

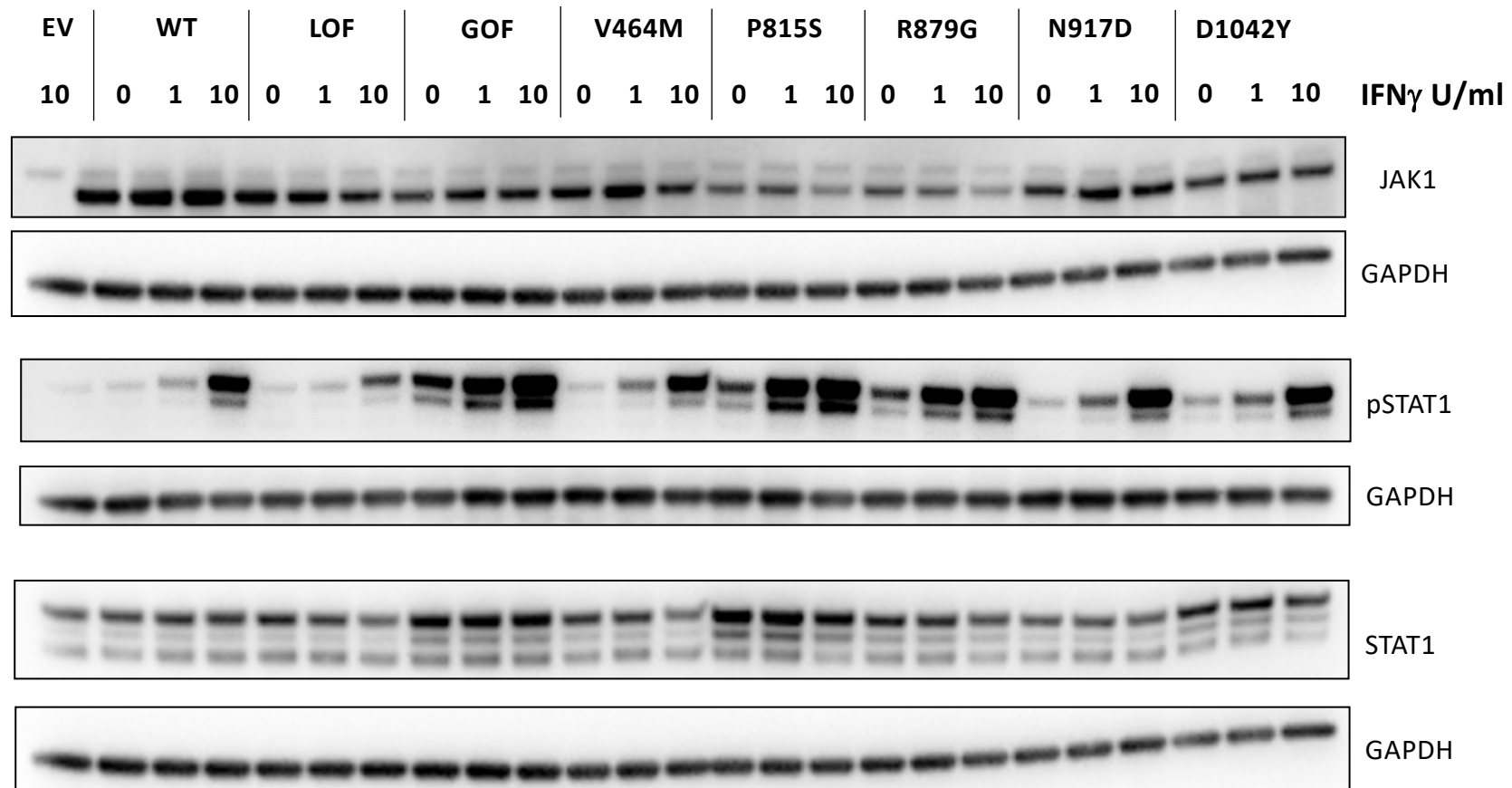

Supplement: Supplementary file 8 — Source data Fig. 3 [file 44321_2025_317_MOESM8_ESM.zip › Figure 3/Replicates Fig.3A/wb 1/Western 1.pdf]

## Slide 1
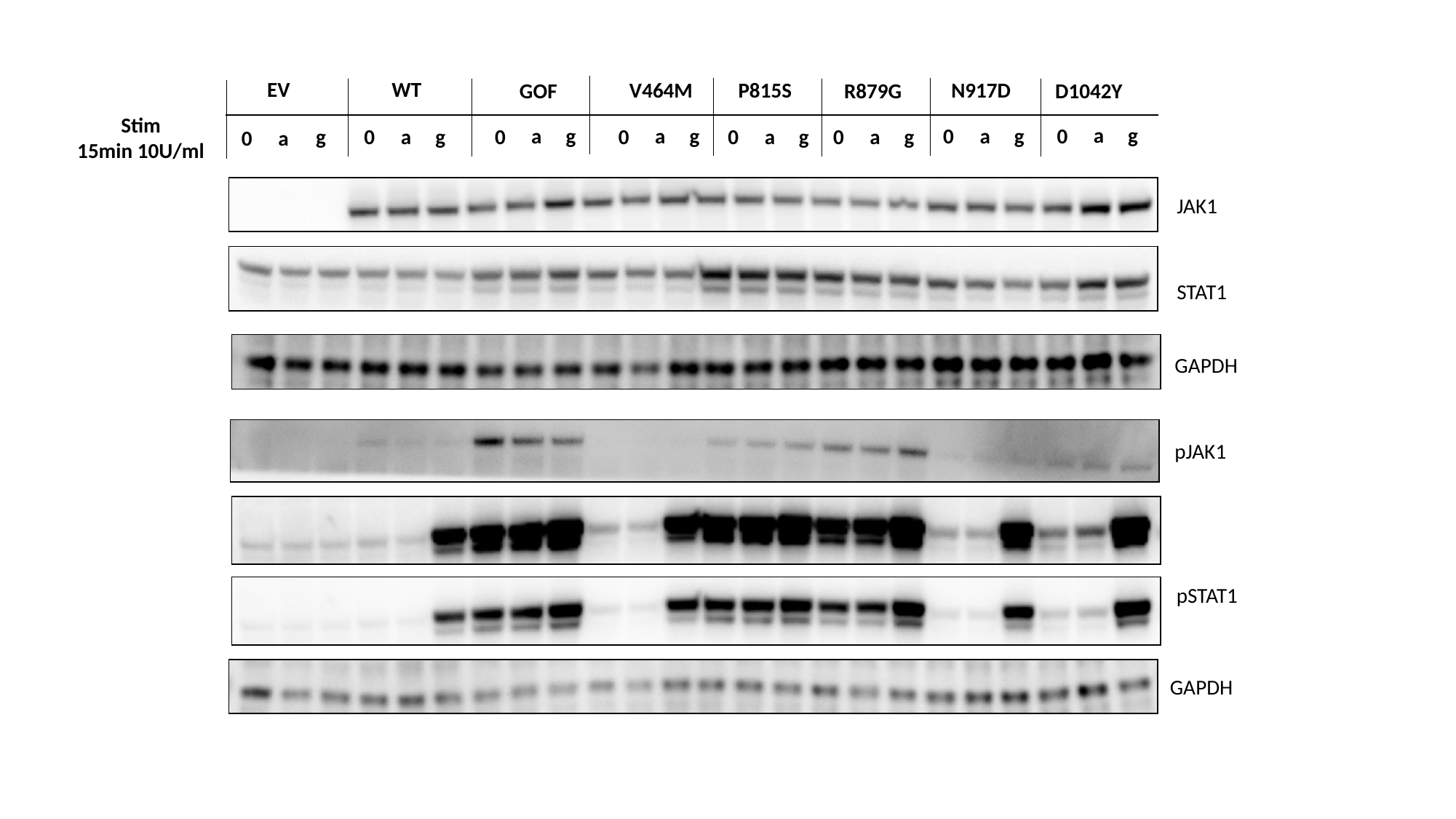

EV
WT
V464M
N917D
P815S
GOF
R879G
D1042Y
Stim
15min 10U/ml
a
g
0
a
g
a
g
a
g
0
g
a
g
0
0
a
g
a
g
0
0
0
a
0
JAK1
STAT1
GAPDH
pJAK1
pSTAT1
GAPDH

Supplement: Supplementary file 8 — Source data Fig. 3 [file 44321_2025_317_MOESM8_ESM.zip › Figure 3/Replicates Fig.3A/wb 5/Western 5.pptx]

Stim  
15min 10U/ml

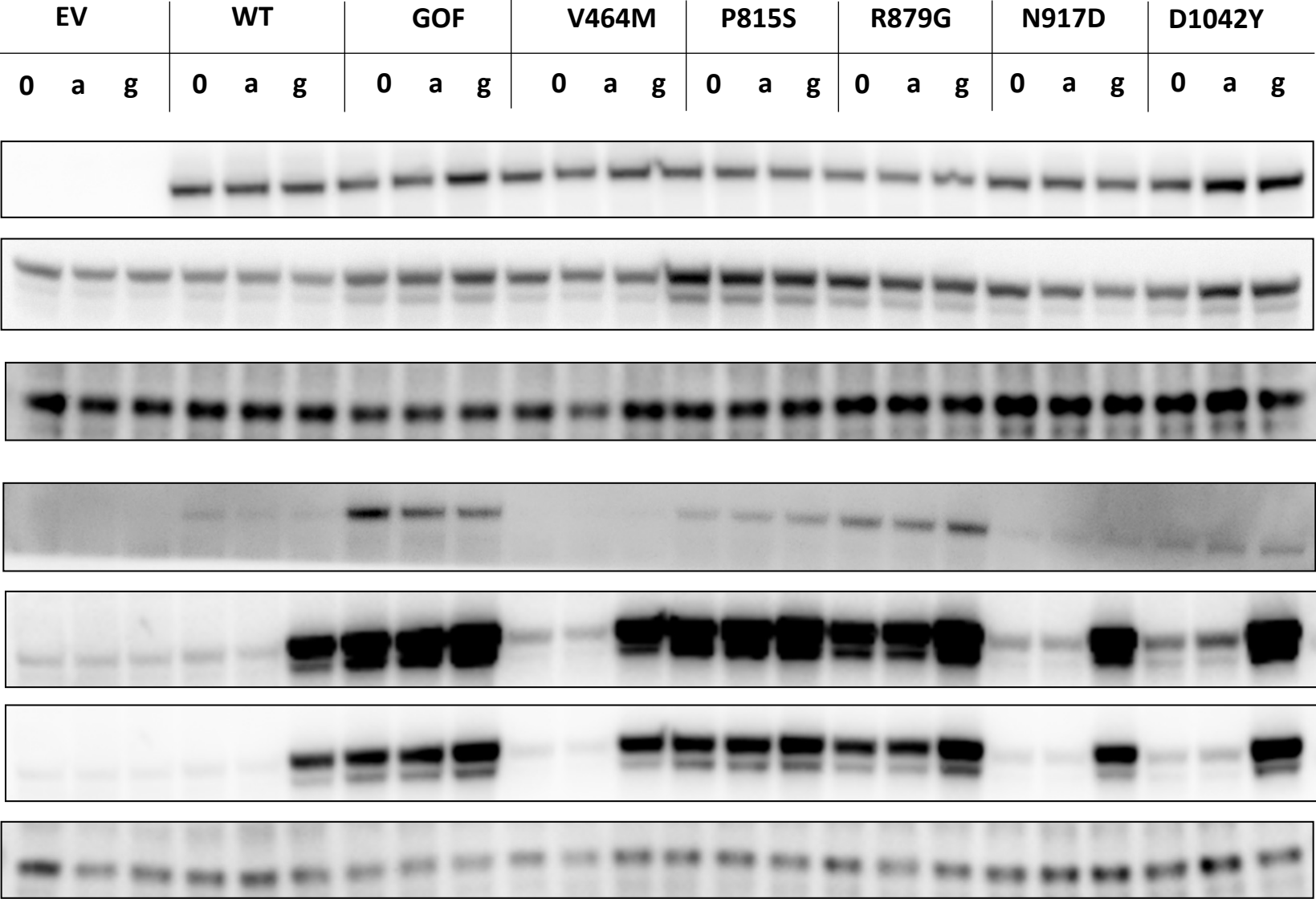

JAK1

STAT1

GAPDH

pJAK1

pSTAT1

GAPDH

Supplement: Supplementary file 8 — Source data Fig. 3 [file 44321_2025_317_MOESM8_ESM.zip › Figure 3/Replicates Fig.3A/wb 5/Western 5.pdf]
