# Supplementary material for: In silico modeling guides identification of novel JAK1 variants associated with immune dysregulation
Source: EMBO Mol Med. 2025 Oct 24;17(12):3275–99. doi: 10.1038/s44321-025-00317-0 (PMC12686074; doi:10.1038/s44321-025-00317-0)
Supplement: Supplementary file 11 — Expanded View Figures [file 44321_2025_317_MOESM11_ESM.pdf]

# Expanded View Figures

**Figure EV1. Validation of in silico pathogenicity prediction for *JAK1* variants.**

(**A**) Heatmap showing mean cluster values of in silico pathogenicity scores for *JAK1* missense variants. Red indicates higher pathogenic values or negative ΔΔG (protein destabilization), blue indicates lower pathogenic values or positive ΔΔG (protein stabilization). Actual mean values are displayed in each cell. Color scale represents normalized values for visualization (red to blue). (**B**) Histogram showing distribution of gnomAD variants ($n = 287$) across the two clusters. *P* value was computed with Bonferroni-corrected Chi-square test and shown in the figure. (**C**) ISGF3 complex (STAT1, STAT2 and IRF9) luciferase reporter activity in U4C cells co-transfected with a plasmid containing the Interferon-Stimulated Response Element (ISRE) and WT or JAK1 variants at baseline or after stimulation with IFN-α ($10^3$ U/ml for 6 h); $n = 3$. JAK1 variants are grouped on the basis of their cluster affiliation. Error bars represent standard error of the mean.

 © The Author(s)

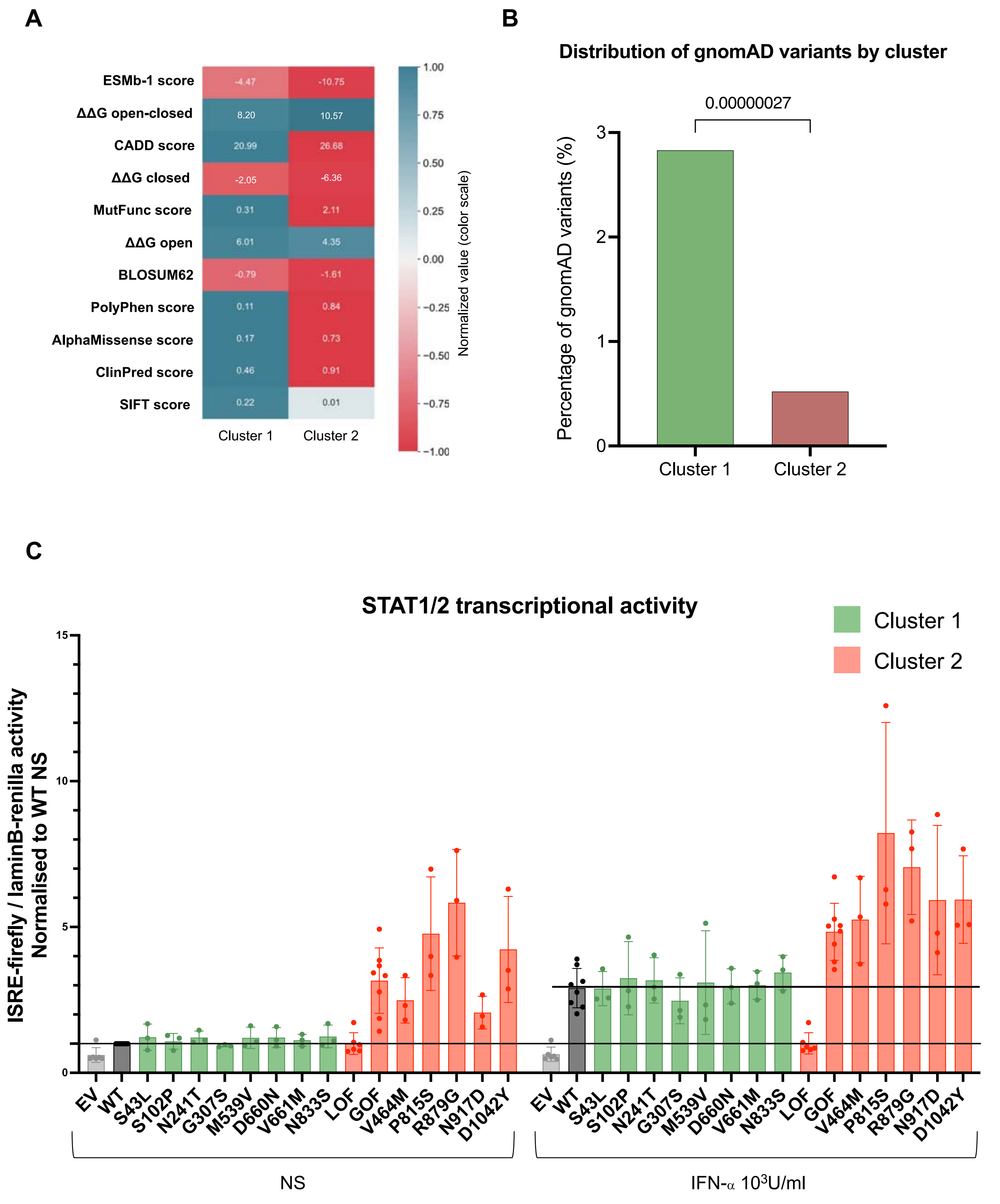
A
ESMb-1 score
ΔΔG open-closed
CADD score
ΔΔG closed
MutFunc score
ΔΔG open
BLOSUM62
PolyPhen score
AlphaMissense score
ClinPred score
SIFT score
Cluster 1
Cluster 2
-4.47
-10.75
8.20
10.57
20.99
26.68
-2.05
-6.36
0.31
2.11
6.01
4.35
-0.79
-1.61
0.11
0.84
0.17
0.73
0.46
0.91
0.22
0.01
Normalized value (color scale)
B
Distribution of gnomAD variants by cluster
0.00000027
Percentage of gnomAD variants (%)
Cluster 1
Cluster 2
C
STAT1/2 transcriptional activity
Cluster 1
Cluster 2
ISRE-firefly / laminB-renilla activity
Normalised to WT NS
EV
WT
S43L
S102P
N241T
G307S
M539V
D660N
V661M
N833S
LOF
GOF
V464M
P815S
R879G
N917D
D1042Y
NS
IFN-α 10³U/ml

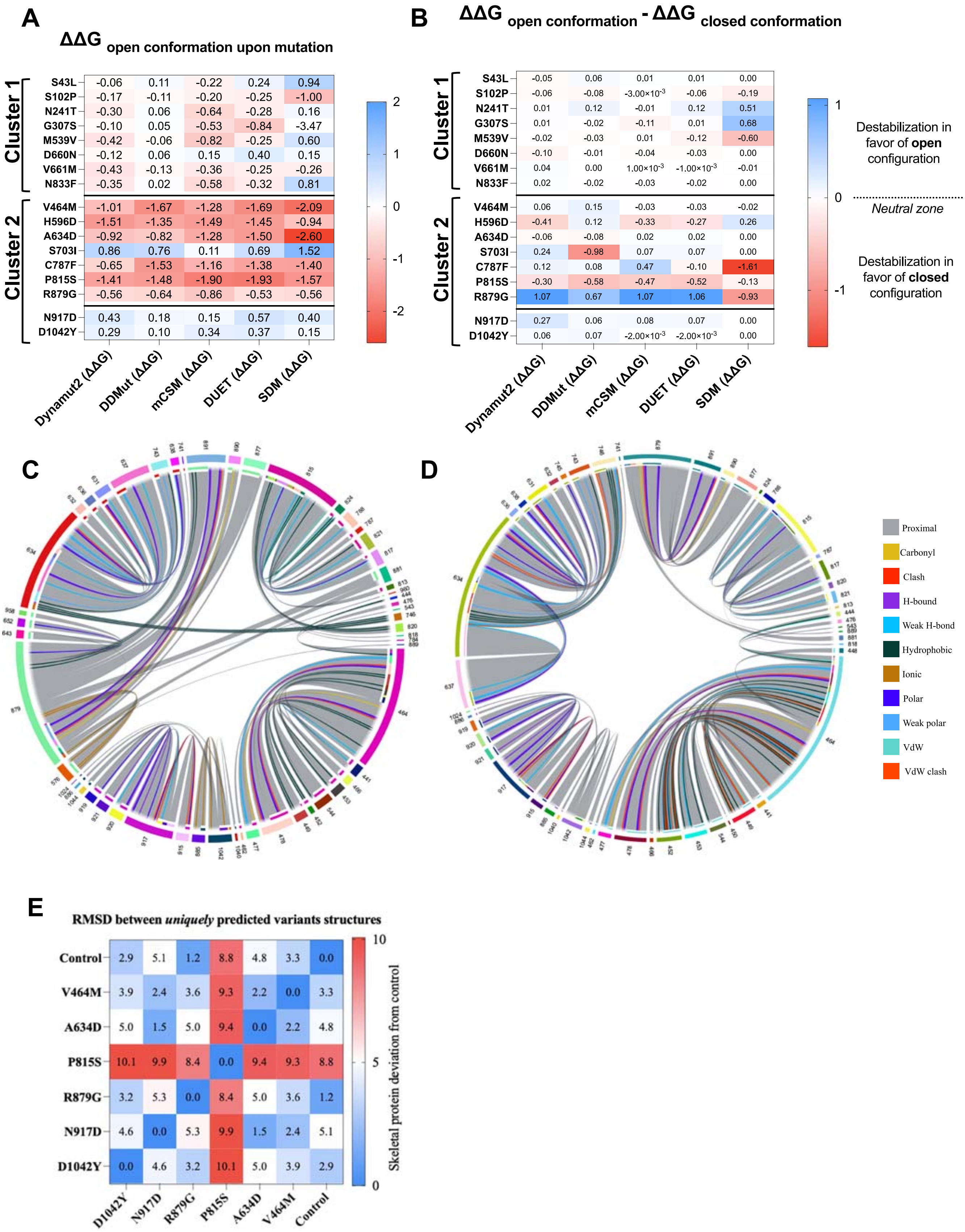
A
ΔΔG open conformation upon mutation
Cluster 1
Cluster 2
S43L -0.06 0.11 -0.22 0.24 0.94
S102P -0.17 -0.11 -0.20 -0.25 -1.00
N241T -0.30 0.06 -0.64 -0.28 0.16
G307S -0.10 0.05 -0.53 -0.84 -3.47
M539V -0.42 -0.06 -0.82 -0.25 0.60
D660N -0.12 0.06 0.15 0.40 0.15
V661M -0.43 -0.13 -0.36 -0.25 -0.26
N833F -0.35 0.02 -0.58 -0.32 0.81
V464M -1.01 -1.67 -1.28 -1.69 -2.09
H596D -1.51 -1.35 -1.49 -1.45 -0.94
A634D -0.92 -0.82 -1.28 -1.50 -2.60
S703I 0.86 0.76 0.11 0.69 1.52
C787F -0.65 -1.53 -1.16 -1.38 -1.40
P815S -1.41 -1.48 -1.90 -1.93 -1.57
R879G -0.56 -0.64 -0.86 -0.53 -0.56
N917D 0.43 0.18 0.15 0.57 0.40
D1042Y 0.29 0.10 0.34 0.37 0.15
Dynamut2 (ΔΔG)
DDMut (ΔΔG)
mCSM (ΔΔG)
DUET (ΔΔG)
SDM (ΔΔG)
2
1
0
-1
-2
B
ΔΔG open conformation - ΔΔG closed conformation
Cluster 1
Cluster 2
S43L -0.05 0.06 0.01 0.01 0.00
S102P -0.06 -0.08 -3.00×10-3 -0.06 -0.19
N241T 0.01 0.12 -0.01 0.12 0.51
G307S 0.01 -0.02 -0.11 0.01 0.68
M539V -0.02 -0.03 0.01 -0.12 -0.60
D660N -0.10 -0.01 -0.04 -0.03 0.00
V661M 0.04 0.00 1.00×10-3 -1.00×10-3 -0.01
N833F 0.02 -0.02 -0.03 -0.02 0.00
V464M 0.06 0.15 -0.03 -0.03 -0.02
H596D -0.41 0.12 -0.33 -0.27 0.26
A634D -0.06 -0.08 0.02 0.02 0.00
S703I 0.24 -0.98 0.07 0.07 0.00
C787F 0.12 0.08 0.47 -0.10 -1.61
P815S -0.30 -0.58 -0.47 -0.52 -0.13
R879G 1.07 0.67 1.07 1.06 -0.93
N917D 0.27 0.06 0.08 0.07 0.00
D1042Y 0.06 0.07 -2.00×10-3 -2.00×10-3 0.00
Dynamut2 (ΔΔG)
DDMut (ΔΔG)
mCSM (ΔΔG)
DUET (ΔΔG)
SDM (ΔΔG)
1
0
-1
Destabilization in favor of open configuration
Neutral zone
Destabilization in favor of closed configuration
C
D
Proximal
Carbonyl
Clash
H-bound
Weak H-bond
Hydrophobic
Ionic
Polar
Weak polar
VdW
VdW clash
E
RMSD between uniquely predicted variants structures
Control 2.9 5.1 1.2 8.8 4.8 3.3 0.0
V464M 3.9 2.4 3.6 9.3 2.2 0.0 3.3
A634D 5.0 1.5 5.0 9.4 0.0 2.2 4.8
P815S 10.1 9.9 8.4 0.0 9.4 9.3 8.8
R879G 3.2 5.3 0.0 8.4 5.0 3.6 1.2
N917D 4.6 0.0 5.3 9.9 1.5 2.4 5.1
D1042Y 0.0 4.6 3.2 10.1 5.0 3.9 2.9
D1042Y
N917D
R879G
P815S
A634D
V464M
Control
10
5
0
Skeletal protein deviation from control

 © The Author(s)

**Figure EV2. Structural insights into uniquely predicted variants.**

(**A**) Predicted stability perturbation (ΔΔG) of JAK1 variants in the open conformation, computed using five different methods (see Appendix Supplementary Methods). (**B**) Comparative analysis of ΔΔG values between open and closed JAK1 conformations, revealing the intrinsic tendency of mutated JAK1 to favor either conformation by minimizing the associated Gibbs free energy costs. Negative values indicate the tendency to adopt the closed conformation, while positive values point to a preference for the open conformation. (**C**, **D**) Chord plots illustrating residue-residue interactions in the JAK1 closed conformation. (**C**) Interactions involving wild-type residues. (**D**) Interactions altered by the variant. Each thread represents an atomic interaction, color-coded by interaction type. (**E**) Heatmap of root-mean-square deviation (RMSD) computed between pair of structures, quantifying the similarity between two superimposed atomic coordinates (see Appendix Supplementary Methods).

© The Author(s)

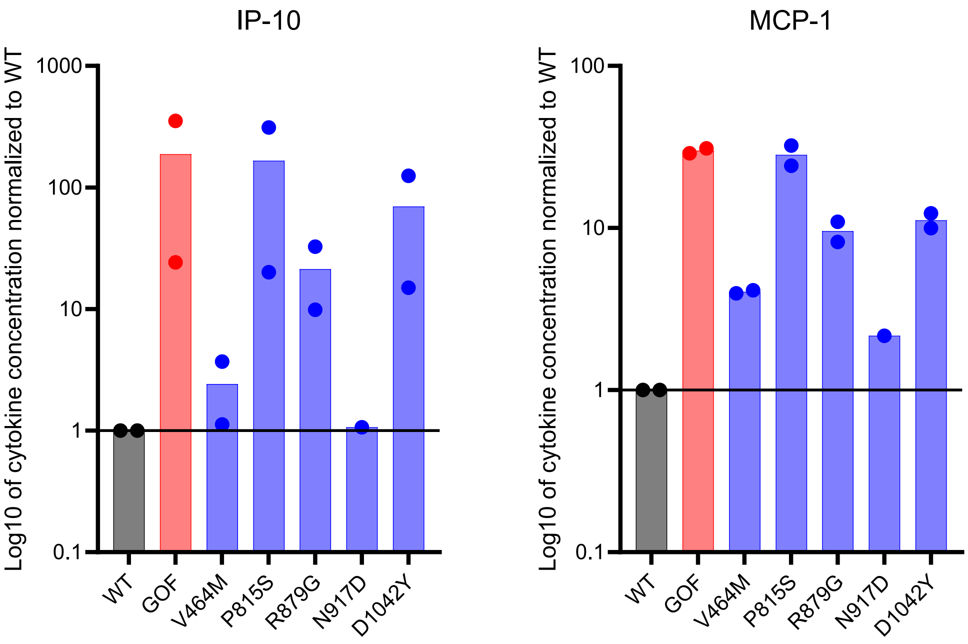


**Figure EV3. Inflammatory cytokines secreted by U4C cells expressing *JAK1* variants.**

MCP-1 or IP-10 levels measured in the supernatant of transfected U4C cells 24 h after transfection with WT, or JAK1 GOF (p.A634D), or patient's variants. $n = 2$ independent experiments.

 © The Author(s)

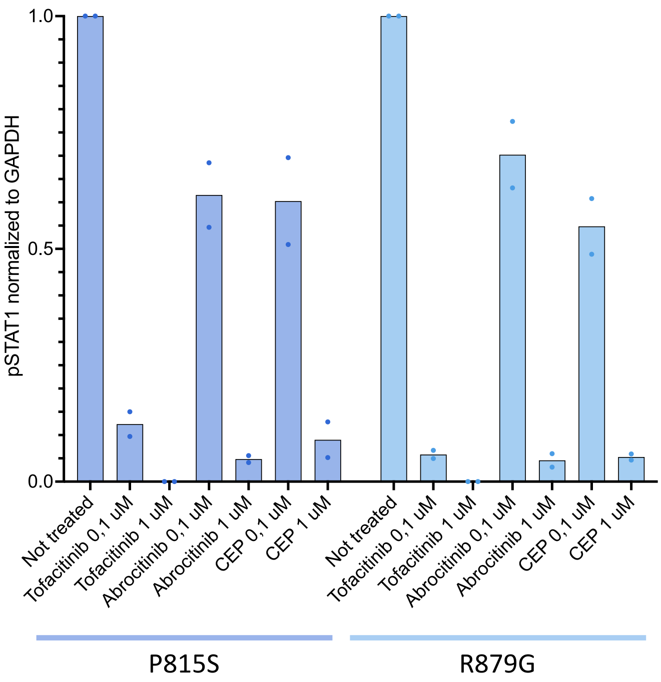


**Figure EV4. Differential effect of JAKinibs on transduced U4C cells.**

Western blot quantification of STAT1 phosphorylation in U4C cells transduced with different *JAK1* variants at baseline and after different JAK inhibitors treatment. pSTAT1 band quantification is normalized to total GAPDH and baseline condition. $n = 2$ independent experiments. Histogram represents the mean.

© The Author(s)
