# Supplementary material for: In silico modeling guides identification of novel JAK1 variants associated with immune dysregulation
Source: EMBO Mol Med. 2025 Oct 24;17(12):3275–99. doi: 10.1038/s44321-025-00317-0 (PMC12686074; doi:10.1038/s44321-025-00317-0)
Supplement: Supplementary file 8 — Source data Fig. 3 [file n4 & n5.pdf › Figure 3/Replicates Fig.3A/n = 4 & 5/n4 & n5.pdf]

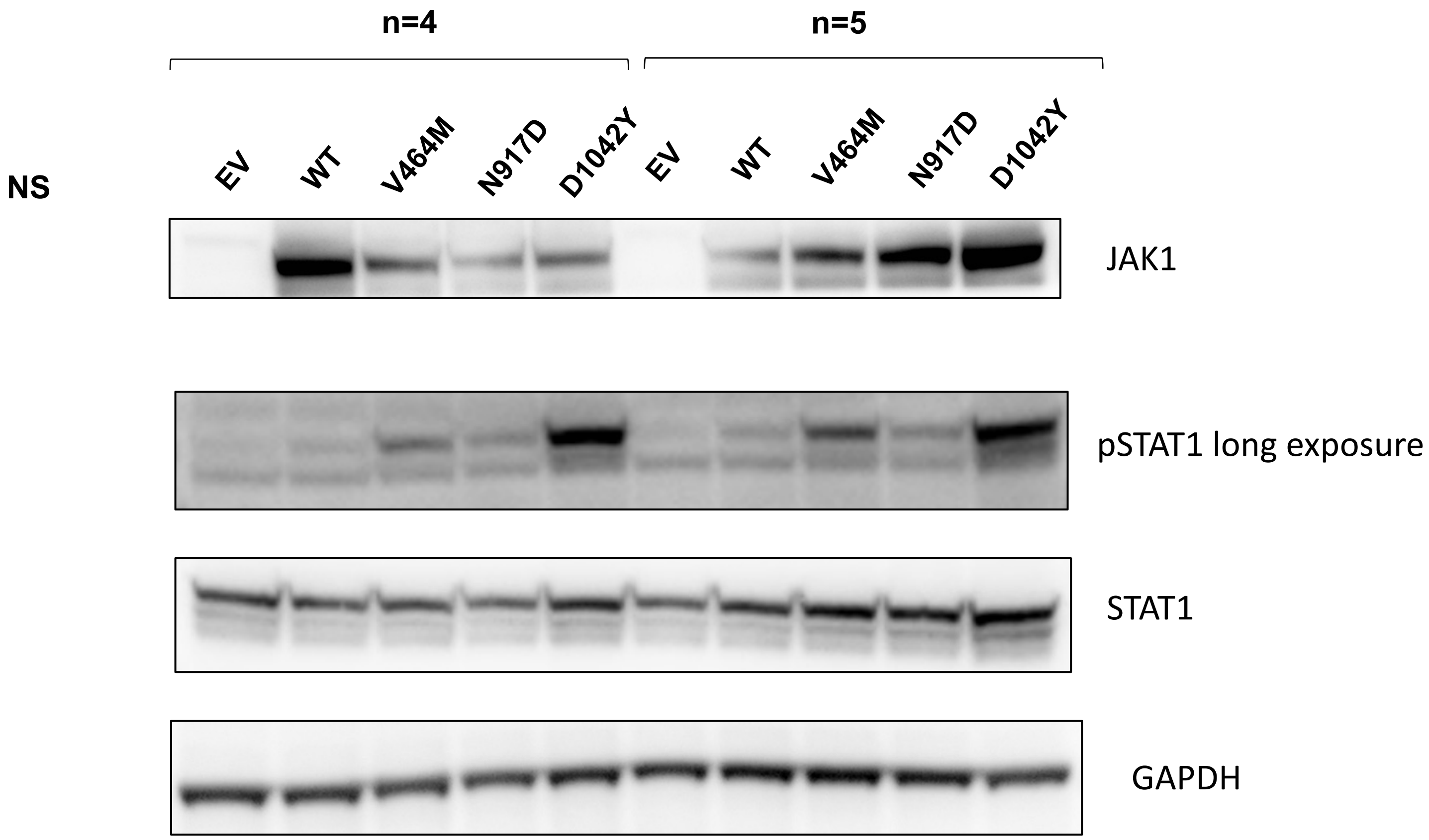
n=4
n=5
NS
EV
WT
V464M
N917D
D1042Y
EV
WT
V464M
N917D
D1042Y
JAK1
pSTAT1 long exposure
STAT1
GAPDH
